# Discovery of 5-Phenylpyrazolopyrimidinone Analogs as Potent Antitrypanosomal Agents with In Vivo Efficacy

**DOI:** 10.1021/acs.jmedchem.3c00161

**Published:** 2023-07-20

**Authors:** Yang Zheng, Magali van den Kerkhof, Tiffany van der Meer, Sheraz Gul, Maria Kuzikov, Bernhard Ellinger, Iwan J. P. de Esch, Marco Siderius, An Matheeussen, Louis Maes, Geert Jan Sterk, Guy Caljon, Rob Leurs

**Affiliations:** † Amsterdam Institute for Molecules, Medicines and Systems, Division of Medicinal Chemistry, Faculty of Science, Vrije Universiteit Amsterdam, De Boelelaan 1108, 1081 HZ Amsterdam, The Netherlands; ‡ Laboratory of Microbiology, Parasitology and Hygiene (LMPH), University of Antwerp, Universiteitsplein 1, 2610 Wilrijk, Belgium; § Fraunhofer Institute for Translational Medicine and Pharmacology ITMP, 22525 Hamburg, Germany; ∥ Fraunhofer Cluster of Excellence for Immune-Mediated Diseases CIMD, 22525 Hamburg, Germany

## Abstract

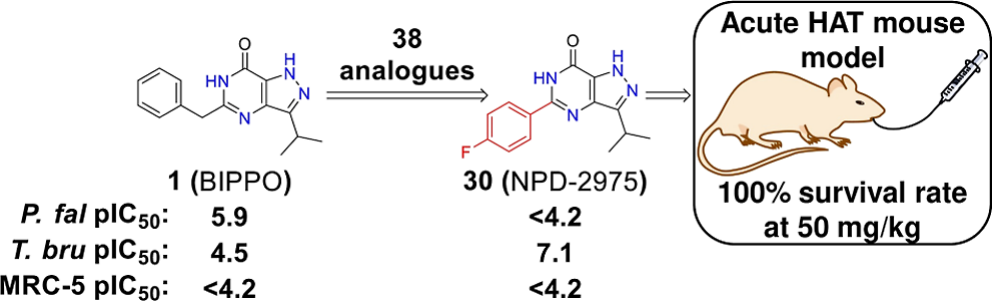

Human African Trypanosomiasis (HAT), caused by *Trypanosoma brucei*, is one of the neglected tropical diseases with a continuing need for new medication. We here describe the discovery of 5-phenylpyrazolopyrimidinone analogs as a novel series of phenotypic antitrypanosomal agents. The most potent compound, **30** (NPD-2975), has an in vitro IC_50_ of 70 nM against *T. b. brucei* with no apparent toxicity against human MRC-5 lung fibroblasts. Showing good physicochemical properties, low toxicity potential, acceptable metabolic stability, and other pharmacokinetic features, **30** was further evaluated in an acute mouse model of *T. b. brucei* infection. After oral dosing at 50 mg/kg twice per day for five consecutive days, all infected mice were cured. Given its good drug-like properties and high in vivo antitrypanosomal potential, the 5-phenylpyrazolopyrimidinone analog **30** represents a promising lead for future drug development to treat HAT.

## Introduction

Human African Trypanosomiasis (HAT), also known as African sleeping sickness, is a neglected parasitic disease (NPD).^1^ Although the detected cases have dropped from 25,000 in 1995 to less than 1000 in 2019, the parasite still represents a risk for 65 million people living in 36 African countries.^2^

The kinetoplastid parasite *Trypanosoma brucei* is the causative agent of HAT^3^ and is transmitted by tsetse flies (*Glossina* sp.). Two subspecies are responsible for the infection in human beings. One of the subspecies, *T. b. gambiense*, causes most human infections (95%) and occurs in Central and West Africa, which is also known as West African trypanosomiasis. While *T. b. rhodesiense* is responsible for infections (5%) in East and Southern Africa, known as East African trypanosomiasis.^4^ HAT develops in two clinical stages.^3^ In the first stage, the parasites multiply in the bloodstream and lymphatic system, causing non-specific symptoms such as headache, fever, and joint pain. Once the parasites penetrate the central nervous system, the disease reaches the second stage, leading to the most common symptom, namely a disturbed sleep pattern. If left untreated, the second stage of HAT will eventually result in a coma or death.^2^

There are only a few treatment options available to control both stages of trypanosomiasis.^5−7^ Thus far, all of them, except fexinidazole (Figure S1), suffer from difficult administration and moderate to severe side effects.^8−10^ As for many infectious diseases, drug resistance often develops, and the most recently approved fexinidazole shares cross-resistance with nifurtimox.^11−13^ Although the clinical trial of a promising new drug, acoziborole (Figure S1), is in progress, it is not easy to reach the goal of HAT elimination by 2030.^14^ Hence, a high urgency to explore novel antitrypanosomal agents remains.

**Figure 1:**
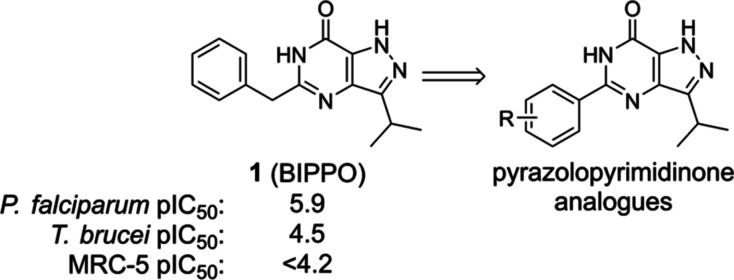
Design of 5-phenylpyrazolopyrimidinone analogs targeting *T. b. brucei*.

Despite this urgency, only a limited number of programs are currently involved in research toward a new HAT treatment.^15−18^ Among them, phosphodiesterases (PDEs) are an important class of targets for HAT drug discovery. PDEs are enzymes that hydrolyze the second messengers cAMP and cGMP to their non-cyclic analogs, AMP and GMP. As these enzymes are found in both humans and parasites, some of them have been proven to be important targets for treating human diseases and potential targets for parasitic diseases.^19−22^ For instance, sildenafil was developed for the treatment of erectile dysfunction by targeting human PDE5.^23^ With abundant knowledge regarding human PDEs, we aimed to develop new antiparasitic treatments via a PDE target-based drug discovery strategy and a phenotypic screening strategy within the EU-funded consortium PDE4NPD (Phosphodiesterase Inhibitors for Neglected Parasitic Diseases).^24^ In 2007, *T. brucei* PDE B1 (*Tbr*PDEB1)^19^ was validated as a target, and in 2018, Blaazer et al.^25^ reported optimization efforts toward *Tbr*PDEB1-selective compounds targeting a parasitic-specific pocket (P-pocket^26^). Besides these target-based efforts, phenotypic screening/evaluation also played an important role. In 2015, BIPPO (**1**, Figure 1) analogs were reported as a novel class of potent anti-*Plasmodium falciparum* agents, potentially acting via their effect on cyclic nucleotide levels.^27^ Considering their antimalarial potency, low molecular weights, good drug-like properties (Table S1), and the fact that these molecules might act as parasite PDE inhibitors, a small additional series of BIPPO analogs (Table 1) was synthesized and phenotypically screened against a panel of protozoan parasites containing *T. b. brucei*, *Trypanosoma cruzi*, and *Leishmania infantum*. This study presents our efforts to characterize novel 5-phenylpyrazolopyrimidinone analogs as potent antitrypanosomal agents with in vivo efficacy.

**Table 1:**
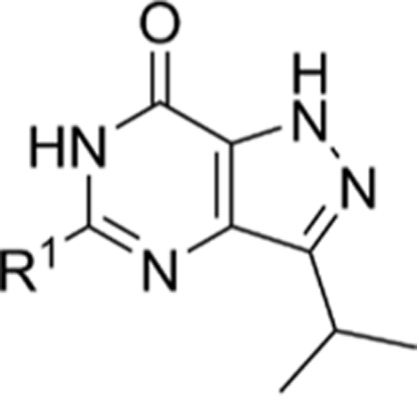
Exploration of SAR in the R^1^ Position

code	R^1^	MW^a^	cLogP^a^	PSA^a^	*T. b. brucei* pIC_50_^b^	MRC-5 pIC_50_^b^
**1** (BIPPO)	Bn	268.3	2.2	70.1	4.5 ± 0.2	<4.2
**9** (NPD-2960)	4-PyCH_2_	269.3	1.0	83.0	<4.2	<4.2
**10** (NPD-0434)	C_6_H_5_OCH_2_	284.3	2.1	79.4	<4.2	<4.2
**11** (NPD-3281)	C_6_H_5_(CH_2_)_2_	282.3	2.3	70.1	<4.2	<4.2
**12** (NPD-3380)	Me	192.2	0.7	70.1	<4.2	<4.2
**13** (NPD-3645)	^*n*^Bu	234.3	2.1	70.1	5.0 ± 0.0	<4.2
**14** (NPD-3379)	^*i*^Pr	220.3	1.8	70.1	4.4 ± 0.1	<4.2
**15** (NPD-3200)	Ph	254.3	2.3	70.1	6.6 ± 0.2	<4.2
**16** (NPD-3488)	4-Py	255.3	1.2	83.0	5.7 ± 0.0	<4.2
**17** (NPD-2973)	4-thiazole	261.3	1.2	83.0	4.9 ± 0.0	<4.2

a cLogP and PSA (polar surface area) are calculated using collaborative drug discovery (CDD).

b Mean values of at least two independent experiments.

## Results

### Chemistry

**Scheme 1:**
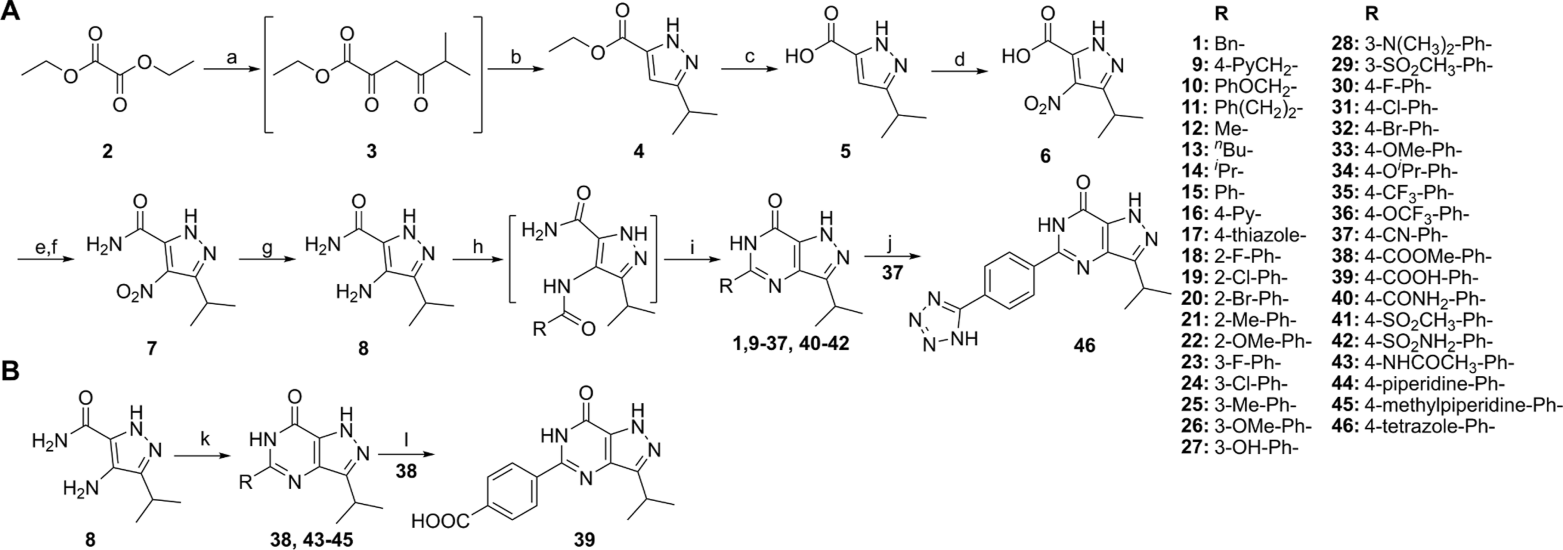
Synthesis of Pyrazolopyrimidinone Analogs Reagents and conditions: (a) 3-methylbutan-2-one, NaOEt, EtOH, 60 °C, 2 h; (b) N_2_H_4_·H_2_O, EtOH, reflux, 2 h, 54% over two steps; (c) NaOH, 1,4-dioxane/H_2_O 1:1 (v/v), 50 °C, 3 h, 62%; (d) conc. H_2_SO_4_, 65% HNO_3_, 60 °C, 3 h, 64%; (e) *cat*. DMF, (COCl)_2_, DCM, 0 °C, 1 h, then RT, 2 h; (f) 7 M NH_3_ in MeOH, 0 °C, 30 min, 69% over two steps; (g) 10% Pd/C, H_2_ (g), EtOH, 60 °C, 18 h, 93%; (h) RCOOH, TEA, bromo-tris-pyrrolidino-phosphonium hexafluorophosphate (PyBroP), DCE, microwave irradiation (MW) 120 °C, 20 min; (i) KO^*t*^Bu, ^*i*^PrOH, MW 130 °C, 30 min, 4–90% over two steps; (j) NaN_3_, NH_4_Cl, DMF, MW 160 °C, 2 h, 69%; (k) RCHO, I_2_, DMF, 80 °C, 16 h, 23–47%; (l) LiOH, 1,4-dioxane/H_2_O 1:1 (v/v), reflux, 2 h, 75%.

The designed pyrazolopyrimidinone analogs were synthesized via the route shown in Scheme 1. The synthesis of **1**, **4**–**8**, **11**, **12**, and **15** was reported previously.^27−30^ This route starts with condensation and ring closure reactions to form the pyrazole ester intermediate **4**. These two steps can be combined into a one-pot reaction. Following hydrolysis and nitration, intermediate **6** was obtained. The nitration reaction is a key step, and the rate of adding the reagent and the reaction temperature need to be carefully controlled.^31^ The first four (**a**–**d**) steps can be performed at a four hundred-gram scale without column purification. Following primary amide formation and reduction of the nitro group, the key intermediate **8** was formed. The final compounds can be obtained with two different methods from **8**. Analogs **9**–**37** and **40**–**42** were prepared by amide coupling followed by ring closure reactions under basic conditions (Scheme 1A), whereas analogs **38** and **43**–**45** were obtained by ring closure reactions with the corresponding aldehydes and iodine (Scheme 1B) due to starting material availability and reactivity. Analog **39** was obtained following hydrolysis of **38**, and [2 + 3] cycloaddition of **37** with NaN_3_ yielded tetrazole **46** with a decent yield. Due to tautomerism of the pyrazole ring in the structures, some carbon signals are too broad or invisible in the 1D NMR spectra, and earlier publications^27,29^ did not report complete chemical characterizations (especially ^13^C NMR signals) for the published analogs.^25,27^ Here, we report the results of ^13^C NMR combined with 2D NMR (HSQC and HMBC) or high-temperature NMR needed to obtain full characterization. For analog **30**, additional efforts with salt formation to prevent tautomerism ended up with sharp ^13^C NMR signals; the further “1,*n*-ADEQUATE” experiment confirmed its structure (Figures S90–94).

### In Vitro Evaluation of Anti-*T. brucei* Activity

In our PDE4NPD program, we initially synthesized a small library of 5-benzyl-3-isopropyl-1*H*-pyrazolo[4,3-*d*]pyrimidin-7(6*H*)-one (BIPPO, **1**, Figure 1) analogs as this scaffold was reported to inhibit PDEs from *P. falciparum*.^27^ Consequently, the pyrazolopyrimidinone scaffold was considered an interesting start to identifying antitrypanosomal compounds. The first small series focused on variations in the 5-position of the BIPPO scaffold (Table 1). The benzyl moiety was replaced with a number of aromatic and aliphatic substituents, and the linker length and flexibility between the pyrazolopyrimidinone moiety and the terminal aromatic substituent were varied to explore structure–activity relationships (SARs). The analogs were tested for activity against *T. b. brucei*, *T. cruzi*, *L. infantum,* and toxicity against the human MRC-5 cell line was determined to measure general toxicity and selectivity. Based on the results shown in Table S2, only *T. b. brucei* inhibition was observed; thereafter, we focused on modifications for improved antitrypanosomal activity.

As shown in Table 1, BIPPO (**1**) is only weakly active against *T. b. brucei*. A pyridine instead of a phenyl was introduced in **9** to increase solubility, but no potency improvement was observed. The same results were obtained with **10** and **11**, in which, respectively, an oxygen atom or a methylene group was introduced into the linker. Pyrazolopyrimidinones with aliphatic substituents (**12**, **13**, and **14**) at R^1^ also exhibited low potency. The potency of analogs with different aromatic substituents varied considerably. When the aromatic rings were directly attached to the pyrazolopyrimidinone moiety, a drastic improvement in antiparasitic activity was observed. Analogs **15** and **16** were 125 and 16 times more potent than **1**, respectively. However, **17** with a thiazole exhibited comparable activity to **1**.

The phenyl-analog **15** had a pIC_50_ of 6.6 against *T. b. brucei* without noticeable toxicity against MRC-5 cells and was the most active compound in our first round of modifications.

Our second round of modifications (Table 2) logically were based on compound **15**. Analogs with different substituents at the *ortho*, *meta*, and *para* positions were synthesized, and activities against *T. b. brucei* (Figure S2) and MRC-5 fibroblasts were determined. Analogs with an *ortho* substituent (**18**–**21**) showed comparable activity to **15**, with the *ortho*-substituted methyl analog **21** being the most potent (pIC_50_ 6.8). The analogs with an *ortho*-halogen substituent (**18**–**20**) were slightly less potent than **15**. The *ortho*-methoxy-substitued **22** was clearly less effective, with a 25-fold reduction in potency. The *meta-*halogen analogs **23** and **24** proved to be significantly more potent than their *ortho*-analogs **18** and **19**, with the *meta*-fluoro analog **23** showing a pIC_50_ of 7.0. Analog **25** with a *meta*-methyl group was equipotent to **15**, while **26** with a *meta*-methoxy group was slightly less potent. In contrast, the introduction of *meta*-OH, −N(CH_3_)_2_, and −SO_2_CH_3_ (**27**–**29**) led to a more than 10-fold reduction in potency compared with **15**.

**Table 2:**
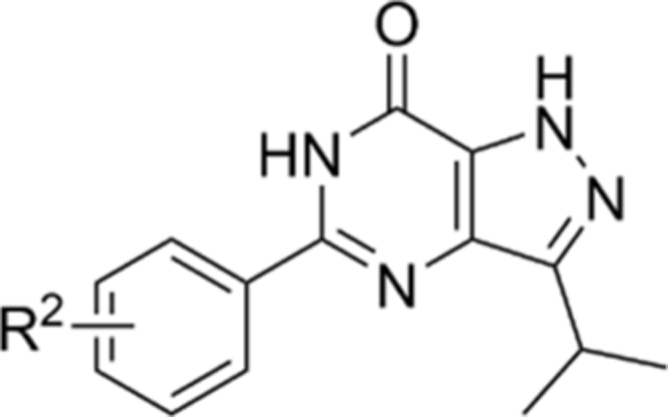
Exploration of SAR on the Phenyl Ring of **15**

code	R^2^	MW^a^	cLogP^a^	PSA^a^	*T. b. brucei* pIC_50_^b^	MRC-5 pIC_50_^b^
**15** (NPD-3200)	H	254.3	2.3	70.1	6.6 ± 0.2	<4.2
**18** (NPD-3199)	2-F	272.3	2.3	70.1	6.0 ± 0.1	<4.2
**19** (NPD-3538)	2-Cl	288.7	2.9	70.1	6.1 ± 0.2	<4.2
**20** (NPD-3539)	2-Br	333.2	2.9	70.1	6.2 ± 0.1	<4.2
**21** (NPD-3589)	2-Me	268.3	2.7	70.1	6.8 ± 0.2	<4.2
**22** (NPD-3590)	2-OMe	284.3	2.1	79.4	5.1 ± 0.1	<4.2
**23** (NPD-3202)	3-F	272.3	2.5	70.1	7.0 ± 0.1	<4.2
**24** (NPD-3591)	3-Cl	288.7	3.1	70.1	6.7 ± 0.3	<4.2
**25** (NPD-3382)	3-Me	268.3	2.8	70.1	6.6 ± 0.2	<4.2
**26** (NPD-3375)	3-OMe	284.3	2.4	79.4	6.3 ± 0.1	<4.2
**27** (NPD-2974)	3-OH	270.3	1.9	90.4	5.2 ± 0.0	<4.2
**28** (NPD-3381)	3-N(CH_3_)_2_	297.4	2.5	73.4	5.1 ± 0.0	<4.2
**29** (NPD-3598)	3-SO_2_CH_3_	332.4	1.2	104.3	4.5 ± 0.0	<4.2
**30** (NPD-2975)	4-F	272.3	2.5	70.1	7.2 ± 0.2	<4.2
**31** (NPD-3204)	4-Cl	288.7	3.1	70.1	7.0 ± 0.2	<4.2
**32** (NPD-2971)	4-Br	333.2	3.2	70.1	6.6 ± 0.3	<4.2
**33** (NPD-2972)	4-OMe	284.3	2.4	79.4	6.1 ± 0.1	<4.2

a cLogP and PSA (polar surface area) are calculated using CDD.

b Mean values of at least two independent experiments.

For analogs with *para*-substituents, **30** with a *para*-F substituent was the most potent in this series with a pIC_50_ of 7.2 (Table 2). A comparable potency was observed for the *para*-Cl analog **31**, but a four-fold reduction in potency was observed for the bromide **32**. The *para*-methoxy group in **33** decreased the pIC_50_ to 6.1, which is three times less active than **15**.

Based on the SAR results from Table 2, our third round of modifications (Table 3) focused on the *para*-position of the phenyl ring in **15** to further improve chemical diversity and physicochemical properties, such as polarity and solubility. Analogs with relatively small substituents **35**–**37** exhibited comparable potency with **15**. Once bigger or polar substituents (**38**–**46**) were introduced, pIC_50_ values decreased dramatically (<5.0), which indicates that only a restricted set of substituents is accepted at the *para*-position of this phenyl ring.

**Table 3:**
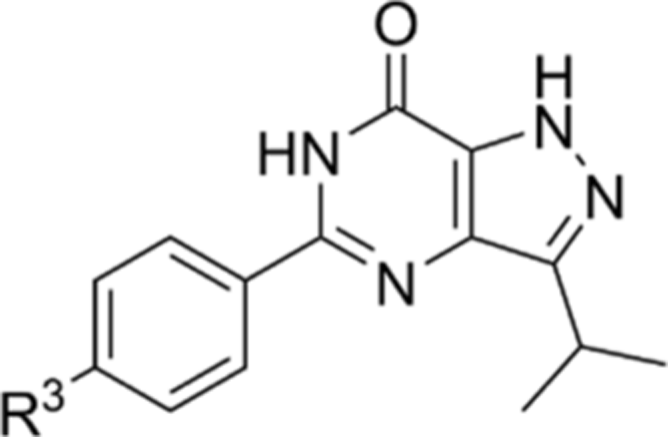
Exploration of SARs on the *Para*-position of the Phenyl Ring

code	R^3^	MW^a^	cLogP^a^	PSA^a^	*T. b. brucei* pIC_50_^b^	MRC-5 pIC_50_^b^
**34** (NPD-3377)	-O^*i*^Pr	312.4	3.2	79.4	<4.2	<4.2
**35** (NPD-3201)	-CF_3_	322.3	3.4	70.1	5.9 ± 0.1	<4.2
**36** (NPD-3597)	-OCF_3_	338.3	3.8	79.4	5.9 ± 0.2	<4.2
**37** (NPD-3203)	-CN	279.3	2.1	93.9	6.6 ± 0.3	<4.2
**38** (NPD-3305)	-COOMe	312.3	2.4	96.4	<4.2	<4.2
**39** (NPD-3489)	-COOH	298.3	2.1	107.4	<4.2	<4.2
**40** (NPD-3371)	-CONH_2_	297.3	1.3	113.2	<4.2	<4.2
**41** (NPD-3376)	-SO_2_CH_3_	332.4	1.2	104.3	4.3 ± 0.1	<4.2
**42** (NPD-3372)	-SO_2_NH_2_	297.3	1.0	113.2	4.4 ± 0.1	<4.2
**43** (NPD-3280)	-NHCOCH_3_	311.3	1.7	99.2	<4.2	<4.2
**44** (NPD-3283)	-piperidine	337.4	3.6	73.4	<4.2	<4.2
**45** (NPD-3282)	-methylpiperazine	352.4	2.1	76.6	4.7 ± 0.4	4.4 ± 0.1
**46** (NPD-3490)	-tetrazole	322.3	2.0	124.6	<4.2	<4.2

a cLogP and PSA (polar surface area) are calculated using CDD.

b Mean values of at least 2 independent experiments.

As **30** (NPD-2975) turned out to be the most interesting compound in this series in terms of in vitro potency and physicochemical properties (cLogP), this compound was selected for further antiparasitic profiling. Table 4 shows the activity of **30** against a panel of protozoan parasites, revealing nanomolar potency against *T. b. brucei* and no activity against *T. cruzi*, *L. infantum*, or *P. falciparum*. Moreover, no toxicity was observed against the human MRC-5 cell line or peritoneal mouse macrophages (PMM), endorsing its high selectivity.

**Table 4:** Potency of **30** against Other parasites

code	*T. b. brucei* pIC_50_	*T. cruzi* pIC_50_	*L. infantum* pIC_50_	*P. falciparum* pIC_50_	MRC-5 pIC_50_	PMM pIC_50_
**30** (NPD-2975)	7.2	<4.2	<4.2	<4.2	<4.2	<4.2

### In Vitro Profiling of **30**

Given its good potency and lack of toxicity, **30** was evaluated in detail in several in vitro assays. The target-based approach in the PDE4NPD consortium had shown that targeting both *Tbr*PDEB1 and *Tbr*PDEB2 with specific inhibitors^25^ will kill trypanosomes, thereby confirming earlier target validation work by Seebeck et al.^19,32^ As the scaffold of **30** was earlier identified to target PDE enzymes,^27^**30** was tested against *Tbr*PDEB1 but proved to be inactive (Table 5, Figure S3). Since both *Tbr*PDEB1 and *Tbr*PDEB2 need to be inhibited to kill trypanosomes,^19^ this observation immediately dismisses the PDE-hypothesis for the observed antiparasitic activity of **30**.

**Table 5:** Detailed In Vitro Profiling of **30** (NPD-2975)

assays	**30**
*Tbr*PDEB1 inhibition	p*K*_i_ < 5.0
CYP450 IC_50_	1A2: 0.16 μM
	2C9: >10 μM
	2C19: 0.42 μM
	2D6: >10 μM
	3A4: >10 μM
*h*ERG	>10 μM
mitochondrial toxicity	<10% toxicity at 10 μM
mini-ames	negative

To identify potential safety issues, **30** was subsequently tested at 10 μM in the *Eurofins Safety-47* panel, which includes 24 GPCRs, two nuclear hormone receptors, three transporters, eight ion channels, six non-kinase enzymes, and four kinase enzymes (Table S3). The activity of these targets was modulated <50% at 10 μM, with the exception of human PDE4D2, which was inhibited by 75% at 10 μM. Since compound **30** also shows some structural similarities with sildenafil, **30** was also tested against PDE5. At 10 μM **30** inhibited PDE5 by only 52%. Screening of **30** as inhibitors of a panel of CYP enzymes resulted in IC_50_ values of 0.16 and 0.42 μM against CYP1A2 and CYP2C19, respectively. Inhibition of CYP2C9, CYP2D6, and CYP3A4 by **30** was not observed in the tested concentration range. Finally, negative results from a mini-Ames test, *h*ERG affinity, and a mitochondrial toxicity test further supported the drug-like quality of **30** (Table 5).

### Metabolic Stability

**Figure 2:**
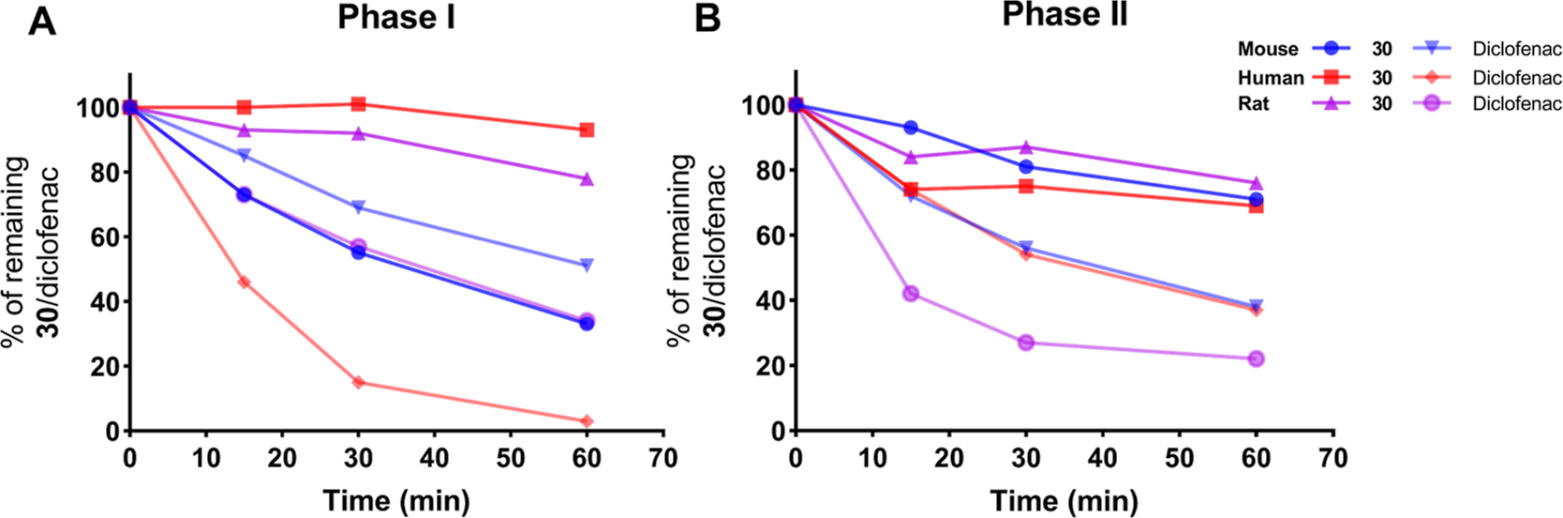
In vitro metabolic stability of **30** in liver microsomes. (A) Simulated phase-I metabolic stability of **30** in mouse, rat, and human liver microsomes with diclofenac as the reference. (B) Simulated phase-II metabolic stability of **30** in mouse, rat, and human liver microsomes with diclofenac as reference. Source data are provided in Table S4.

Metabolic stability was assessed by incubating **30** and the control diclofenac with mouse, rat, and human liver microsomes in the absence or presence of uridine diphosphate glucuronic acid (UDPGA) to stimulate phase-II metabolism. The results indicated that **30** was metabolized the slowest by human microsomes and to a moderate extent in rodent microsomes (Figure 2). Phase-I metabolism in mouse microsomes resulted in 55% of the parent drug remaining after 30 min of incubation, which can be defined as acceptable metabolic stability.^33^ No significant difference was observed when metabolism by UGT enzymes was induced by the addition of UDPGA (phase-II metabolism), suggesting that phase-I metabolism is the main route of metabolism in **30**.

Based on its low cytotoxicity, potent in vitro activity (Table 2), good selectivity (Table 4), and acceptable metabolic stability in mouse microsomes (Figure 2), **30** was progressed to in vivo evaluation in mouse.

### In Vivo Pharmacokinetics

**Figure 3:**
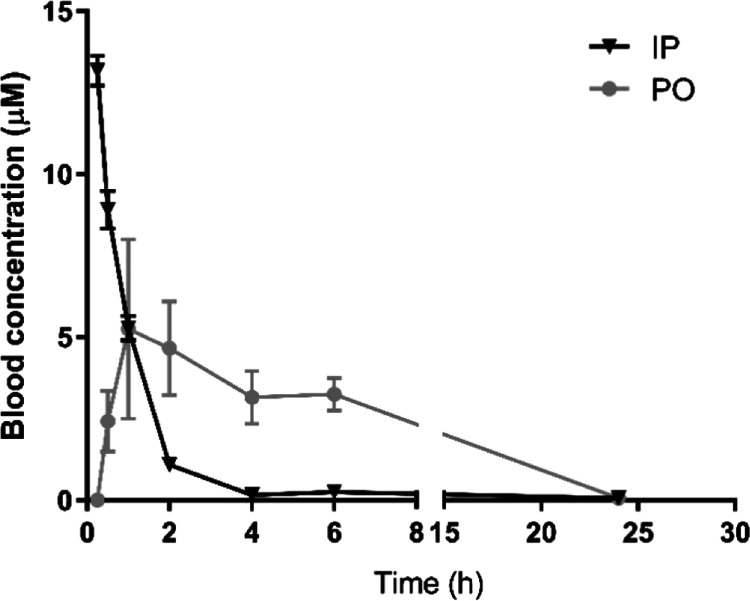
Blood levels (0–24 h) of **30** in mice after a single IP dose (10 mg/kg) or PO dose (50 mg/kg). Results are expressed in mean blood concentration (μM) ± standard error of the mean.

The pharmacokinetic profiles of **30** were determined after either oral (PO) or intraperitoneal (IP) administration (Figure 3), and their blood concentrations were used to derive various pharmacokinetic parameters (Table 6). Both PO and IP administration quickly led to micromolar blood concentrations that exceeded the IC_50_ more than 50-fold (Table 6, Figure 3). For the subsequent evaluation of **30** in a mouse model of acute *T. b. brucei* infection, 50 mg/kg PO administration was used.

**Table 6:** Pharmacokinetic Parameters of **30**

compound	dosing	*T*_max_ (h)	*C*_max_ (μM)	*T*_1/2_ (h)	AUC_0–6h_ (ng·h/mL)	Cl (mL/min)
**30**	50 mg/kg PO	1	5.25	3.46	6064.75	58.5
	10 mg/kg IP		13.18	1.06	3928.37	171

### In Vivo Evaluation of **30**

**Figure 4:**
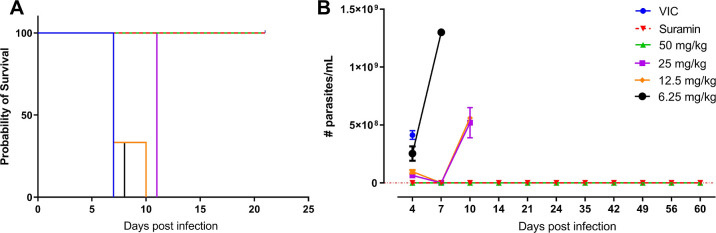
In vivo evaluation of **30** in a stage-I mouse model of HAT. Survival rate (A) and parasitemia (B) of stage-I *T. b. brucei*-infected mice treated with vehicle (*n* = 3), suramin (*n* = 3) at 10 mg/kg or **30** (*n* = 3) at 50, 25, 12.5, and 6.25 mg/kg.

Next to suramin (10 mg/kg IP s.i.d. for 5 days) as a positive reference in a mouse model of acute *T. b. brucei* infection, treatment with **30** at 50 mg/kg twice daily PO for 5 consecutive days resulted in apparent full clearance of parasitemia (Figure 4). In contrast to the high parasitemia and early mortality in the vehicle-treated mice, all **30**-treated animals in the highest dosing group were devoid of peripheral blood parasites throughout the 60 days post-infection (dpi) follow-up period. An additional SL RNA qPCR confirmed the absence or undetectable levels of parasites in peripheral blood. A clear dose-dependent in vivo efficacy was recorded, as all animals treated at 25 mg/kg b.i.d. for 5 days relapsed and succumbed within 11 dpi.

## Discussion

We present a series of 5-phenylpyrazolopyrimidinone analogs with low nanomolar IC_50_ values and promising in vivo efficacy against *T. b. brucei*. Based on the published anti-*P. falciparum* inhibitor **1** (BIPPO), a library of 38 analogs was designed and phenotypically screened against *T. b. brucei*. The shortening of the linker between the phenyl group and the pyrazolopyrimidinone moiety drastically increased its potency, resulting in **15** with a pIC_50_ of 6.6. Further modification based on the structure of **15** with various substituents on its phenyl ring led to the discovery of **30** with an IC_50_ of 70 nM against *T. b. brucei* and no noticeable toxicity for a number of other protozoan parasites or human cell lines. Additional modifications based on the structure of **30** to improve solubility did not yield analogs with improved activity. Potentially, this might be a result of limited space or a lipophilic environment in the binding pocket of its target, which is currently unknown.

Follow-up analysis revealed that **30** did not inhibit the *Tbr*PDEB1 enzyme, a validated target for HAT, and showed good selectivity over a range of targets (24 GPCRs, 2 nuclear hormone receptors, 3 transporters, 8 ion channels, 6 non-kinase enzymes, and 4 kinase enzymes). Also, **30** tested negative in a mini-Ames test and showed no *h*ERG liability, two crucial criteria in drug discovery for a ‘lead’ compound. With respect to metabolism, **30** did not inhibit CYP2C9, CYP2D6, or CYP3A4 but did have moderate CYP1A2 and 2C19 liability and was metabolically stable in human and rodent liver microsomes, resulting in micromolar levels of **30** in mouse serum after PO and IP administration. In an acute mouse model of HAT, **30** yielded a 100% survival rate at 50 mg/kg b.i.d. for 5 days, making it an interesting lead for HAT treatment. Unfortunately, its mode of action is still unknown and is currently being investigated with a metabolomics approach^34,35^ and an RNAi method, as previously reported.^36^

Although the number of HAT patients is decreasing every year, many people living in sub-Saharan Africa are still at risk of infection. In the past few years, a number of publications^25,33,37−44^ indeed specifically focused on antitrypanosomal drug discovery. Compared to these published scaffolds, the pyrazolopyrimidinones (including **30**) have a relatively low molecular weight, which is for most of the analogs lower than 300 Dalton. This is a very nice feature of this compound series and can be further exploited during lead optimization. Next to that, other physicochemical properties (such as cLogP, number of hydrogen bond donors/acceptors) of **30** fit with Lipinski’s rule of five,^45^ indicating good drug-like properties. Also, the selectivity and metabolic stability of **30** are remarkable. Last but not least, its in vivo pharmacokinetic features and in vivo efficacy are outstanding since a complete cure was obtained at 50 mg/kg b.i.d. PO for 5 days without relapse at 60 dpi.

## Conclusions

To conclude, our pyrazolopyrimidinone analogs with phenyl substituents are novel, potent, and selective antitrypanosomal agents. Compound **30** with a *para*-fluorophenyl group exhibits an IC_50_ of 70 nM against *T. b. brucei*. Follow-up physiochemical feature analysis, metabolic stability, and pharmacokinetic testing revealed its excellent drug-like properties. Importantly, the absence of detectable parasite levels in peripheral blood following an oral dose of 50 mg/kg b.i.d. for 5 days in mice disclosed its promising in vivo potential and deserves further exploration for future drug development.

## Experimental Section

### In Vitro Evaluation

All compounds tested (**1**, **9**–**46**) pass a publicly available pan-assay interference compound filter.^46,47^ The antiparasitic assays and the *Tbr*PDEB1 enzyme assay were carried out as described in Blaazer et al.^25^ Briefly, phosphodiesterase activity assays were performed using the PDELight HTS cAMP phosphodiesterase Kit (Lonza, Walkersville, USA) at 25 °C in non-binding, low volume 384 wells plates (Corning, Kennebunk, ME, USA). PDE activity was measured in “stimulation buffer” (50 mM Hepes, 100 mM NaCl, 10 mM MgCl_2_, 0.5 mM EDTA, 0.05 mg/mL BSA, pH 7.5). Dose response curves were measured in triplo. The compounds were diluted in DMSO. Inhibitor dilutions (2.5 μL) were transferred to the 384 wells plate, 2.5 μL PDE in stimulation buffer was added and mixed, 5 μL cAMP (at 2 × km up to 20 μM) was added, and the assay was incubated for 20 min at 300 rpm. The reaction was terminated with 5 μL Lonza Stop Buffer supplemented with 10 μM NPD-0001. Luminescence was determined in a Victor3 luminometer. Antitrypanosomal assays were carried out with the *T. brucei* Squib 427 strain cultured in Hirumi’s modified Iscove’s medium 9 (HMI-9) supplemented with 10% fetal bovine serum at 37 °C in a 5% CO_2_ atmosphere. Parasites were seeded at a concentration of 1.5 × 10^4^ parasites/well. Four-fold dilutions of the test compounds were added, with the highest in-test concentration of 64 μM. After 72 h of drug exposure, viability was determined by incubation with 10 μg/mL resazurin (Sigma-Aldrich, St. Louis, MO, USA) and fluorescence reading after 24 h. The percentage growth inhibition compared to untreated control wells was used to calculate the 50% inhibitory concentration (IC_50_). The CYP450 assays, *h*ERG cardiotoxicity assay, mitochondrial toxicity assay, and data analysis were carried out as described in Moraes et al.^48^ The mini-Ames test (Wuxi^49^) and the Eurofins Safety47 panel (Eurofins^50^) screens were outsourced to CROs.

### Metabolic Stability

The microsomal stability assay was carried out based on the BD Biosciences Guidelines for Use (TF000017 Rev1.0) with minor adaptations. Male mouse and pooled human liver microsomes (Corning) were purchased and stored at −80 °C until use. Both CYP450 and other NADPH-dependent enzymes (phase-I metabolism) and UGT enzymes (phase-II metabolism) were evaluated for NPD-2975 (**30**) with a working concentration of 5 μM. Diclofenac was used as a reference drug. Compound **30** was incubated with 0.5 mg/mL liver microsomes in potassium phosphate buffer, and reactions were initiated by the addition of 1 mM NADPH and 2 mM UDPGA cofactors for phase-I and phase-II metabolism, respectively. Samples were collected after 0, 15, 30, and 60 min. At these time points, 20 μL was withdrawn from the reaction mixture, and 80 μL cold acetonitrile (MeCN), containing the internal standard tolbutamide, was added to inactivate the enzymes and precipitate the proteins. The mixture was vortexed for 30 s and centrifuged at 4 °C for 5 min at 15,000 rpm. The supernatant was stored at −80 °C until analysis. The loss of parent compound was determined using liquid chromatography (UPLC) (Waters Aquity) coupled with tandem quadrupole mass spectrometry (MS^2^) (Waters Xevo), equipped with an electrospray ionization (ESI) interface, and operated in the multiple reaction monitoring (MRM) mode.

### Pharmacokinetics

Analog **30** (NPD-2975) was evaluated for its pharmacokinetic properties after a single dose, either IP at 10 mg/kg or PO at 50 mg/kg. Blood drops from the animals were sampled before treatment and at 0.5, 1, 2, 4, 6, and 24 h after PO dosage; sampling after IP dosage was identical with an additional time point of 0.25 h. The blood drops were analyzed adopting the dry blood spot collection and analysis by LC–MS^2^. Briefly, blood samples were collected from the tail vein using capillary tubes and dropped (15 μL) on Whatman FTA DMPK cards (B). The spots were left to air dry at room temperature for at least 2 h. For analysis, a 6 mm disk was punched out and extracted in 75:25% MeCN/water containing the internal standard, tolbutamide. The brain tissue of the animals was collected on ice at autopsy 24 h post-treatment after perfusion. The tissue was immediately homogenized using a GentleMacs tissue homogenizer. The homogenates were then either immediately processed for analysis or stored at −80 °C. The tissue samples were subjected to protein precipitation by adding MeCN, followed by a centrifugation step at 4 °C for 5 min at 15,000 rpm. The supernatant was further diluted in 75:25% MeCN/water. The bio-analysis used liquid chromatography (UPLC) (Waters Aquity) coupled with tandem quadrupole mass spectrometry (MS^2^) (Waters Xevo), equipped with an ESI interface, and operated in the MRM mode. Standard curves in whole blood were made for calibration and validation. Standard pharmacokinetic parameters were determined using Topfit software.

### Acute Mouse Model

Mice were allocated to groups of three and were infected by an IP injection with 10^4^ trypomastigotes of *T. b. brucei* (suramin-sensitive Squib 427 strain). Compound **30** (NPD-2975) was formulated in PEG_400_ at 12.5 and 6.25 mg/mL, envisaging a maximal dosing volume of 100 μL/25 g live body weight. Next to including a vehicle control group with only PEG_400_, suramin was included as the reference drug and was injected IP s.i.d. for 5 consecutive days at 10 mg/kg. **30** was administered PO b.i.d. for 5 consecutive days at 25 or 50 mg/kg. The first treatment was given 30 min prior to the artificial infection. Drug efficacy was evaluated by microscopic determination of the parasitemia in tail vein blood samples at several time points until 63 dpi. An additional SL RNA qPCR assay was performed for all surviving animals to confirm parasitological cure. Animals were observed for the occurrence/presence of clinical or adverse effects during the course of the experiment. In cases of very severe clinical signs, either due to toxicity or clinical disease, animals were euthanized for animal welfare reasons. All animal experiments were conducted in compliance with institutional guidelines and following approval by the Ethical Committee of the University of Antwerp, Belgium [UA-ECD 2014–96].

### Chemistry

#### General Information

All starting materials were obtained from commercial suppliers and used without purification. Anhydrous THF, DCM, and DMF were obtained by passing through an activated alumina column prior to use. All reactions were carried out under a nitrogen atmosphere, unless mentioned otherwise. TLC analyses were performed using Merck F_254_ aluminum-backed silica plates and visualized with 254 nm UV light. Flash column chromatography was executed using Biotage Isolera equipment. All HRMS spectra were recorded on a Bruker microTOF mass spectrometer using ESI in positive-ion mode. All NMR spectra were recorded on either a Bruker Avance 300, 400, 500, or 600 spectrometer at 25 °C, unless mentioned otherwise. The peak multiplicities are defined as follows: s, singlet; d, doublet; t, triplet; q, quartet; p, pentet; dd, doublet of doublets; dt, doublet of triplets; td, triplet of doublets; br, broad; m, multiplet, app, and apparent. The spectra were referenced to the internal solvent peak as follows: CDCl_3_ (δ = 7.26 ppm in ^1^H NMR, δ = 77.16 ppm in ^13^C NMR), DMSO-*d*_6_ (δ = 2.50 ppm in ^1^H NMR, δ = 39.52 ppm in ^13^C NMR). IUPAC names were adapted from ChemBioDraw Ultra 19.0. Purities were measured with the aid of analytical LC–MS using a Shimadzu LC-20AD liquid chromatography pump system with a Shimadzu SPDM20A diode array detector, with MS detection performed with a Shimadzu LCMS-2010EV mass spectrometer operating in the positive (or negative) ionization mode. The column used was an Xbridge (C18) 5 μm column (100 mm × 4.6 mm). The following solutions are used for the eluents. Solvent A: H_2_O/HCOOH 999:1, and solvent B: MeCN/HCOOH 999:1. The eluent program used is as follows: flow rate: 1.0 mL/min, start with 95% A in a linear gradient to 10% A over 4.5 min, hold 1.5 min at 10% A, in 0.5 min in a linear gradient to 95% A, hold 1.5 min at 95% A, and total run time: 8.0 min. Compound purities were calculated as the percentage peak area of the analyzed compound by UV detection at 254 nm. All final compounds (**1**, **9**-**46**) are >95% pure by HPLC analysis (Figures S4–S142). Note: not all ^13^C signals are visible in the spectrum due to the rapid tautomerism of non-N-substituted pyrazoles. HSQC and HMBC were measured to assign ^13^C signals if needed.

#### Ethyl 3-Isopropyl-1*H*-pyrazole-5-carboxylate (**4**)

Diethyl oxalate **2** (20.0 mL, 147 mmol) and 3-methylbutan-2-one (15.8 mL, 150 mmol) were added to a mixture of NaOEt (14.0 g, 206 mmol) in EtOH (300 mL) at RT over 1 h. The reaction mixture was heated at 60 °C for 2 h, after which AcOH (47.7 mL, 294 mmol) and 64–65% N_2_H_4_ aqueous solution (9.10 g, 160 mmol) were added, and the mixture was stirred under reflux for 2 h. Water (200 mL) was added after the reaction mixture was evaporated under reduced pressure, and the mixture was extracted with EtOAc (3 × 150 mL). The combined organic layers were washed with brine, dried over MgSO_4_, and concentrated in vacuo. The residue was subjected to silica gel column chromatography and eluted with EtOAc/cyclohexane 1:1 to give the title product **4** as a white solid (14.5 g, 54% for two steps). ^1^H NMR (300 MHz, CDCl_3_): δ 7.75 (br s, 1H), 6.63 (s, 1H), 4.37 (q, *J* = 7.1 Hz, 2H), 3.05 (hept, *J* = 7.7 Hz, 1H), 1.37 (t, *J* = 7.0 Hz, 3H), 1.30 (d, *J* = 6.8 Hz, 6H). ^13^C NMR (151 MHz, CDCl_3_): δ 161.5, 155.0, 140.6, 105.0, 61.2, 26.6, 22.5, 14.4. LC–MS: *t*_R_ = 3.61 min, purity: >99%, not ionized. Spectral data agree with a previous report.^29^

#### 3-Isopropyl-1*H*-pyrazole-5-carboxylic Acid (**5**)

Ester **4** (27.0 g, 148 mmol) and NaOH (14.8 g, 370 mmol) were dissolved in 1,4-dioxane (400 mL) and water (400 mL) and heated to 50 °C for 3 h. The reaction mixture was concentrated under reduced pressure and washed with EtOAc (3 × 200 mL); the pH was adjusted to 1 with the concentrated aqueous HCl solution, and the title product **5** was filtered as an off-white solid (14.2 g, 62%). ^1^H NMR (300 MHz, DMSO-*d*_6_): δ 6.47 (s, 1H), 2.94 (hept, *J* = 7.1 Hz, 1H), 1.21 (d, *J* = 6.9 Hz, 6H). ^13^C NMR (151 MHz, DMSO-*d*_6_): δ 163.3, 153.1, 141.6, 104.6, 26.1, 22.8. LC–MS: *t*_R_ = 2.72 min, purity: >99%, *m*/*z* [M – H]^−^: 153. Spectral data agree with a previous report.^29^

#### 3-Isopropyl-4-nitro-1*H*-pyrazole-5-carboxylic Acid (**6**)

Carboxylic acid **5** (14.2 g, 92.0 mmol) was added portion-wise to concentrated H_2_SO_4_ (74 mL, 1.36 mol) at RT with stirring. The reaction mixture was heated to 60 °C, and HNO_3_ (65%, 17.8 mL, 279 mmol) was added dropwise. The reaction was then stirred at 60 °C for 3 h, cooled to RT, and poured onto 200 g of ice with stirring. After 15 min, the white precipitate was isolated by filtration, washed with water (300 mL), and dried under reduced pressure to give the title product **6** as a white solid (11.7 g, 64%). ^1^H NMR (600 MHz, DMSO-*d*_6_): δ 3.49 (hept, *J* = 7.1, Hz, 1H), 1.29 (d, *J* = 7.0 Hz, 6H). ^13^C NMR (151 MHz, DMSO-*d*_6_): δ 162.8, 149.8, 129.4, 25.5, 21.2. LC–MS: *t*_R_ = 2.70 min, purity: >99%, *m*/*z* [M – H]^−^: 198. Spectral data agree with a previous report.^29^

#### 3-Isopropyl-4-nitro-1*H*-pyrazole-5-carboxamide (**7**)

Oxalyl chloride (16.6 mL, 189 mmol) was added dropwise to a suspension of carboxylic acid **6** (12.6 g, 63.1 mmol) in DCM (240 mL) containing DMF (300 μL, 3.87 mmol) at 0 °C. The reaction mixture was stirred at 0 °C for 1 h, allowed to warm to RT, and stirred for another 2 h. The reaction mixture was added dropwise to a solution of 7 M NH_3_ in MeOH (54.1 mL, 379 mmol) at 0 °C, stirred for 0.5 h. The reaction mixture was concentrated in vacuo and purified by flash column chromatography on silica gel with a gradient elution of MeOH in DCM (0–10%) to yield the title compound **7** as a white solid (8.65 g, 69%). ^1^H NMR (300 MHz, DMSO-*d*_6_): δ 13.84 (br s, 1H), 7.99 (s, 1H), 7.71 (s, 1H), 3.53 (hept, *J* = 7.2 Hz, 1H), 1.28 (d, *J* = 6.9 Hz, 6H). ^13^C NMR (151 MHz, DMSO-*d*_6_): δ 162.2, 149.4, 143.5, 128.2, 25.2, 20.8. LC–MS: *t*_R_ = 2.61 min, purity: >99%, *m*/*z* [M + H]^+^: 199. Spectral data agree with a previous report.^29^

#### 4-Amino-3-isopropyl-1*H*-pyrazole-5-carboxamide (**8**)

Carboxamide **7** (5.70 g, 28.8 mmol) and 10% palladium on carbon (1.00 g, 0.940 mmol) in EtOH (90 mL) were stirred under H_2_ atmosphere at 60 °C for 18 h. The reaction mixture was filtered, and the solid was washed with MeOH (50 mL). The filtrate was concentrated in vacuo and purified by flash column chromatography on silica gel eluting with DCM/MeOH 9:1 to give the title product **8** as an off-white solid (4.50 g, 93%). ^1^H NMR (400 MHz, DMSO-*d*_6_, 373 K): δ 12.08 (br s, 1H), 6.81 (br s, 2H), 3.05–2.89 (m, 3H), 1.22 (d, *J* = 7.0 Hz, 6H). ^13^C NMR (151 MHz, DMSO-*d*_6_): δ 166.4, 133.4, 132.4, 128.0, 23.4, 21.4. LC–MS: *t*_R_ = 2.11 min, purity: 99%, *m*/*z* [M + H]^+^: 169. Spectral data agree with a previous report.^29^

#### General Procedure for the Synthesis of Analogs **1**, **9**–**46**

Method A: 4-Amino-3-isopropyl-1*H*-pyrazole-5-carboxamide **8** (1.0 equiv) and the corresponding acid (1.0 equiv), PyBrop (1.1 equiv), and TEA (2.0 equiv) were combined in DCE and heated using microwave irradiation at 120 °C for 20 min. The reaction mixture was purified by column chromatography with an eluent of DCM and MeOH to get amide intermediates as off-white solids. Then, the amide intermediate was combined with KO^*t*^Bu (2.0 equiv) in ^*i*^PrOH (10 mL) and heated using microwave irradiation at 130 °C for 30 min. The reaction mixture was concentrated in vacuo and purified by column chromatography to get the final products.

Method B: 4-Amino-3-isopropyl-1*H*-pyrazole-5-carboxamide **8** (1.0 equiv) and the corresponding aldehyde (1.0 equiv), I_2_ (2.0 equiv) were combined in DMF and heated at 80 °C 16 h. The reaction was quenched with a saturated Na_2_S_2_O_3_ aqueous solution and extracted with EtOAc. The combined organic layers were washed with water, concentrated in vacuo, and purified by column chromatography to obtain the final products.

#### 5-Benzyl-3-isopropyl-1,6-dihydro-7*H*-pyrazolo[4,3-*d*]pyrimidin-7-one **1** (NPD-0019)

Prepared from **8** (80 mg) via Method A as a white solid (87 mg, 68% for two steps). ^1^H NMR (500 MHz, DMSO-*d*_6_): δ 13.63 (br s, 1H), 12.18 (br s, 1H), 7.37–7.28 (m, 4H), 7.25–7.20 (m, 1H), 3.90 (s, 2H), 3.24 (hept, *J* = 6.8 Hz, 1H), 1.32 (d, *J* = 7.0 Hz, 6H). ^13^C NMR (151 MHz, DMSO-*d*_6_): δ 152.4 (HMBC), 150.3 (HMBC), 137.1, 128.7, 128.4, 126.6, 40.3, 25.8 (HSQC), 21.8. LC–MS: *t*_R_ = 3.66 min, purity: >99%, *m*/*z* [M + H]^+^: 269; HR-MS: calcd for C_15_H_16_N_4_O [M + H]^+^, 269.1397; found, 269.1385. Spectral data agree with a previous report.^27^

#### 3-Isopropyl-5-(pyridin-4-ylmethyl)-1,6-dihydro-7*H*-pyrazolo[4,3-*d*]pyrimidin-7-one **9** (NPD-2960)

Prepared from **8** (80 mg) via Method A as a white solid (75 mg, 59% for two steps). ^1^H NMR (500 MHz, DMSO-*d*_6_ + 1 drop of D_2_O): δ 8.48 (d, *J* = 5.3 Hz, 2H), 7.32 (d, *J* = 5.6 Hz, 2H), 3.95 (s, 2H), 3.22 (hept, *J* = 6.9 Hz, 1H), 1.29 (d, *J* = 7.0 Hz, 6H). ^13^C NMR (126 MHz, DMSO-*d*_6_ + 1 drop of D_2_O): δ 150.6 (HMBC), 149.6, 145.9, 124.2, 39.5, 26.1 (HSQC), 21.8. LC–MS: *t*_R_ = 2.26 min, purity: 98%, *m*/*z* [M + H]^+^: 270; HR-MS: calcd for C_14_H_15_N_5_O [M + H]^+^, 270.1349; found, 270.1341.

#### 5-(Benzyloxy)-3-isopropyl-1,6-dihydro-7*H*-pyrazolo[4,3-*d*]pyrimidin-7-one **10** (NPD-0434)

Prepared from **8** (197 mg) via Method A as a white solid (200 mg, 60% for two steps). ^1^H NMR (600 MHz, DMSO-*d*_6_): δ 13.73 (br s, 1H), 12.40 (br s, 1H), 7.33–7.28 (m, 2H), 7.08–7.03 (m, 2H), 6.99–6.95 (m, 1H), 4.95 (s, 2H), 3.26 (app s, 1H), 1.33 (d, *J* = 7.0 Hz, 6H). ^13^C NMR (151 MHz, DMSO-*d*_6_): δ 157.9, 150.8 (HMBC), 148.9 (HMBC), 142.3 (HMBC), 129.5, 121.2, 114.8, 67.8, 26.2 (HSQC), 21.8. LC–MS: *t*_R_ = 3.80 min, purity: >99%, *m*/*z* [M + H]^+^: 285; HR-MS: calcd for C_15_H_16_N_4_O_2_ [M + H]^+^, 285.1346; found, 285.1341.

#### 3-Isopropyl-5-phenethyl-1,6-dihydro-7*H*-pyrazolo[4,3-*d*]pyrimidin-7-one **11** (NPD-3281)

Prepared from **8** (80 mg) via Method A as a white solid (96 mg, 71% for two steps). ^1^H NMR (600 MHz, DMSO-*d*_6_): δ 13.52 (br s, 1H), 12.11 (br s, 1H), 7.29–7.25 (m, 4H), 7.20–7.16 (m, 1H), 3.26 (app s, 1H), 3.07–2.97 (m, 2H), 2.88 (app s, 2H), 1.34 (d, *J* = 6.9 Hz, 6H). ^13^C NMR (151 MHz, DMSO-*d*_6_): δ 154.3 (HMBC), 153.5 (HMBC), 150.8 (HMBC), 141.3, 137.5 (HMBC), 128.9, 128.7, 126.5, 36.2, 33.2, 26.6 (HSQC), 22.3. LC–MS: *t*_R_ = 3.87 min, purity: >99%, *m*/*z* [M + H]^+^: 283; HR-MS: calcd for C_16_H_18_N_4_O [M + H]^+^, 283.1553; found, 283.1545. Spectral data agree with a previous report.^27^

#### 3-Isopropyl-5-methyl-1,6-dihydro-7*H*-pyrazolo[4,3-*d*]pyrimidin-7-one **12** (NPD-3380)

Prepared from **8** (156 mg) via Method A as a white solid (0.16 g, 90% for two steps). ^1^H NMR (300 MHz, DMSO-*d*_6_): δ 13.52 (br s, 1H), 11.99 (br s, 1H), 3.23 (hept, *J* = 6.2 Hz, 1H), 2.31 (s, 3H), 1.32 (d, *J* = 7.0 Hz, 6H). ^13^C NMR (126 MHz, DMSO-*d*_6_): δ 151.1, 141.1 (HMBC), 26.4 (HSQC), 22.4, 21.5. LC–MS: *t*_R_ = 2.46 min, purity: >99%, *m*/*z* [M + H]^+^: 193; HR-MS: calcd for C_9_H_12_N_4_O [M + H]^+^, 193.1084; found, 193.1090. Spectral data agree with a previous report.^30^

#### 5-Butyl-3-isopropyl-1,6-dihydro-7*H*-pyrazolo[4,3-*d*]pyrimidin-7-one **13** (NPD-3645)

Prepared from **8** (72 mg) via Method A as a white solid (88 mg, 42% for two steps). ^1^H NMR (500 MHz, DMSO-*d*_6_): δ 13.52 (br s, 1H), 12.02 (br s, 1H), 3.29–3.17 (m, 1H), 2.61–2.53 (m, 2H), 1.65 (app p, *J* = 7.6 Hz, 2H), 1.37–1.28 (m, 8H), 0.89 (t, *J* = 7.4 Hz, 3H). ^13^C NMR (126 MHz, DMSO-*d*_6_): δ 154.5 (HMBC), 150.6 (HMBC), 141.4 (HMBC), 34.2, 29.7, 26.5, 22.3, 22.1, 14.2. LC–MS: *t*_R_ = 3.58 min, purity: >99%, *m*/*z* [M + H]^+^: 235; HR-MS: calcd for C_12_H_18_N_4_O [M + H]^+^, 235.1553; found, 235.1562.

#### 3,5-Diisopropyl-1,6-dihydro-7*H*-pyrazolo[4,3-*d*]pyrimidin-7-one **14** (NPD-3379)

Prepared from **8** (0.15 g) via Method A as a white solid (0.17 g, 87% for two steps). ^1^H NMR (300 MHz, DMSO-*d*_6_): δ 13.54 (br s, 1H), 11.88 (br s, 1H), 3.23 (hept, *J* = 6.8 Hz, 1H), 2.87 (hept, *J* = 7.8 Hz, 1H), 1.34 (d, *J* = 6.9 Hz, 6H), 1.22 (d, *J* = 6.8 Hz, 6H). ^13^C NMR (151 MHz, DMSO-*d*_6_): δ 158.1 (HMBC), 150.7 (HMBC), 32.8, 26.3 (HSQC), 21.8, 20.7. LC–MS: *t*_R_ = 3.42 min, purity: >99%, *m*/*z* [M + H]^+^: 221; HR-MS: calcd for C_11_H_16_N_4_O [M + H]^+^, 221.1397; found, 221.1405.

#### 3-Isopropyl-5-phenyl-1,6-dihydro-7*H*-pyrazolo[4,3-*d*]pyrimidin-7-one **15** (NPD-3200)

Prepared from **8** (0.15 g) via Method A as a white solid (73 mg, 32% for two steps). ^1^H NMR (500 MHz, DMSO-*d*_6_ + 1 drop of D_2_O): δ 8.04–7.98 (m, 2H), 7.55–7.48 (m, 3H), 3.33 (hept, *J* = 7.0 Hz, 1H), 1.37 (d, *J* = 7.0 Hz, 6H). ^13^C NMR (126 MHz, DMSO-*d*_6_ + 1 drop of D_2_O): δ 151.8 (HMBC), 150.4 (HMBC), 143.3 (HMBC), 133.6, 131.3, 129.3, 128.0, 26.6 (HSQC), 22.4. LC–MS: *t*_R_ = 3.78 min, purity: >99%, *m*/*z* [M + H]^+^: 255; HR-MS: calcd for C_14_H_14_N_4_O [M + Na]^+^, 277.1060; found, 277.1070. Spectral data agree with a previous report.^27^

#### 3-Isopropyl-5-(pyridin-4-yl)-1,6-dihydro-7*H*-pyrazolo[4,3-*d*]pyrimidin-7-one **16** (NPD-3488)

Prepared from **8** (86 mg) via Method A as a white solid (43 mg, 33% for two steps). ^1^H NMR (300 MHz, DMSO-*d*_6_ + 1 drop of D_2_O): δ 8.69 (d, *J* = 6.0 Hz, 2H), 7.96 (d, *J* = 6.0 Hz, 2H), 3.33 (app s, 1H), 1.36 (d, *J* = 6.9 Hz, 6H). ^13^C NMR (151 MHz, DMSO-*d*_6_ + 1 drop of D_2_O): δ 150.6, 148.1 (HMBC), 143.8 (HMBC), 140.7, 121.8, 26.8 (HSQC), 22.3. LC–MS: *t*_R_ = 2.63 min, purity: >99%, *m*/*z* [M + H]^+^: 256; HR-MS: calcd for C_13_H_13_N_5_O [M + H]^+^, 256.1193; found, 256.1186.

#### 3-Isopropyl-5-(thiazol-4-yl)-1,6-dihydro-7H-pyrazolo[4,3-*d*]pyrimidin-7-one **17** (NPD-2973)

Prepared from **8** (80 mg) via Method A as a white solid (67 mg, 54% for two steps). ^1^H NMR (500 MHz, DMSO-*d*_6_ + 1 drop of D_2_O): δ 9.26 (s, 1H), 8.50 (s, 1H), 3.33 (app s, 1H), 1.38 (d, *J* = 6.8 Hz, 6H). ^13^C NMR (126 MHz, DMSO-*d*_6_ + 1 drop of D_2_O): δ 155.4 (HSQC), 149.1, 149.0 (HMBC), 144.5, 135.8 (HMBC), 121.9, 25.8, 22.0. LC–MS: *t*_R_ = 3.40 min, purity: >99%, *m*/*z* [M + H]^+^: 262; HR-MS: calcd for C_11_H_11_N_5_OS [M + H]^+^, 262.0757; found, 262.0756.

#### 5-(2-Fluorophenyl)-3-isopropyl-1,6-dihydro-7*H*-pyrazolo[4,3-*d*]pyrimidin-7-one **18** (NPD-3199)

Prepared from **8** (80 mg) via Method A as a white solid (43 mg, 33% for two steps). ^1^H NMR (500 MHz, DMSO-*d*_6_): δ 13.82 (br s, 1H), 12.38 (br s, 1H), 7.71 (app t, *J* = 7.2 Hz, 1H), 7.62–7.54 (m, 1H), 7.40–7.31 (m, 2H), 3.32–3.26 (m, 1H), 1.35 (d, *J* = 7.0 Hz, 6H). ^13^C NMR (126 MHz, DMSO-*d*_6_): δ 159.5 (d, *J* = 249.6 Hz), 146.6, 132.3 (d, *J* = 8.3 Hz), 131.1 (d, *J* = 1.6 Hz), 124.6 (d, *J* = 3.3 Hz), 122.7 (d, *J* = 13.1 Hz), 116.1 (d, *J* = 21.4 Hz), 25.5, 21.9. LC–MS: *t*_R_ = 3.66 min, purity: >99%, *m*/*z* [M + H]^+^: 273; HR-MS: calcd for C_14_H_13_FN_4_O [M + H]^+^, 273.1146; found, 273.1144.

#### 5-(2-Chlorophenyl)-3-isopropyl-1,6-dihydro-7*H*-pyrazolo[4,3-*d*]pyrimidin-7-one **19** (NPD-3538)

Prepared from **8** (156 mg) via Method A as a white solid (0.15 g, 56% for two steps). ^1^H NMR (500 MHz, DMSO-*d*_6_ + 1 drop of D_2_O): δ 7.57–7.48 (m, 3H), 7.47–7.42 (m, 1H), 3.33–3.21 (m, 1H), 1.30 (d, *J* = 7.0 Hz, 6H). ^13^C NMR (126 MHz, DMSO-*d*_6_ + 1 drop of D_2_O): δ 152.5 (HMBC), 149.9 (HMBC), 144.3 (HMBC), 134.2, 132.5, 132.5, 131.8, 130.6, 128.2, 26.6 (HSQC), 22.6. LC–MS: *t*_R_ = 3.66 min, purity: >99%, *m*/*z* [M + H]^+^: 289; HR-MS: calcd for C_14_H_13_ClN_4_O [M + H]^+^, 289.0851; found, 289.0850.

#### 5-(2-Bromophenyl)-3-isopropyl-1,6-dihydro-7*H*-pyrazolo[4,3-*d*]pyrimidin-7-one **20** (NPD-3539)

Prepared from **8** (0.16 g) via Method A as a white solid (0.28 mg, 88% for two steps). ^1^H NMR (600 MHz, DMSO-*d*_6_ + 1 drop of D_2_O): δ 7.76 (dd, *J* = 8.0, 1.0 Hz, 1H), 7.59 (dd, *J* = 7.6, 1.7 Hz, 1H), 7.52 (app td, *J* = 7.5, 1.2 Hz, 1H), 7.46 (app td, *J* = 7.7, 1.8 Hz, 1H), 3.27 (hept, *J* = 6.9 Hz, 1H), 1.35 (d, *J* = 7.0 Hz, 6H). ^13^C NMR (151 MHz, DMSO-*d*_6_ + 1 drop of D_2_O): δ 150.4 (HMBC), 136.6, 133.1, 131.8, 131.6, 128.1, 122.0, 26.3, 22.3. LC–MS: *t*_R_ = 3.69 min, purity: >99%, *m*/*z* [M + H]^+^: 333; HR-MS: calcd for C_14_H_13_BrN_4_O [M + H]^+^, 333.0346; found, 333.0333.

#### 3-Isopropyl-5-(*o*-tolyl)-1,6-dihydro-7*H*-pyrazolo[4,3-*d*]pyrimidin-7-one **21** (NPD-3589)

Prepared from **8** (0.16 g) via Method A as a white solid (0.11 g, 43% for two steps). ^1^H NMR (300 MHz, DMSO-*d*_6_): δ 13.76 (br s, 1H), 12.25 (br s, 1H), 7.49–7.43 (m, 1H), 7.42–7.37 (m, 1H), 7.36–7.27 (m, 2H), 3.30–3.23 (m, 1H), 2.36 (s, 3H), 1.36 (d, *J* = 7.0 Hz, 6H). ^13^C NMR (151 MHz, DMSO-*d*_6_): δ 151.0 (HMBC), 142.4 (HMBC), 136.4, 134.4, 130.6, 129.7, 129.4, 125.8, 26.3 (HSQC), 21.9, 19.7. LC–MS: *t*_R_ = 3.79 min, purity: >99%, *m*/*z* [M + H]^+^: 269; HR-MS: calcd for C_15_H_16_N_4_O [M + H]^+^, 269.1397; found, 269.1405.

#### 3-Isopropyl-5-(2-methoxyphenyl)-1,6-dihydro-7*H*-pyrazolo[4,3-*d*]pyrimidin-7-one **22** (NPD-3590)

Prepared from **8** (0.20 g) via Method A as a white solid (17 mg, 5% for two steps). ^1^H NMR (500 MHz, DMSO-*d*_6_ + 1 drop of D_2_O): δ 7.63 (d, *J* = 7.5 Hz, 1H), 7.50 (app t, *J* = 7.8 Hz, 1H), 7.16 (d, *J* = 8.4 Hz, 1H), 7.07 (app t, *J* = 7.4 Hz, 1H), 3.83 (s, 3H), 3.28 (app s, 1H), 1.34 (d, *J* = 6.8 Hz, 6H). ^13^C NMR (126 MHz, DMSO-*d*_6_ + 1 drop of D_2_O): δ 157.0, 150.9 (HMBC), 149.1 (HMBC), 142.2 (HMBC), 131.7, 130.5, 123.1, 120.5, 111.8, 55.8, 26.1 (HSQC), 21.9. LC–MS: *t*_R_ = 3.96 min, purity: >99%, *m*/*z* [M + H]^+^: 285; HR-MS: calcd for C_15_H_16_N_4_O_2_ [M + H]^+^, 285.1346; found, 285.1333.

#### 5-(3-Fluorophenyl)-3-isopropyl-1,6-dihydro-7*H*-pyrazolo[4,3-*d*]pyrimidin-7-one **23** (NPD-3202)

Prepared from **8** (0.16 g) via Method A as a white solid (95 mg, 37% for two steps). ^1^H NMR (300 MHz, CD_3_OD): δ 7.87 (d, *J* = 7.8 Hz, 1H), 7.84–7.78 (m, 1H), 7.63–7.52 (m, 1H), 7.32 (app td, *J* = 8.5, 2.5 Hz, 1H), 3.49 (app s, 1H), 1.50 (d, *J* = 7.0 Hz, 6H). ^13^C NMR (151 MHz, CD_3_OD): δ 164.3 (d, *J* = 244.9 Hz), 153.7 (HMBC), 150.7 (HMBC), 145.1 (HMBC), 135.7 (d, *J* = 7.2 Hz), 131.8 (d, *J* = 8.3 Hz), 123.0 (app s), 118.6 (d, *J* = 21.6 Hz), 115.5 (d, *J* = 24.3 Hz), 28.1, 22.3. LC–MS: *t*_R_ = 3.98 min, purity: 98%, *m*/*z* [M + H]^+^: 273; HR-MS: calcd for C_14_H_13_FN_4_O [M + Na]^+^, 295.0966; found, 295.0954.

#### 5-(3-Chlorophenyl)-3-isopropyl-1,6-dihydro-7*H*-pyrazolo[4,3-*d*]pyrimidin-7-one **24** (NPD-3591)

Prepared from **8** (0.17 g) via Method A as a white solid (12 mg, 4% for two steps). ^1^H NMR (500 MHz, DMSO-*d*_6_ + 1 drop of D_2_O): δ 8.06 (s, 1H), 7.98 (d, *J* = 7.2 Hz, 1H), 7.62–7.52 (m, 2H), 3.34 (app s, 1H), 1.38 (d, *J* = 6.9 Hz, 6H). ^13^C NMR (126 MHz, DMSO-*d*_6_ + 1 drop of D_2_O): δ 151.9 (HMBC), 149.1 (HMBC), 143.5 (HMBC), 135.3, 133.5, 130.6, 130.4, 127.3, 126.3, 26.4 (HSQC), 22.0. LC–MS: *t*_R_ = 4.25 min, purity: 99%, *m*/*z* [M + H]^+^: 289; HR-MS: calcd for C_14_H_13_ClN_4_O [M + Na]^+^, 311.0670; found, 311.0676.

#### 3-Isopropyl-5-(*m*-tolyl)-1,6-dihydro-7*H*-pyrazolo[4,3-*d*]pyrimidin-7-one **25** (NPD-3382)

Prepared from **8** (0.15 g) via Method A as a white solid (0.15 g, 63% for two steps). ^1^H NMR (300 MHz, DMSO-*d*_6_ + 1 drop of D_2_O): δ 7.83–7.72 (m, 2H), 7.44–7.27 (m, 2H), 3.32 (app s, 1H), 2.36 (s, 3H), 1.35 (d, *J* = 6.9 Hz, 6H). ^13^C NMR (151 MHz, DMSO-*d*_6_ + 1 drop of D_2_O): δ 151.5 (HMBC), 149.7 (HMBC), 137.7, 133.1, 131.1, 128.4, 128.0, 124.7, 26.6, 21.9, 21.0. LC–MS: *t*_R_ = 4.10 min, purity: >99%, *m*/*z* [M + H]^+^: 269; HR-MS: calcd for C_15_H_16_N_4_O [M + H]^+^, 269.1397; found, 269.1386.

#### 3-Isopropyl-5-(3-methoxyphenyl)-1,6-dihydro-7*H*-pyrazolo[4,3-*d*]pyrimidin-7-one **26** (NPD-3375)

Prepared from **8** (0.18 g) via Method A as a white solid (0.10 g, 33% for two steps). ^1^H NMR (600 MHz, DMSO-*d*_6_): δ 13.73 (br s, 1H), 12.40 (br s, 1H), 7.69 (s, 1H), 7.68–7.63 (m, 1H), 7.43 (app t, *J* = 8.0 Hz, 1H), 7.10 (dd, *J* = 8.1, 2.0 Hz, 1H), 3.86 (s, 3H), 3.33 (1H, confirmed with HSQC), 1.41 (d, *J* = 7.0 Hz, 6H). ^13^C NMR (151 MHz, DMSO-*d*_6_): δ 159.3, 149.3 (HMBC), 143.0 (HMBC), 134.4, 129.7, 119.8, 116.5, 112.5, 55.3, 26.2 (HSQC), 21.9. LC–MS: *t*_R_ = 3.87 min, purity: >99%, *m*/*z* [M + H]^+^: 285; HR-MS: calcd for C_15_H_16_N_4_O_2_ [M + H]^+^, 285.1346; found, 285.1339.

#### 5-(3-Hydroxyphenyl)-3-isopropyl-1,6-dihydro-7*H*-pyrazolo[4,3-*d*]pyrimidin-7-one **27** (NPD-2974)

Prepared from **8** (80 mg) via Method A as a white solid (53 mg, 41% for two steps). ^1^H NMR (500 MHz, DMSO-*d*_6_): δ 13.72 (br s, 1H), 12.31 (br s, 1H), 9.73 (s, 1H), 7.51–7.46 (m, 2H), 7.30 (app t, *J* = 8.0 Hz, 1H), 6.94 – 6.89 (m, 1H), 3.30 (app s, 1H), 1.39 (d, *J* = 6.9 Hz, 6H). ^13^C NMR (126 MHz, DMSO-*d*_6_): δ 157.6, 154.4, 151.5, 150.1, 137.4, 134.7, 130.1, 126.1, 118.6, 118.0, 114.5, 26.6, 22.2. LC–MS: *t*_R_ = 3.23 min, purity: >99%, *m*/*z* [M + H]^+^: 271; HR-MS: calcd for C_14_H_14_N_4_O_2_ [M + H]^+^, 271.1190; found, 271.1185.

#### 5-(3-(Dimethylamino)phenyl)-3-isopropyl-1,6-dihydro-7*H*-pyrazolo[4,3-*d*]pyrimidin-7-one **28** (NPD-3381)

Prepared from **8** (0.15 g) via Method A as a white solid (0.12 g, 45% for two steps). ^1^H NMR (600 MHz, DMSO-*d*_6_): δ 13.67 (br s, 1H), 12.33 (br s, 1H), 7.38 (d, *J* = 7.3 Hz, 2H), 7.30 (app t, *J* = 7.9 Hz, 1H), 6.86 (d, *J* = 8.7 Hz, 1H), 3.33 (1H, confirmed with D_2_O), 2.98 (s, 6H), 1.40 (d, *J* = 6.9 Hz, 6H). ^13^C NMR (151 MHz, DMSO-*d*_6_): δ 154.2, 151.0, 150.4, 142.3 (HMBC), 137.1, 129.1, 125.9, 115.4, 114.3, 111.1, 40.2, 26.3, 21.9. LC–MS: *t*_R_ = 3.71 min, purity: >99%, *m*/*z* [M + H]^+^: 298; HR-MS: calcd for C_16_H_19_N_5_O [M + H]^+^, 298.1662; found, 298.1662.

#### 3-Isopropyl-5-(3-(methylsulfonyl)phenyl)-1,6-dihydro-7*H*-pyrazolo[4,3-*d*]pyrimidin-7-one **29** (NPD-3598)

Prepared from **8** (0.13 g) via Method A as a white solid (61 mg, 24% for two steps). ^1^H NMR (500 MHz, DMSO-*d*_6_ + 1 drop of D_2_O): δ 8.54 (s, 1H), 8.37 (d, *J* = 7.8 Hz, 1H), 8.05 (d, *J* = 7.8 Hz, 1H), 7.80 (app t, *J* = 7.8 Hz, 1H), 3.35 (hept, *J* = 6.1 Hz, 1H), 3.27 (s, 3H), 1.38 (d, *J* = 7.0 Hz, 6H). ^13^C NMR (126 MHz, DMSO-*d*_6_ + 1 drop of D_2_O): δ 151.6 (HMBC), 148.8 (HMBC), 143.2 (HMBC), 141.4, 134.7, 132.9, 130.3, 129.1, 126.4, 43.8, 26.3 (HSQC), 22.2. LC–MS: *t*_R_ = 3.29 min, purity: 95%, *m*/*z* [M + H]^+^: 333; HR-MS: calcd for C_15_H_16_N_4_O_3_S [M + H]^+^, 333.1016; found, 333.1012.

#### 5-(4-Fluorophenyl)-3-isopropyl-1,6-dihydro-7*H*-pyrazolo[4,3-*d*]pyrimidin-7-one **30** (NPD-2975)

Prepared from **8** (80 mg) via Method A as a white solid (80 mg, 62% for two steps). ^1^H NMR (600 MHz, DMSO-*d*_6_): δ 13.73 (br s, 1H), 12.38 (br s, 1H), 8.18–8.12 (m, 2H), 7.39–7.33 (m, 2H), 3.36–3.32 (m, 1H), 1.39 (d, *J* = 7.0 Hz, 6H). ^13^C NMR (151 MHz, DMSO-*d*_6_): δ 163.5 (d, *J* = 248.2 Hz), 151.4 (HMBC), 148.8 (HMBC), 130.0 (d, *J* = 8.8 Hz), 129.8 (d, *J* = 2.7 Hz), 115.5 (d, *J* = 22.1 Hz), 26.2 (HSQC), 21.9. LC–MS: *t*_R_ = 3.89 min, purity: >99%, *m*/*z* [M + H]^+^: 273; HR-MS: calcd for C_14_H_13_FN_4_O [M + H]^+^, 273.1146; found, 273.1144; Anal. calcd for C_14_H_13_FN_4_O: C, 61.76; H, 4.81; N, 20.58; O, 5.88. Found: C, 61.92; H, 4.89; N, 20.5; O 6.52. More detailed 2D NMR analysis is attached in the Supporting Information.

#### 5-(4-Chlorophenyl)-3-isopropyl-1,6-dihydro-7*H*-pyrazolo[4,3-*d*]pyrimidin-7-one **31** (NPD-3204)

Prepared from **8** (0.16 g) via Method A as a white solid (0.10 g, 37% for two steps). ^1^H NMR (300 MHz, CD_3_OD): δ 8.01 (d, *J* = 8.1 Hz, 2H), 7.55 (d, *J* = 8.2 Hz, 2H), 3.56–3.37 (m, 1H), 1.47 (d, *J* = 6.6 Hz, 6H). ^13^C NMR (126 MHz, CD_3_OD): δ 138.0, 133.5, 130.2, 130.0, 28.0, 22.3. LC–MS: *t*_R_ = 4.32 min, purity: 98%, *m*/*z* [M + H]^+^: 289; HR-MS: calcd for C_14_H_13_ClN_4_O [M + H]^+^, 289.0851; found, 289.0839.

#### 5-(4-Bromophenyl)-3-isopropyl-1,6-dihydro-7*H*-pyrazolo[4,3-*d*]pyrimidin-7-one **32** (NPD-2971)

Prepared from **8** (80 mg) via Method A as a white solid (86 mg, 54% for two steps). ^1^H NMR (500 MHz, DMSO-*d*_6_ + 1 drop of D_2_O): δ 8.00 (d, *J* = 8.7 Hz, 2H), 7.72 (d, *J* = 8.7 Hz, 2H), 3.32 (hept, *J* = 7.0 Hz, 1H), 1.38 (d, *J* = 6.9 Hz, 6H). ^13^C NMR (126 MHz, DMSO-*d*_6_): δ 148.8, 132.5, 131.6, 129.6, 124.3, 29.1, 21.9. LC–MS: *t*_R_ = 4.38 min, purity: >99%, *m*/*z* [M + H]^+^: 333; HR-MS: calcd for C_14_H_13_BrN_4_O [M + H]^+^, 333.0346; found, 333.0347.

#### 3-Isopropyl-5-(4-methoxyphenyl)-1,6-dihydro-7*H*-pyrazolo[4,3-*d*]pyrimidin-7-one **33** (NPD-2972)

Prepared from **8** (80 mg) via Method A as a white solid (79 mg, 58% for two steps). ^1^H NMR (500 MHz, DMSO-*d*_6_ + 1 drop of D_2_O): δ 8.08 – 8.02 (m, 2H), 7.05 (d, *J* = 8.8 Hz, 2H), 3.82 (s, 3H), 3.31 (app s, 1H), 1.38 (d, *J* = 6.9 Hz, 6H). ^13^C NMR (126 MHz, DMSO-*d*_6_): δ 161.2, 129.1, 125.4, 114.0, 55.4, 26.3, 21.9. LC–MS: *t*_R_ = 3.86 min, purity: >99%, *m*/*z* [M + H]^+^: 285; HR-MS: calcd for C_15_H_16_N_4_O_2_ [M + H]^+^, 285.1346; found, 285.1343.

#### 5-(4-Isopropoxyphenyl)-3-isopropyl-1,6-dihydro-7*H*-pyrazolo[4,3-*d*]pyrimidin-7-one **34** (NPD-3377)

Prepared from **8** (0.15 g) via Method A as a white solid (0.10 g, 36% for two steps). ^1^H NMR (600 MHz, DMSO-*d*_6_): δ 13.68 (br s, 1H), 12.21 (br s, 1H), 8.05 (d, *J* = 8.8 Hz, 2H), 7.03 (d, *J* = 8.8 Hz, 2H), 4.73 (hept, *J* = 5.9 Hz, 1H), 3.31 (app s, 1H), 1.40 (d, *J* = 6.9 Hz, 6H), 1.30 (d, *J* = 6.0 Hz, 6H). ^13^C NMR (151 MHz, DMSO-*d*_6_): δ 159.4, 151.1 (HMBC), 149.3 (HMBC), 129.1, 125.0, 115.2, 69.4, 26.3 (HSQC), 21.9, 21.8. LC–MS: *t*_R_ = 4.40 min, purity: >99%, *m*/*z* [M + H]^+^: 313; HR-MS: calcd for C_17_H_20_N_4_O_2_ [M + H]^+^, 313.1659; found, 313.1651.

#### 3-Isopropyl-5-(4-(trifluoromethyl)phenyl)-1,6-dihydro-7*H*-pyrazolo[4,3-*d*]pyrimidin-7-one **35** (NPD-3201)

Prepared from **8** (0.16 g) via Method A as a white solid (0.13 g, 42% for two steps). ^1^H NMR (300 MHz, CD_3_OD): δ 8.16 (d, *J* = 6.9 Hz, 2H), 7.80 (d, *J* = 7.4 Hz, 2H), 3.52–3.36 (m, 1H), 1.43 (d, *J* = 5.7 Hz, 6H). ^13^C NMR (126 MHz, CD_3_OD): δ 154.1 (HMBC), 150.7 (HMBC), 145.5 (HMBC), 138.5, 133.3 (q, *J* = 32.6 Hz), 129.4, 126.7 (q, *J* = 3.6 Hz), 125.4 7 (q, *J* = 271.8 Hz), 28.1 (HSQC), 22.3. LC–MS: *t*_R_ = 4.43 min, purity: >99%, *m*/*z* [M + H]^+^: 323; HR-MS: calcd for C_15_H_13_F_3_N_4_O [M + Na]^+^, 345.0934; found, 345.0920.

#### 3-Isopropyl-5-(4-(trifluoromethoxy)phenyl)-1,6-dihydro-7*H*-pyrazolo[4,3-*d*]pyrimidin-7-one **36** (NPD-3597)

Prepared from **8** (0.20 g) via Method A as a white solid (60 mg, 15% for two steps). ^1^H NMR (500 MHz, DMSO-*d*_6_ + 1 drop of D_2_O): δ 8.16–8.09 (m, 2H), 7.49 (d, *J* = 8.1 Hz, 2H), 3.32 (app s, 1H), 1.36 (d, *J* = 6.7 Hz, 6H). ^13^C NMR (126 MHz, DMSO-*d*_6_ + 1 drop of D_2_O): δ 151.3 (HMBC), 149.8, 132.6, 129.8, 123.1 (HMBC), 120.4 (q, *J* = 257.1 Hz), 26.4 (HSQC), 21.9. LC–MS: *t*_R_ = 4.54 min, purity: >99%, *m*/*z* [M + H]^+^: 339; HR-MS: calcd for C_15_H_13_F_3_N_4_O_2_ [M + H]^+^, 339.1063; found, 339.1069.

#### 4-(3-Isopropyl-7-oxo-6,7-dihydro-1*H*-pyrazolo[4,3-*d*]pyrimidin-5-yl)benzonitrile **37** (NPD-3203)

Prepared from **8** (0.16 g) via Method A as a white solid (98 mg, 37% for two steps). ^1^H NMR (300 MHz, CD_3_OD): δ 8.20 (d, *J* = 8.4 Hz, 2H), 7.90 (d, *J* = 8.5 Hz, 2H), 3.46 (hept, *J* = 7.1 Hz, 1H), 1.48 (d, *J* = 7.0 Hz, 6H). ^13^C NMR (126 MHz, CD_3_OD): δ 137.6, 132.2, 128.0, 117.8, 113.8, 20.9. LC–MS: *t*_R_ = 3.69 min, purity: >99%, *m*/*z* [M + H]^+^: 280; HR-MS: calcd for C_15_H_13_N_5_O [M + H]^+^, 280.1193; found, 280.1182.

#### Methyl 4-(3-isopropyl-7-oxo-6,7-dihydro-1*H*-pyrazolo[4,3-*d*]pyrimidin-5-yl)benzoate **38** (NPD-3305)

Prepared from **8** (0.15 g) via Method B as a white solid (95 mg, 34%). ^1^H NMR (600 MHz, DMSO-*d*_6_): δ 13.79 (s, 1H), 12.57 (s, 1H), 8.21 (d, *J* = 8.3 Hz, 2H), 8.08 (d, *J* = 8.3 Hz, 2H), 3.89 (s, 3H), 3.33 (1H, confirmed with HSQC), 1.39 (d, *J* = 6.9 Hz, 6H). ^13^C NMR (151 MHz, DMSO-*d*_6_): δ 165.7, 154.0, 151.3, 148.7, 137.2, 136.9, 131.1, 129.3, 127.9, 126.1, 52.4, 26.3, 21.9. LC–MS: *t*_R_ = 3.83 min, purity: 97%, *m*/*z* [M + H]^+^: 313; HR-MS: calcd for C_16_H_16_N_4_O_3_ [M + H]^+^, 313.1295; found, 313.1284.

#### 4-(3-Isopropyl-7-oxo-6,7-dihydro-1*H*-pyrazolo[4,3-*d*]pyrimidin-5-yl)benzoic Acid **39** (NPD-3489)

Ester **38** (0.28 g, 0.89 mmol) and LiOH (37 mg, 0.89 mmol) were added to a mixture of 1,4-dioxane (10 mL) and water (10 mL) and refluxed for 2 h. The reaction mixture was concentrated under reduced pressure and washed with EtOAc (3 × 20 mL); the pH was adjusted to 1 with 1 M HCl aqueous solution, and the product was filtered as an off-white solid (0.20 g, 75%). ^1^H NMR (600 MHz, DMSO-*d*_6_): δ 13.80 (br s, 1H), 13.20 (br s, 1H), 12.47 (br s, 1H), 8.18 (d, *J* = 8.5 Hz, 2H), 8.05 (d, *J* = 8.5 Hz, 2H), 3.33 (1H, confirmed with HSQC), 1.40 (d, *J* = 7.0 Hz, 6H). ^13^C NMR (151 MHz, DMSO-*d*_6_): δ 166.8, 151.2 (HMBC), 148.7 (HMBC), 136.9, 132.3, 129.4, 127.7, 26.0 (HSQC), 21.9. LC–MS: *t*_R_ = 3.16 min, purity: 99%, *m*/*z* [M + H]^+^: 299; HR-MS: calcd for C_15_H_14_N_4_O_3_ [M + H]^+^, 299.1139; found, 299.1101.

#### 4-(3-Isopropyl-7-oxo-6,7-dihydro-1*H*-pyrazolo[4,3-*d*]pyrimidin-5-yl)benzamide **40** (NPD-3371)

Prepared from **8** (0.15 g) via Method A as a white solid (49 mg, 18% for two steps). ^1^H NMR (300 MHz, DMSO-*d*_6_): δ 13.77 (br s, 1H), 12.45 (br s, 1H), 8.19–8.06 (m, 3H), 7.99 (d, *J* = 8.1 Hz, 2H), 7.51 (s, 1H), 3.33 (1H, confirmed with HSQC), 1.40 (d, *J* = 6.8 Hz, 6H). ^13^C NMR (151 MHz, DMSO-*d*_6_): δ 167.2, 151.3 (HMBC), 149.0 (HMBC), 142.9 (HMBC), 135.8, 135.5, 127.6, 127.3, 26.3 (HMBC), 21.9. LC–MS: *t*_R_ = 2.76 min, purity: 97%, *m*/*z* [M + H]^+^: 298; HR-MS: calcd for C_15_H_15_N_5_O_2_ [M + Na]^+^, 320.1118; found, 320.1105.

#### 3-Isopropyl-5-(4-(methylsulfonyl)phenyl)-1,6-dihydro-7*H*-pyrazolo[4,3-*d*]pyrimidin-7-one **41** (NPD-3376)

Prepared from **8** (0.15 g) via Method A as a white solid (90 mg, 30% for two steps). ^1^H NMR (600 MHz, DMSO-*d*_6_): δ 13.82 (br s, 1H), 12.64 (br s, 1H), 8.30 (d, *J* = 8.5 Hz, 2H), 8.06 (d, *J* = 8.3 Hz, 2H), 3.33 (1H, confirmed with HSQC), 3.29 (s, 3H), 1.40 (d, *J* = 6.9 Hz, 6H). ^13^C NMR (151 MHz, DMSO-*d*_6_): δ 154.0 (HMBC), 151.4 (HMBC), 148.3 (HMBC), 143.6, 142.2, 137.7, 128.5, 127.1, 43.3, 26.3, 21.9. LC–MS: *t*_R_ = 3.27 min, purity: 97%, *m*/*z* [M + H]^+^: 333; HR-MS: calcd for C_15_H_16_N_4_O_3_S [M + H]^+^, 333.1016; found, 333.1011.

#### 4-(3-Isopropyl-7-oxo-6,7-dihydro-1*H*-pyrazolo[4,3-*d*]pyrimidin-5-yl)benzenesulfonamide **42** (NPD-3372)

Prepared from **8** (0.15 g) via Method A as a white solid (56 mg, 19% for two steps). ^1^H NMR (600 MHz, DMSO-*d*_6_): δ 13.82 (br s, 1H), 12.56 (br s, 1H), 8.23 (d, *J* = 8.4 Hz, 2H), 7.95 (d, *J* = 8.3 Hz, 2H), 7.51 (s, 2H), 3.33 (1H, confirmed with HSQC), 1.41 (d, *J* = 6.9 Hz, 6H). ^13^C NMR (151 MHz, DMSO-*d*_6_): δ 151.3 (HMBC), 145.6, 136.1, 128.2, 125.8, 26.3 (HSQC), 21.9. LC–MS: *t*_R_ = 2.96 min, purity: >99%, *m*/*z* [M + H]^+^: 334; HR-MS: calcd for C_14_H_15_N_5_O_3_S [M + H]^+^, 334.0969; found, 334.0953.

#### *N*-(4-(3-Isopropyl-7-oxo-6,7-dihydro-1*H*-pyrazolo[4,3-*d*]pyrimidin-5-yl)phenyl)acetamide **43** (NPD-3280)

Prepared from **8** (0.15 g) via Method B as a white solid (0.13 g, 47%). ^1^H NMR (600 MHz, DMSO-*d*_6_): δ 13.66 (br s, 1H), 12.26 (br s, 1H), 10.18 (s, 1H), 8.03 (d, *J* = 8.7 Hz, 2H), 7.70 (d, *J* = 8.5 Hz, 2H), 3.37–3.34 (m, 1H), 2.08 (s, 3H), 1.39 (d, *J* = 6.9 Hz, 6H). ^13^C NMR (151 MHz, DMSO-*d*_6_): δ 168.7, 150.9 (HMBC), 149.4 (HMBC), 142.4 (HMBC), 141.4, 128.1, 127.4, 118.4, 27.3 (HSQC), 24.1, 21.9. LC–MS: *t*_R_ = 3.06 min, purity: >99%, *m*/*z* [M + H]^+^: 312; HR-MS: calcd for C_16_H_17_N_5_O_2_ [M + H]^+^, 312.1455; found, 312.1443.

#### 3-Isopropyl-5-(4-(piperidin-1-yl)phenyl)-1,6-dihydro-7*H*-pyrazolo[4,3-*d*]pyrimidin-7-one **44** (NPD-3283)

Prepared from **8** (0.15 g) via Method B as a white solid (70 mg, 23%). ^1^H NMR (300 MHz, DMSO-*d*_6_): δ 13.62 (br s, 1H), 12.03 (br s, 1H), 7.97 (d, *J* = 8.9 Hz, 2H), 7.00 (d, *J* = 9.0 Hz, 2H), 3.32–3.26 (m, 5H), 1.60 (app s, 6H), 1.39 (d, *J* = 7.0 Hz, 6H). ^13^C NMR (151 MHz, DMSO-*d*_6_): δ 152.5, 150.6 (HMBC), 141.8 (HMBC), 128.4, 121.5, 114.1, 48.3, 26.3 (HMBC), 24.9, 24.0, 21.9. LC–MS: *t*_R_ = 4.34 min, purity: >99%, *m*/*z* [M + H]^+^: 338; HR-MS: calcd for C_19_H_23_N_5_O [M + H]^+^, 338.1975; found, 338.1964.

#### 3-Isopropyl-5-(4-(4-methylpiperazin-1-yl)phenyl)-1,6-dihydro-7*H*-pyrazolo[4,3-*d*]pyrimidin-7-one **45** (NPD-3282)

Prepared from **8** (0.15 g) via Method B as a white solid (0.11 g, 35%). ^1^H NMR (600 MHz, CDCl_3_): δ 10.78 (br s, 1H), 7.89 (d, *J* = 7.9 Hz, 2H), 6.87 (app s, 2H), 3.48 (hept, *J* = 6.6 Hz, 1H), 3.22 (app s, 4H), 2.53 (app s, 4H), 2.33 (s, 3H), 1.51 (d, *J* = 6.9 Hz, 6H). ^13^C NMR (151 MHz, CDCl_3_): δ 155.8, 152.9, 152.2 (HMBC), 149.5, 139.0, 128.3, 122.7, 114.8, 54.8, 47.7, 46.2, 26.9, 22.0. LC–MS: *t*_R_ = 2.53 min, purity: 98%, *m*/*z* [M + H]^+^: 353; HR-MS: calcd for C_19_H_24_N_6_O [M + H]^+^, 353.2084; found, 353.2078.

#### 5-(4-(1*H*-Tetrazol-5-yl)phenyl)-3-isopropyl-1,6-dihydro-7*H*-pyrazolo[4,3-*d*]pyrimidin-7-one **46** (NPD-3490)

A mixture of **37** (0.15 g, 0.54 mmol), NaN_3_ (0.56 g, 8.6 mmol), and NH_4_Cl (0.46 g, 8.6 mmol) in DMF (5 mL) was heated using microwave irradiation at 160 °C for 2 h, after which the reaction mixture was dissolved in water (50 mL) and extracted with EtOAc (3 × 50 mL). The combined organic layers were washed with brine, concentrated in vacuo, and purified by flash column chromatography on silica gel with a gradient elution of EtOAc in cyclohexane (20%–50%) to give the title compound as a white solid (0.12 g, 69%). ^1^H NMR (600 MHz, DMSO-*d*_6_): δ 13.80 (br s, 1H), 12.57 (br s, 1H), 8.31 (d, *J* = 8.2 Hz, 2H), 8.19 (d, *J* = 8.0 Hz, 2H), 3.33 (1H, confirmed with HSQC), 1.42 (d, *J* = 6.7 Hz, 6H). ^13^C NMR (151 MHz, DMSO-*d*_6_): δ 151.2 (HMBC), 148.7 (HMBC), 142.6 (HMBC), 135.4, 128.5, 127.1, 125.8 (HMBC), 26.3 (HMBC), 21.9. LC–MS: *t*_R_ = 3.10 min, purity: >99%, *m*/*z* [M + H]^+^: 323; HR-MS: calcd for C_15_H_14_N_8_O [M + H]^+^, 323.1363; found, 323.1357.

## Supporting Information Available

Structures of approved HAT treatments; physicochemical properties of **1** (BIPPO); phenotypic activity of close BIPPO analogues against *T. b. brucei*, *T. cruzi*, and *L. infantum*; antitrypanosomal potency of **23**, **30**, and **31**; anti-*Tbr*PDEB1 activity of **30**; safety profile of analogue **30** (NPD-2975) from Eurofins; in vitro metabolic stability of **30** using mouse, rat, and human S9 microsomal fractions; chemical characterization of final compounds; antitrypanosomal potency of final compounds; and LCMS, ^1^H NMR, ^13^C NMR, and 2D NMR spectroscopy data. (PDF)

## Abbreviations

ADQUATE: adequate sensitivity double-quantum spectroscopy
CDD: collaborative drug discovery
dpi: days post infection
HAT: human African Trypanosomiasis
MRC-5: medical research council cell strain 5
NPD: neglected parasitic disease
PDE: phosphodiesterase
PMM: peritoneal macrophage
RT: room temperature
UGT: uridine 5′-diphospho-glucuronosyltransferase
WHO: World Health Organization.

## References

[ref1] Kennedy P. G. E. Clinical Features, Diagnosis, and Treatment of Human African Trypanosomiasis (Sleeping Sickness). Lancet Neurol. 2013, 12, 186–194. 10.1016/s1474-4422(12)70296-x. 23260189

[ref2] World Health Organization. Trypanosomiasis. https://www.who.int/health-topics/human-african-trypanosomiasis (accessed December 15, 2022).

[ref3] Büscher P.; Cecchi G.; Jamonneau V.; Priotto G. Human African Trypanosomiasis. Lancet 2017, 390, 2397–2409. 10.1016/s0140-6736(17)31510-6. 28673422

[ref4] WHO. WHO HAT 2020. https://www.who.int/news-room/fact-sheets/detail/trypanosomiasis-human-african-(sleeping-sickness) (accessed 04 18, 2022).

[ref5] Lindner A. K.; Lejon V.; Chappuis F.; Seixas J.; Kazumba L.; Barrett M. P.; Mwamba E.; Erphas O.; Akl E. A.; Villanueva G.; Bergman H.; Simarro P.; Kadima Ebeja A.; Priotto G.; Franco J. R. New WHO Guidelines for Treatment of Gambiense Human African Trypanosomiasis Including Fexinidazole: Substantial Changes for Clinical Practice. Lancet Infect. Dis. 2020, 20, e38–e46. 10.1016/s1473-3099(19)30612-7. 31879061

[ref6] De Koning H. P. The Drugs of Sleeping Sickness: their Mechanisms of Action and Resistance, and a Brief History. Trop. Med. Infect. Dis. 2020, 5, 14 10.3390/tropicalmed5010014. 31963784 PMC7157662

[ref7] Franco J.; Scarone L.; Comini M. A. Drugs and Drug Resistance in African and American Trypanosomiasis. Annu. Rep. Med. Chem. 2018, 51, 97–133. 10.1016/BS.ARMC.2018.08.003.

[ref8] Benaim G.; Lopez-Estrano C.; Docampo R.; Moreno S. N. J. A Calmodulin-Stimulated Ca2+ Pump in Plasma-Membrane Vesicles from Trypanosoma Brucei; Selective Inhibition by Pentamidine. Biochem. J. 1993, 296, 759–763. 10.1042/bj2960759. 8280074 PMC1137760

[ref9] Fairlamb A. H.; Henderson G. B.; Cerami A. Trypanothione Is the Primary Target for Arsenical Drugs against African Trypanosomes. Proc. Natl. Acad. Sci. U.S.A. 1989, 86, 2607–2611. 10.1073/pnas.86.8.2607. 2704738 PMC286966

[ref10] Baumann H.; Strassmann G. Suramin Inhibits the Stimulation of Acute Phase Plasma Protein Genes by IL-6-Type Cytokines in Rat Hepatoma Cells. J. Immunol. 1993, 151, 1456–1462. 10.4049/jimmunol.151.3.1456. 7687632

[ref11] Vincent I. M.; Creek D.; Watson D. G.; Kamleh M. A.; Woods D. J.; Wong P. E.; Burchmore R. J. S.; Barrett M. P. A Molecular Mechanism for Eflornithine Resistance in African Trypanosomes. PLoS Pathog. 2010, 6, e1001204 10.1371/journal.ppat.1001204. 21124824 PMC2991269

[ref12] Wilkinson S. R.; Taylor M. C.; Horn D.; Kelly J. M.; Cheeseman I. A Mechanism for Cross-Resistance to Nifurtimox and Benznidazole in Trypanosomes. Proc. Natl. Acad. Sci. U.S.A. 2008, 105, 5022–5027. 10.1073/pnas.0711014105. 18367671 PMC2278226

[ref13] Wyllie S.; Foth B. J.; Kelner A.; Sokolova A. Y.; Berriman M.; Fairlamb A. H. Nitroheterocyclic Drug Resistance Mechanisms in Trypanosoma Brucei. J. Antimicrob. Chemother. 2016, 71, 625–634. 10.1093/jac/dkv376. 26581221 PMC4743696

[ref14] Casulli A. New Global Targets for NTDs in the WHO Roadmap 2021–2030. PLoS Neglected Trop. Dis. 2021, 15, e0009373 10.1371/journal.pntd.0009373. PMC8118239 33983940

[ref15] De Rycker M.; Wyllie S.; Horn D.; Read K. D.; Gilbert I. H. Anti-Trypanosomatid Drug Discovery: Progress and Challenges. Nat. Rev. Microbiol. 2022, 21, 35–50. 10.1038/s41579-022-00777-y. 35995950 PMC9395782

[ref16] Nagle A. S.; Khare S.; Kumar A. B.; Supek F.; Buchynskyy A.; Mathison C. J. N.; Chennamaneni N. K.; Pendem N.; Buckner F. S.; Gelb M. H.; Molteni V. Recent Developments in Drug Discovery for Leishmaniasis and Human African Trypanosomiasis. Chem. Rev. 2014, 114, 11305–11347. 10.1021/cr500365f. 25365529 PMC4633805

[ref17] Altamura F.; Rajesh R.; Catta-Preta C. M. C.; Moretti N. S.; Cestari I. The Current Drug Discovery Landscape for Trypanosomiasis and Leishmaniasis: Challenges and Strategies to Identify Drug Targets. Drug Dev. Res. 2022, 83, 225–252. 10.1002/ddr.21664. 32249457

[ref18] Field M. C.; Horn D.; Fairlamb A. H.; Ferguson M. A. J.; Gray D. W.; Read K. D.; de Rycker M.; Torrie L. S.; Wyatt P. G.; Wyllie S.; Gilbert I. H. Anti-Trypanosomatid Drug Discovery: An Ongoing Challenge and a Continuing Need. Nat. Rev. Microbiol. 2017, 15, 217–231. 10.1038/nrmicro.2016.193. 28239154 PMC5582623

[ref19] Oberholzer M.; Marti G.; Baresic M.; Kunz S.; Hemphill A.; Seebeck T. The Trypanosoma Brucei CAMP Phosphodiesterases TbrPDEBl and TbrPDEB2: Flagellar Enzymes That Are Essential for Parasite Virulence. FASEB J. 2007, 21, 720–731. 10.1096/fj.06-6818com. 17167070

[ref20] Kunz S.; Balmer V.; Sterk G. J.; Pollastri M. P.; Leurs R.; Müller N.; Hemphill A.; Spycher C. The Single Cyclic Nucleotide-Specific Phosphodiesterase of the Intestinal Parasite Giardia Lamblia Represents a Potential Drug Target. PLoS Neglected Trop. Dis. 2017, 11, e0005891 10.1371/journal.pntd.0005891. PMC5617230 28915270

[ref21] Wang H.; Yan Z.; Geng J.; Kunz S.; Seebeck T.; Ke H. Crystal Structure of the Leishmania Major Phosphodiesterase LmjPDEB1 and Insight into the Design of the Parasite-Selective Inhibitors. Mol. Microbiol. 2007, 66, 1029–1038. 10.1111/j.1365-2958.2007.05976.x. 17944832 PMC2950065

[ref22] Long T.; Rojo-Arreola L.; Shi D.; El-Sakkary N.; Jarnagin K.; Rock F.; Meewan M.; Rascón A. A.; Lin L.; Cunningham K. A.; Lemieux G. A.; Podust L.; Abagyan R.; Ashrafi K.; McKerrow J. H.; Caffrey C. R. Phenotypic, Chemical and Functional Characterization of Cyclic Nucleotide Phosphodiesterase 4 (PDE4) as a Potential Anthelmintic Drug Target. PLoS Neglected Trop. Dis. 2017, 11, e0005680 10.1371/journal.pntd.0005680. PMC5526615 28704396

[ref23] Boolell M.; Allen M. J.; Ballard S. A.; Gepi-Attee S.; Muirhead G. J.; Naylor A. M.; Osterloh I. H.; Gingell C. S. An Orally Active Type 5 Cyclic GMP-Specific Phosphodiesterase Inhibitor for the Treatment of Penile Erectile Dysfunction. Int. J. Impot. Res. 1996, 8, 47–52. 8858389

[ref24] Eruopean Commision. PDE4NPD, 2018 https://cordis.europa.eu/project/id/602666.

[ref25] Blaazer A. R.; Singh A. K.; De Heuvel E.; Edink E.; Orrling K. M.; Veerman J. J. N.; Van Den Bergh T.; Jansen C.; Balasubramaniam E.; Mooij W. J.; Custers H.; Sijm M.; Tagoe D. N. A.; Kalejaiye T. D.; Munday J. C.; Tenor H.; Matheeussen A.; Wijtmans M.; Siderius M.; De Graaf C.; Maes L.; De Koning H. P.; Bailey D. S.; Sterk G. J.; De Esch I. J. P.; Brown D. G.; Leurs R. Targeting a Subpocket in Trypanosoma Brucei Phosphodiesterase B1 (TbrPDEB1) Enables the Structure-Based Discovery of Selective Inhibitors with Trypanocidal Activity. J. Med. Chem. 2018, 61, 3870–3888. 10.1021/acs.jmedchem.7b01670. 29672041 PMC5949723

[ref26] Jansen C.; Wang H.; Kooistra A. J.; De Graaf C.; Orrling K. M.; Tenor H.; Seebeck T.; Bailey D.; De Esch I. J. P.; Ke H.; Leurs R. Discovery of Novel Trypanosoma Brucei Phosphodiesterase B1 Inhibitors by Virtual Screening against the Unliganded TbrPDEB1 Crystal Structure. J. Med. Chem. 2013, 56, 2087–2096. 10.1021/jm3017877. 23409953 PMC3635145

[ref27] Howard B. L.; Harvey K. L.; Stewart R. J.; Azevedo M. F.; Crabb B. S.; Jennings I. G.; Sanders P. R.; Manallack D. T.; Thompson P. E.; Tonkin C. J.; Gilson P. R. Identification of Potent Phosphodiesterase Inhibitors That Demonstrate Cyclic Nucleotide-Dependent Functions in Apicomplexan Parasites. ACS Chem. Biol. 2015, 10, 1145–1154. 10.1021/cb501004q. 25555060

[ref28] Newkirk R.; Maenz D.; Classen H. Oilseed processing. U.S. patent, 0,124,222A1, 2003, 1 (19), 1–4.

[ref29] DeNinno M. P.; Andrews M.; Bell A. S.; Chen Y.; Eller-Zarbo C.; Eshelby N.; Etienne J. B.; Moore D. E.; Palmer M. J.; Visser M. S.; Yu L. J.; Zavadoski W. J.; Michael Gibbs E. The Discovery of Potent, Selective, and Orally Bioavailable PDE9 Inhibitors as Potential Hypoglycemic Agents. Bioorg. Med. Chem. Lett. 2009, 19, 2537–2541. 10.1016/j.bmcl.2009.03.024. 19339180

[ref30] Moravcová D.; Havlicek L.; Krystof V.; Lenobel R.; Strnad M. Pyrazolo[4,3-d]Pyrimidines, Processes for Their Preparation and Methods for Therapy. EP1348707A1, 2003.

[ref31] Dale D. J.; Dunn P. J.; Golightly C.; Hughes M. L.; Levett P. C.; Pearce A. K.; Searle P. M.; Ward G.; Wood A. S. The Chemical Development of the Commercial Route to Sildenafil: A Case History. Org. Process Res. Dev. 2000, 4, 17–22. 10.1021/op9900683.

[ref32] de Koning H. P.; Gould M. K.; Sterk G. J.; Tenor H.; Kunz S.; Luginbuehl E.; Seebeck T. Pharmacological Validation of Trypanosoma Brucei Phosphodiesterases as Novel Drug Targets. J. Infect. Dis. 2012, 206, 229–237. 10.1093/infdis/jir857. 22291195 PMC3379837

[ref33] Hulpia F.; Mabille D.; Campagnaro G. D.; Schumann G.; Maes L.; Roditi I.; Hofer A.; de Koning H. P.; Caljon G.; Van Calenbergh S. Combining Tubercidin and Cordycepin Scaffolds Results in Highly Active Candidates to Treat Late-Stage Sleeping Sickness. Nat. Commun. 2019, 10, 5564–5611. 10.1038/s41467-019-13522-6. 31804484 PMC6895180

[ref34] Creek D. J.; Mazet M.; Achcar F.; Anderson J.; Kim D.-H.; Kamour R.; Morand P.; Millerioux Y.; Biran M.; Kerkhoven E. J.; Chokkathukalam A.; Weidt S. K.; Burgess K. E. V.; Breitling R.; Watson D. G.; Bringaud F.; Barrett M. P. Probing the Metabolic Network in Bloodstream-Form Trypanosoma Brucei Using Untargeted Metabolomics with Stable Isotope Labelled Glucose. PLoS Pathog. 2015, 11, e1004689 10.1371/journal.ppat.1004689. 25775470 PMC4361558

[ref35] Vincent I. M.; Creek D. J.; Burgess K.; Woods D. J.; Burchmore R. J. S.; Barrett M. P. Untargeted Metabolomics Reveals a Lack Of Synergy between Nifurtimox and Eflornithine against Trypanosoma Brucei. PLoS Neglected Trop. Dis. 2012, 6, e1618 10.1371/journal.pntd.0001618. PMC3341325 22563508

[ref36] Schumann Burkard G.; Jutzi P.; Roditi I. Genome-Wide RNAi Screens in Bloodstream Form Trypanosomes Identify Drug Transporters. Mol. Biochem. Parasitol. 2011, 175, 91–94. 10.1016/j.molbiopara.2010.09.002. 20851719

[ref37] Klug D. M.; Tschiegg L.; Diaz R.; Rojas-Barros D.; Perez-Moreno G.; Ceballos G.; García-Hernández R.; Martinez-Martinez M. S.; Manzano P.; Ruiz L. M.; Caffrey C. R.; Gamarro F.; Pacanowska D. G.; Ferrins L.; Navarro M.; Pollastri M. P. Hit-to-Lead Optimization of Benzoxazepinoindazoles As Human African Trypanosomiasis Therapeutics. J. Med. Chem. 2020, 63, 2527–2546. 10.1021/acs.jmedchem.9b01506. 31670951 PMC8329730

[ref38] Hulpia F.; Bouton J.; Campagnaro G. D.; Alfayez I. A.; Mabille D.; Maes L.; de Koning H. P.; Caljon G.; Van Calenbergh S.; Van Calenbergh S. C6–O-Alkylated 7-Deazainosine Nucleoside Analogues: Discovery of Potent and Selective Anti-Sleeping Sickness Agents. Eur. J. Med. Chem. 2020, 188, 112018 10.1016/j.ejmech.2019.112018. 31931339

[ref39] Pedron J.; Boudot C.; Brossas J.-Y.; Pinault E.; Bourgeade-Delmas S.; Sournia-Saquet A.; Boutet-Robinet E.; Destere A.; Tronnet A.; Bergé J.; Bonduelle C.; Deraeve C.; Pratviel G.; Stigliani J.-L.; Paris L.; Mazier D.; Corvaisier S.; Since M.; Malzert-Fréon A.; Wyllie S.; Milne R.; Fairlamb A. H.; Valentin A.; Courtioux B.; Verhaeghe P. New 8-Nitroquinolinone Derivative Displaying Submicromolar in Vitro Activities against Both Trypanosoma Brucei and Cruzi. ACS Med. Chem. Lett. 2020, 11, 464–472. 10.1021/acsmedchemlett.9b00566. 32292551 PMC7153024

[ref40] Singh B.; Bernatchez J. A.; McCall L. I.; Calvet C. M.; Ackermann J.; Souza J. M.; Thomas D.; Silva E. M.; Bachovchin K. A.; Klug D. M.; Jalani H. B.; Bag S.; Buskes M. J.; Leed S. E.; Roncal N. E.; Penn E. C.; Erath J.; Rodriguez A.; Sciotti R. J.; Campbell R. F.; McKerrow J.; Siqueira-Neto J. L.; Ferrins L.; Pollastri M. P. Scaffold and Parasite Hopping: Discovery of New Protozoal Proliferation Inhibitors. ACS Med. Chem. Lett. 2020, 11, 249–257. 10.1021/acsmedchemlett.9b00453. 32184953 PMC7073875

[ref41] Saccoliti F.; Madia V. N.; Tudino V.; De Leo A.; Pescatori L.; Messore A.; De Vita D.; Scipione L.; Brun R.; Kaiser M.; Mäser P.; Calvet C. M.; Jennings G. K.; Podust L. M.; Pepe G.; Cirilli R.; Faggi C.; Di Marco A.; Battista M. R.; Summa V.; Costi R.; Di Santo R. Design, Synthesis, and Biological Evaluation of New 1-(Aryl-1 H-Pyrrolyl)(Phenyl)Methyl-1 H-Imidazole Derivatives as Antiprotozoal Agents. J. Med. Chem. 2019, 62, 1330–1347. 10.1021/acs.jmedchem.8b01464. 30615444

[ref42] Bachovchin K. A.; Sharma A.; Bag S.; Klug D. M.; Schneider K. M.; Singh B.; Jalani H. B.; Buskes M. J.; Mehta N.; Tanghe S.; Momper J. D.; Sciotti R. J.; Rodriguez A.; Mensa-Wilmot K.; Pollastri M. P.; Ferrins L. Improvement of Aqueous Solubility of Lapatinib-Derived Analogues: Identification of a Quinolinimine Lead for Human African Trypanosomiasis Drug Development. J. Med. Chem. 2019, 62, 665–687. 10.1021/acs.jmedchem.8b01365. 30565932 PMC6556231

[ref43] Woodring J. L.; Bachovchin K. A.; Brady K. G.; Gallerstein M. F.; Erath J.; Tanghe S.; Leed S. E.; Rodriguez A.; Mensa-Wilmot K.; Sciotti R. J.; Pollastri M. P. Optimization of Physicochemical Properties for 4-Anilinoquinazoline Inhibitors of Trypanosome Proliferation. Eur. J. Med. Chem. 2017, 141, 446–459. 10.1016/j.ejmech.2017.10.007. 29049963 PMC5682201

[ref44] Woodland A.; Thompson S.; Cleghorn L. A. T.; Norcross N.; De Rycker M.; Grimaldi R.; Hallyburton I.; Rao B.; Norval S.; Stojanovski L.; Brun R.; Kaiser M.; Frearson J. A.; Gray D. W.; Wyatt P. G.; Read K. D.; Gilbert I. H. Discovery of Inhibitors of Trypanosoma Brucei by Phenotypic Screening of a Focused Protein Kinase Library. ChemMedChem 2015, 10, 1809–1820. 10.1002/cmdc.201500300. 26381210 PMC4648050

[ref45] Lipinski C. A.; Lombardo F.; Dominy B. W.; Feeney P. J. Experimental and Computational Approaches to Estimate Solubility and Permeability in Drug Discovery and Development Settings. Adv. Drug Deliv. Rev. 1997, 23, 3–25. 10.1016/s0169-409x(96)00423-1. 11259830

[ref46] Baell J. B.; Holloway G. A. New Substructure Filters for Removal of Pan Assay Interference Compounds (PAINS) from Screening Libraries and for Their Exclusion in Bioassays. J. Med. Chem. 2010, 53, 2719–2740. 10.1021/jm901137j. 20131845

[ref47] Xie X.-Q. PAINS Remover. https://www.cbligand.org/PAINS/ (accessed 01 24, 2023).

[ref48] Moraes C. B.; Witt G.; Kuzikov M.; Ellinger B.; Calogeropoulou T.; Prousis K. C.; Mangani S.; Di Pisa F.; Landi G.; Iacono L. D.; Pozzi C.; Freitas-Junior L. H.; dos Santos Pascoalino B.; Bertolacini C. P.; Behrens B.; Keminer O.; Leu J.; Wolf M.; Reinshagen J.; Cordeiro-da-Silva A.; Santarem N.; Venturelli A.; Wrigley S.; Karunakaran D.; Kebede B.; Pöhner I.; Müller W.; Panecka-Hofman J.; Wade R. C.; Fenske M.; Clos J.; Alunda J. M.; Corral M. J.; Uliassi E.; Bolognesi M. L.; Linciano P.; Quotadamo A.; Ferrari S.; Santucci M.; Borsari C.; Costi M. P.; Gul S. Accelerating Drug Discovery Efforts for Trypanosomatidic Infections Using an Integrated Transnational Academic Drug Discovery Platform. SLAS Discovery 2019, 24, 346–361. 10.1177/2472555218823171. 30784368 PMC6484532

[ref49] Genetic Toxicology Screening Assays at Wuxi. https://labtesting.wuxiapptec.com/safety-assessment-services/genetic-in-vitro-toxicology/ (accessed 12 06, 2022).

[ref50] Safety47 Panel Dose Response. SAFETYscan Eurofins. https://www.eurofinsdiscoveryservices.com/catalogmanagement/viewItem/Safety47-Panel-Dose-Response-SAFETYscan-DiscoverX/87-1003DR (accessed 12 06, 2022).

