# Supplementary material for: Discovery of 5-Phenylpyrazolopyrimidinone Analogs as Potent Antitrypanosomal Agents with In Vivo Efficacy
Source: J Med Chem. 2023 Jul 20;66(15):10252–64. doi: 10.1021/acs.jmedchem.3c00161 (PMC10424178; doi:10.1021/acs.jmedchem.3c00161)
Supplement: Supplementary file 1 — jm3c00161_si_001.pdf [file jm3c00161_si_001.pdf]

Supporting information

Discovery of 5-phenylpyrazolopyrimidinone analogues as potent antitrypanosomal agents with *in vivo* efficacy

Yang Zheng[a,1], Magali van den Kerkhof[b,1], Tiffany van der Meer[a], Sheraz Gul[c,d], Maria Kuzikov[c,d], Bernhard Ellinger[c,d], Iwan J.P. de Esch[a], Marco Siderius[a], An Matheeussen[b], Louis Maes[b], Geert Jan Sterk[a], Guy Caljon[b,1]* and Rob Leurs[a,1]*

[a] Amsterdam Institute for Molecules, Medicines and Systems, Division of Medicinal Chemistry, Faculty of Science, Vrije Universiteit Amsterdam, De Boelelaan 1108, 1081 HZ Amsterdam, the Netherlands.

[b] Laboratory of Microbiology, Parasitology and Hygiene (LMPH), University of Antwerp, Universiteitsplein 1, 2610 Wilrijk, Belgium.

[c] Fraunhofer Institute for Translational Medicine and Pharmacology ITMP, 22525 Hamburg, Germany.

[d] Fraunhofer Cluster of Excellence for Immune-Mediated Diseases CIMD, 22525 Hamburg, Germany

[1] Authors contributed equally to this manuscript.

* Corresponding authors: guy.caljon@uantwerpen.be (Guy Caljon); r.leurs@vu.nl (Rob Leurs).

Table of contents

Figure S1. Structures of approved HAT treatments.	Page S2
Table S1. Physicochemical properties of **1** (BIPPO).	Page S2
Table S2. Phenotypic activity of close BIPPO analogues against *T. b. brucei, T. cruzi* and *L. infantum.*	Page S3
Figure S2. Antitrypanosomal potency of **23**, **30** and **31**.	Page S4
Figure S3. Anti-*Tbr*PDEB1 activity *of* **30**.	Page S4
Table S2. Safety profile of analogue **30** (NPD-2975) from *Eurofins*.	Page S5
Table S3. *In vitro* metabolic stability of **30** using mouse, rat and human S9 microsomal fractions.	Page S6
Table S4. Chemical characterization of final compounds.	Page S7
Table S5. Antitrypanosomal potency of final compounds.	Page S8-9
Figure S4-S142. LCMS, ^{1}H NMR, ^{13}C NMR and 2D NMR spectroscopy data.	Page S10-S100

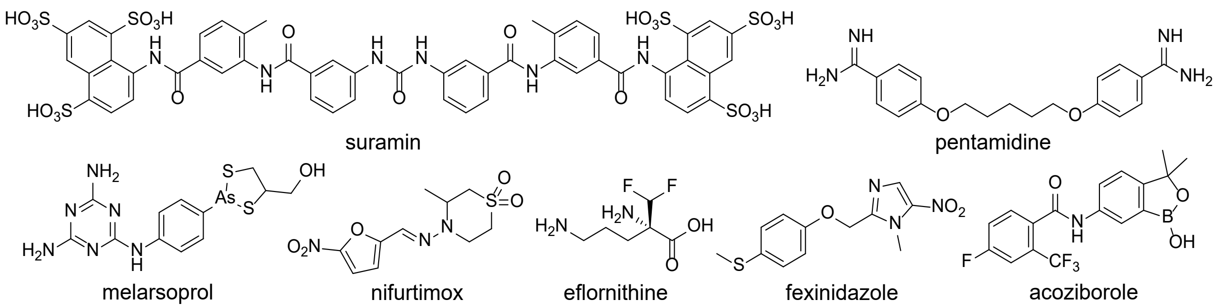

Figure S1. Structures of approved HAT treatments.

Table S1. Physicochemical properties of **1** (BIPPO).

Compound No.	**1** (BIPPO)
PPB-Mouse	88.6%
PPB-Human	88.3%
Solubility (mg/L) @pH 2.2	51
Solubility (mg/L) @pH 4.5	50
Solubility (mg/L) @pH 6.8	47
$t_{0.5}$-Mouse (min)	29
Clint (microL/min/mg protein)	48
$t_{0.5}$-Human (min)	>130
Clint (microL/min/mg protein)	<5.3

Table S2. Phenotypic activity of close BIPPO analogues against *T. b. brucei, T. cruzi* and *L. infantum.*

Code	R^1	*T. b. brucei* $pIC_{50}{}^a$	*T. cruzi* $pIC_{50}{}^a$	*L. infantum* $pIC_{50}{}^a$	MRC-5 $pIC_{50}{}^a$
**1** (BIPPO)	Bn	4.5 ± 0.2	< 4.2	< 4.2	< 4.2
**9** (NPD-2960)	4-PyCH$_2$	< 4.2	< 4.2	< 4.2	< 4.2
**10** (NPD-0434)	$C_6H_5OCH_2$	< 4.2	< 4.2	< 4.2	< 4.2
**11** (NPD-3281)	$C_6H_5(CH_2)_2$	< 4.2	< 4.2	< 4.2	< 4.2
**12** (NPD-3380)	Me	< 4.2	< 4.2	< 4.2	< 4.2
**13** (NPD-3645)	^{n}Bu	5.0 ± 0.0	< 4.2	< 4.2	< 4.2
**14** (NPD-3379)	^{i}Pr	4.4 ± 0.1	< 4.2	< 4.2	< 4.2
**15** (NPD-3200)	Ph	6.6 ± 0.1	< 4.2	< 4.2	< 4.2
**16** (NPD-3488)	4-Py	5.7 ± 0.0	< 4.2	< 4.2	< 4.2
**17** (NPD-2973)	4-thiazole	4.9 ± 0.0	< 4.2	< 4.2	< 4.2

[a] Mean values of at least two independent experiments.

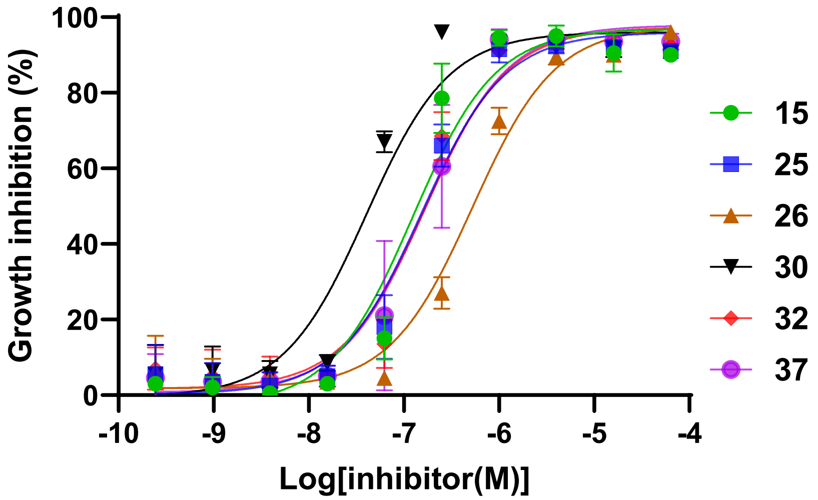

Figure S2. Antitrypanosomal potency of **15**, **25**, **26**, **30** **32** and **37**. Representative drug susceptibility curves (mean ± standard deviation) of **15** (green), **25** (blue), **26** (brown), **30** (black), **32** (red) and **37** (purple) against *T. brucei* with each biological replicate comprised of at least three technical replicates.

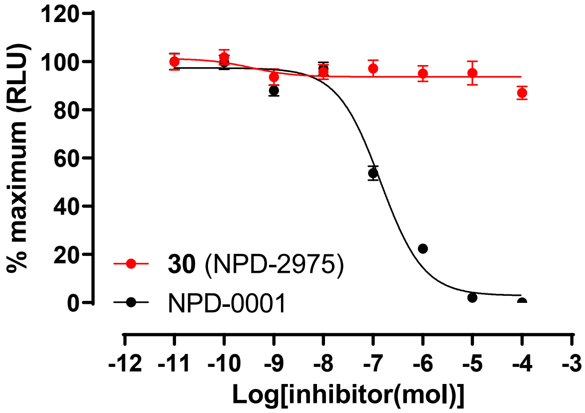

Figure S3. Anti-*Tbr*PDEB1 activity *of* **30**. Representative dose-response curves (mean ± standard error of the mean) of **30** (red) for inhibition of the enzymatic activity of *Tbr*PDEB1 catalytic domain with **NPD-0001** (black) as a reference compound.

Table S3. Safety profile of analogue **30** (NPD-2975) from *Eurofins*.

DiscoverX Gene Symbol	Assay Mode	NPD-2975 10 µM % Response		
GPCRs		Replicate 1	Replicate 2	Average
ADORA2A	Agonist	0.0	1.7	0.9
ADORA2A	Antagonist	15.7	19.2	17.4
ADRA1A	Agonist	1.2	1.6	1.4
ADRA1A	Antagonist	0.1	0.0	0.1
ADRA2A	Agonist	0.0	0.0	0.0
ADRA2A	Antagonist	27.1	32.8	30.0
ADRB1	Agonist	0.3	1.1	0.7
ADRB1	Antagonist	0.0	0.0	0.0
ADRB2	Agonist	0.0	0.0	0.0
ADRB2	Antagonist	0.0	0.0	0.0
AVPR1A	Agonist	0.0	0.0	0.0
AVPR1A	Antagonist	0.0	0.0	0.0
CCKAR	Agonist	0.4	0.0	0.2
CCKAR	Antagonist	21.3	33.4	27.4
CHRM1	Agonist	0.0	0.0	0.0
CHRM1	Antagonist	1.5	0.0	0.7
CHRM2	Agonist	0.0	0.0	0.0
CHRM2	Antagonist	32.4	24.9	28.7
CHRM3	Agonist	0.5	1.8	1.2
CHRM3	Antagonist	15.1	6.7	10.9
CNR1	Agonist	0.0	0.0	0.0
CNR1	Antagonist	8.9	12.4	10.7
CNR2	Agonist	2.0	10.0	6.0
CNR2	Antagonist	4.1	3.8	4.0
DRD1	Agonist	0.0	0.0	0.0
DRD1	Antagonist	3.5	2.9	3.2
DRD2S	Agonist	2.5	1.9	2.2
DRD2S	Antagonist	1.5	3.0	2.3
EDNRA	Agonist	2.0	1.9	2.0
EDNRA	Antagonist	38.5	20.0	29.3
HRH1	Agonist	0.7	2.7	1.7
HRH1	Antagonist	0.0	6.6	3.3
HRH2	Agonist	1.2	0.0	0.6
HRH2	Antagonist	0.0	0.0	0.0
HTR1A	Agonist	0.0	0.0	0.0
HTR1A	Antagonist	14.8	28.1	21.5
HTR1B	Agonist	0.0	0.0	0.0
HTR1B	Antagonist	9.3	9.6	9.5
HTR2A	Agonist	0.0	0.0	0.0
HTR2A	Antagonist	12.3	10.7	11.5
HTR2B	Agonist	0.0	0.0	0.0
HTR2B	Antagonist	5.2	21.0	13.1
OPRD1	Agonist	0.0	0.0	0.0
OPRD1	Antagonist	13.8	18.5	16.2
OPRK1	Agonist	0.0	0.0	0.0
OPRK1	Antagonist	14.8	14.3	14.6
OPRM1	Agonist	0.0	0.0	0.0
OPRM1	Antagonist	26.4	33.8	30.1

DiscoverX Gene Symbol	Assay Mode	NPD-2975 10 µM % Response		
Nuclear Hormone Receptors		Replicate 1	Replicate 2	Average
AR	Agonist	0.0	0.0	0.0
AR	Antagonist	11.7	0.0	5.8
GR	Agonist	0.0	0.0	0.0
GR	Antagonist	0.0	0.0	0.0
Transporters		Replicate 1	Replicate 2	Average
DAT	Blocker	17.2	12.2	14.7
NET	Blocker	0.0	0.0	0.0
SERT	Blocker	0.0	0.0	0.0
Ion Channels		Replicate 1	Replicate 2	Average
CAV1.2	Blocker	0.0	0.0	0.0
GABAA	Opener	2.3	4.3	3.3
GABAA	Blocker	0.0	6.3	3.1
hERG	Blocker	0.0	60.9	30.5
HTR3A	Opener	0.0	0.0	0.0
HTR3A	Blocker	7.4	9.1	8.3
KvLQT1/minK	Opener	2.5	0.7	1.6
KvLQT1/minK	Blocker	3.4	1.3	2.3
nAChR(a4/b2)	Opener	1.9	0.0	0.9
nAChR(a4/b2)	Blocker	40.5	19.7	30.1
NAV1.5	Blocker	0.0	4.6	2.3
NMDAR (1A/2B)	Opener	7.0	0.0	3.5
NMDAR (1A/2B)	Blocker	5.2	0.0	2.6
Non-Kinase Enzymes		Replicate 1	Replicate 2	Average
AChE	Inhibitor	0.0	0.0	0.0
COX1	Inhibitor	8.5	17.0	12.7
COX2	Inhibitor	0.0	0.0	0.0
MAOA	Inhibitor	12.6	0.0	6.3
PDE3A	Inhibitor	0.0	0.0	0.0
PDE4D2	Inhibitor	79.2	71.7	75.5
Kinases		Replicate 1	Replicate 2	Average
INSR	Inhibitor	0.0	0.5	0.3
LCK	Inhibitor	35.4	46.0	40.7
ROCK1	Inhibitor	7.7	7.5	7.6
VEGFR2	Inhibitor	0.0	0.0	0.0

Table S4. *In vitro* metabolic stability of **30** using mouse, rat and human S9 microsomal fractions.

Microsomes	Phase-I/II	Time (min)	**30**[a]	**Diclofenac**[a]
Mouse	CYP450 - NADPH	0	100	100
		15	73 ± 2.8	85 ± 0.0
		30	55 ± 1.8	62 ± 7.1
		60	33 ± 3.4	49 ± 2.4
			(n=2)	(n=2)
	UGT enzymes	0	100	100
		15	93 ± 4.2	56 ± 16
		30	81 ± 2.7	46 ± 10
		60	71 ± 5.4	39 ± 0.0
			(n=2)	(n=2)
Rat	CYP450 - NADPH	0	100	100
		15	93 ± 10.2	73 ± 15.1
		30	92 ± 2.4	57 ± 24.0
		60	78 ± 0.2	34 ± 36.5
			(n=2)	(n=3)
	UGT enzymes	0	100	100
		15	84 ± 4.9	42 ± 21.8
		30	87 ± 3.6	27 ± 23.2
		60	76 ± 7.1	22 ± 17.9
			(n=2)	(n=3)
Human	CYP450 - NADPH	0	100	100
		15	102 ± 5.3	43 ± 3.5
		30	99 ± 3.1	14 ± 1.4
		60	92 ± 3.1	3 ± 0.0
			(n=3)	(n=2)
	UGT enzymes	0	100	100
		15	81 ± 1.1	21 ± 0.7
		30	79 ± 2.9	14 ± 1.4
		60	75 ± 2.2	11 ± 0.7
			(n=4)	(n=2)

[a] Results are based on at least two repeats and are expressed as mean percentage remaining **30** ± standard error of mean (SEM).

Table S5. Chemical characterization of final compounds.

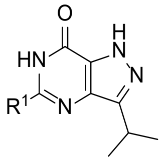

Code	R^1	Formula	Yields from **8**	LCMS retention time (min)	LCMS purity at 254 nm	HR-MS $[M+H]^+$	
						Calculated	Found
**1**	Bn	$C_{15}H_{16}N_4O$	68%	3.66	>99%	269.1397	269.1385
**9**	4-PyCH_2	$C_{14}H_{15}N_5O$	59%	2.26	98%	270.1349	270.1341
**10**	$C_6H_5OCH_2$	$C_{15}H_{16}N_4O_2$	60%	3.80	>99%	285.1346	285.1341
**11**	$C_6H_5(CH_2)_2$	$C_{16}H_{18}N_4O$	71%	3.87	>99%	283.1553	283.1545
**12**	Me	$C_9H_{12}N_4O$	90%	2.46	>99%	193.1084	193.1090
**13**	^{n}Bu	$C_{12}H_{18}N_4O$	42%	3.58	>99%	235.1553	235.1562
**14**	^{i}Pr	$C_{11}H_{16}N_4O$	87%	3.42	>99%	221.1397	221.1405
**15**	Ph	$C_{14}H_{14}N_4O$	32%	3.78	>99%	277.1060[a]	277.1070[a]
**16**	4-Py	$C_{13}H_{13}N_5O$	33%	2.63	>99%	256.1193	256.1186
**17**	4-thiazole	$C_{11}H_{11}N_5OS$	54%	3.40	>99%	262.0757	262.0756
**18**	2-F-Ph	$C_{14}H_{13}FN_4O$	33%	3.66	>99%	273.1146	273.1144
**19**	2-Cl-Ph	$C_{14}H_{13}ClN_4O$	56%	3.66	>99%	289.0851	289.0850
**20**	2-Br-Ph	$C_{14}H_{13}BrN_4O$	88%	3.69	>99%	333.0346	333.0333
**21**	2-Me-Ph	$C_{15}H_{16}N_4O$	43%	3.79	>99%	269.1397	269.1405
**22**	2-OMe-Ph	$C_{15}H_{16}N_4O_2$	5%	3.96	>99%	285.1346	285.1333
**23**	3-F-Ph	$C_{14}H_{13}FN_4O$	37%	3.98	98%	295.0966[a]	295.0954[a]
**24**	3-Cl-Ph	$C_{14}H_{13}ClN_4O$	4%	4.25	99%	311.0670[a]	311.0676[a]
**25**	3-Me-Ph	$C_{15}H_{16}N_4O$	63%	4.10	>99%	269.1397	269.1386
**26**	3-OMe-Ph	$C_{15}H_{16}N_4O_2$	33%	3.87	>99%	285.1346	285.1339
**27**	3-OH-Ph	$C_{14}H_{14}N_4O_2$	41%	3.23	>99%	271.1190	271.1185
**28**	3-$N(CH_3)_2$-Ph	$C_{16}H_{19}N_5O$	45%	3.71	>99%	298.1662	298.1662
**29**	3-SO_2CH_3-Ph	$C_{15}H_{16}N_4O_3S$	24%	3.29	95%	333.1016	333.1012
**30**	4-F-Ph	$C_{14}H_{13}FN_4O$	62%	3.89	>99%	273.1146	273.1144
**31**	4-Cl-Ph	$C_{14}H_{13}ClN_4O$	37%	4.32	98%	289.0851	289.0839
**32**	4-Br-Ph	$C_{14}H_{13}BrN_4O$	54%	4.38	>99%	333.0346	333.0347
**33**	4-OMe-Ph	$C_{15}H_{16}N_4O_2$	58%	3.86	>99%	285.1346	285.1343
**34**	4-O^{i}Pr-Ph	$C_{17}H_{20}N_4O_2$	36%	4.40	>99%	313.1659	313.1651
**35**	4-CF_3-Ph	$C_{15}H_{13}F_3N_4O$	42%	4.43	>99%	345.0934[a]	345.0920[a]
**36**	4-OCF_3-Ph	$C_{15}H_{13}F_3N_4O_2$	15%	4.54	>99%	339.1063	339.1069
**37**	4-CN-Ph	$C_{15}H_{13}N_5O$	37%	3.69	>99%	280.1193	280.1182
**38**	4-COOMe-Ph	$C_{16}H_{16}N_4O_3$	34%	3.83	97%	313.1295	313.1284
**39**	4-COOH-Ph	$C_{15}H_{14}N_4O_3$	26%	3.16	99%	299.1139	299.1101
**40**	4-$CONH_2$-Ph	$C_{15}H_{15}N_5O_2$	18%	2.76	97%	320.1118[a]	320.1105[a]
**41**	4-SO_2Me-Ph	$C_{15}H_{16}N_4O_3S$	30%	3.27	97%	333.1016	333.1011
**42**	4-SO_2NH_2-Ph	$C_{14}H_{15}N_5O_3S$	19%	2.96	>99%	334.0969	334.0953
**43**	4-$NHCOCH_3$-Ph	$C_{16}H_{17}N_5O_2$	47%	3.06	>99%	312.1455	312.1443
**44**	4-(*N*-piperidine)-Ph	$C_{19}H_{23}N_5O$	23%	4.34	>99%	338.1975	338.1964
**45**	4-(*N*-methylpipera-zine)-Ph	$C_{19}H_{24}N_6O$	35%	2.53	98%	353.2084	353.2078
**46**	4-tetrazole-Ph	$C_{15}H_{14}N_8O$	26%	3.10	>99%	323.1363	323.1357

[a]: $[M+Na]^+$.

Table S6. Anti-*T.brucei* potency of final compounds.

**Code**	**Screening concentration (µM)**										**IC_{50}**	**IC_{90}**
	64	16	4	1	0.25	0.06	0.016	0.004	0.001	0.0002		
	**Growth inhibition (%)**										**µM**	
**9** (NPD-2960)	9	15	7	11	7						> 64.0	> 64.0
**9** (NPD-2960)	5	8	7	7	0						> 64.0	> 64.0
**10** (NPD-0434)	15	3	1	0	0						> 64.0	> 64.0
**10** (NPD-0434)	7	5	0	0	0						> 64.0	> 64.0
**11** (NPD-3281)	30	12	14	11	15						> 64.0	> 64.0
**11** (NPD-3281)	50	39	9	3	11						64.0	> 64.0
**12** (NPD-3380)	0	0	0	0	0						> 64.0	> 64.0
**12** (NPD-3380)	9	11	2	1	2						> 64.0	> 64.0
**13** (NPD-3645)	75	67	22	6	3	2	8				9.5	> 64.0
**13** (NPD-3645)	72	65	27	0	0	0	0				9.3	> 64.0
**14** (NPD-3379)	66	23	0	0	0						38.2	> 64.0
**14** (NPD-3379)	60	17	8	10	7						46.4	> 64.0
**15** (NPD-3200)	86	84	84	84	50						0.3	N.D.
**15** (NPD-3200)	78	80	79	76	39						0.4	N.D.
**15** (NPD-3200)	89	87	93	93	72	19	4	0	0	2	0.14	0.82
**15** (NPD-3200)	91	94	97	96	85	11	2	1	4	4	0.13	0.47
**16** (NPD-3488)	85	85	69	27	8						2.1	> 64.0
**16** (NPD-3488)	82	80	75	31	21	9	12				1.8	> 64.0
**17** (NPD-2973)	80	54	10	3	3						14.1	> 64.0
**17** (NPD-2973)	82	58	5	0	0						13.0	> 64.0
**18** (NPD-3199)	85	85	84	58	11						0.8	> 64.0
**18** (NPD-3199)	77	80	79	48	5						1.1	> 64.0
**19** (NPD-3538)	72	71	72	48	6	21	0				1.1	> 64.0
**19** (NPD-3538)	77	78	78	70	28	0	5				0.5	> 64.0
**20** (NPD-3539)	71	70	73	56	18	19	1				0.8	> 64.0
**20** (NPD-3539)	75	77	78	68	26	5	13				0.6	> 64.0
**21** (NPD-3589)	83	90	81	81	55	6	7				0.2	16.0
**21** (NPD-3589)	78	77	79	79	72	28	0				0.1	> 64.0
**22** (NPD-3590)	81	76	34	2	0	7	5				6.8	> 64.0
**22** (NPD-3590)	77	66	29	0	0	0	0				8.8	> 64.0
**23** (NPD-3202)	87	86	85	86	75						< 0.3	> 64.0
**23** (NPD-3202)	81	81	80	80	63						< 0.3	> 64.0
**23** (NPD-3202)	80	79	79	79	74	30	0				0.1	> 64.0
**23** (NPD-3202)	86	85	85	84	80	35	0				0.1	> 64.0
**24** (NPD-3591)	80	81	78	79	50	14	7				0.3	> 64.0
**24** (NPD-3591)	81	79	78	76	70	29	6				0.1	> 64.0
**25** (NPD-3382)	85	84	84	79	37						0.4	N.D.
**25** (NPD-3382)	82	82	82	79	43						0.3	N.D.
**25** (NPD-3382)	90	91	91	89	62	24	7	0	0	0	0.16	2.0
**25** (NPD-3382)	92	93	94	94	70	12	3	5	7	11	0.16	0.79
**26** (NPD-3375)	92	85	82	66	28						0.6	N.D.
**26** (NPD-3375)	91	91	81	62	39						0.5	N.D.
**26** (NPD-3375)	95	89	88	70	24	4	3	4	0	0	0.55	20.16
**26** (NPD-3375)	97	91	91	75	30	5	4	5	8	13	0.46	3.67
**27** (NPD-2974)	80	72	34	6	3						7.2	> 64.0
**27** (NPD-2974)	81	77	37	6	0						6.3	> 64.0
**28** (NPD-3381)	87	72	20	0	0						8.9	> 64.0
**28** (NPD-3381)	84	70	26	0	6						8.5	> 64.0
**29** (NPD-3598)	79	24	4	0	7	0	2				30.8	> 64.0
**30** (NPD-2975)	81	76	76	76	76						< 0.3	N.D.
**30** (NPD-2975)	83	81	84	82	79						< 0.3	N.D.
**30** (NPD-2975)	85	84	84	84	82	45	33				0.1	N.D.

**30** (NPD-2975)	84	85	84	85	82	39	11				0.1	N.D.
**30** (NPD-2975)	90	90	91	93	96	69	10	3	2	0	0.04	0.18
**30** (NPD-2975)	91	93	93	94	96	65	7	8	11	11	0.04	0.19
**31** (NPD-3204)	90	89	87	85	83						< 0.3	64.0
**31** (NPD-3204)	85	85	81	81	71						< 0.3	> 64.0
**31** (NPD-3204)	82	83	81	81	74	39	3				0.1	> 64.0
**31** (NPD-3204)	89	89	87	84	81	36	0				0.1	> 64.0
**32** (NPD-2971)	81	78	76	67	28						0.5	N.D.
**32** (NPD-2971)	85	83	84	78	30						0.4	N.D.
**32** (NPD-2971)	92	92	93	91	73	18	6	3	0	3	0.14	0.93
**32** (NPD-2971)	93	94	94	91	64	9	4	9	10	11	0.18	0.95
**33** (NPD-2972)	83	79	74	52	10						0.9	> 64.0
**33** (NPD-2972)	85	83	82	59	18						0.7	> 64.0
**34** (NPD-3377)	47	42	34	2	0						> 64.0	> 64.0
**34** (NPD-3377)	61	52	34	10	11						13.7	> 64.0
**35** (NPD-3201)	93	87	84	46	10						1.2	32.0
**35** (NPD-3201)	91	83	79	42	0						1.3	53.8
**36** (NPD-3597)	95	83	70	42	6	2	2				1.5	35.9
**36** (NPD-3597)	81	78	73	52	32	6	8				0.9	> 64.0
**37** (NPD-3203)	89	88	86	84	46						0.3	N.D.
**37** (NPD-3203)	83	81	75	72	33						0.5	N.D.
**37** (NPD-3203)	95	94	94	96	72	35	7	3	0	0	0.11	0.71
**37** (NPD-3203)	92	93	94	92	49	7	3	4	7	9	0.26	0.94
**38** (NPD-3305)	48	9	6	5	2						> 64.0	> 64.0
**38** (NPD-3305)	43	10	2	5	0						> 64.0	> 64.0
**39** (NPD-3489)	4	3	0	0	0						> 64.0	> 64.0
**40** (NPD-3371)	5	2	0	0	0						> 64.0	> 64.0
**40** (NPD-3371)	4	7	0	5	5						> 64.0	> 64.0
**41** (NPD-3376)	52	22	1	0	0						58.4	> 64.0
**41** (NPD-3376)	62	16	15	5	14						44.6	> 64.0
**42** (NPD-3372)	70	26	5	0	0						34.1	> 64.0
**42** (NPD-3372)	64	20	6	6	0						41.2	> 64.0
**43** (NPD-3280)	12	11	11	12	15						> 64.0	> 64.0
**43** (NPD-3280)	13	26	13	14	4						> 64.0	> 64.0
**44** (NPD-3283)	38	35	29	20	20						> 64.0	> 64.0
**44** (NPD-3283)	83	48	28	32	8						17.3	> 64.0
**45** (NPD-3282)	50	26	13	10	15						64.0	> 64.0
**45** (NPD-3282)	97	87	11	12	7						8.1	24.3
**46** (NPD-3490)	23	16	1	3	3						> 64.0	> 64.0

N.D.: not determined.

Acquired by	: Admin
Date Acquired	: 29/11/2017 2:59:24 PM
Sample Name	: DOSC-Y
Sample ID	:
Tray#	: 1
Vial#	: 18
Injection Volume	: 5
Data File	: C:\LabSolutions\Data\2017\2017-wk48\DOSC-Y.lcd
Background File	: blanco 29112017.lcd
Method File	: Method SCAN ACID standard neg.lcm
Report Format	: DefaultLCMS.lcr
Tuning File	: C:\LabSolutions\Tuning\Tuning-ESI-pos-neg01072015a.lct
Processed by	: Admin
Modified Date	: 29/11/2017 4:45:31 PM

Chromatogram
DOSC-Y C:\LabSolutions\Data\2017\2017-wk48\DOSC-Y.lcd

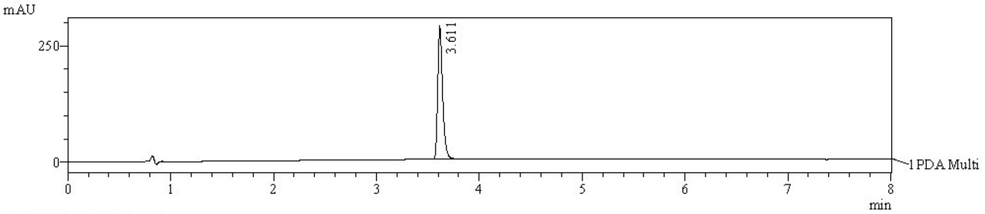

1 PDA Multi 1 / 254nm 4nm
PDA Ch1 254nm 4nm

Peak#	Name	Ret. Time	Area	Area%
1		3.611	935139	100.000

MS Chromatogram
DOSC-Y C:\LabSolutions\Data\2017\2017-wk48\DOSC-Y.lcd

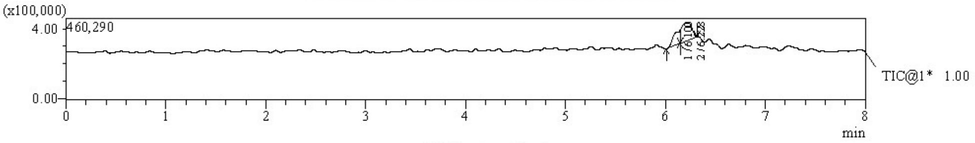

MS Spectrum Graph

MS Spectrum Table

Figure S4. LCMS spectrum of intermediate **4**.

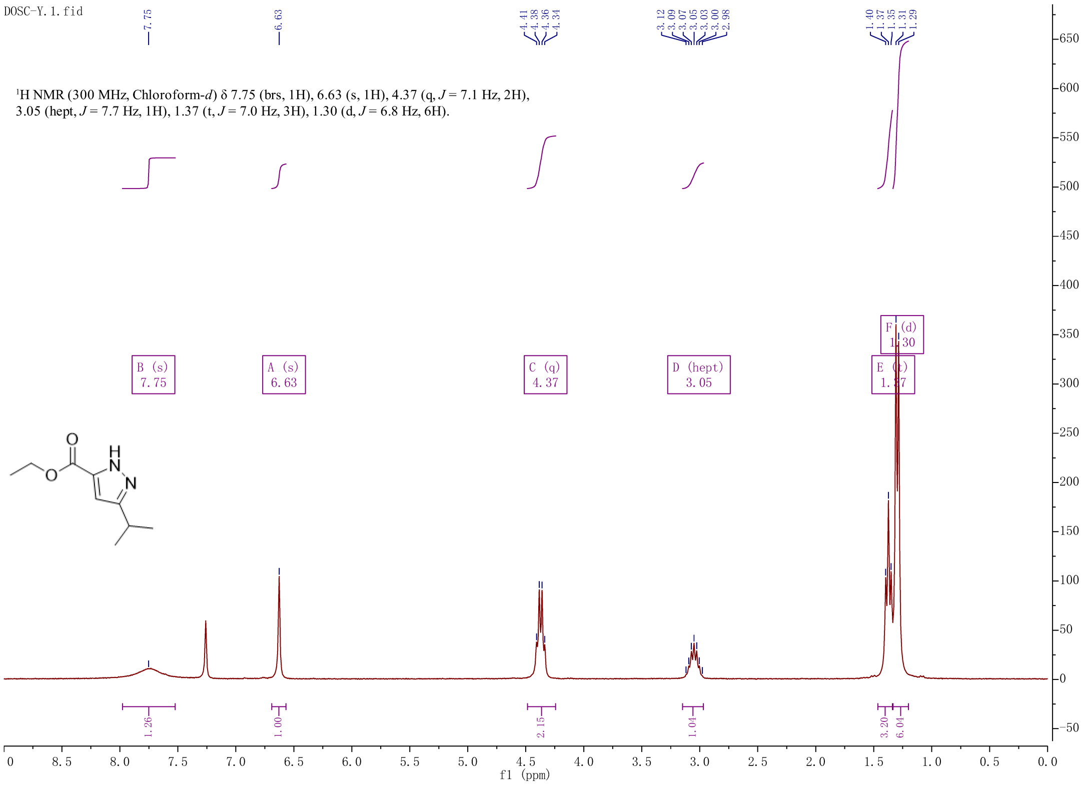

Figure S5. ^{1}H NMR spectrum of intermediate **4**.

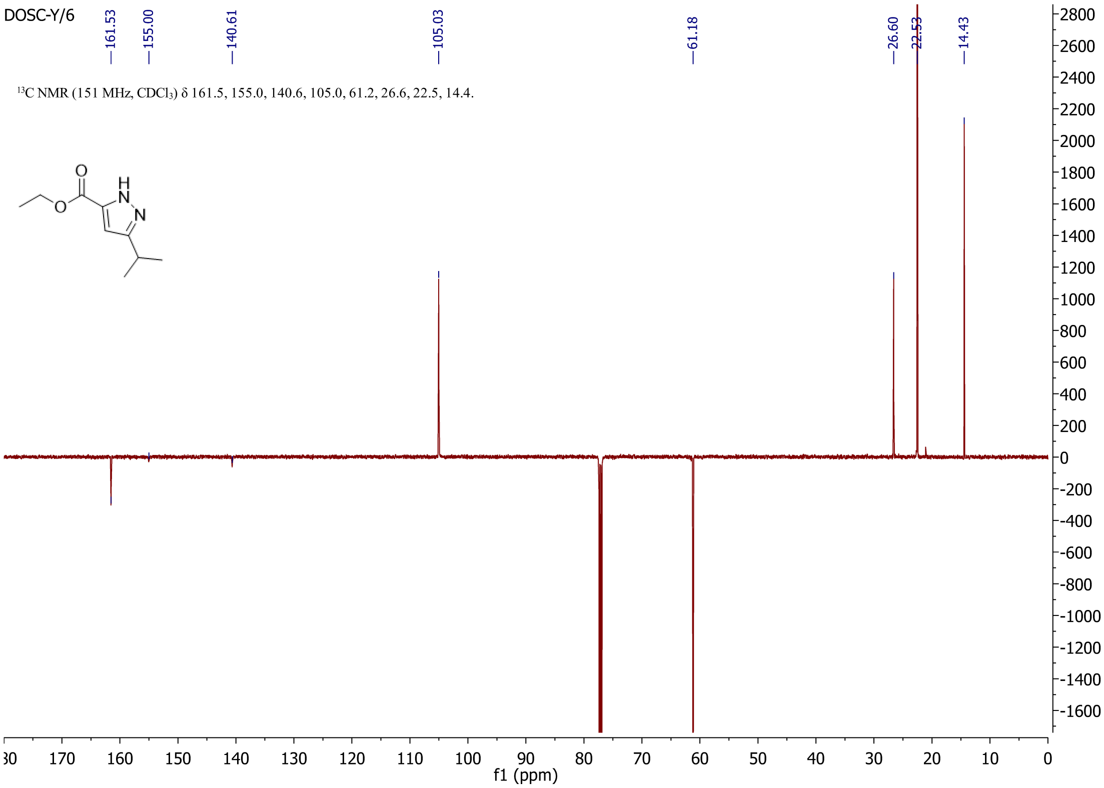

Figure S6. ^{13}C NMR spectrum of intermediate **4**.

Acquired by	: Admin
Date Acquired	: 30/11/2017 4:35:45 PM
Sample Name	: YAZH01-174
Sample ID	:
Tray#	: 1
Vial#	: 19
Injection Volume	: 3
Data File	: C:\LabSolutions\Data\2017\2017-wk48\YAZH01-174.lcd
Background File	: blanco 30112017.lcd
Method File	: Method SCAN ACID standard neg.lcm
Report Format	: DefaultLCMS.lcr
Tuning File	: C:\LabSolutions\Tuning\Tuning-ESI-pos-neg01072015a.lct
Processed by	: Admin
Modified Date	: 30/11/2017 5:16:55 PM

Chromatogram
YAZH01-174 C:\LabSolutions\Data\2017\2017-wk48\YAZH01-174.lcd

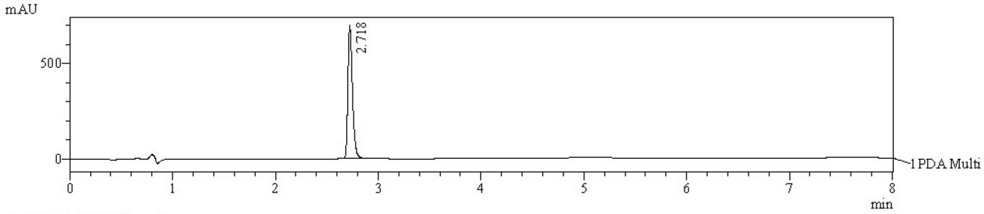

1 PDA Multi 1 / 230nm 4nm
PDA Ch1 230nm 4nm

Peak#	Name	Ret. Time	Area	Area%
1		2.718	2128372	100.000

MS Chromatogram
YAZH01-174 C:\LabSolutions\Data\2017\2017-wk48\YAZH01-174.lcd

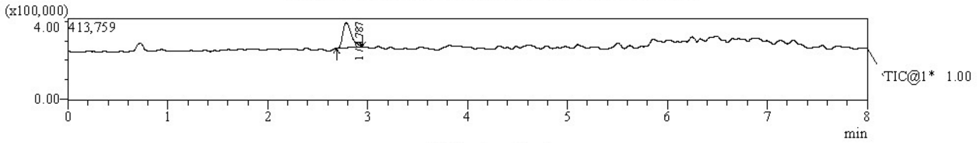

MS Spectrum Graph
#1 Ret.Time:Averaged 2.780-2.800(Scan#:279-281)
Mass Peaks:48 Base Peak:307.15(33293) Polarity:Neg Segment1 - Event1

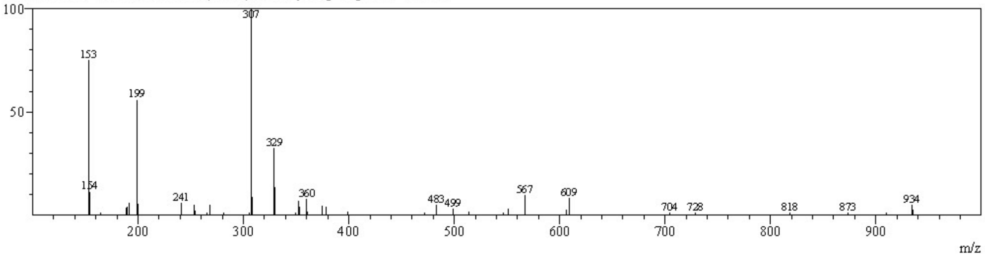

MS Spectrum Table
#:1 Ret.Time:
BG Mode:Calc 2.690<->2.950(270<->296)
Mass Peaks:48 Base Peak:307.15(33293) Polarity:Neg Segment1 - Event1

#	m/z	Abs.Inten.	Rel.Inten.	Charge	Polarity	Monoisotopic
1	152.95	24991	75.06			
2	154.00	3728	11.20			
3	164.20	371	1.11			
4	188.95	1070	3.21			
5	189.95	1269	3.81			
6	191.15	1966	5.91			
7	191.95	356	1.07			
8	198.90	18560	55.75			
9	200.15	1817	5.46			
10	241.10	1926	5.78			
11	252.85	1604	4.82			
12	253.85	698	2.10			
13	264.85	333	1.00			
14	268.10	1599	4.80			
15	280.80	395	1.19			
16	305.80	408	1.23			
17	307.15	33293	100.00			
18	308.30	2856	8.58			

Figure S7. LCMS spectrum of intermediate **5**.

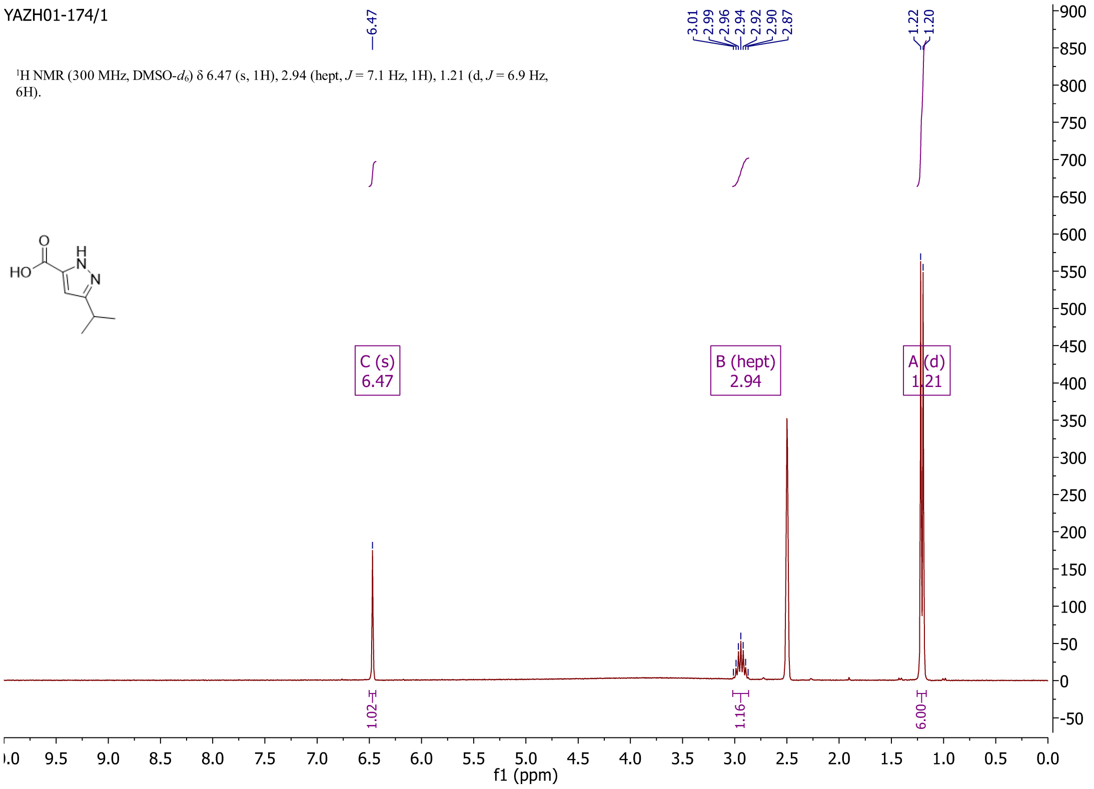

Figure S8. ^{1}H NMR spectrum of compound **5**.

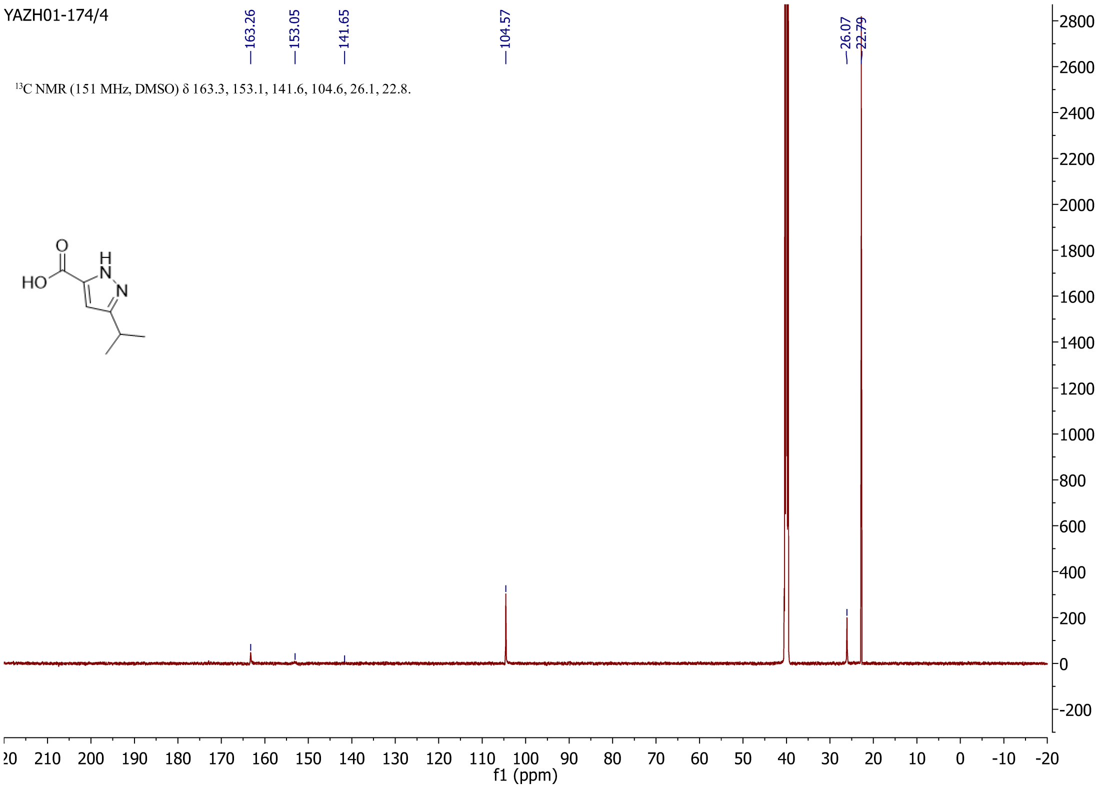

Figure S9. ^{13}C NMR spectrum of compound **5**.

Acquired by	: Admin
Date Acquired	: 8/12/2017 10:01:17 AM
Sample Name	: YAZH01-175
Sample ID	:
Tray#	: 1
Vial#	: 2
Injection Volume	: 1
Data File	: C:\LabSolutions\Data\2017\2017-wk49\YAZH01-175.lcd
Background File	: blanco 08122017.lcd
Method File	: Method SCAN ACID standard neg.lcm
Report Format	: DefaultLCMS.lcr
Tuning File	: C:\LabSolutions\Tuning\Tuning-ESI-pos-neg01072015a.lct
Processed by	: Admin
Modified Date	: 8/12/2017 10:19:37 AM

Chromatogram
YAZH01-175 C:\LabSolutions\Data\2017\2017-wk49\YAZH01-175.lcd

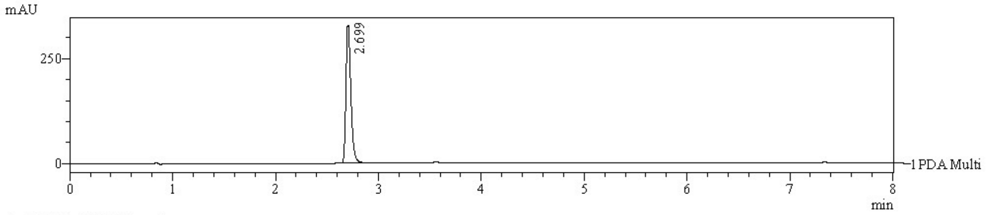

1 PDA Multi 1 / 254nm 4nm
PDA Ch1 254nm 4nm

Peak#	Name	Ret. Time	Area	Area%
1		2.699	1061032	100.000

MS Chromatogram
YAZH01-175 C:\LabSolutions\Data\2017\2017-wk49\YAZH01-175.lcd

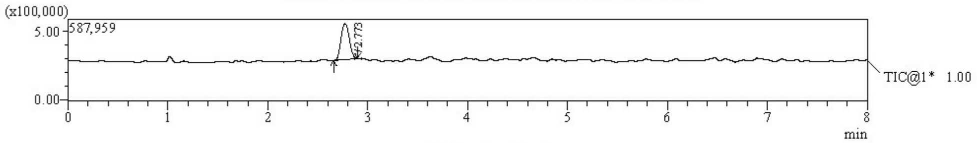

MS Spectrum Graph
#1 Ret.Time:Averaged 2.760-2.780(Scan#:277-279)
Mass Peaks:23 Base Peak:197.95(130385) Polarity:Neg Segment1 - Event1

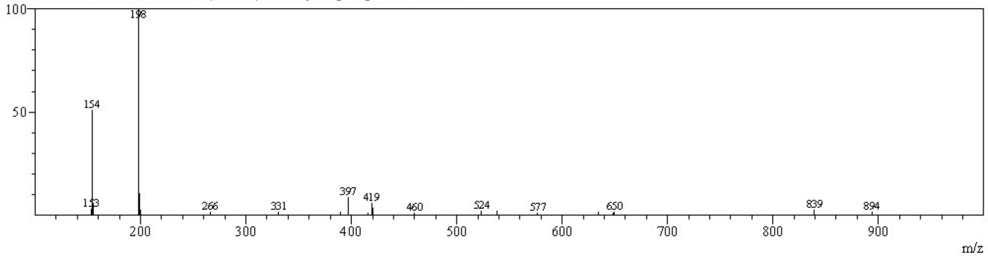

MS Spectrum Table
#:1 Ret.Time:
BG Mode:Calc 2.660<->2.900(267<->291)
Mass Peaks:23 Base Peak:197.95(130385) Polarity:Neg Segment1 - Event1

#	m/z	Abs.Inten.	Rel.Inten.	Charge	Polarity	Monoisotopic	#	m/z	Abs.Inten.	Rel.Inten.	Charge	Polarity	Monoisotopic
1	153.35	3881	2.98				10	397.10	11182	8.58			
2	153.80	66389	50.92				11	415.75	1609	1.23			
3	154.90	6937	5.32				12	419.10	7617	5.84			
4	197.95	130385	100.00				13	420.10	4737	3.63			
5	199.20	13982	10.72				14	460.05	1372	1.05			
6	200.30	3011	2.31				15	523.60	2524	1.94			
7	266.20	2025	1.55				16	537.85	2480	1.90			
8	331.05	1917	1.47				17	576.80	1364	1.05			
9	390.05	1939	1.49				18	634.40	1305	1.00			

Figure S10. LCMS spectrum of compound intermediate **6**.

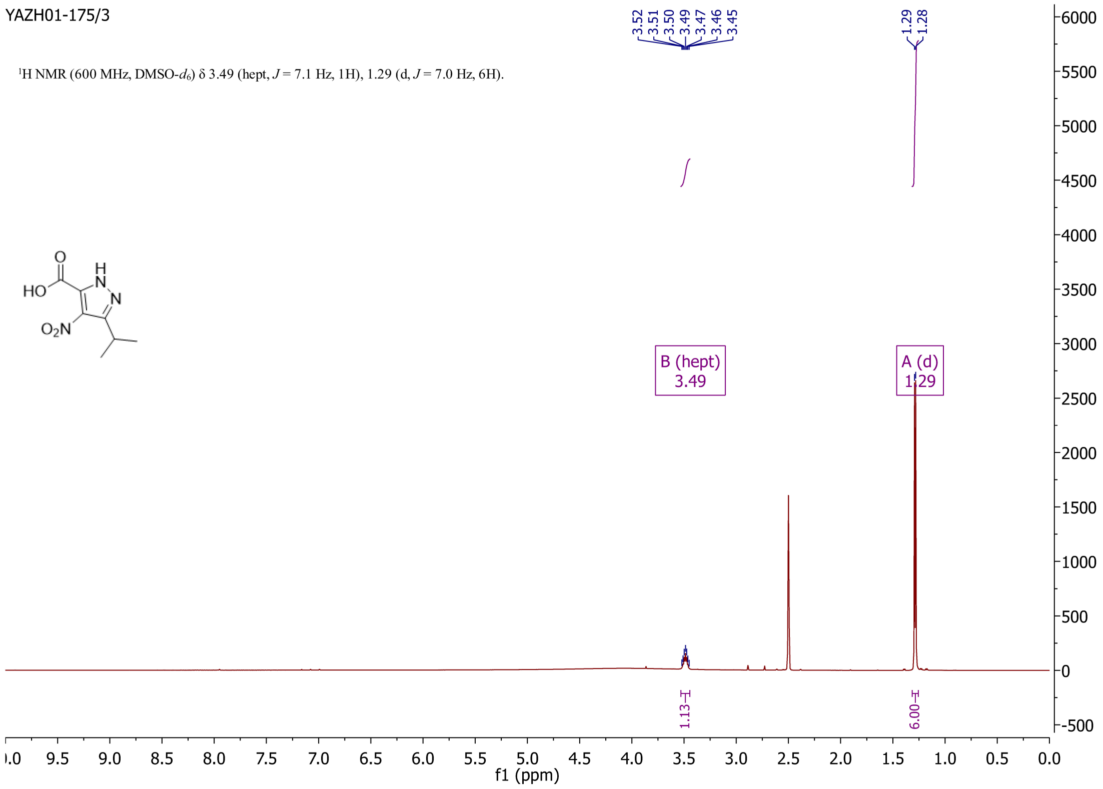

Figure S11. ^{1}H NMR spectrum of compound intermediate **6**.

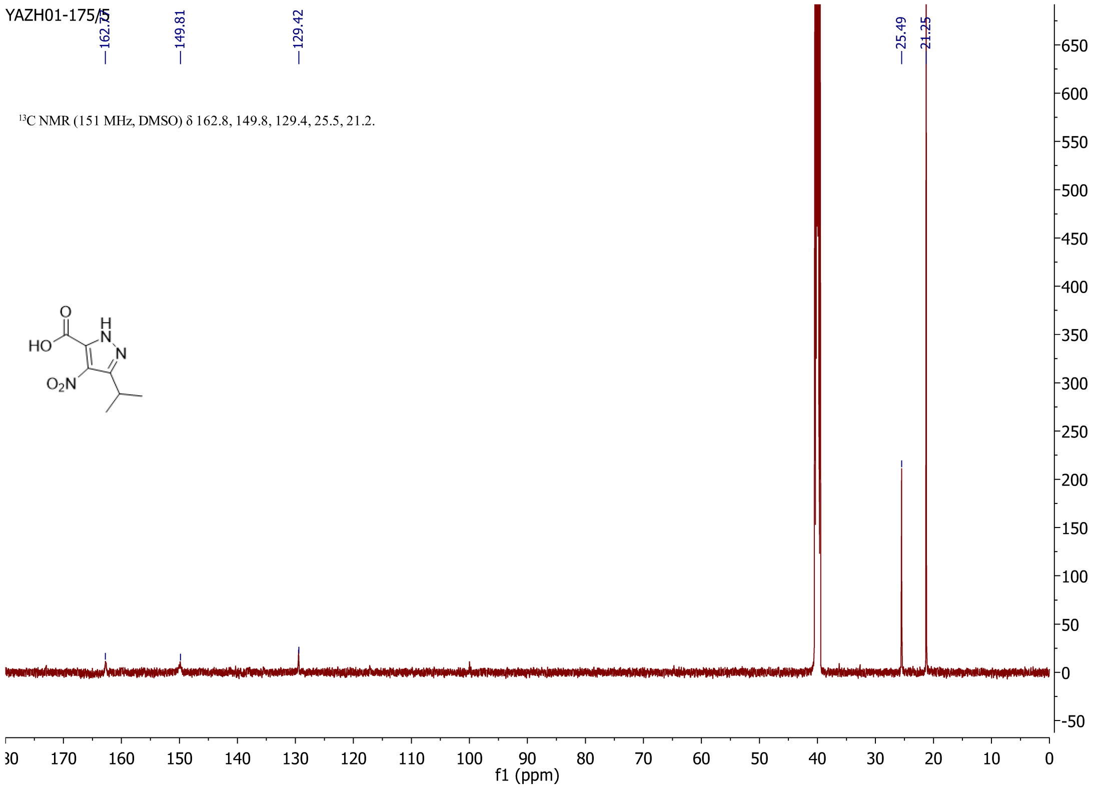

Figure S12. ^{13}C NMR spectrum of compound intermediate **6**.

Acquired by	: Admin
Date Acquired	: 17/1/2018 12:53:30 PM
Sample Name	: YAZH01-178-2
Sample ID	:
Tray#	: 1
Vial#	: 12
Injection Volume	: 3
Data File	: C:\LabSolutions\Data\2018\2018-wk03\YAZH01-178-2.lcd
Background File	: azoblanco_170118.lcd
Method File	: Method SCAN ACID standard MW50.lcm
Report Format	: DefaultLCMS.lcr
Tuning File	: C:\LabSolutions\Tuning\Tuning-ESI-pos-neg01072015a.lct
Processed by	: Admin
Modified Date	: 17/1/2018 1:28:51 PM

Chromatogram
YAZH01-178-2 C:\LabSolutions\Data\2018\2018-wk03\YAZH01-178-2.lcd

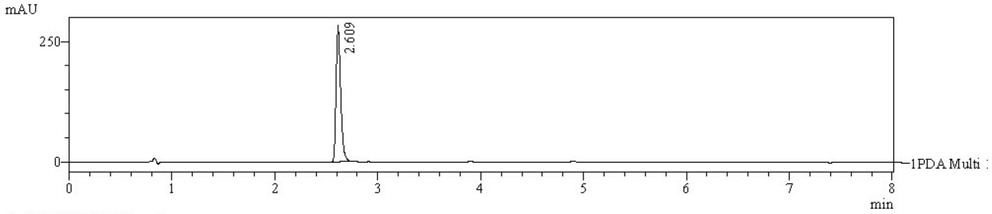

1 PDA Multi 1 / 254nm 4nm
PDA Ch1 254nm 4nm

Peak#	Name	Ret. Time	Area	Area%
1		2.609	874974	100.000

MS Chromatogram
YAZH01-178-2 C:\LabSolutions\Data\2018\2018-wk03\YAZH01-178-2.lcd

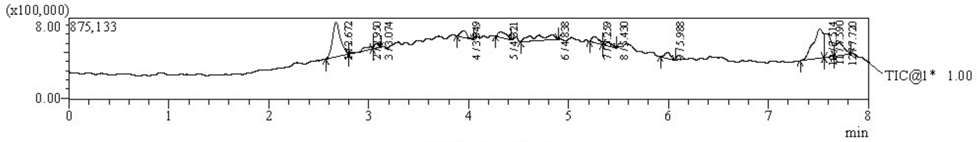

MS Spectrum Graph
#1 Ret.Time:Averaged 2.660-2.680(Scan#:267-269)
Mass Peaks:13 Base Peak:199.05(253253) Polarity:Pos Segment1 - Event1

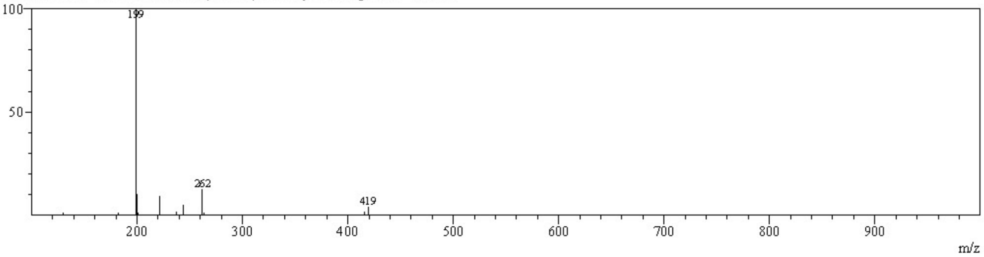

MS Spectrum Table

#:1 Ret.Time:
BG Mode:Calc 2.570<->2.800(258<->281)
Mass Peaks:13 Base Peak:199.05(253253) Polarity:Pos Segment1 - Event1

#	m/z	Abs.Inten.	Rel.Inten.	Charge	Polarity	Monoisotopic
1	83.05	22307	8.81			
2	130.25	3021	1.19			
3	182.15	3060	1.21			
4	199.05	253253	100.00			
5	200.05	25879	10.22			
6	201.05	2877	1.14			
7	221.10	22731	8.98			
8	237.10	3977	1.57			
9	244.30	12009	4.74			
10	261.95	31542	12.45			
11	263.20	3209	1.27			
12	415.90	4394	1.74			
13	419.20	9988	3.94			

Figure S13. LCMS spectrum of compound intermediate **7**.

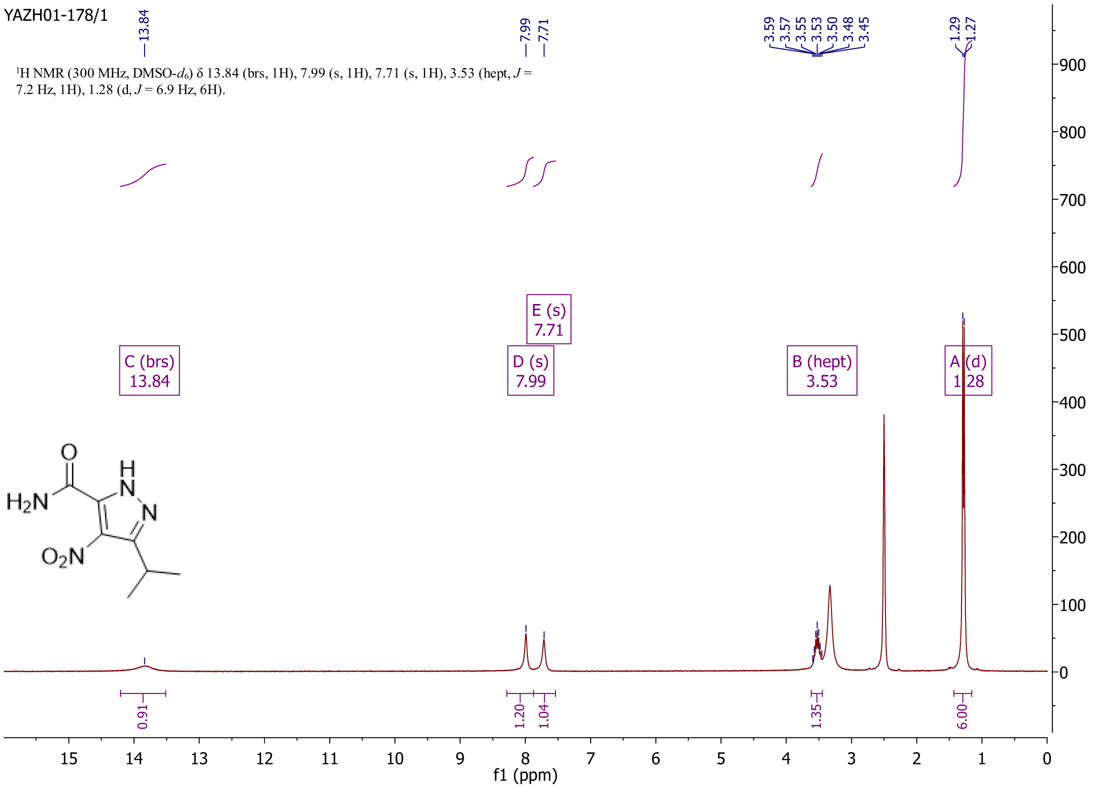

Figure S14. ^{1}H NMR spectrum of compound intermediate **7**.

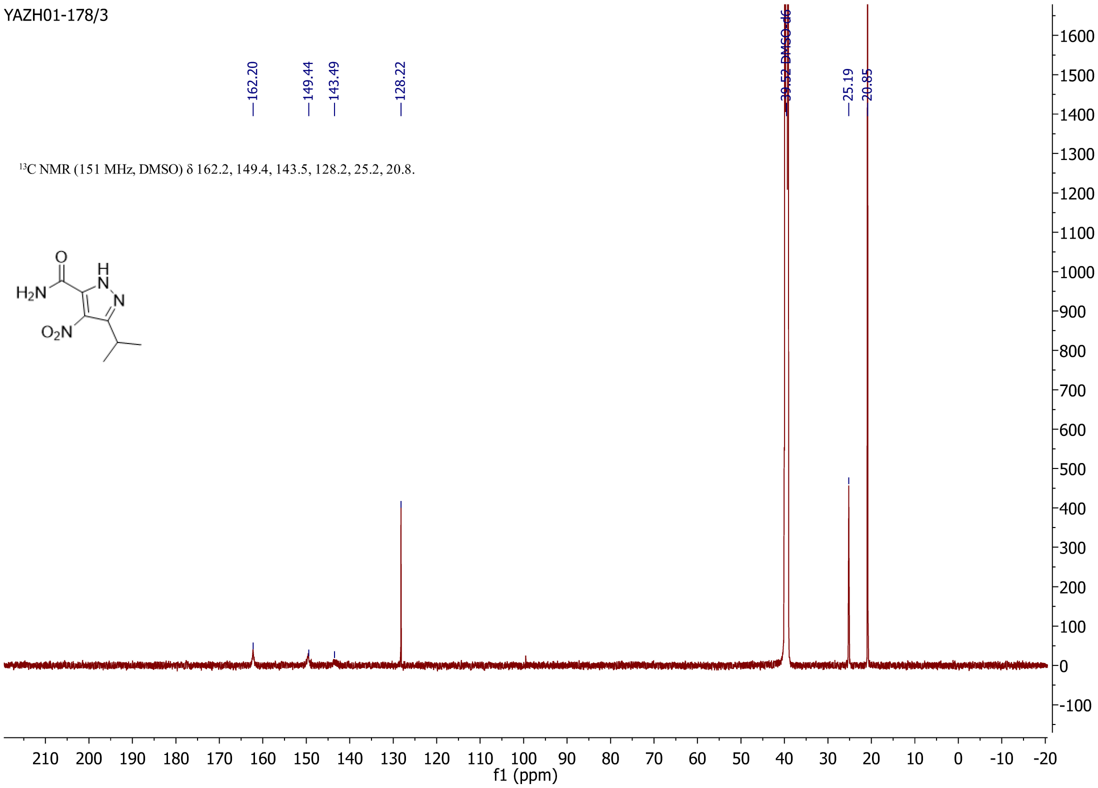

Figure S15. ^{13}C NMR spectrum of compound intermediate **7**.

Acquired by	: Admin
Date Acquired	: 4/6/2019 12:32:06 PM
Sample Name	: YAZH01-280
Sample ID	:
Tray#	: 1
Vial#	: 15
Injection Volume	: 2
Data File	: C:\LabSolutions\Data\2019\2019-wk23\YAZH01-280.lcd
Background File	: blanco2.lcd
Method File	: Method SCAN BASE standard.lcm
Report Format	: DefaultLCMS.lcr
Tuning File	: C:\LabSolutions\Tuning\Tuning-ESI-pos-neg01072015a.lct
Processed by	: Admin
Modified Date	: 4/6/2019 1:39:14 PM

PDA Graph

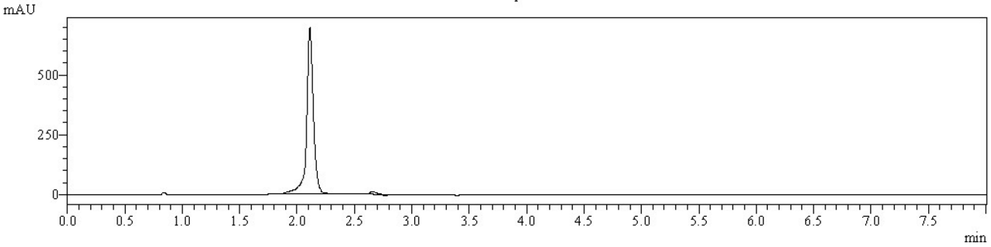

PeakTable

PDA Ch1 254nm 4nm

Peak#	Name	Ret. Time	Area	Area %
1		2.106	2851886	98.737
2		2.653	32373	1.121
3		5.243	4115	0.142

MS Chromatogram
YAZH01-280 C:\LabSolutions\Data\2019\2019-wk23\YAZH01-280.lcd

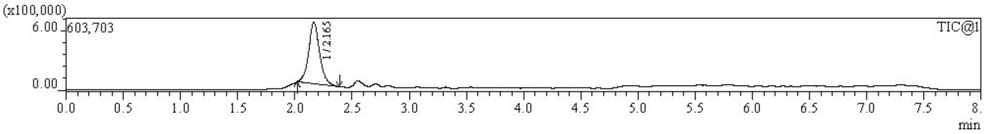

MS Spectrum Graph

#1 Ret.Time:Averaged 2.160-2.180(Scan#:217-219)
BG Mode:Calc 2.020<->2.390(203<->240)
Mass Peaks:8 Base Peak:169.00(340656) Polarity:Pos Segment1 - Event1

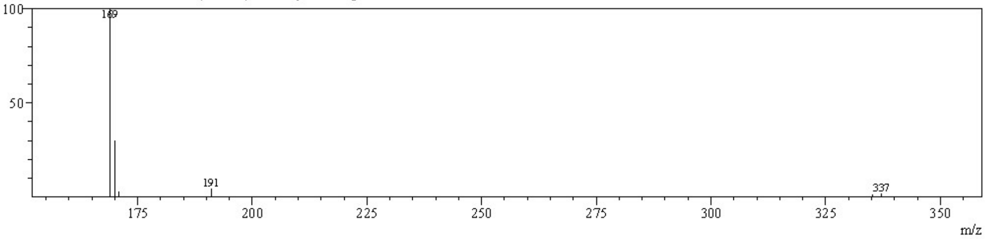

MS Spectrum Table

#:1 Ret.Time:
BG Mode:Calc 2.020<->2.390(203<->240)
Mass Peaks:8 Base Peak:169.00(340656) Polarity:Pos Segment1 - Event1

#	m/z	Abs.Inten.	Rel.Inten.	Charge	Polarity	Monoisotopic	#	m/z	Abs.Inten.	Rel.Inten.	Charge	Polarity	Monoisotopic
1	151.95	18506	5.43				5	191.00	14913	4.38			
2	169.00	340656	100.00				6	335.15	4759	1.40			
3	170.00	100719	29.57				7	337.10	4998	1.47			
4	171.00	8610	2.53				8	359.10	5800	1.70			

Figure S16. LCMS spectrum of compound **8**.

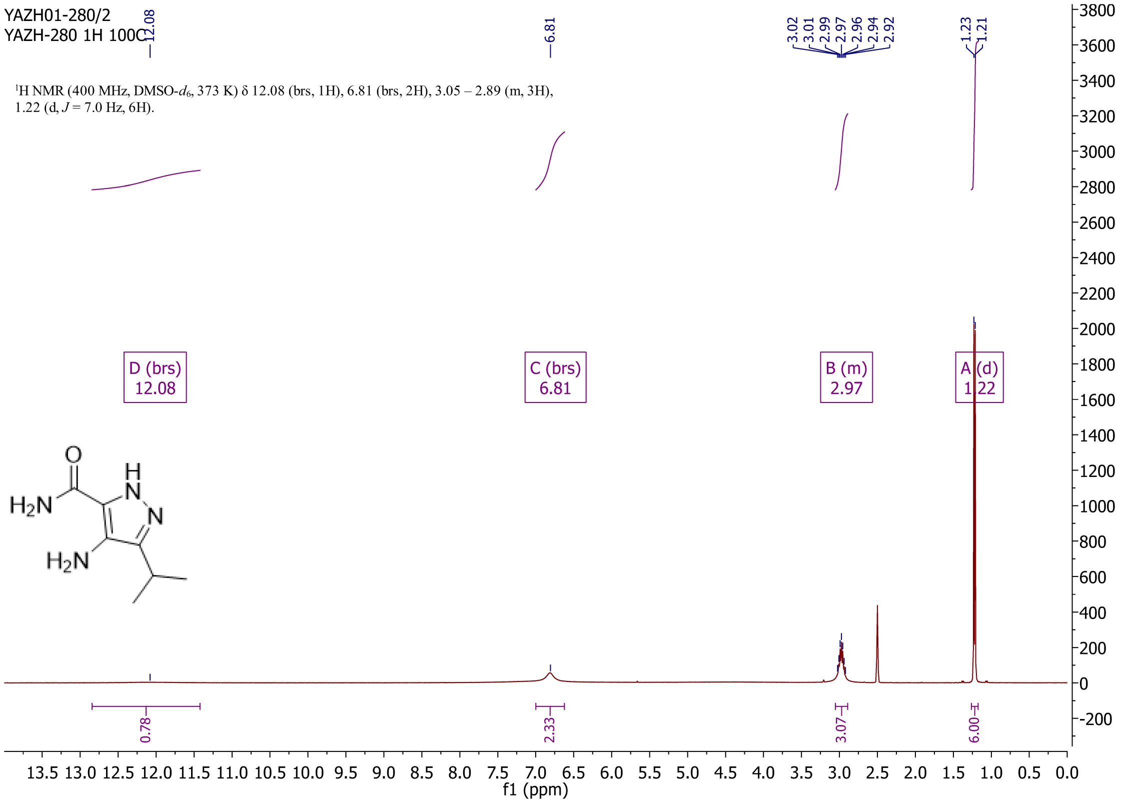

Figure S17. ^{1}H NMR spectrum of compound **8** at 373.15 K.

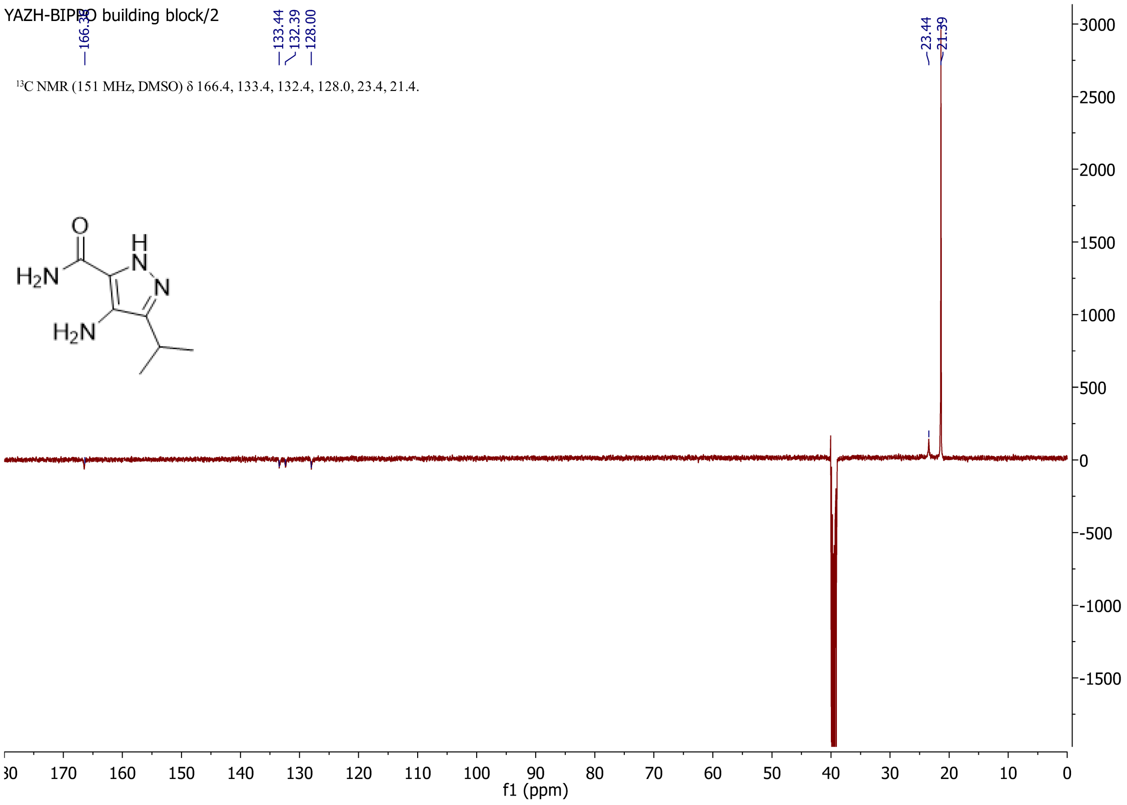

Figure S18. ^{13}C NMR spectrum of intermediate **8**.

Acquired by	: Admin
Date Acquired	: 11/6/2019 8:48:12 AM
Sample Name	: BIPPO
Sample ID	:
Tray#	: 1
Vial#	: 1
Injection Volume	: 2
Data File	: C:\LabSolutions\Data\2019\2019-wk24\BIPPO.lcd
Background File	: Blanco11062019.lcd
Method File	: Method SCAN ACID standard.lcm
Report Format	: DefaultLCMS.lcr
Tuning File	: C:\LabSolutions\Tuning\Tuning-ESI-pos-neg01072015a.lct
Processed by	: Admin
Modified Date	: 11/6/2019 9:17:16 AM

PDA Graph

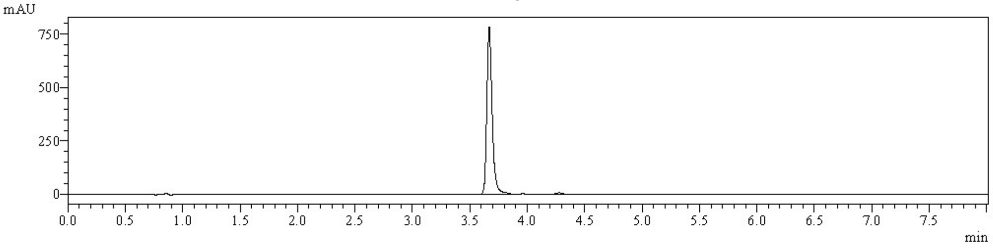

PeakTable

PDA Ch1 254nm 4nm

Peak#	Name	Ret. Time	Area	Area %
1		3.663	2492261	99.036
2		4.272	24258	0.964

MS Chromatogram
BIPPO C:\LabSolutions\Data\2019\2019-wk21-30\2019-wk24\BIPPO.lcd

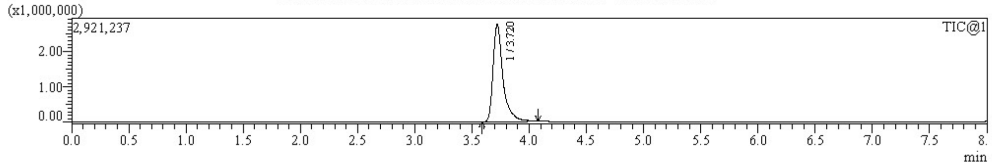

MS Spectrum Graph

#:1 Ret.Time:Averaged 3.710-3.730(Scan#:372-374)
BG Mode:Calc 3.590<->4.080(360<->409)
Mass Peaks:11 Base Peak:269.10(1938726) Polarity:Pos Segment1 - Event1

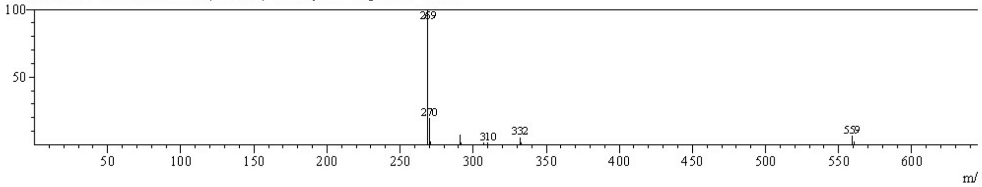

MS Spectrum Table

#:1 Ret.Time:
BG Mode:Calc 3.590<->4.080(360<->409)
Mass Peaks:11 Base Peak:269.10(1938726) Polarity:Pos Segment1 - Event1

#	m/z	Abs.Inten.	Rel.Inten.	Charge	Polarity	Monoisotopic
1	269.10	1938726	100.00			
2	270.10	372456	19.21			
3	271.05	38608	1.99			
4	291.10	138093	7.12			
5	292.05	27110	1.40			
6	307.05	21862	1.13			
7	310.15	25842	1.33			
8	332.10	103390	5.33			
9	333.10	21381	1.10			
10	559.30	122379	6.31			
11	560.30	39867	2.06			

Figure S19. LCMS spectrum of compound **1** (NPD-0019).

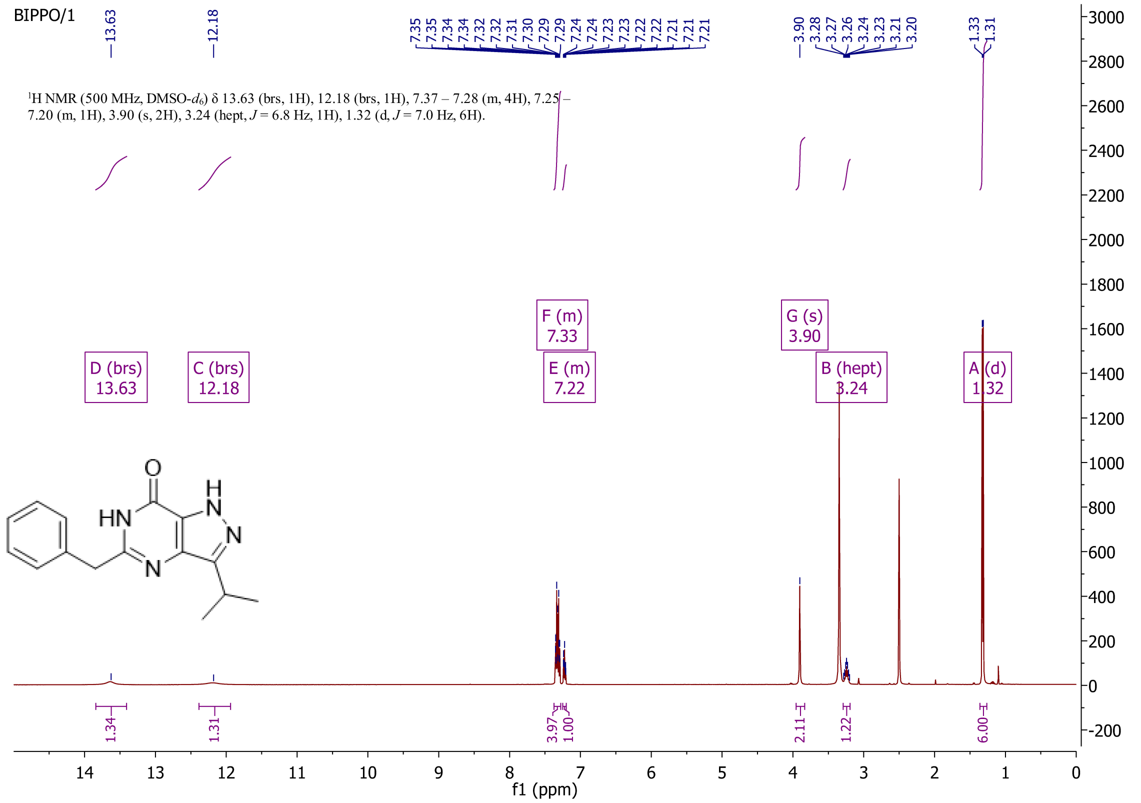

Figure S20. ^{1}H NMR spectrum of compound **1** (NPD-0019).

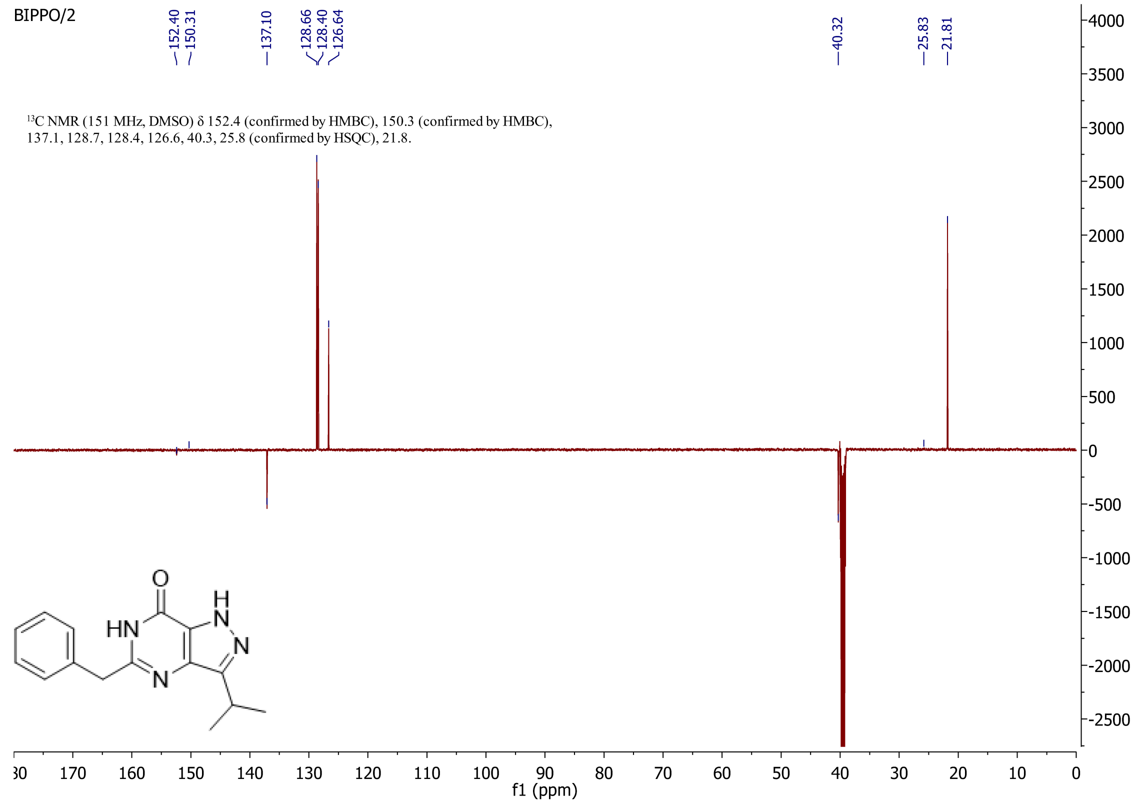

Figure S21. ^{13}C NMR spectrum of compound **1** (NPD-0019).

Acquired by	: Admin
Date Acquired	: 4/20/2016 2:03:12 PM
Sample Name	: YAZH-115
Sample ID	:
Tray#	: 1
Vial#	: 33
Injection Volume	: 1
Data File	: C:\LabSolutions\Data\2016 - wk16\YAZH-115.lcd
Background File	: blanco 20042016.lcd
Method File	: Method SCAN ACID standard.lcm
Report Format	: DefaultLCMS.lcr
Tuning File	: C:\LabSolutions\Tuning\Tuning-ESI-pos-neg01072015a.lct
Processed by	: Admin
Modified Date	: 4/20/2016 2:22:13 PM

PDA Graph

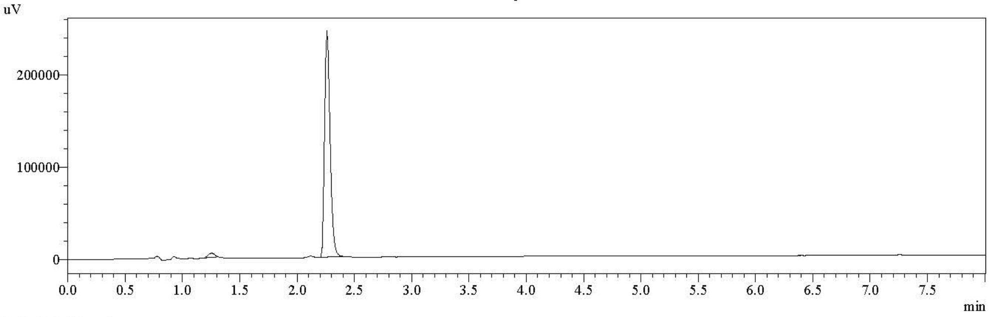

PDA Ch1 254nm 4nm

Peak#	Name	Ret. Time	Area	Area %
1		1.249	17766	2.151
2		2.257	808015	97.849

MS Spectrum Graph

#:1 Ret.Time:Averaged 2.340-2.360(Scan#:235-237)
Mass Peaks:11 Base Peak:270.05(167629) Polarity:Pos Segment1 - Event1

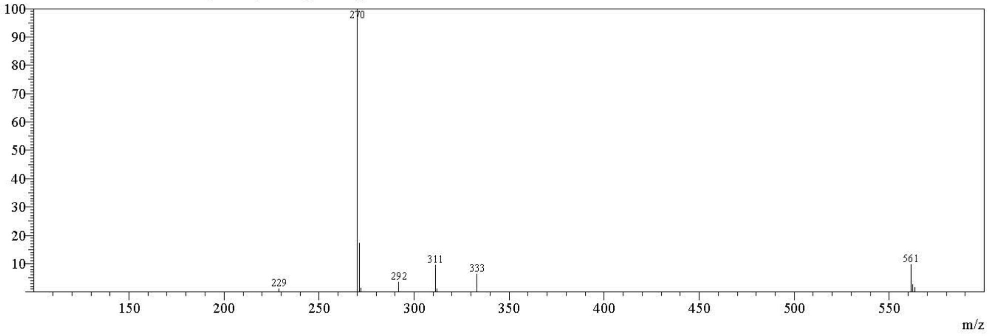

MS Spectrum Table

#:1 Ret.Time:
BG Mode:Calc 2.250<->2.510(226<->252)
Mass Peaks:11 Base Peak:270.05(167629) Polarity:Pos Segment1 - Event1

#	m/z	Abs.Inten.	Rel.Inten.	Charge	Polarity	Monoisotopic	#	m/z	Abs.Inten.	Rel.Inten.	Charge	Polarity	Monoisotopic
1	228.95	2033	1.21				7	312.20	1915	1.14			
2	270.05	167629	100.00				8	333.15	10548	6.29			
3	271.10	28885	17.23				9	561.30	16070	9.59			
4	272.15	2103	1.25				10	562.20	4684	2.79			
5	292.00	5834	3.48				11	563.35	2637	1.57			
6	311.10	15897	9.48										

Figure S22. LCMS spectrum of compound **9** (NPD-2960).

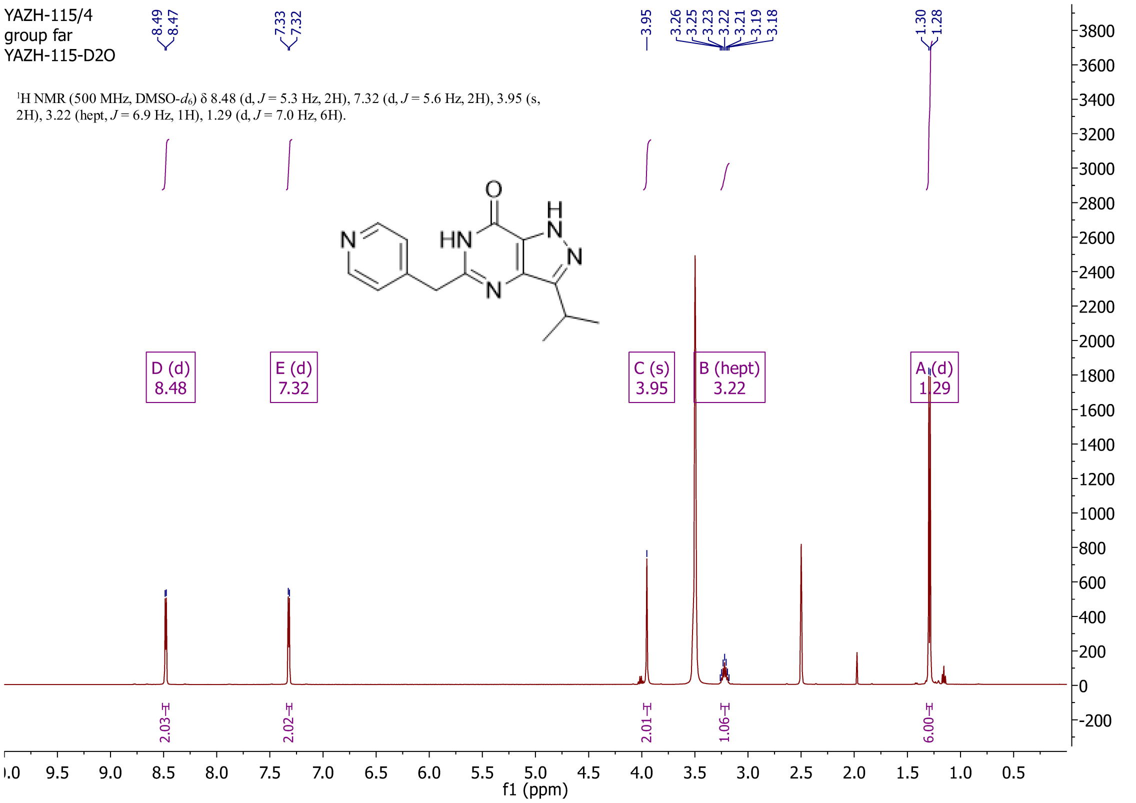

Figure S23. ^{1}H NMR spectrum of compound **9** (NPD-2960).

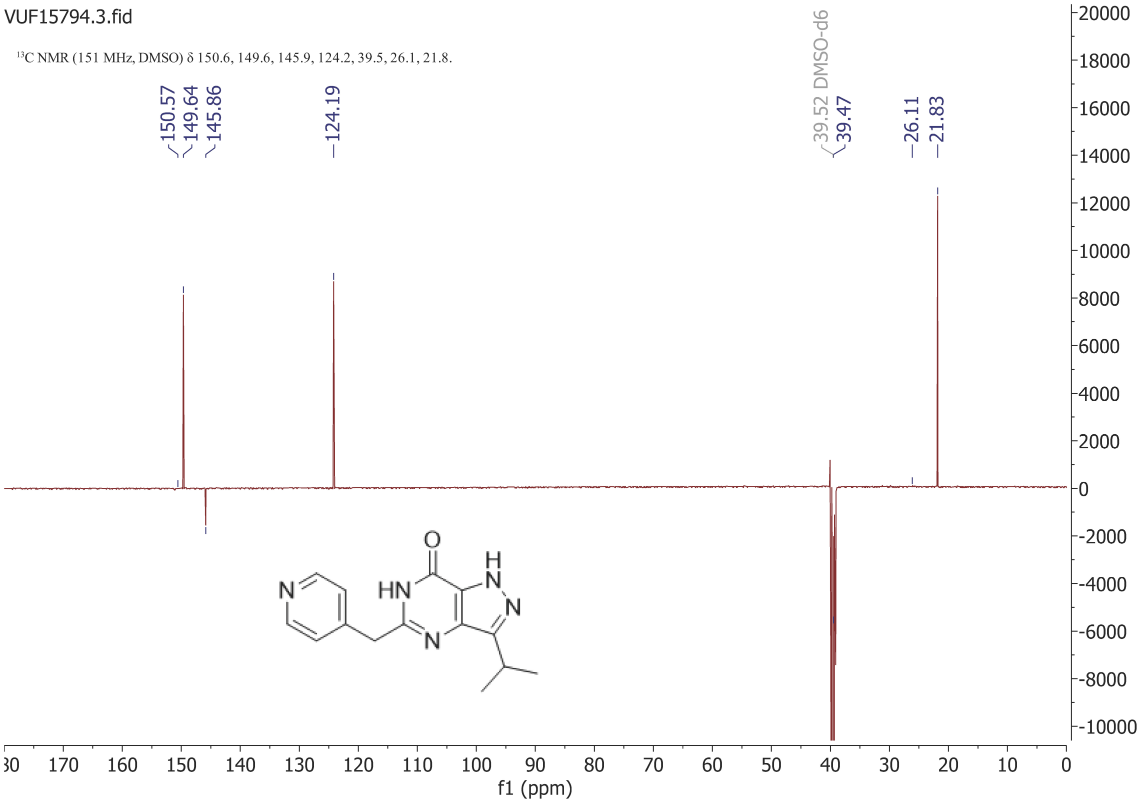

Figure S24. ^{13}C NMR spectrum of compound **9** (NPD-2960).

Acquired by	: Admin
Date Acquired	: 18/5/2020 10:17:40 AM
Sample Name	: VUF14762
Sample ID	:
Tray#	: 1
Vial#	: 2
Injection Volume	: 5
Data File	: C:\LabSolutions\Data\2020\2020-wk21\VUF14762.lcd
Background File	: blanco18052020.lcd
Method File	: Method SCAN ACID standard.lcm
Report Format	: DefaultLCMS.lcr
Tuning File	: C:\LabSolutions\Tuning\Tuning-ESI-pos-neg01072015a.lct
Processed by	: Admin
Modified Date	: 18/5/2020 11:40:37 AM

PDA Graph

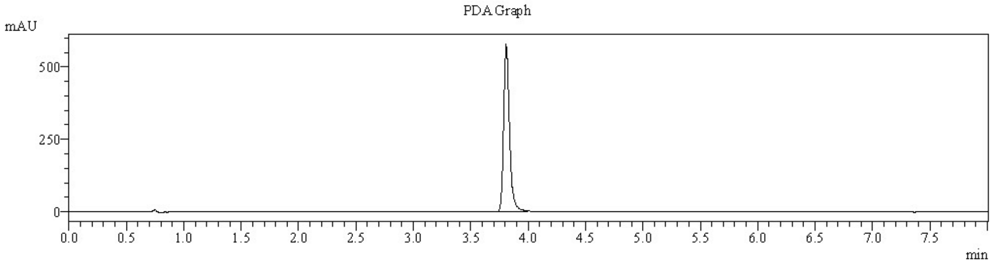

PeakTable

PDA Ch1 254nm 4nm

Peak#	Name	Ret. Time	Area	Area %
1		3.804	2001393	100.000

MS Chromatogram

VUF14762 C:\LabSolutions\Data\2020\2020-wk21\VUF14762.lcd

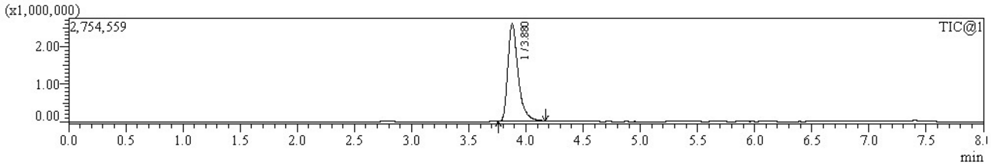

MS Spectrum Graph

#1 Ret.Time:Averaged 3.870-3.890(Scan#:388-390)
BG Mode:Calc 3.760<->4.170(377<->418)
Mass Peaks:15 Base Peak:285.15(1522515) Polarity:Pos Segment1 - Event1

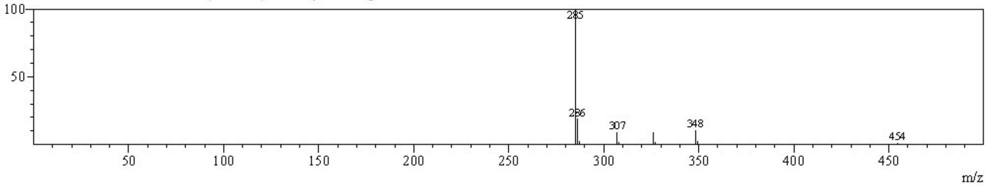

MS Spectrum Table

#:1 Ret.Time:
BG Mode:Calc 3.760<->4.170(377<->418)
Mass Peaks:15 Base Peak:285.15(1522515) Polarity:Pos Segment1 - Event1

#	m/z	Abs.Inten.	Rel.Inten.	Charge	Polarity	Monoisotopic
1	285.15	1522515	100.00			
2	286.15	289149	18.99			
3	287.15	29062	1.91			
4	307.15	136443	8.96			
5	308.15	26916	1.77			
6	326.20	128833	8.46			
7	327.20	27427	1.80			
8	348.15	158628	10.42			
9	349.25	36134	2.37			
10	454.55	16442	1.08			
11	591.40	46174	3.03			
12	592.40	15798	1.04			
13	596.40	26961	1.77			
14	596.70	48665	3.20			
15	597.75	22692	1.49			

Figure S25. LCMS spectrum of compound **10** (NPD-0434).

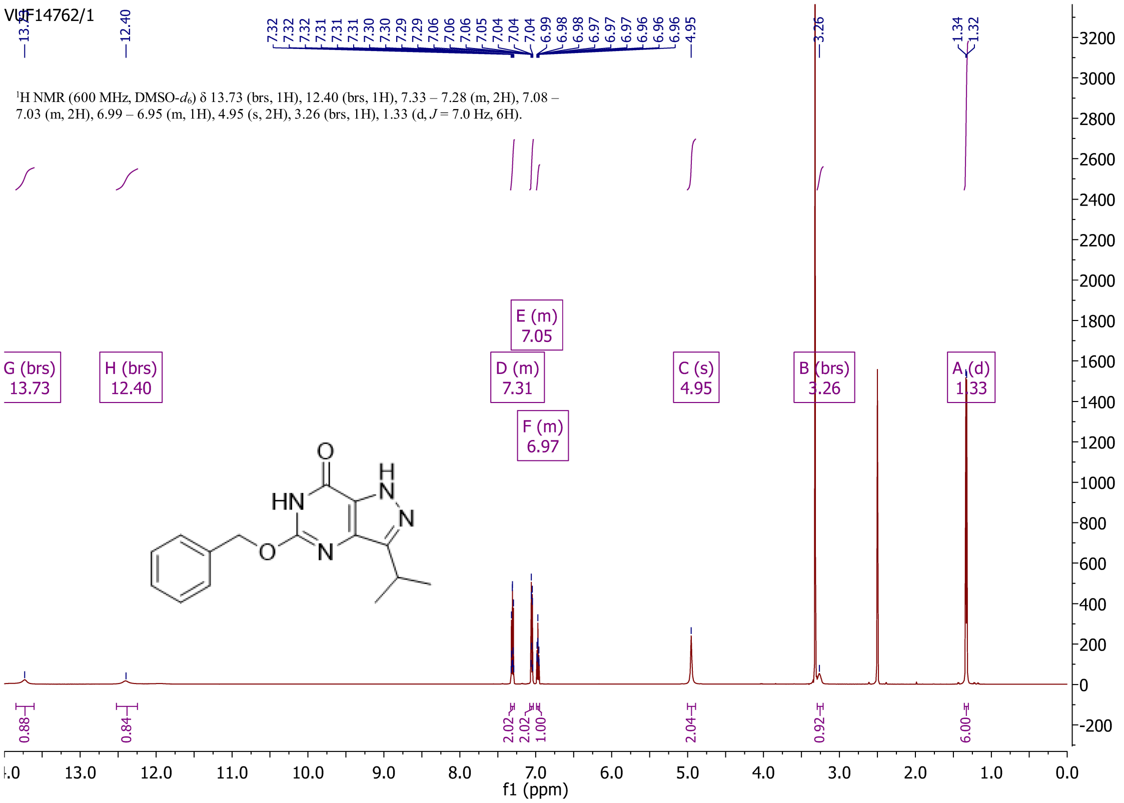

Figure S26. ¹H NMR spectrum of compound **10** (NPD-0434).

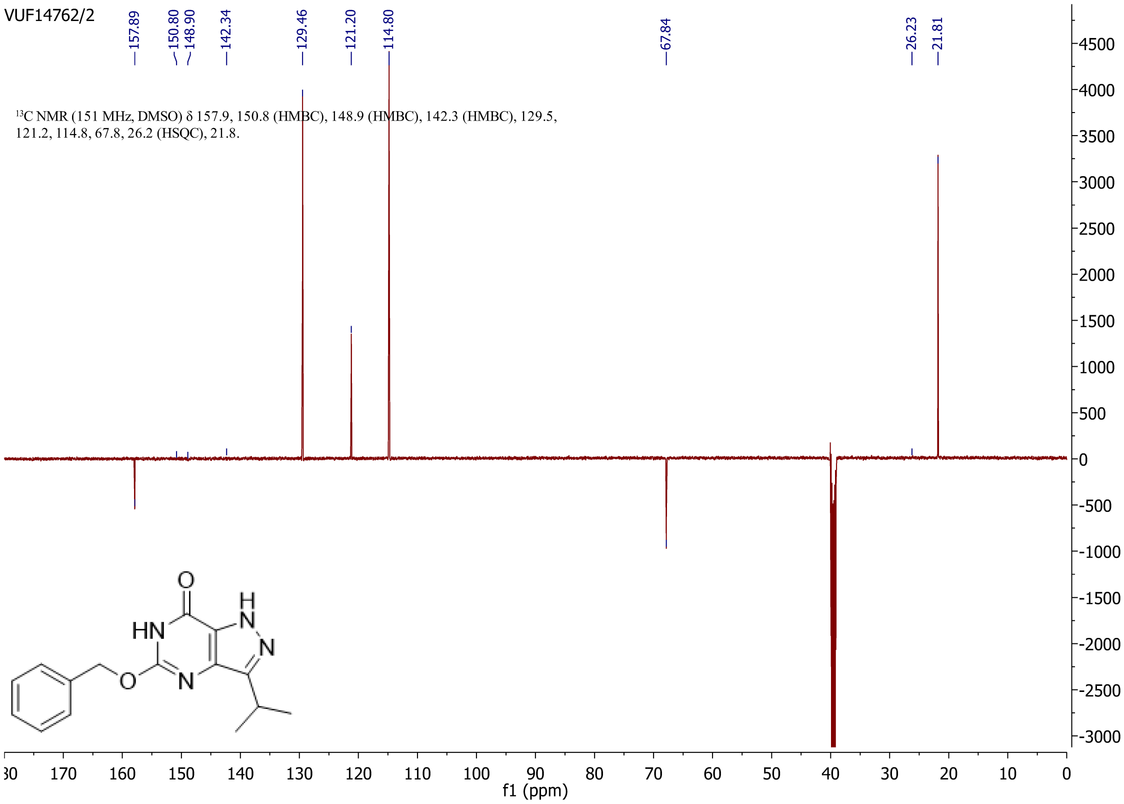

Figure S27. ¹³C NMR spectrum of compound **10** (NPD-0434).

Acquired by	: Admin
Date Acquired	: 13/12/2017 1:15:35 PM
Sample Name	: YAZH01-177-2
Sample ID	:
Tray#	: 1
Vial#	: 14
Injection Volume	: 3
Data File	: C:\LabSolutions\Data\2017\2017-wk50\YAZH01-177-2.lcd
Background File	: blanco 13122017.lcd
Method File	: Method SCAN ACID standard.lcm
Report Format	: DefaultLCMS.lcr
Tuning File	: C:\LabSolutions\Tuning\Tuning-ESI-pos-neg01072015a.lct
Processed by	: Admin
Modified Date	: 13/12/2017 3:51:59 PM

Chromatogram
YAZH01-177-2 C:\LabSolutions\Data\2017\2017-wk50\YAZH01-177-2.lcd

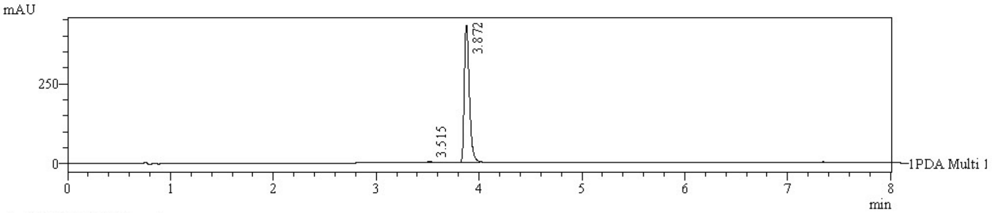

1 PDA Multi 1 / 254nm 4nm
PDA Ch1 254nm 4nm

Peak#	Name	Ret. Time	Area	Area%
1		3.515	10088	0.690
2		3.872	1451224	99.310

MS Chromatogram
YAZH01-177-2 C:\LabSolutions\Data\2017\2017-wk50\YAZH01-177-2.lcd

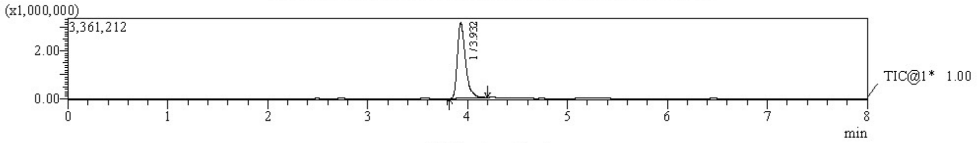

MS Spectrum Graph
#1 Ret.Time:Averaged 3.920-3.940(Scan#:393-395)
Mass Peaks:9 Base Peak:283.10(2292484) Polarity:Pos Segment1 - Event1

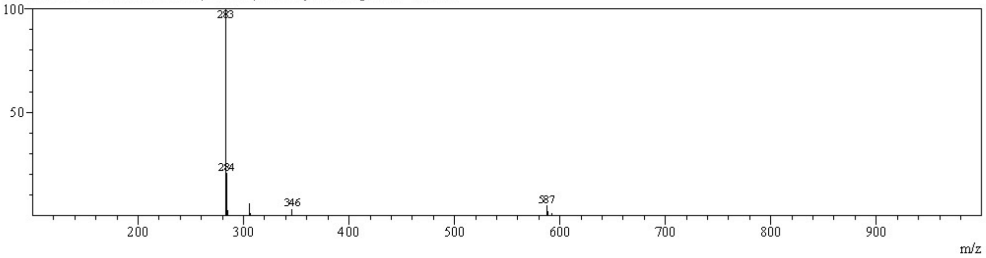

MS Spectrum Table
#:1 Ret.Time:
BG Mode:Calc 3.820<->4.200(383<->421)
Mass Peaks:9 Base Peak:283.10(2292484) Polarity:Pos Segment1 - Event1

#	m/z	Abs.Inten.	Rel.Inten.	Charge	Polarity	Monoisotopic
1	283.10	2292484	100.00			
2	284.10	466861	20.36			
3	285.15	56524	2.47			
4	305.10	128110	5.59			
5	306.10	23597	1.03			
6	346.15	72424	3.16			
7	587.30	112077	4.89			
8	588.30	42963	1.87			
9	592.40	30587	1.33			

Figure S28. LCMS spectrum of compound **11** (NPD-3281).

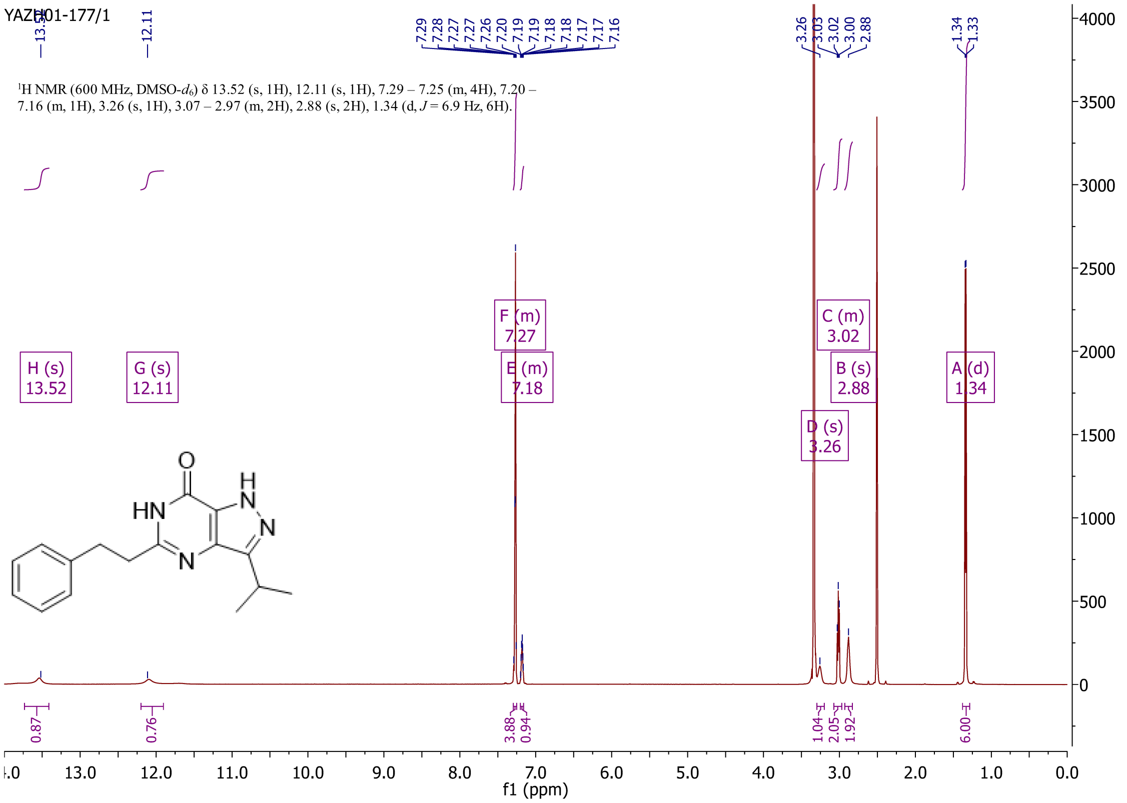

Figure S29. ^{1}H NMR spectrum of compound **11** (NPD-3281).

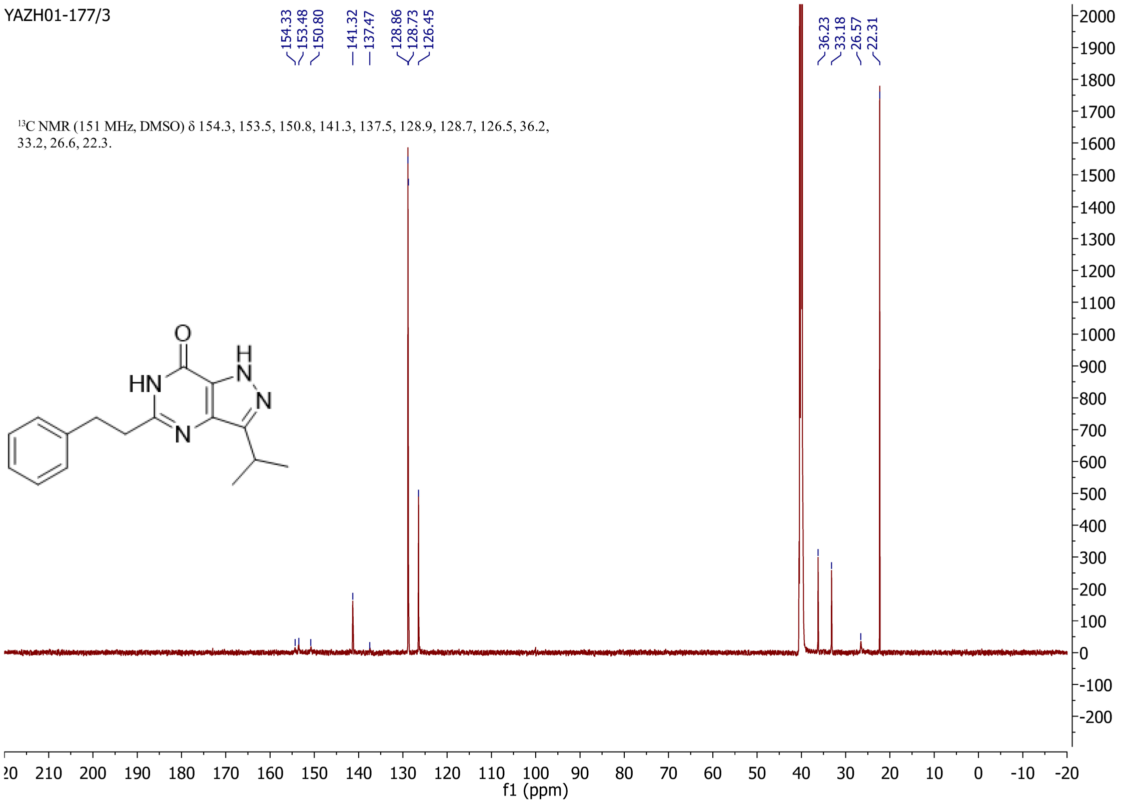

Figure S30. ^{13}C NMR spectrum of compound **11** (NPD-3281).

Acquired by	: Admin
Date Acquired	: 19/4/2018 1:51:00 PM
Sample Name	: YAZH01-204
Sample ID	:
Tray#	: 1
Vial#	: 19
Injection Volume	: 4
Data File	: C:\LabSolutions\Data\2018\2018-wk16\YAZH01-204.lcd
Background File	: blanco 19042018.lcd
Method File	: Method SCAN ACID standard.lcm
Report Format	: DefaultLCMS.lcr
Tuning File	: C:\LabSolutions\Tuning\Tuning-ESI-pos-neg01072015a.lct
Processed by	: Admin
Modified Date	: 19/4/2018 3:41:19 PM

Chromatogram
YAZH01-204 C:\LabSolutions\Data\2018\2018-wk16\YAZH01-204.lcd

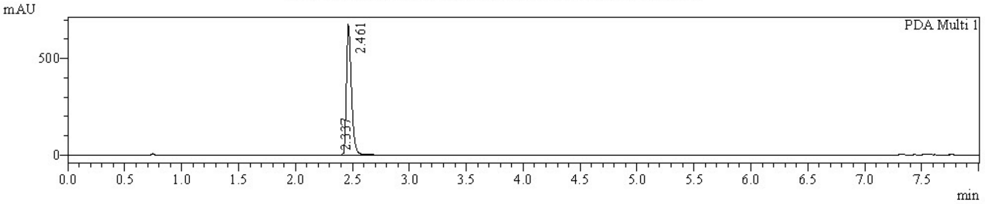

1 PDA Multi 1 / 254nm 4nm

PeakTable

PDA Ch1 254nm 4nm

Peak#	Name	Ret. Time	Area	Area%
1		2.337	446	0.022
2		2.461	2042814	99.978

MS Chromatogram
YAZH01-204 C:\LabSolutions\Data\2018\2018-wk16\YAZH01-204.lcd

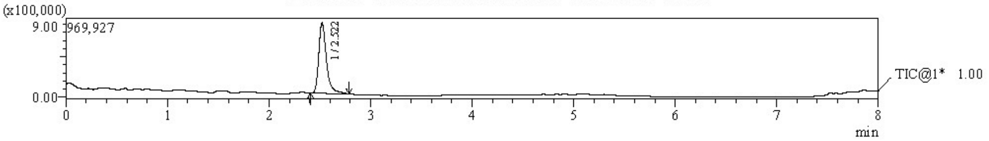

MS Spectrum Graph

#1 Ret.Time: Averaged 2.510-2.530(Scan#:252-254)
Mass Peaks:9 Base Peak:193.05(675266) Polarity:Pos Segment1 - Event1

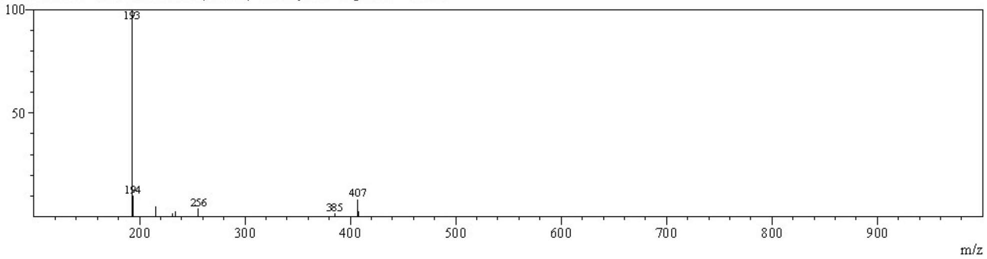

MS Spectrum Table

#:1 Ret.Time:
BG Mode:Calc 2.410<->2.790(242<->280)
Mass Peaks:9 Base Peak:193.05(675266) Polarity:Pos Segment1 - Event1

#	m/z	Abs.Inten.	Rel.Inten.	Charge	Polarity	Monoisotopic
1	193.05	675266	100.00			
2	194.05	65587	9.71			
3	215.00	32079	4.75			
4	231.00	8680	1.29			
5	234.05	16383	2.43			
6	256.05	24769	3.67			
7	385.05	9084	1.35			
8	407.15	55292	8.19			
9	408.15	15853	2.35			

Figure S31. LCMS spectrum of compound **12** (NPD-3380).

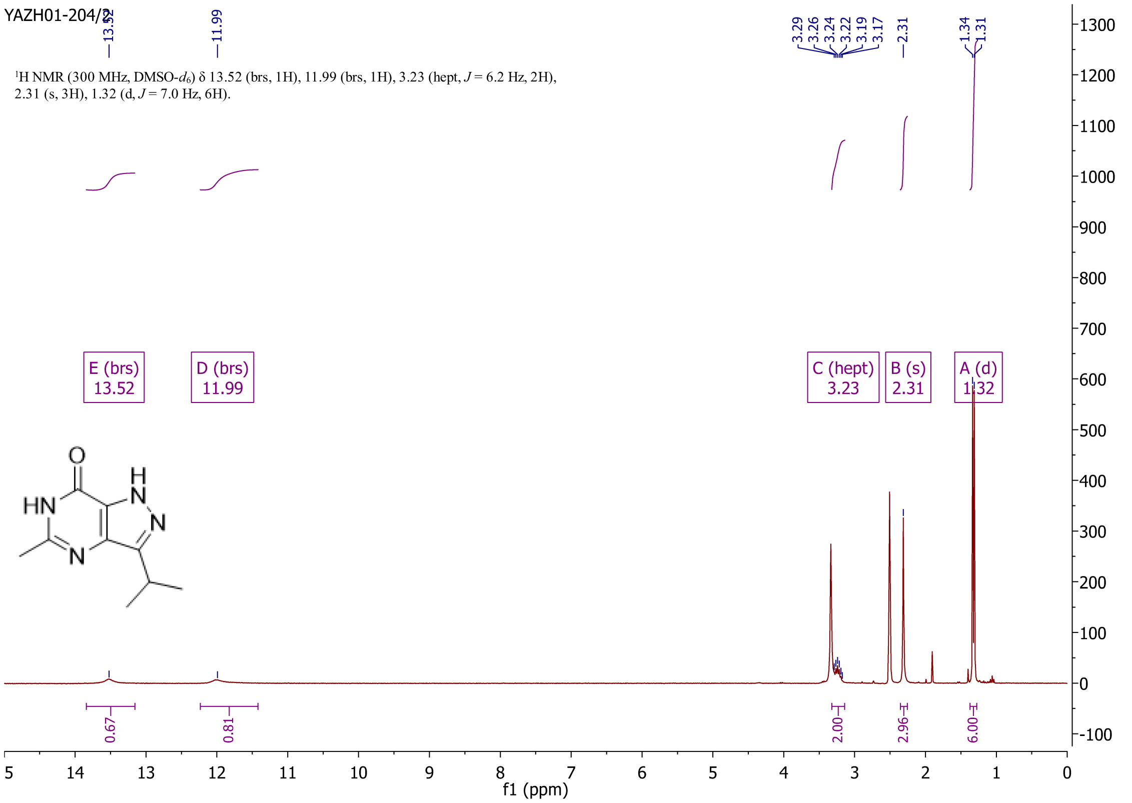

Figure S32. ^{1}H NMR spectrum of compound **12** (NPD-3380).

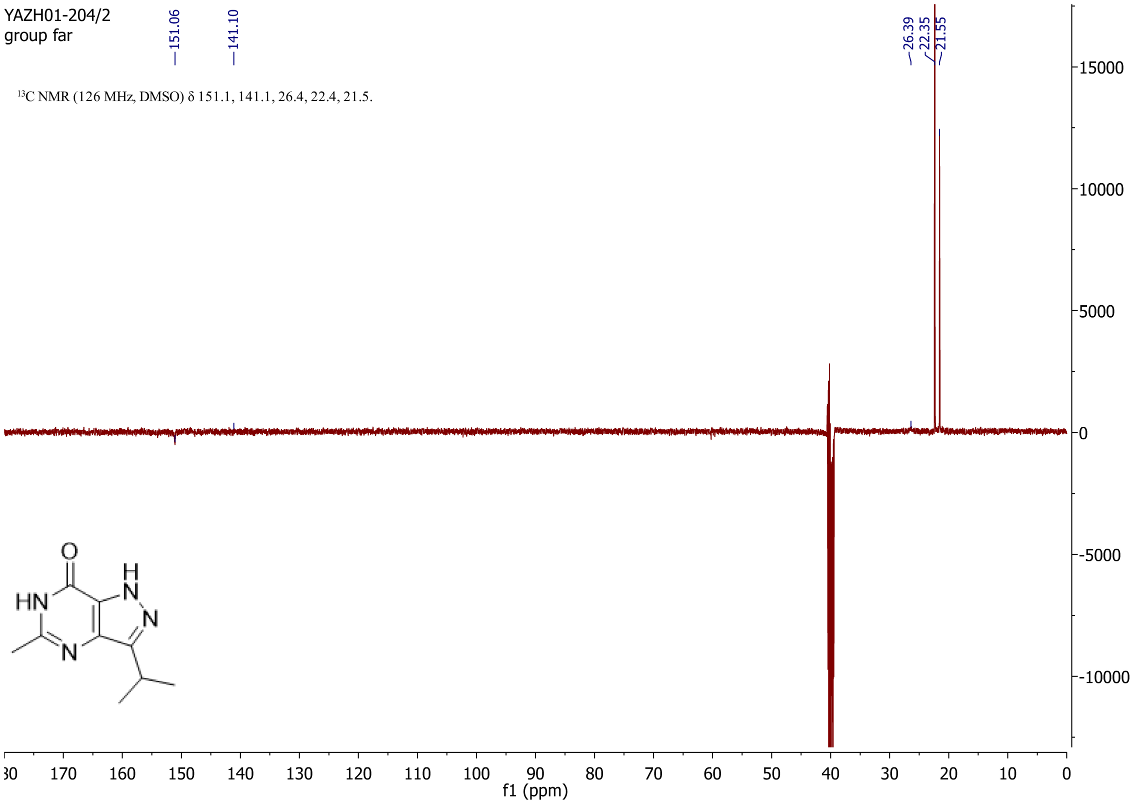

Figure S33. ^{13}C NMR spectrum of compound **12** (NPD-3380).

Acquired by	: Admin
Date Acquired	: 1/4/2019 11:04:28 AM
Sample Name	: YAZH_39
Sample ID	:
Tray#	: 1
Vial#	: 8
Injection Volume	: 3
Data File	: C:\LabSolutions\Data\2019\2019-wk14\YAZH_39.lcd
Background File	: blanco 01042019.lcd
Method File	: Method SCAN ACID standard.lcm
Report Format	: DefaultLCMS.lcr
Tuning File	: C:\LabSolutions\Tuning\Tuning-ESI-pos-neg01072015a.lct
Processed by	: Admin
Modified Date	: 1/4/2019 11:34:36 AM

PDA Graph

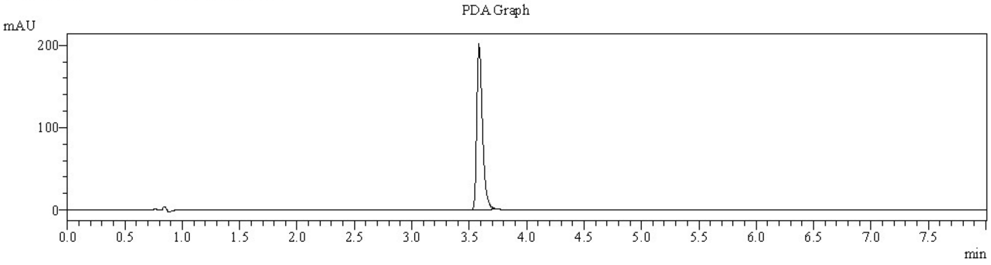

PeakTable

PDA Ch1 254nm 4nm

Peak#	Name	Ret. Time	Area	Area%
1		3.581	688185	100.000

MS Chromatogram
YAZH_39 C:\LabSolutions\Data\2019\2019-wk14\YAZH_39.lcd

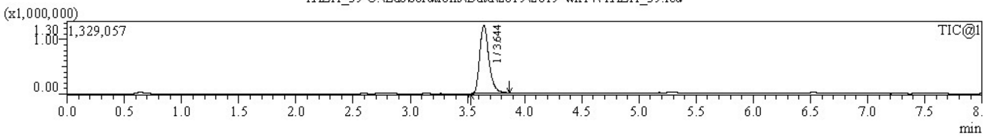

MS Spectrum Graph

#1 Ret.Time:Averaged 3.630-3.650(Scan#:364-366)
BG Mode:Calc 3.530<->3.870(354<->388)
Mass Peaks:6 Base Peak:235.05(956820) Polarity:Pos Segment1 - Event1

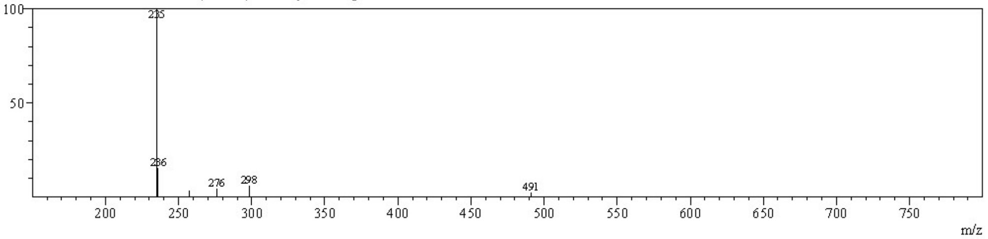

MS Spectrum Table

#:1 Ret.Time:
BG Mode:Calc 3.530<->3.870(354<->388)
Mass Peaks:6 Base Peak:235.05(956820) Polarity:Pos Segment1 - Event1

#	m/z	Abs.Inten.	Rel.Inten.	Charge	Polarity	Monoisotopic
1	235.05	956820	100.00			
2	236.05	144245	15.08			
3	257.10	31609	3.30			
4	276.10	38169	3.99			
5	298.10	56634	5.92			
6	491.30	21946	2.29			

Figure S34. LCMS spectrum of compound **13** (NPD-3645).

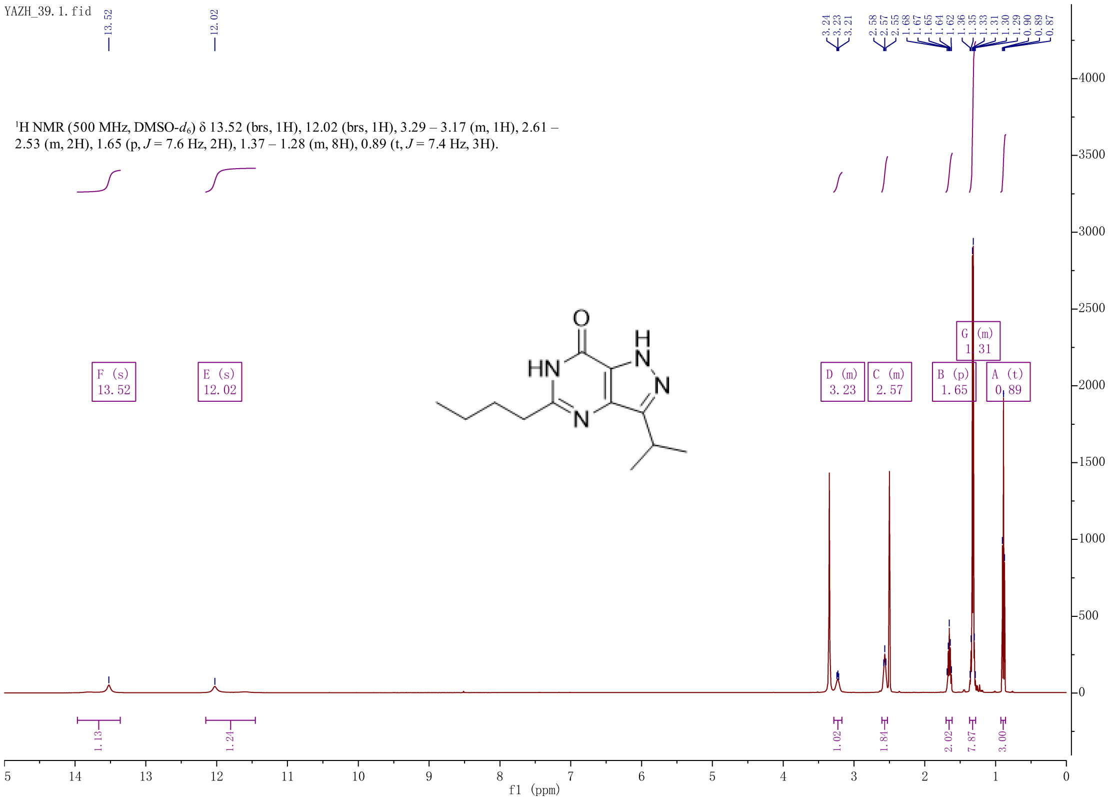

Figure S35. ^{1}H NMR spectrum of compound **13** (NPD-3645).

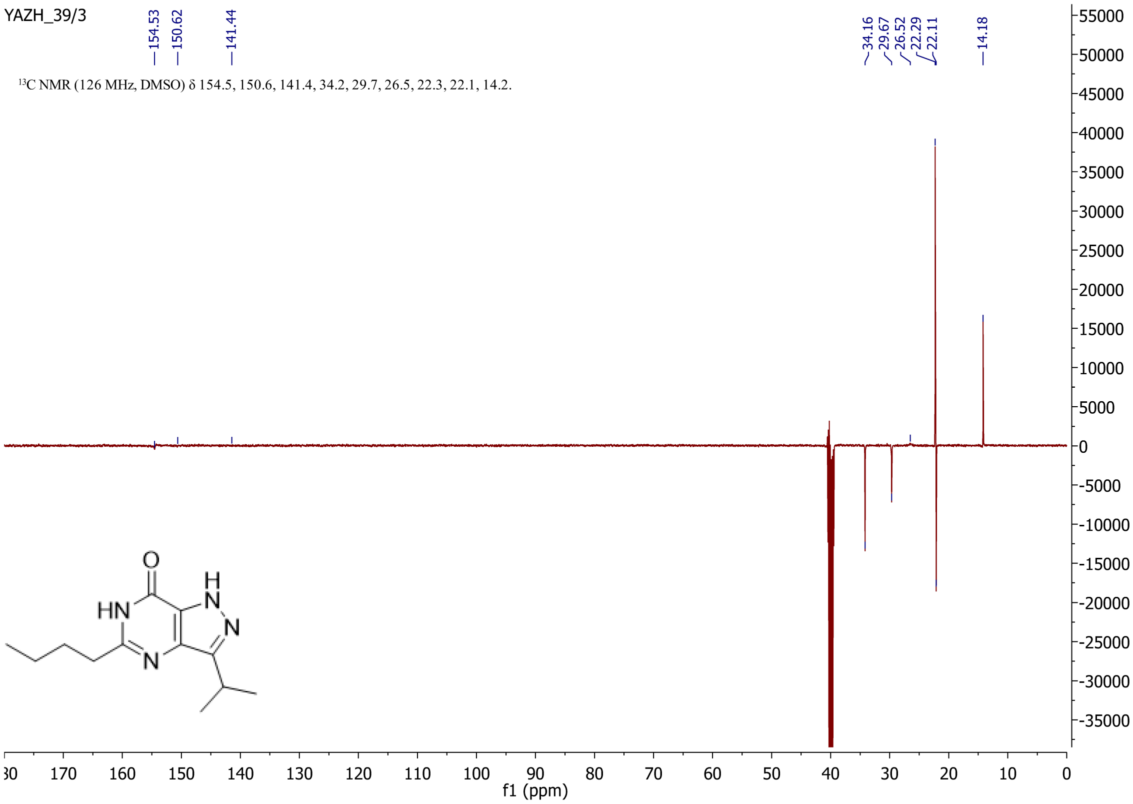

Figure S36. ^{13}C NMR spectrum of compound **13** (NPD-3645).

Acquired by : Admin
Date Acquired : 19/4/2018 12:59:05 PM
Sample Name : YAZH01-203-1
Sample ID :
Tray# : 1
Vial# : 28
Injection Volume : 4
Data File : C:\LabSolutions\Data\2018\2018-wk16\YAZH01-203-1.lcd
Background File : blanco 19042018.lcd
Method File : Method SCAN ACID standard.lcm
Report Format : DefaultLCMS.lcr
Tuning File : C:\LabSolutions\Tuning\Tuning-ESI-pos-neg01072015a.lct
Processed by : Admin
Modified Date : 19/4/2018 1:10:48 PM

Chromatogram
YAZH01-203-1 C:\LabSolutions\Data\2018\2018-wk16\YAZH01-203-1.lcd

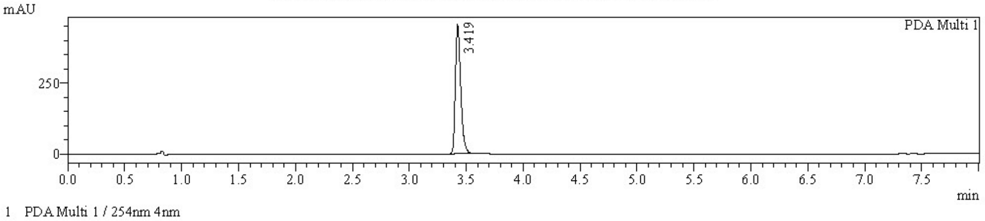

PeakTable

PDA Ch1 254nm 4nm

Peak#	Name	Ret. Time	Area	Area %
1		3.419	1470710	100.000

MS Chromatogram
YAZH01-203-1 C:\LabSolutions\Data\2018\2018-wk16\YAZH01-203-1.lcd

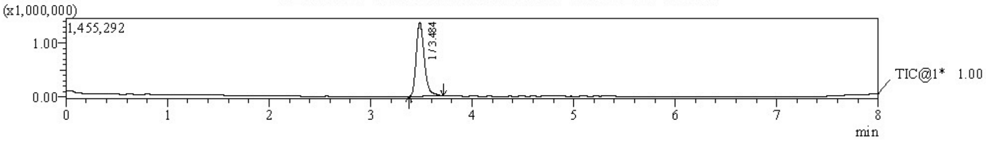

MS Spectrum Graph
#1 Ret.Time: Averaged 3.470-3.490(Scan#:348-350)
Mass Peaks:8 Base Peak:221.10(1031293) Polarity:Pos Segment1 - Event1

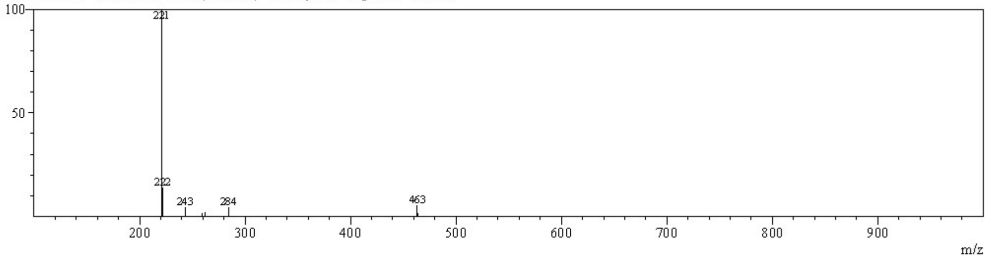

MS Spectrum Table
#:1 Ret.Time:
BG Mode:Calc 3.380<->3.720(339<->373)
Mass Peaks:8 Base Peak:221.10(1031293) Polarity:Pos Segment1 - Event1

#	m/z	Abs.Inten.	Rel.Inten.	Charge	Polarity	Monoisotopic
1	221.10	1031293	100.00			
2	222.05	140359	13.61			
3	243.00	41841	4.06			
4	259.10	11590	1.12			
5	262.05	20613	2.00			
6	284.10	42671	4.14			
7	463.25	50815	4.93			
8	464.20	15306	1.48			

Figure S37. LCMS spectrum of compound **14** (NPD-3379).

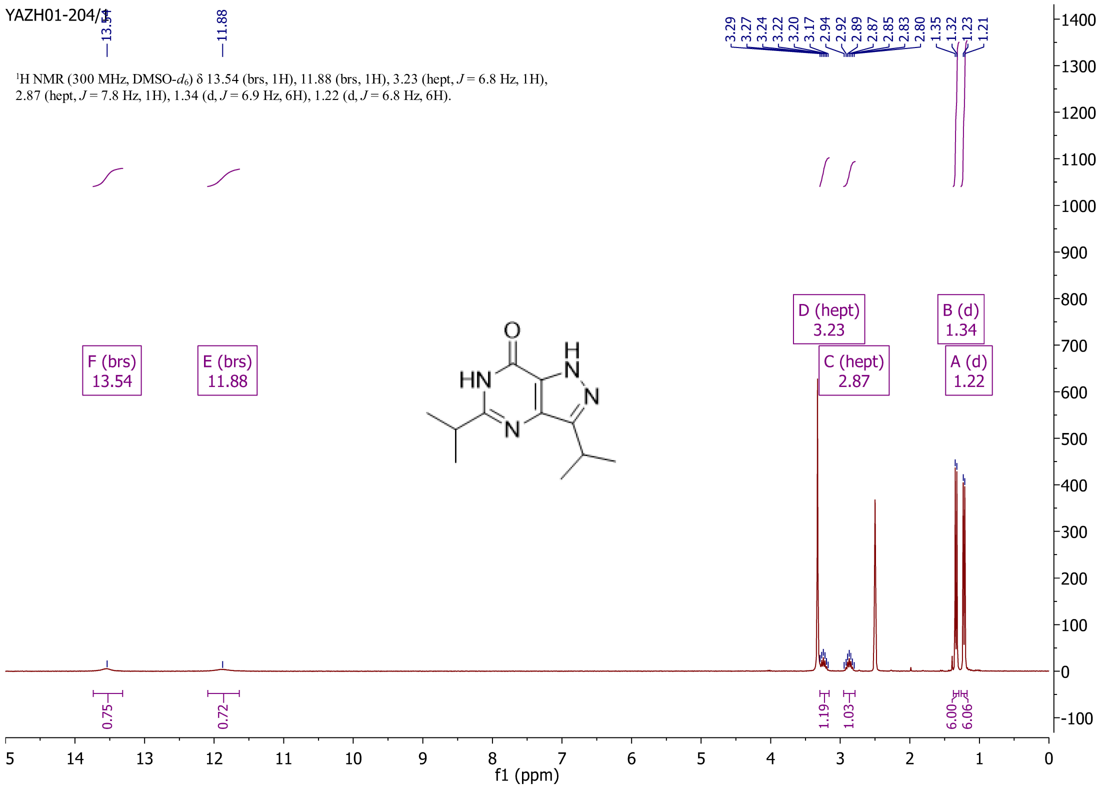

Figure S38. ^{1}H NMR spectrum of compound **14** (NPD-3379).

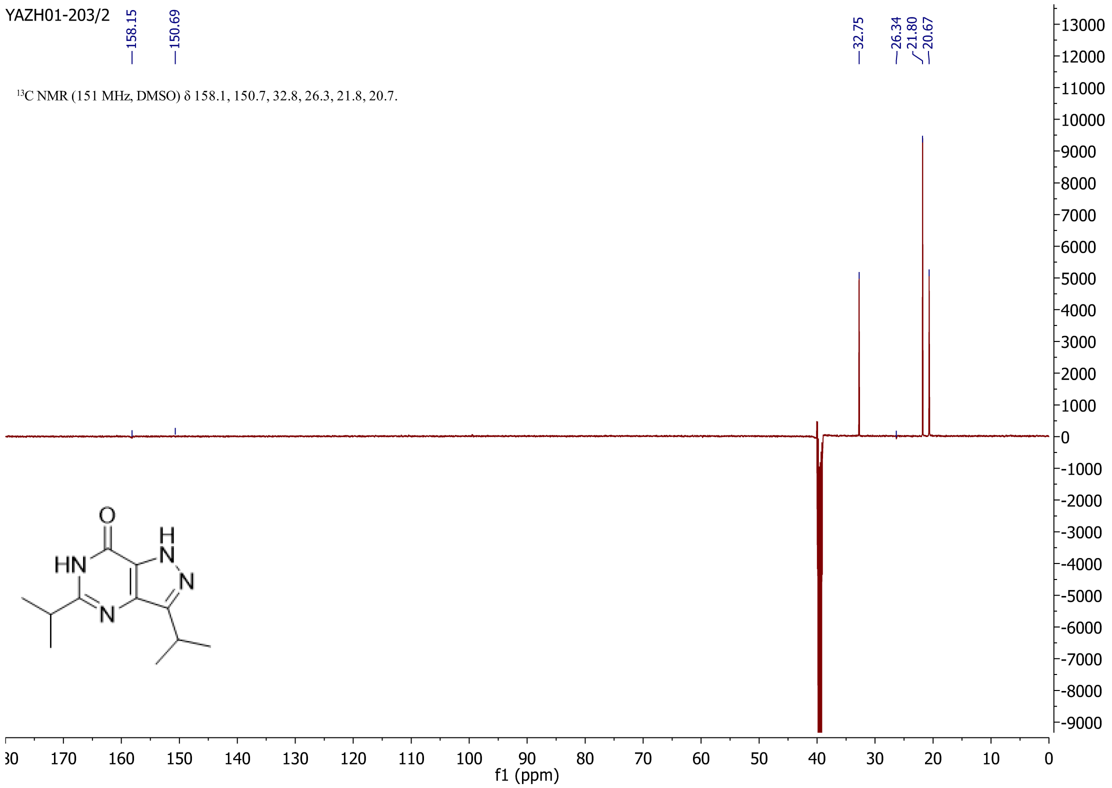

Figure S39. ^{13}C NMR spectrum of compound **14** (NPD-3379).

Acquired by : Admin
Date Acquired : 7/14/2017 4:35:06 PM
Sample Name : YAZH01-116
Sample ID :
Tray# : 1
Vial# : 28
Injection Volume : 10
Data File : C:\LabSolutions\Data\2017\2017-wk28\YAZH01-116.lcd
Background File : BLANCO_14072017.lcd
Method File : Method SCAN ACID standard.lcm
Report Format : DefaultLCMS.lcr
Tuning File : C:\LabSolutions\Tuning\Tuning-ESI-pos-neg01072015a.lct
Processed by : Admin
Modified Date : 7/14/2017 5:24:01 PM

Chromatogram
YAZH01-116 C:\LabSolutions\Data\2017\2017-wk28\YAZH01-116.lcd

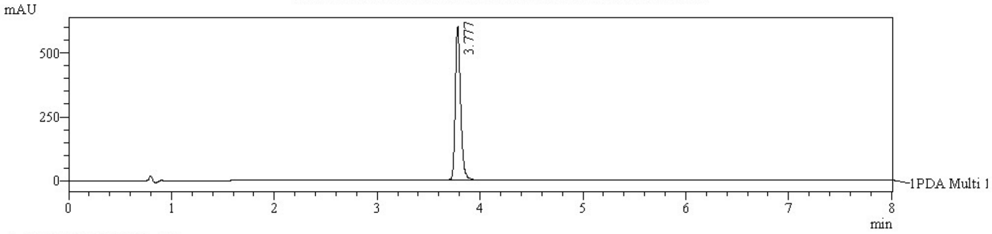

1 PDA Multi 1 / 254nm 4nm

PeakTable

PDA Ch1 254nm 4nm

Peak#	Name	Ret. Time	Area	Area %
1		3.777	2126142	100.000

MS Chromatogram
YAZH01-116 C:\LabSolutions\Data\2017\2017-wk28\YAZH01-116.lcd

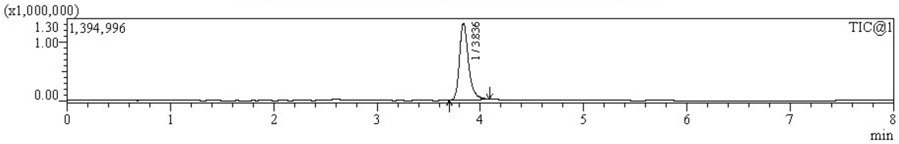

MS Spectrum Graph
#1 Ret.Time:Averaged 3.830-3.850(Scan#:384-386)
Mass Peaks:13 Base Peak:255.10(772560) Polarity:Pos Segment1 - Event1

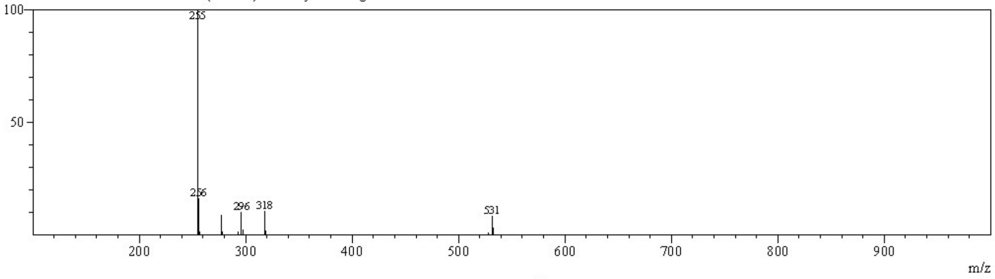

MS Spectrum Table
#:1 Ret.Time:
BG Mode:Calc 3.700<->4.090(371<->410)
Mass Peaks:13 Base Peak:255.10(772560) Polarity:Pos Segment1 - Event1

#	m/z	Abs.Inten.	Rel.Inten.	Charge	Polarity	Monoisotopic
1	255.10	772560	100.00			
2	256.10	125886	16.29			
3	257.10	10257	1.33			
4	277.10	66368	8.59			
5	278.10	9738	1.26			
6	293.00	11959	1.55			
7	296.10	76995	9.97			
8	297.15	16084	2.08			
9	318.10	80834	10.46			
10	319.10	15800	2.05			
11	528.50	7861	1.02			
12	531.30	64127	8.30			
13	532.30	24020	3.11			

Figure S40. LCMS spectrum of compound **15** (NPD-3200).

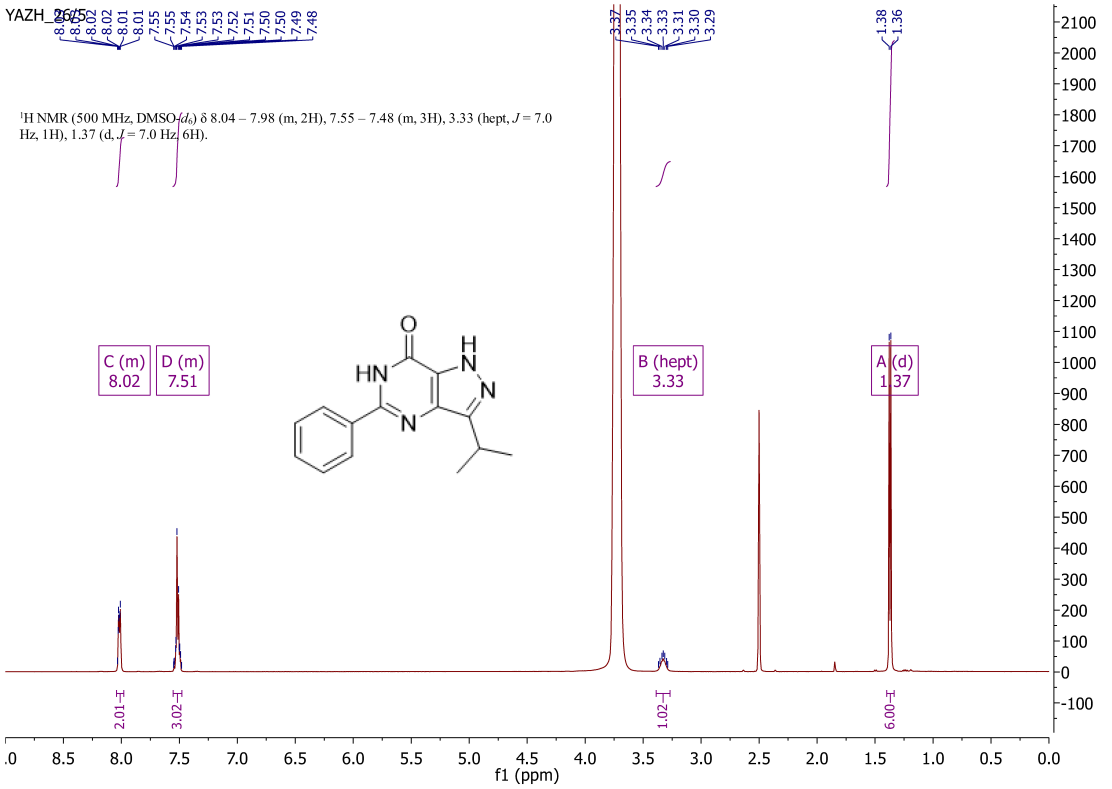

Figure S41. ^{1}H NMR spectrum of compound **15** (NPD-3200).

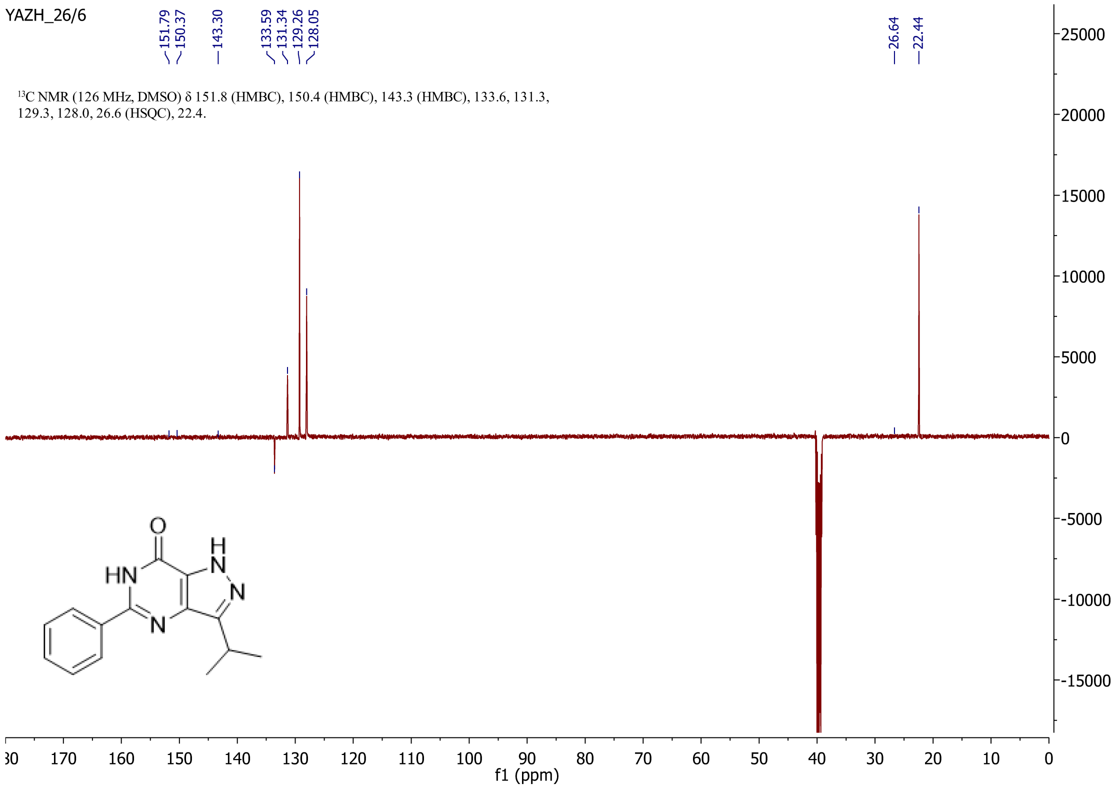

Figure S42. ^{13}C NMR spectrum of compound **15** (NPD-3200).

Acquired by	: Admin
Date Acquired	: 7/5/2018 1:29:57 PM
Sample Name	: YAZH01-209
Sample ID	:
Tray#	: 1
Vial#	: 25
Injection Volume	: 3
Data File	: C:\LabSolutions\Data\2018\2018-wk19\YAZH01-209.lcd
Background File	: blanco_070518.lcd
Method File	: Method SCAN ACID standard.lcm
Report Format	: DefaultLCMS.lcr
Tuning File	: C:\LabSolutions\Tuning\Tuning-ESI-pos-neg01072015a.lct
Processed by	: Admin
Modified Date	: 7/5/2018 1:54:47 PM

PDA Graph

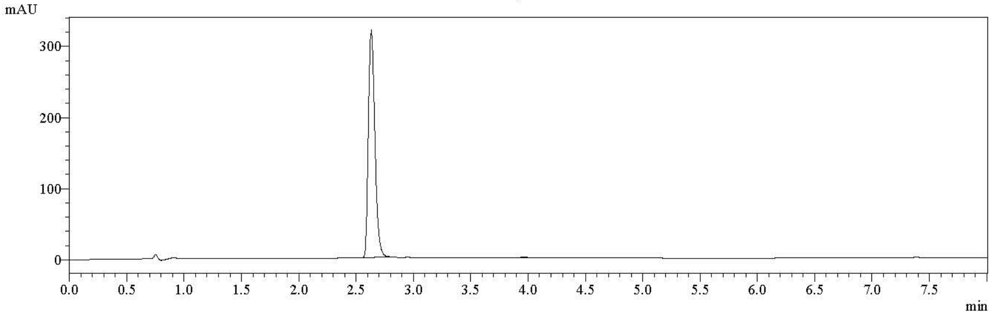

PDA Ch1 254nm 4nm

Peak#	Name	Ret. Time	Area	Area %
1		2.485	2629	0.207
2		2.627	1261672	99.458
3		3.961	4251	0.335

MS Spectrum Graph

#:1 Ret.Time:Averaged 2.690-2.710(Scan#:270-272)
Mass Peaks:10 Base Peak:256.05(637614) Polarity:Pos Segment1 - Event1

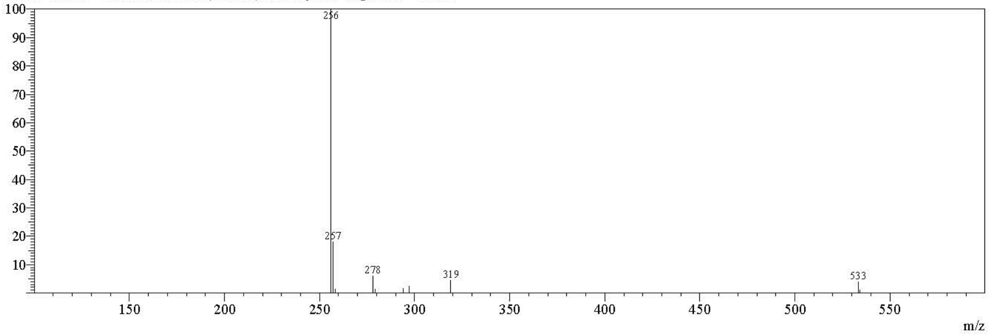

MS Spectrum Table

#:1 Ret.Time:
BG Mode:Calc 2.580<->2.920(259<->293)
Mass Peaks:10 Base Peak:256.05(637614) Polarity:Pos Segment1 - Event1

#	m/z	Abs.Inten.	Rel.Inten.	Charge	Polarity	Monoisotopic
1	256.05	637614	100.00			
2	257.00	114485	17.96			
3	258.10	8195	1.29			
4	278.05	38614	6.06			
5	279.10	8815	1.38			
6	294.10	9417	1.48			
7	297.15	15763	2.47			
8	319.05	28833	4.52			
9	533.25	24938	3.91			
10	534.20	7571	1.19			

Figure S43. LCMS spectrum of compound **16** (NPD-3488).

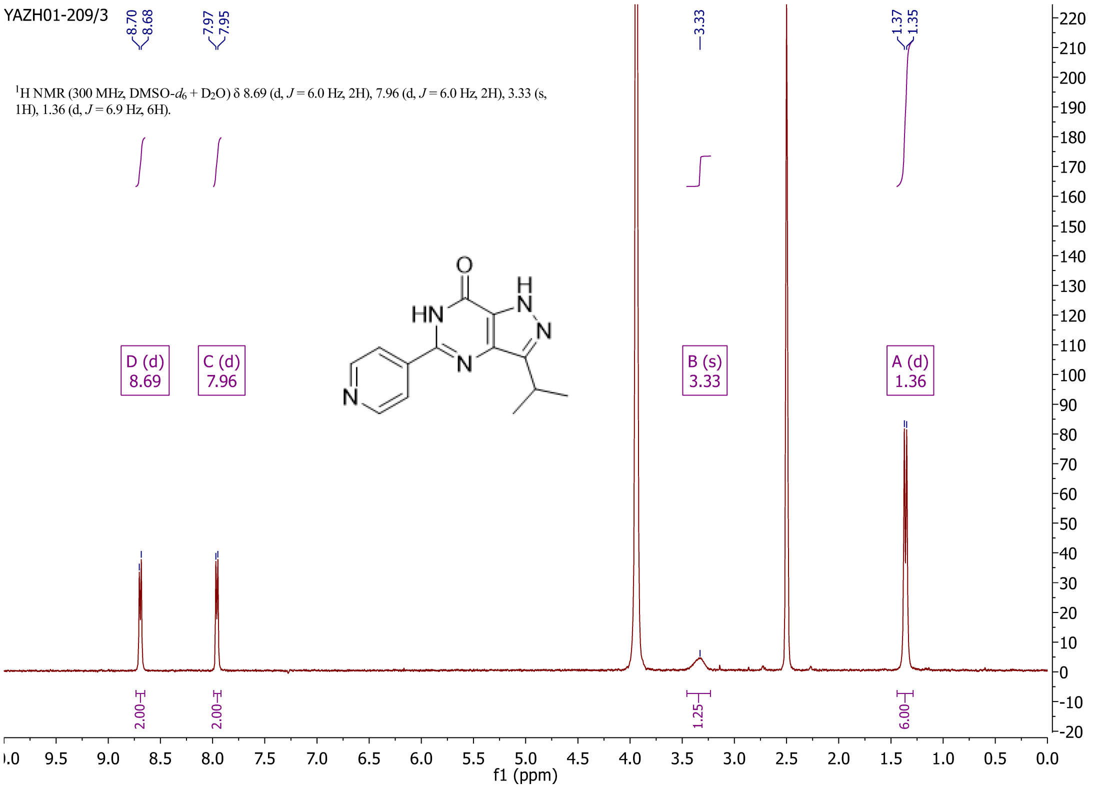

Figure S44. ^{1}H NMR spectrum of compound **16** (NPD-3488).

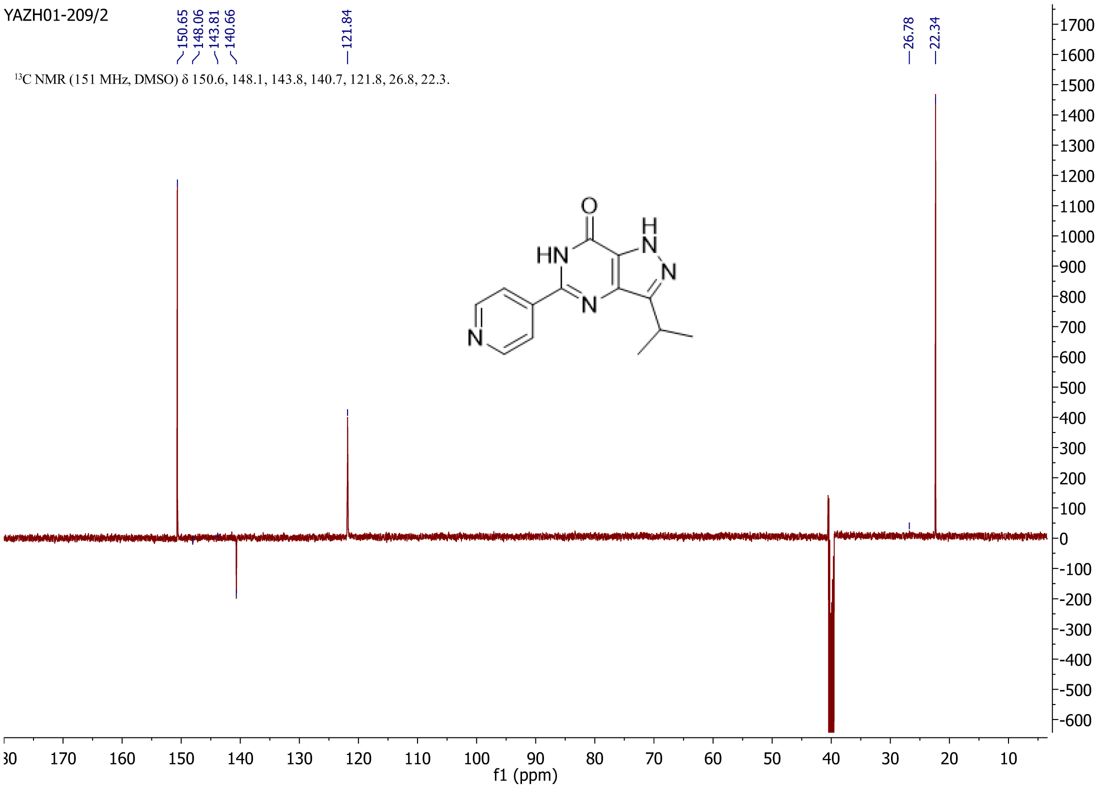

Figure S45. ^{13}C NMR spectrum of compound **16** (NPD-3488).

Acquired by	: Admin
Date Acquired	: 5/17/2016 12:43:55 PM
Sample Name	: YAZH-131
Sample ID	:
Tray#	: 1
Vial#	: 20
Injection Volume	: 1
Data File	: C:\LabSolutions\Data\2016 - wk20\YAZH-131.lcd
Background File	: blanco 17052016.lcd
Method File	: Method SCAN ACID standard.lcm
Report Format	: DefaultLCMS.lcr
Tuning File	: C:\LabSolutions\Tuning\Tuning-ESI-pos-neg01072015a.lct
Processed by	: Admin
Modified Date	: 5/17/2016 2:59:15 PM

PDA Graph

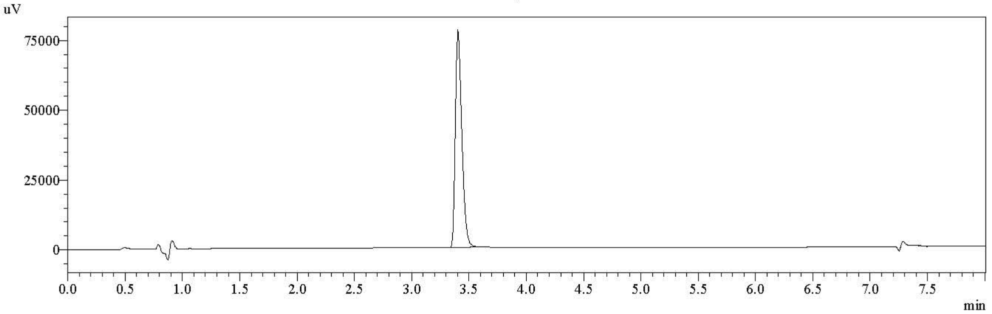

PDA Ch1 254nm 4nm

Peak#	Name	Ret. Time	Area	Area%
1		3.399	307369	100.000

MS Spectrum Graph

#:1 Ret.Time:Averaged 3.470-3.490(Scan#:348-350)
Mass Peaks:11 Base Peak:262.00(159271) Polarity:Pos Segment1 - Event1

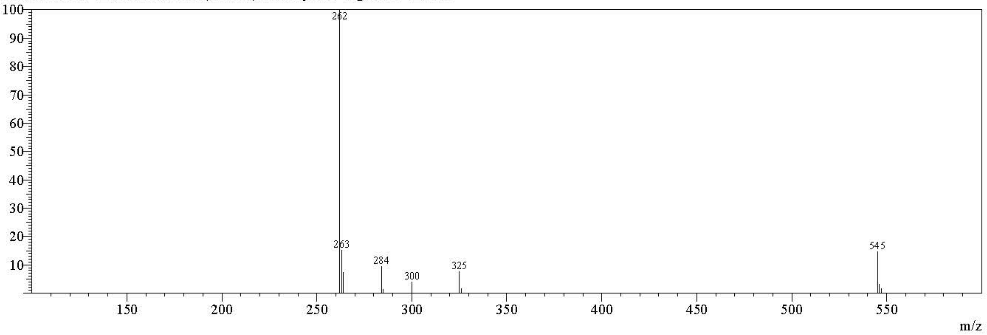

MS Spectrum Table

#:1 Ret.Time:
BG Mode:Calc 3.370<->3.640(338<->365)
Mass Peaks:11 Base Peak:262.00(159271) Polarity:Pos Segment1 - Event1

#	m/z	Abs.Inten.	Rel.Inten.	Charge	Polarity	Monoisotopic
1	262.00	159271	100.00			
2	263.05	24117	15.14			
3	264.00	11613	7.29			
4	283.95	15021	9.43			
5	285.05	2316	1.45			
6	300.05	6172	3.88			

#	m/z	Abs.Inten.	Rel.Inten.	Charge	Polarity	Monoisotopic
7	325.00	12159	7.63			
8	326.10	2444	1.53			
9	545.05	23434	14.71			
10	546.10	5129	3.22			
11	547.20	2335	1.47			

Figure S46. LCMS spectrum of compound **17** (NPD-2973).

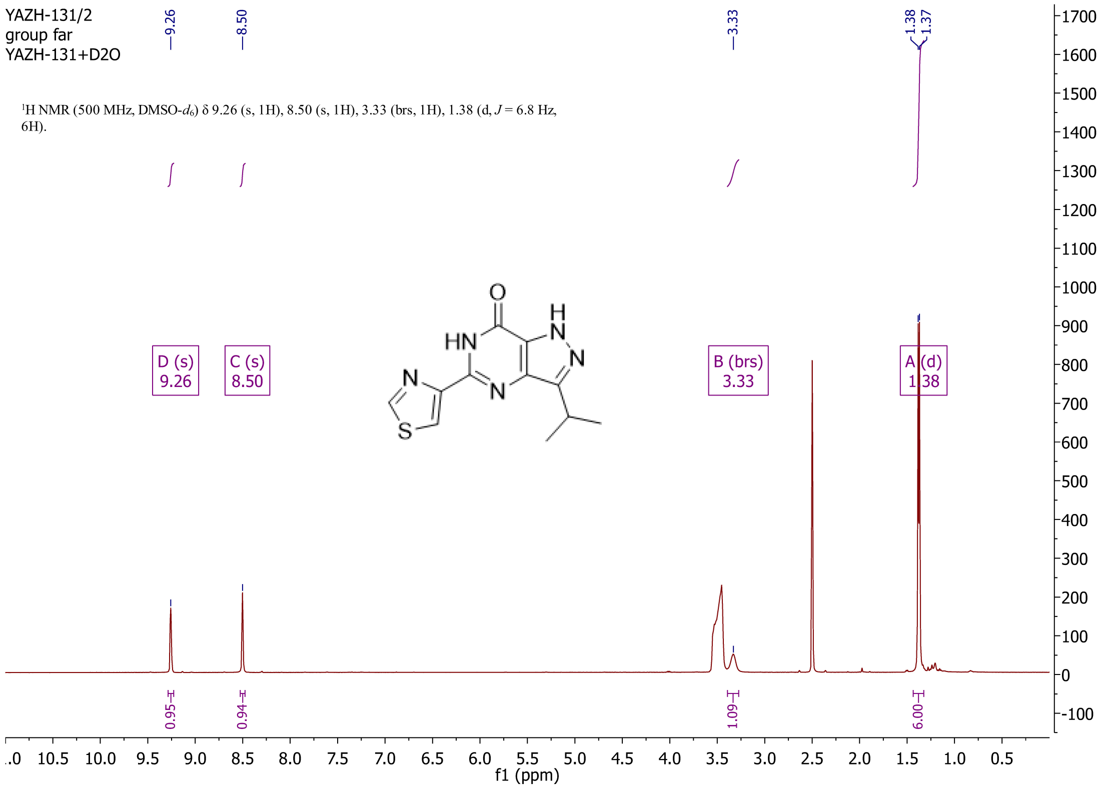

Figure S47. ^{1}H NMR spectrum of compound **17** (NPD-2973).

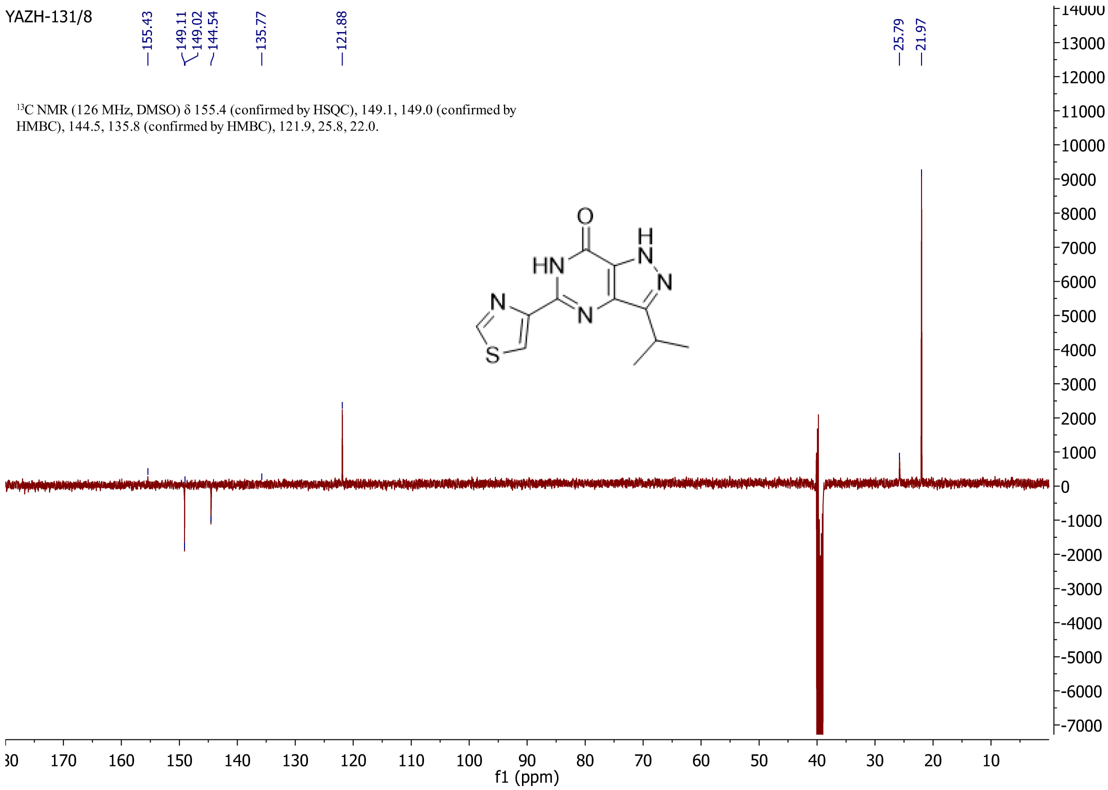

Figure S48. ^{13}C NMR spectrum of compound **17** (NPD-2973).

Acquired by	: Admin
Date Acquired	: 7/13/2017 1:11:19 PM
Sample Name	: YAZH01-115
Sample ID	:
Tray#	: 1
Vial#	: 6
Injection Volume	: 5
Data File	: C:\LabSolutions\Data\2017\2017-wk28\YAZH01-115.lcd
Background File	: blanco 13072017.lcd
Method File	: Method SCAN ACID standard.lcm
Report Format	: DefaultLCMS.lcr
Tuning File	: C:\LabSolutions\Tuning\Tuning-ESI-pos-neg01072015a.lct
Processed by	: Admin
Modified Date	: 7/13/2017 1:56:30 PM

Chromatogram
YAZH01-115 C:\LabSolutions\Data\2017\2017-wk28\YAZH01-115.lcd

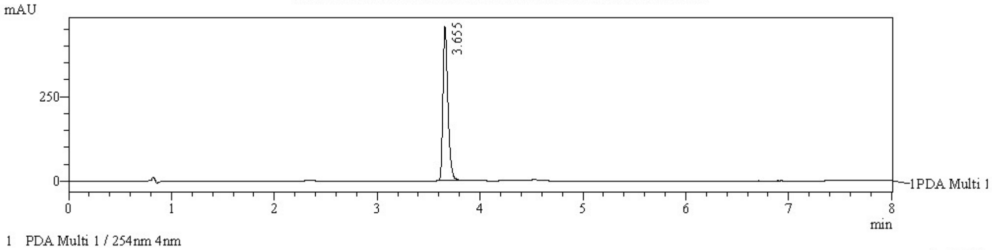

1 PDA Multi 1 / 254nm 4nm

PeakTable

PDA Ch1 254nm 4nm

Peak#	Name	Ret. Time	Area	Area %
1		3.655	1481181	100.000

MS Chromatogram
YAZH01-115 C:\LabSolutions\Data\2017\2017-wk28\YAZH01-115.lcd

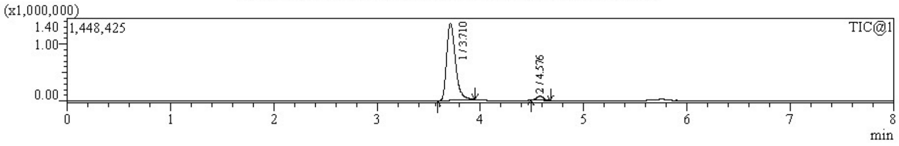

MS Spectrum Graph

#:1 Ret.Time:Averaged 3.700-3.720(Scan#:371-373)
Mass Peaks:13 Base Peak:273.10(820569) Polarity:Pos Segment1 - Event1

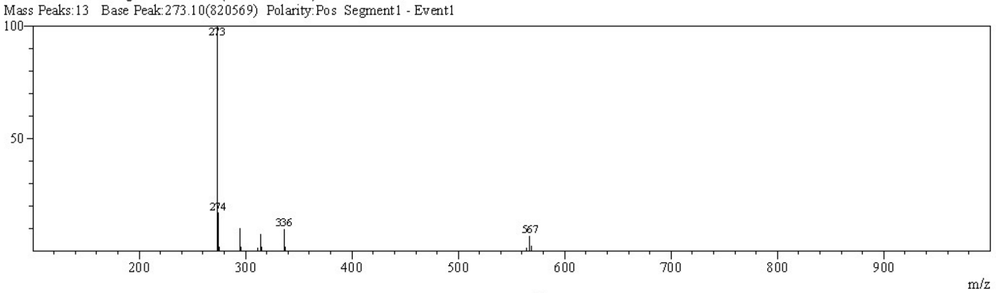

MS Spectrum Table

#:1 Ret.Time:
BG Mode:Calc 3.590<->3.950(360<->396)
Mass Peaks:13 Base Peak:273.10(820569) Polarity:Pos Segment1 - Event1

#	m/z	Abs.Inten.	Rel.Inten.	Charge	Polarity	Monoisotopic
1	273.10	820569	100.00			
2	274.10	140608	17.14			
3	275.10	15194	1.85			
4	295.05	81492	9.93			
5	296.10	13743	1.67			
6	311.00	10039	1.22			
7	314.10	62866	7.66			
8	315.10	13856	1.69			
9	336.10	80666	9.83			
10	337.10	15321	1.87			
11	564.45	11126	1.36			
12	567.25	55571	6.77			
13	568.30	20296	2.47			

Figure S49. LCMS spectrum of compound **18** (NPD-3199).

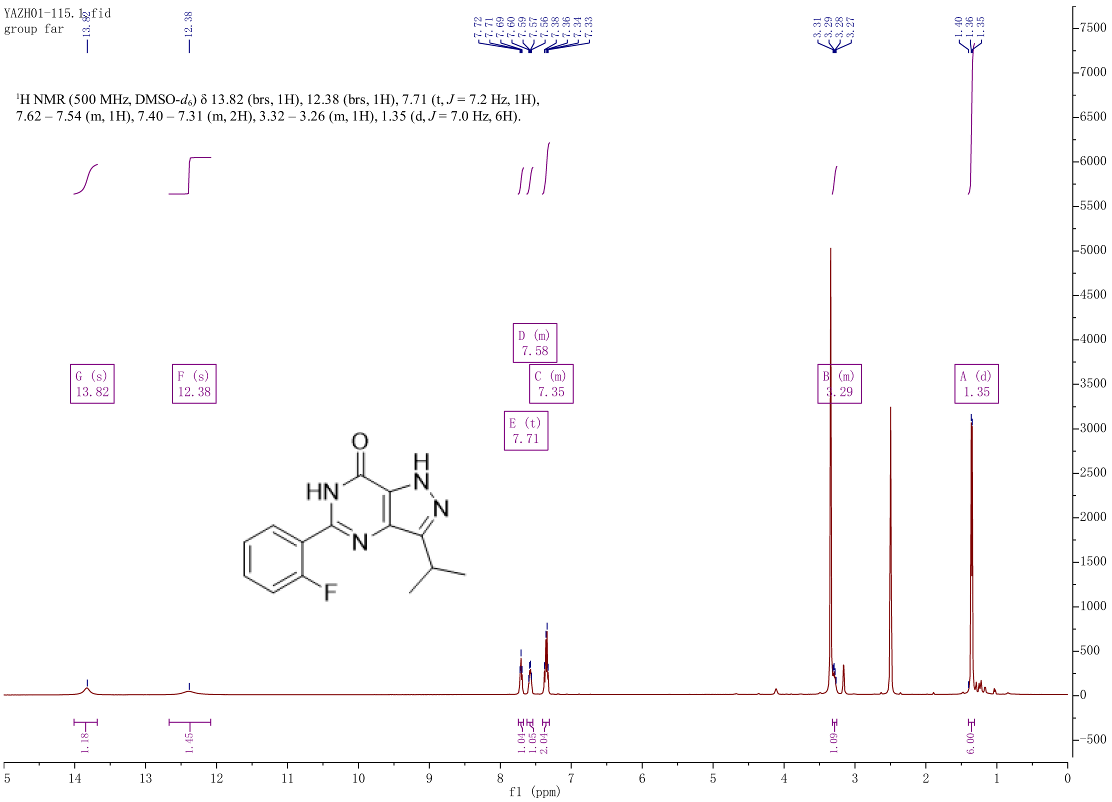

Figure S50. ^{1}H NMR spectrum of compound **18** (NPD-3199).

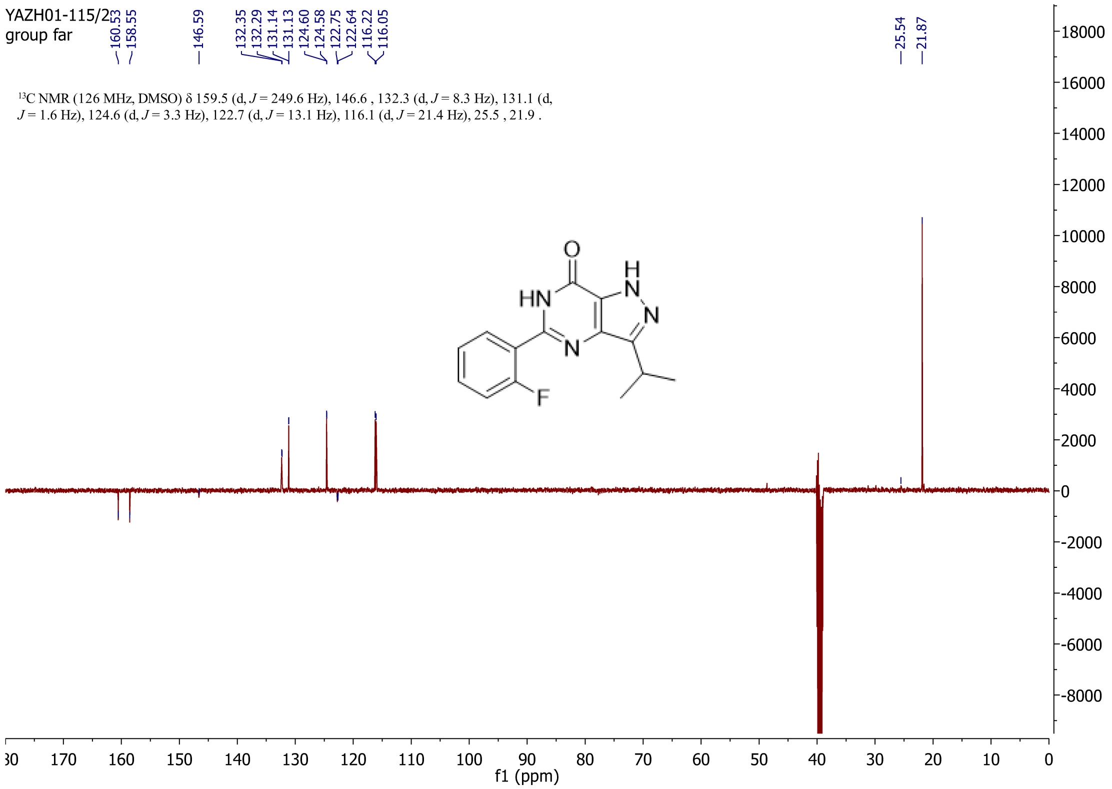

Figure S51. ^{13}C NMR spectrum of compound **18** (NPD-3199).

Acquired by : Admin
Date Acquired : 20/12/2018 3:44:02 PM
Sample Name : ODVI-002
Sample ID :
Tray# : 1
Vial# : 8
Injection Volume : 5
Data File : C:\LabSolutions\Data\2018\2018-wk51\ODVI-002.lcd
Background File : blanco 20122018.lcd
Method File : Method SCAN ACID standard.lcm
Report Format : DefaultLCMS.lcr
Tuning File : C:\LabSolutions\Tuning\Tuning-ESI-pos-neg01072015a.lct
Processed by : Admin
Modified Date : 20/12/2018 3:52:29 PM

Chromatogram
ODVI-002 C:\LabSolutions\Data\2018\2018-wk51\ODVI-002.lcd

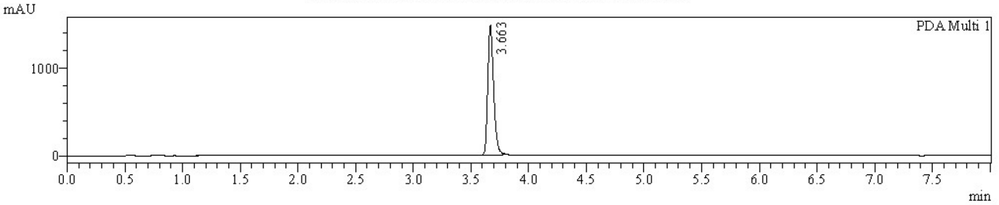

1 PDA Multi 1 / 254nm 4nm

PeakTabl

PDA Ch1 254nm 4nm

Peak#	Name	Ret. Time	Area	Area %
1		3.663	5181695	100.000

MS Chromatogram
ODVI-002 C:\LabSolutions\Data\2018\2018-wk51\ODVI-002.lcd

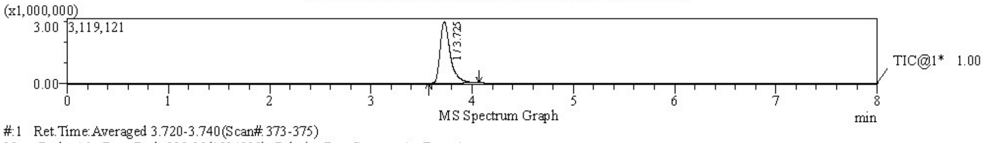

MS Spectrum Graph

#:1 Ret.Time:Averaged 3.720-3.740(Scan#:373-375)
Mass Peaks:16 Base Peak:289.05(1524922) Polarity:Pos Segment1 - Event1

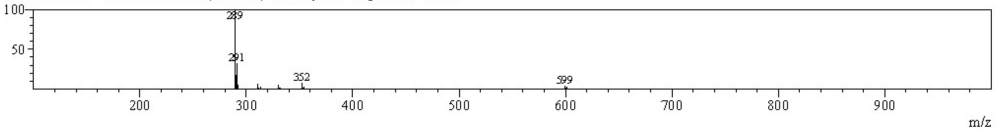

MS Spectrum Table

#:1 Ret.Time:
BG Mode:Calc 3.570<->4.070(358<->408)
Mass Peaks:16 Base Peak:289.05(1524922) Polarity:Pos Segment1 - Event1

#	m/z	Abs.Inten.	Rel.Inten.	Charge	Polarity	Monoisotopic
1	289.05	1524922	100.00			
2	290.05	260433	17.08			
3	291.00	481508	31.58			
4	292.00	75350	4.94			
5	311.00	90213	5.92			
6	312.05	15563	1.02			
7	313.05	36115	2.37			
8	330.05	75293	4.94			
9	331.05	16309	1.07			
10	332.10	25098	1.65			
11	352.05	119175	7.82			
12	353.05	21959	1.44			
13	354.10	42185	2.77			
14	599.15	58059	3.81			
15	600.15	22825	1.50			
16	601.15	46128	3.02			

Figure S52. LCMS spectrum of compound **19** (NPD-3538).

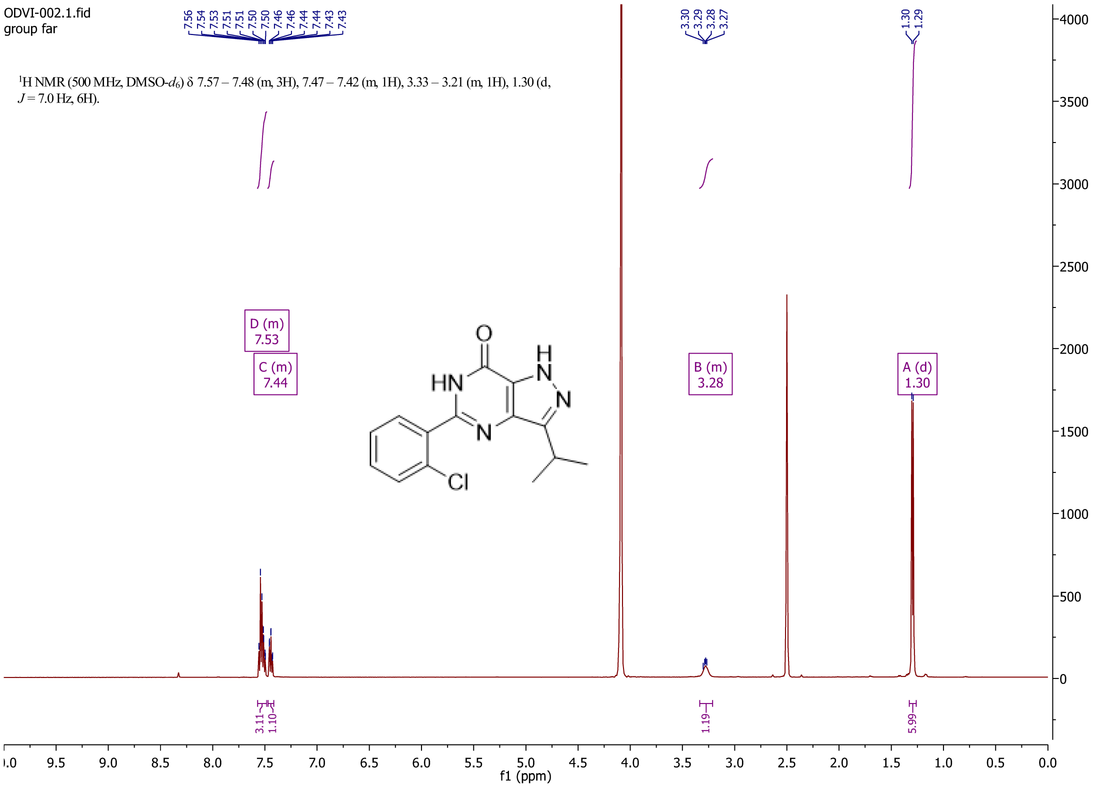

Figure S53. ^{1}H NMR spectrum of compound **19** (NPD-3538).

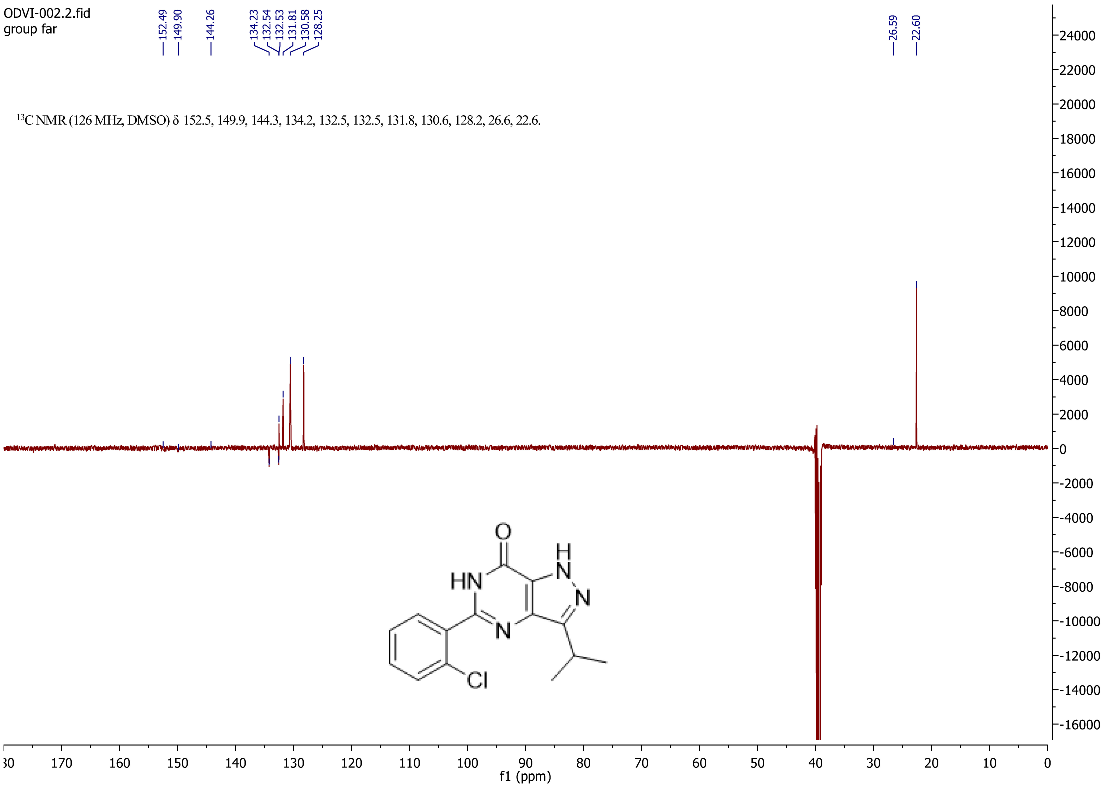

Figure S54. ^{13}C NMR spectrum of compound **19** (NPD-3538).

Acquired by	: Admin
Date Acquired	: 15/11/2018 9:42:38 AM
Sample Name	: ODVI01_037 Pure 15112018
Sample ID	:
Tray#	: 1
Vial#	: 1
Injection Volume	: 1
Data File	: C:\LabSolutions\Data\2018\2018-wk46\ODVI01_037 Pure 15112018.lcd
Background File	: blanco_151118.lcd
Method File	: Method SCAN ACID standard.lcm
Report Format	: DefaultLCMS.lcr
Tuning File	: C:\LabSolutions\Tuning\Tuning-ESI-pos-neg01072015a.lct
Processed by	: Admin
Modified Date	: 15/11/2018 9:58:15 AM

Chromatogram
ODVI01_037 Pure 15112018 C:\LabSolutions\Data\2018\2018-wk46\ODVI01_037 Pure 15112018.lcd

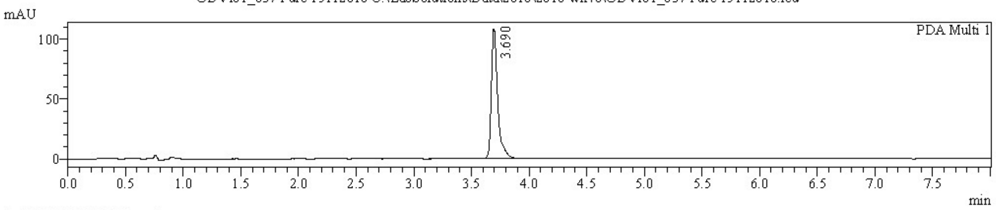

1 PDA Multi 1 / 254nm 4nm

Peak#	Name	Ret. Time	Area	Area %
1		3.690	410572	100.000

MS Chromatogram
ODVI01_037 Pure 15112018 C:\LabSolutions\Data\2018\2018-wk46\ODVI01_037 Pure 15112018.lcd

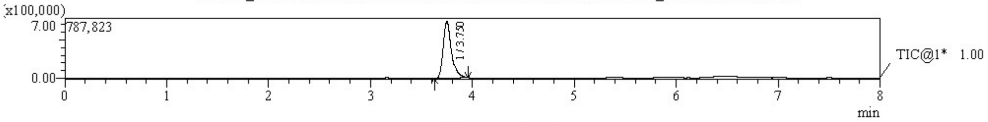

MS Spectrum Graph
#:1 Ret.Time:Averaged 3.740-3.760(Scan#:375-377)
Mass Peaks:22 Base Peak:335.00(280581) Polarity:Pos Segment1 - Event1

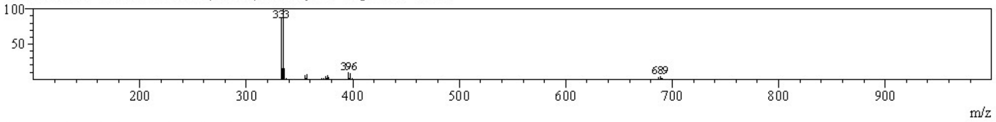

#:2 Ret.Time:Single 3.930(Scan#:394)
Mass Peaks:4 Base Peak:332.95(4071) Polarity:Pos Segment1 - Event1

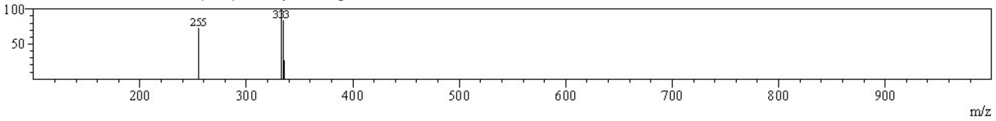

MS Spectrum Table
#:1 Ret.Time:
BG Mode:Calc 3.630<->3.960(364<->397)
Mass Peaks:22 Base Peak:335.00(280581) Polarity:Pos Segment1 - Event1

#	m/z	Abs.Inten.	Rel.Inten.	Charge	Polarity	Monoisotopic	#	m/z	Abs.Inten.	Rel.Inten.	Charge	Polarity	Monoisotopic
1	333.00	245343	87.44				5	337.15	3821	1.36			
2	334.00	43466	15.49				6	354.95	14857	5.30			
3	335.00	280581	100.00				7	356.05	5018	1.79			
4	336.00	42171	15.03				8	357.00	18710	6.67			

Figure S55. LCMS spectrum of compound **20** (NPD-3539).

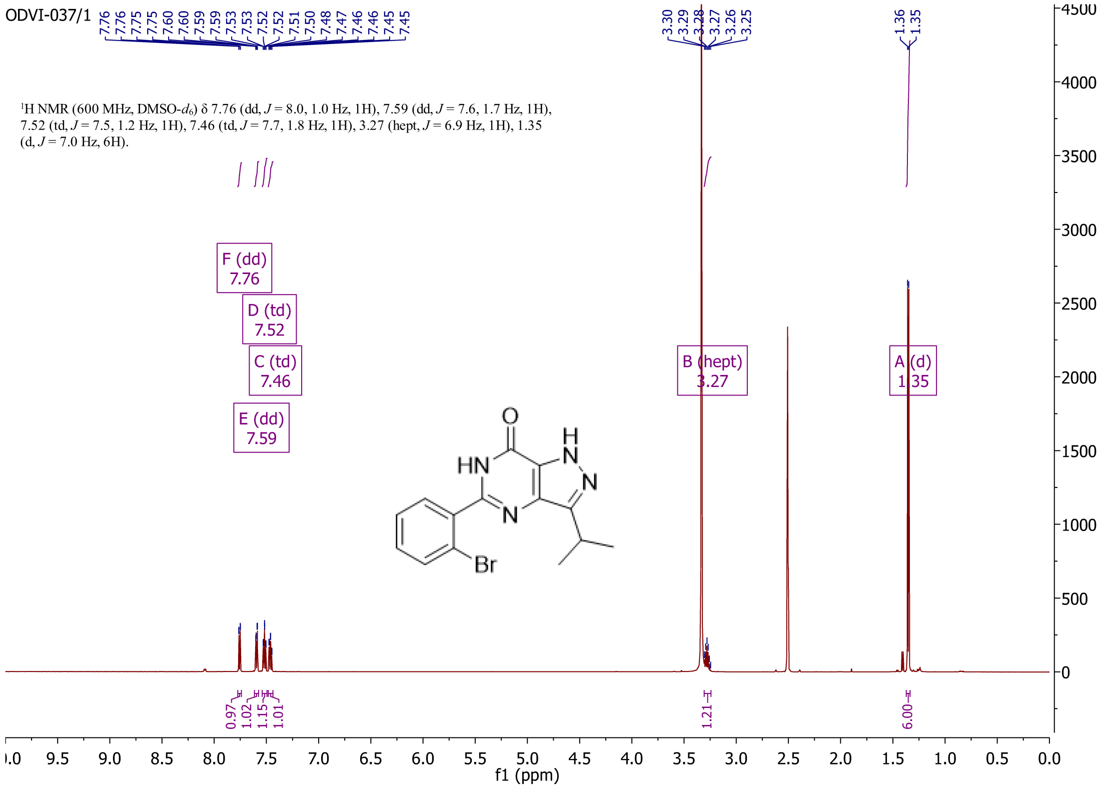

Figure S56. ^{1}H NMR spectrum of compound **20** (NPD-3539).

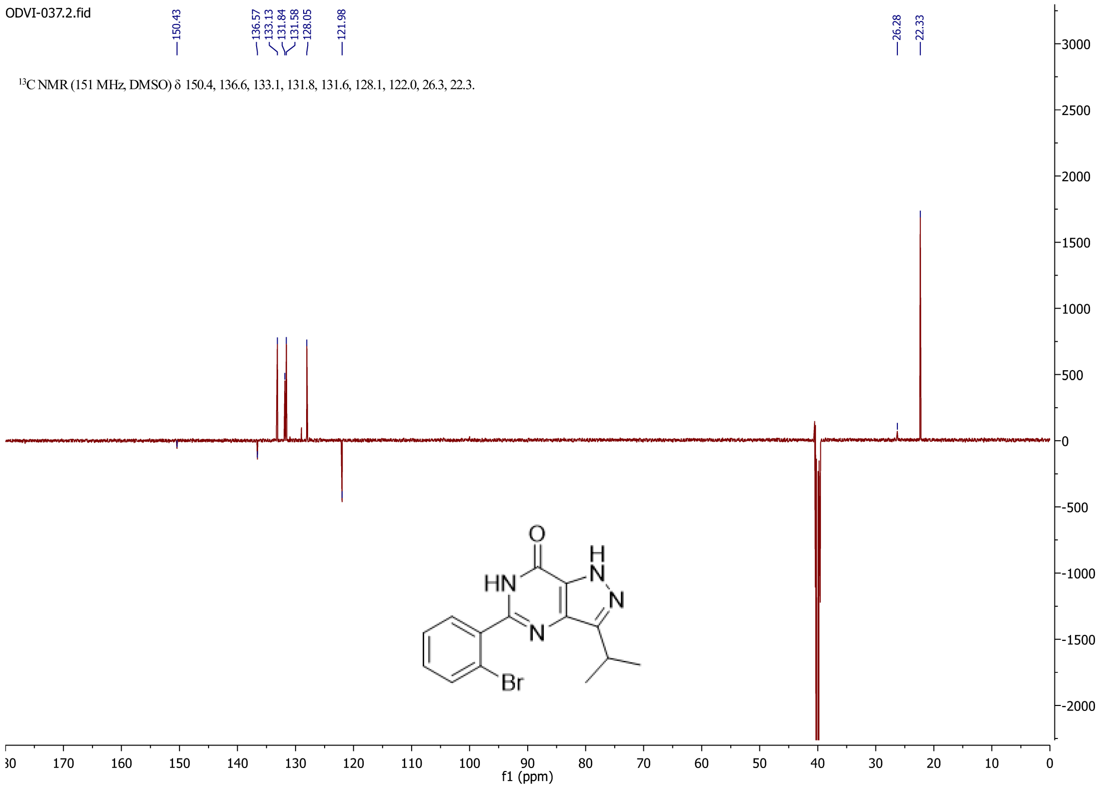

Figure S57. ^{13}C NMR spectrum of compound **20** (NPD-3539).

Acquired by : Admin
Date Acquired : 3/7/2019 12:46:37 PM
Sample Name : VUF16899
Sample ID :
Tray# : 1
Vial# : 17
Injection Volume : 1
Data File : C:\LabSolutions\Data\2019\2019-wk27\VUF16899.lcd
Background File : blanco 03072019.lcd
Method File : Method SCAN ACID standard.lcm
Report Format : DefaultLCMS.lcr
Tuning File : C:\LabSolutions\Tuning\Tuning-ESI-pos-neg01072015a.lct
Processed by : Admin
Modified Date : 3/7/2019 1:01:11 PM

Chromatogram
VUF16899 C:\LabSolutions\Data\2019\2019-wk27\VUF16899.lcd

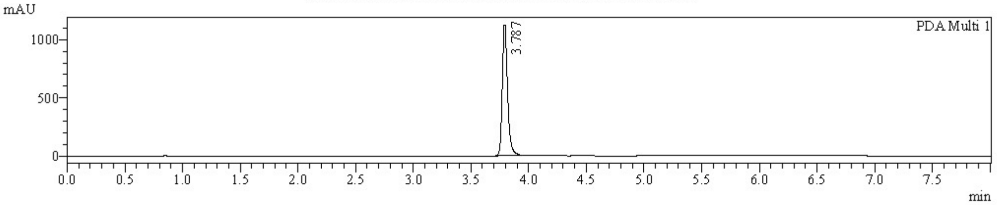

1 PDA Multi 1 / 254nm 4nm

PeakTabl

PDA Ch1 254nm 4nm

Peak#	Name	Ret. Time	Area	Area %
1		3.787	3569842	100.000

MS Chromatogram
VUF16899 C:\LabSolutions\Data\2019\2019-wk27\VUF16899.lcd

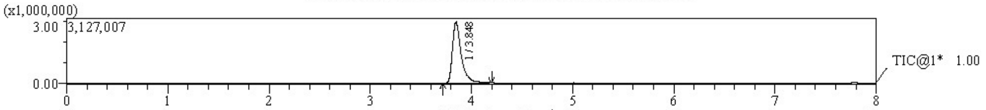

MS Spectrum Graph

#:1 Ret.Time:Averaged 3.840-3.860(Scan#:385-387)
Mass Peaks:10 Base Peak:269.05(2106094) Polarity:Pos Segment1 - Event1

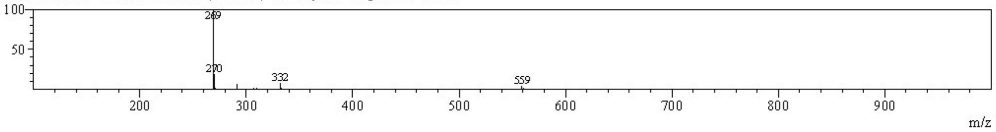

MS Spectrum Table

#:1 Ret.Time:
BG Mode:Calc 3.720<->4.200(373<->421)
Mass Peaks:10 Base Peak:269.05(2106094) Polarity:Pos Segment1 - Event1

#	m/z	Abs.Inten.	Rel.Inten.	Charge	Polarity	Monoisotopic	#	m/z	Abs.Inten.	Rel.Inten.	Charge	Polarity	Monoisotopic
1	269.05	2106094	100.00				6	310.05	36373	1.73			
2	270.05	377771	17.94				7	332.10	151953	7.21			
3	271.05	35728	1.70				8	333.10	30653	1.46			
4	291.05	138338	6.57				9	559.25	70066	3.33			
5	307.05	23669	1.12				10	560.20	23844	1.13			

Figure. S58 LCMS spectrum of compound **21** (NPD-3589).

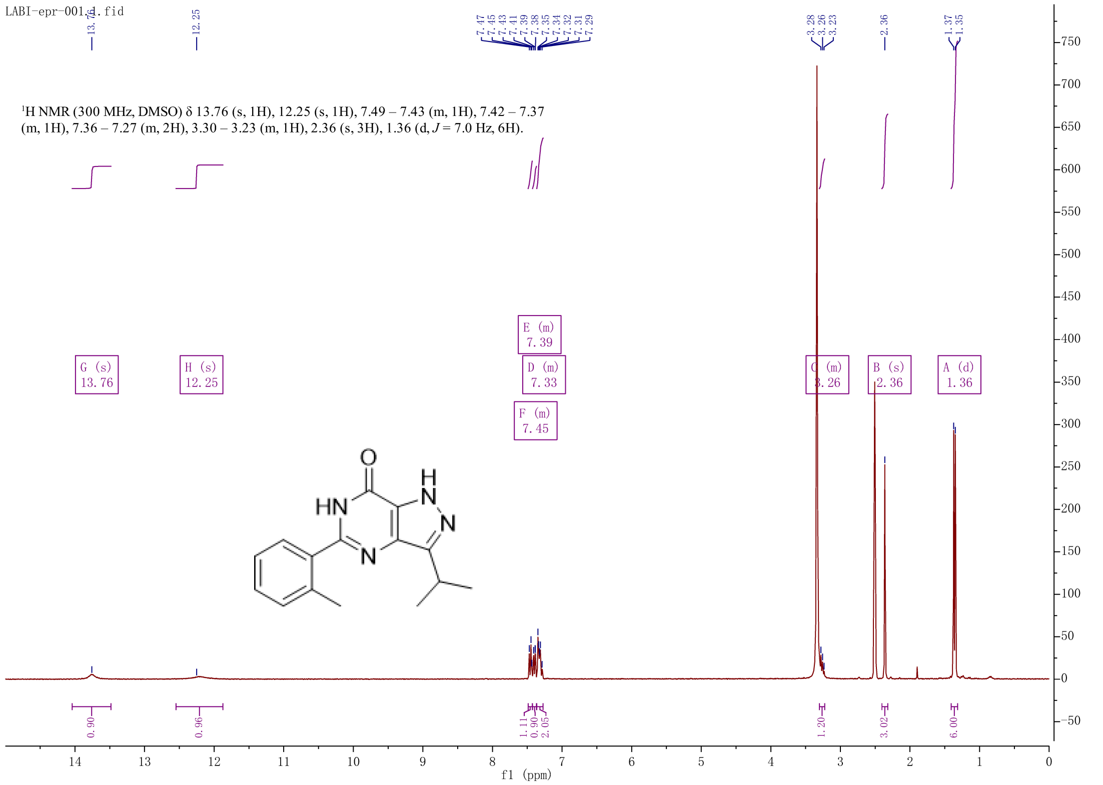

Figure S59. ^{1}H NMR spectrum of compound **21** (NPD-3589).

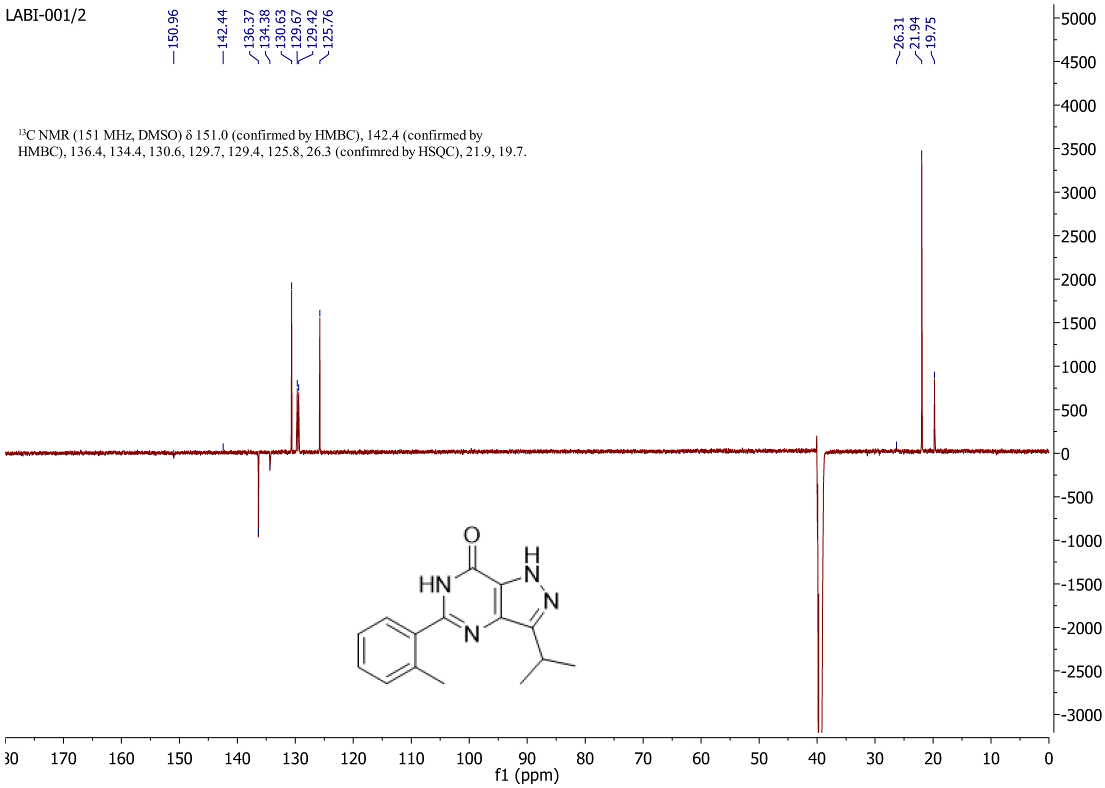

Figure S60. ^{13}C NMR spectrum of compound **21** (NPD-3589).

Acquired by : Admin
Date Acquired : 3/12/2018 3:17:33 PM
Sample Name : LABI-FINALPR002
Sample ID :
Tray# : 1
Vial# : 51
Injection Volume : 5
Data File : C:\LabSolutions\Data\2018\2018-wk49\LABI-FINALPR002.lcd
Background File : blanco 03122018.lcd
Method File : Method SCAN ACID standard.lcm
Report Format : DefaultLCMS.lcr
Tuning File : C:\LabSolutions\Tuning\Tuning-ESI-pos-neg01072015a.lct
Processed by : Admin
Modified Date : 3/12/2018 3:27:49 PM

Chromatogram
LABI-FINALPR002 C:\LabSolutions\Data\2018\2018-wk49\LABI-FINALPR002.lcd

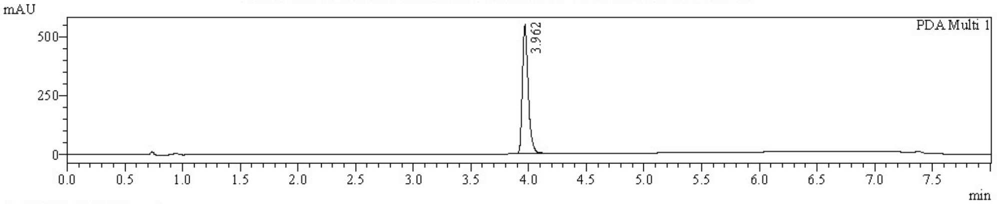

1 PDA Multi 1 / 254nm 4nm

PeakTable

PDA Ch1 254nm 4nm

Peak#	Name	Ret. Time	Area	Area %
1		3.962	1898026	100.000

MS Chromatogram
LABI-FINALPR002 C:\LabSolutions\Data\2018\2018-wk49\LABI-FINALPR002.lcd

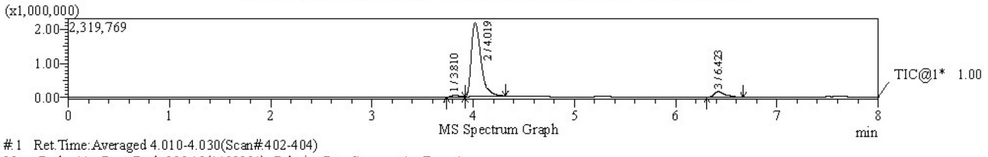

MS Spectrum Graph

#1 Ret.Time:Averaged 4.010-4.030(Scan#:402-404)
Mass Peaks:11 Base Peak:285.10(1408931) Polarity:Pos Segment1 - Event1

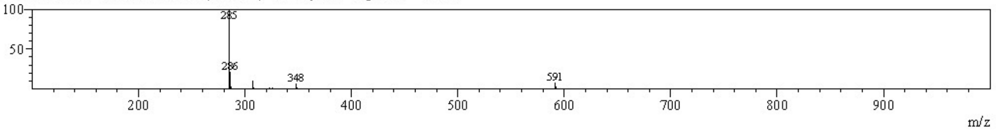

MS Spectrum Table

#1 Ret.Time:
BG Mode:Calc 3.920<->4.320(393<->433)
Mass Peaks:11 Base Peak:285.10(1408931) Polarity:Pos Segment1 - Event1

#	m/z	Abs.Inten.	Rel.Inten.	Charge	Polarity	Monoisotopic
1	285.10	1408931	100.00			
2	286.10	292161	20.74			
3	287.05	27635	1.96			
4	307.05	139653	9.91			
5	308.10	18033	1.28			
6	323.05	14162	1.01			
7	326.15	18220	1.29			
8	348.05	86367	6.13			
9	349.05	17684	1.26			
10	591.20	105593	7.49			
11	592.20	36837	2.61			

Figure S61. LCMS spectrum of compound **22** (NPD-3590).

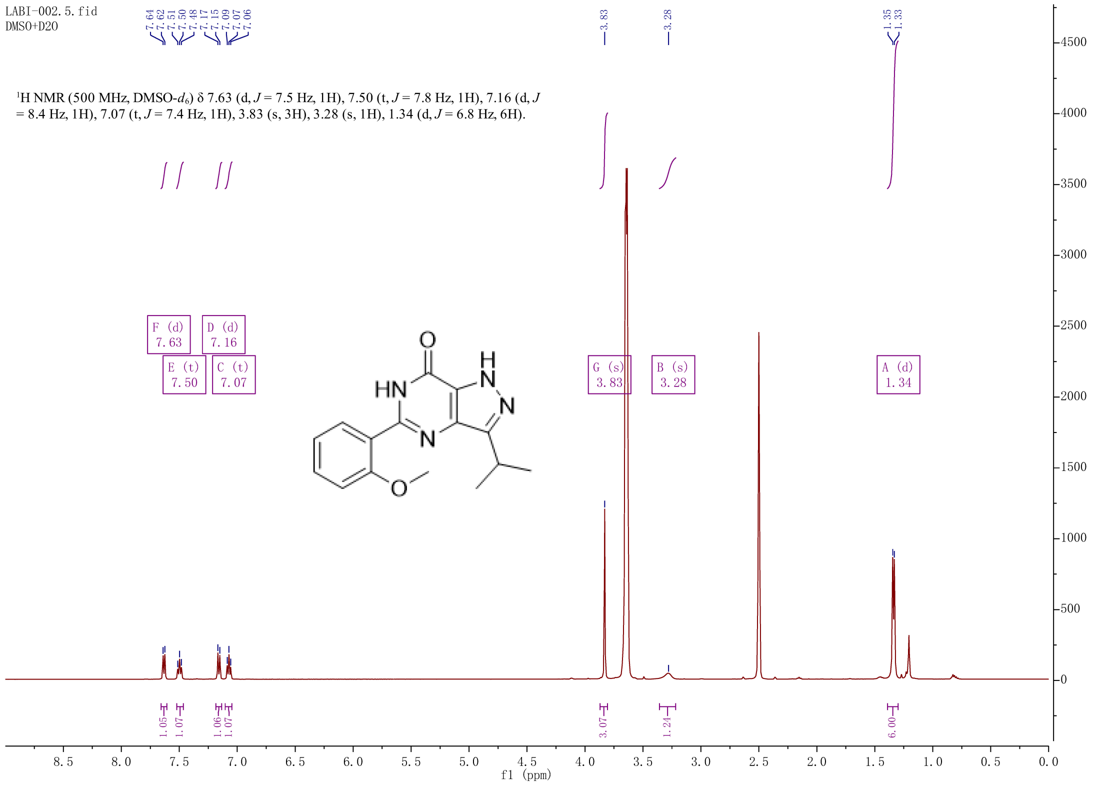

Figure S62. ^{1}H NMR spectrum of compound **22** (NPD-3590).

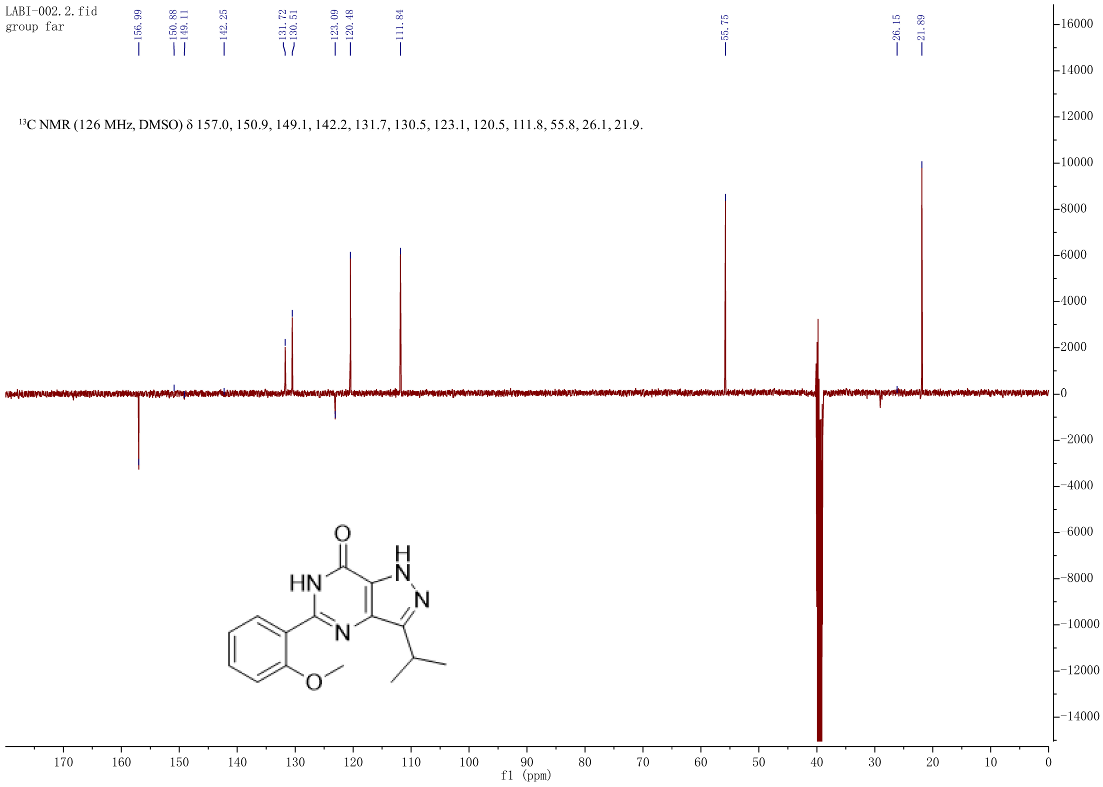

Figure S63. ^{13}C NMR spectrum of compound **22** (NPD-3590).

Acquired by	: Admin
Date Acquired	: 18/6/2019 3:31:13 PM
Sample Name	: VUF16256
Sample ID	:
Tray#	: 1
Vial#	: 34
Injection Volume	: 5
Data File	: C:\LabSolutions\Data\2019\2019-wk25\VUF16256.lcd
Background File	: Blanco_17062019.lcd
Method File	: Method SCAN ACID standard.lcm
Report Format	: DefaultLCMS.lcr
Tuning File	: C:\LabSolutions\Tuning\Tuning-ESI-pos-neg01072015a.lct
Processed by	: Admin
Modified Date	: 18/6/2019 3:50:24 PM

PDA Graph

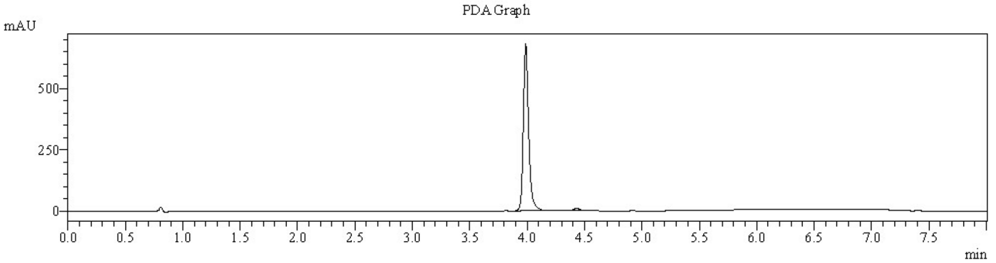

PeakTable

PDA Ch1 254nm 4nm

Peak#	Name	Ret. Time	Area	Area %
1		3.982	2151511	98.367
2		4.424	35727	1.633

MS Chromatogram
VUF16256 C:\LabSolutions\Data\2019\2019-wk25\VUF16256.lcd

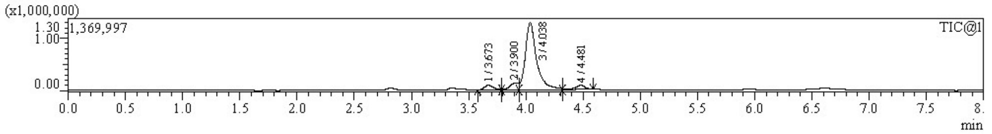

MS Spectrum Graph

#1 Ret.Time:Averaged 4.030-4.050(Scan#:404-406)
BG Mode:Calc 3.940<->4.320(395<->433)
Mass Peaks:16 Base Peak:273.10(656676) Polarity:Pos Segment1 - Event1

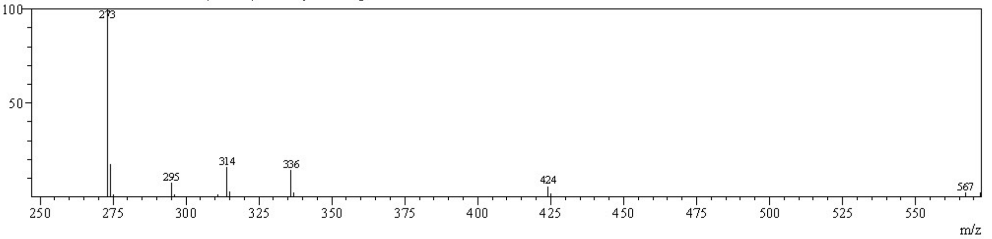

MS Spectrum Table

#:1 Ret.Time:
BG Mode:Calc 3.940<->4.320(395<->433)
Mass Peaks:16 Base Peak:273.10(656676) Polarity:Pos Segment1 - Event1

#	m/z	Abs.Inten.	Rel.Inten.	Charge	Polarity	Monoisotopic
1	247.05	36967	5.63			
2	273.10	656676	100.00			
3	274.10	112147	17.08			
4	275.05	8639	1.32			
5	295.05	49024	7.47			
6	296.05	7690	1.17			
7	311.05	7815	1.19			
8	314.10	102327	15.58			

#	m/z	Abs.Inten.	Rel.Inten.	Charge	Polarity	Monoisotopic
9	315.05	19551	2.98			
10	336.05	91548	13.94			
11	337.05	15753	2.40			
12	424.15	36360	5.54			
13	425.15	9337	1.42			
14	567.20	15113	2.30			
15	572.20	15073	2.30			
16	572.65	22697	3.46			

Figure S64. LCMS spectrum of compound **23** (NPD-3202).

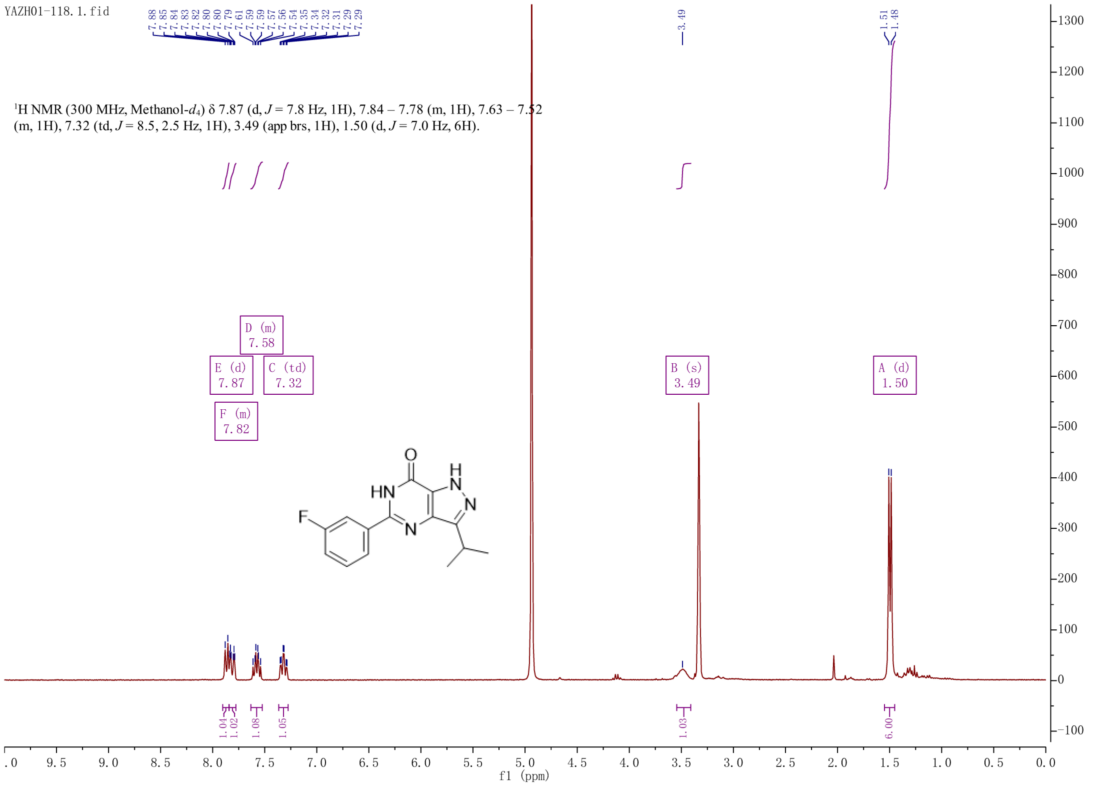

Figure S65. ¹H NMR spectrum of compound **23** (NPD-3202).

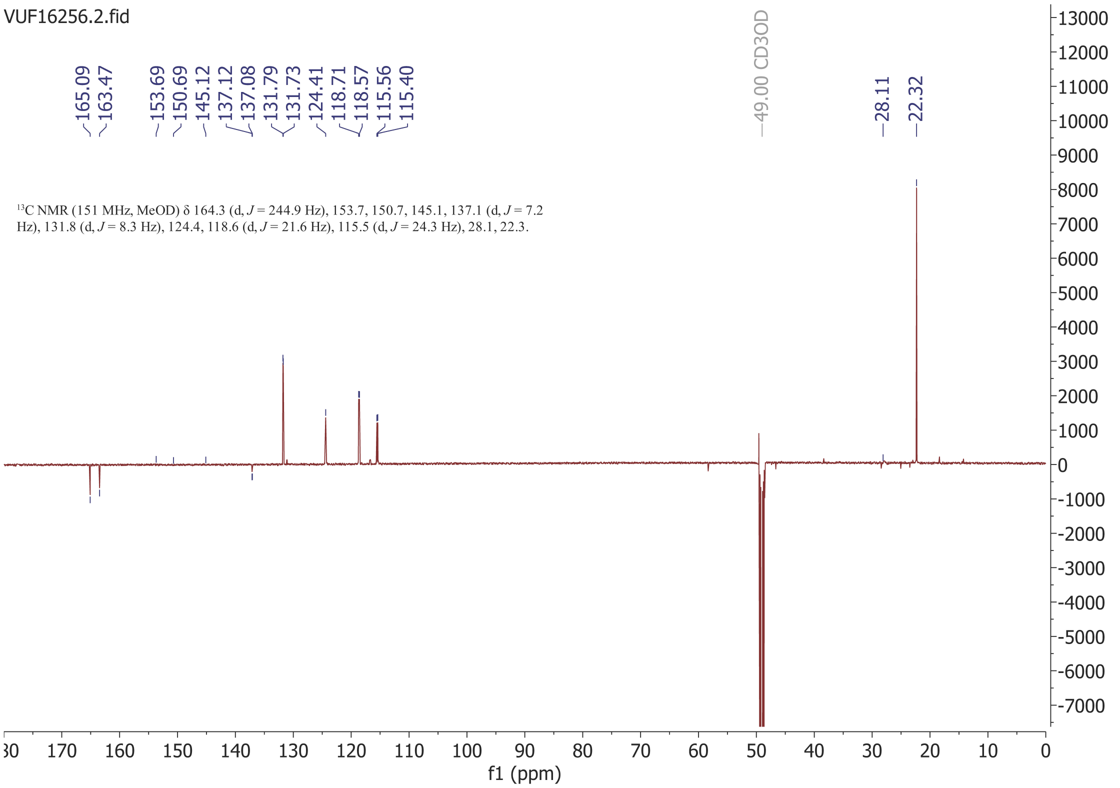

Figure S66. ¹³C NMR spectrum of compound **23** (NPD-3202).

Acquired by	: Admin
Date Acquired	: 21/11/2018 9:39:12 AM
Sample Name	: LABI-EPR-003
Sample ID	:
Tray#	: 1
Vial#	: 3
Injection Volume	: 1
Data File	: C:\LabSolutions\Data\2018\2018-wk47\LABI-EPR-003.lcd
Background File	: Blanco 21112018.lcd
Method File	: Method SCAN ACID standard.lcm
Report Format	: DefaultLCMS.lcr
Tuning File	: C:\LabSolutions\Tuning\Tuning-ESI-pos-neg01072015a.lct
Processed by	: Admin
Modified Date	: 2/12/2020 3:21:13 PM

PDA Graph

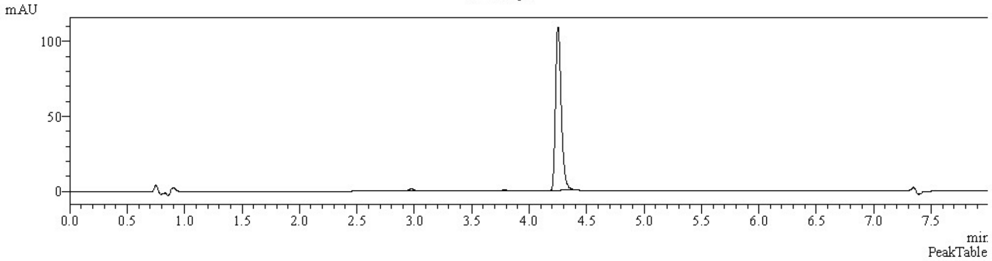

PeakTable

PDA Ch1 254nm 4nm

Peak#	Name	Ret. Time	Area	Area %
1		2.966	3475	0.901
2		3.783	713	0.185
3		4.245	381408	98.914

MS Chromatogram
LABI-EPR-003 C:\LabSolutions\Data\2018\2018-wk41-51\2018-wk47\LABI-EPR-003.lcd

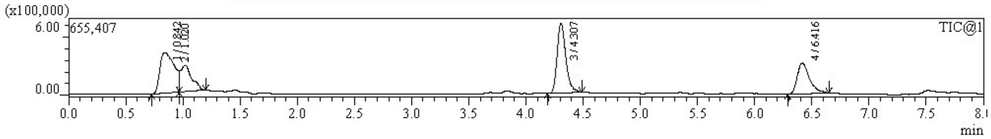

MS Spectrum Graph

#1 Ret.Time:Averaged 4.300-4.320(Scan#:431-433)
BG Mode:Calc 4.190<->4.490(420<->450)
Mass Peaks:18 Base Peak:289.05(269467) Polarity:Pos Segment1 - Event1

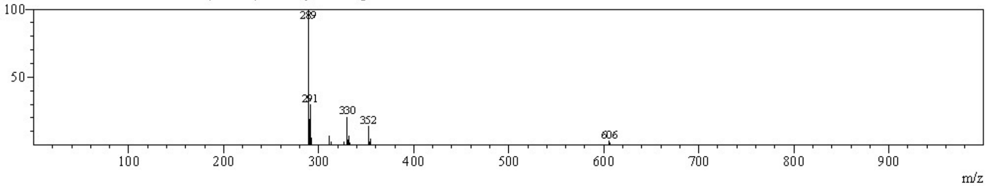

MS Spectrum Table

#:1 Ret.Time:
BG Mode:Calc 4.190<->4.490(420<->450)
Mass Peaks:18 Base Peak:289.05(269467) Polarity:Pos Segment1 - Event1

#	m/z	Abs.Inten.	Rel.Inten.	Charge	Polarity	Monoisotopic	#	m/z	Abs.Inten.	Rel.Inten.	Charge	Polarity	Monoisotopic
1	289.05	269467	100.00				10	331.10	10065	3.74			
2	290.00	49867	18.51				11	332.10	18171	6.74			
3	291.10	79825	29.62				12	333.10	4125	1.53			
4	292.10	14178	5.26				13	352.10	37789	14.02			
5	311.05	17517	6.50				14	353.25	5742	2.13			
6	313.05	5487	2.04				15	354.05	12482	4.63			
7	327.10	5969	2.22				16	605.15	5778	2.14			
8	329.25	2972	1.10				17	606.00	7511	2.79			
9	330.10	55307	20.52				18	606.95	3608	1.34			

Figure S67. LCMS spectrum of compound **24** (NPD-3591).

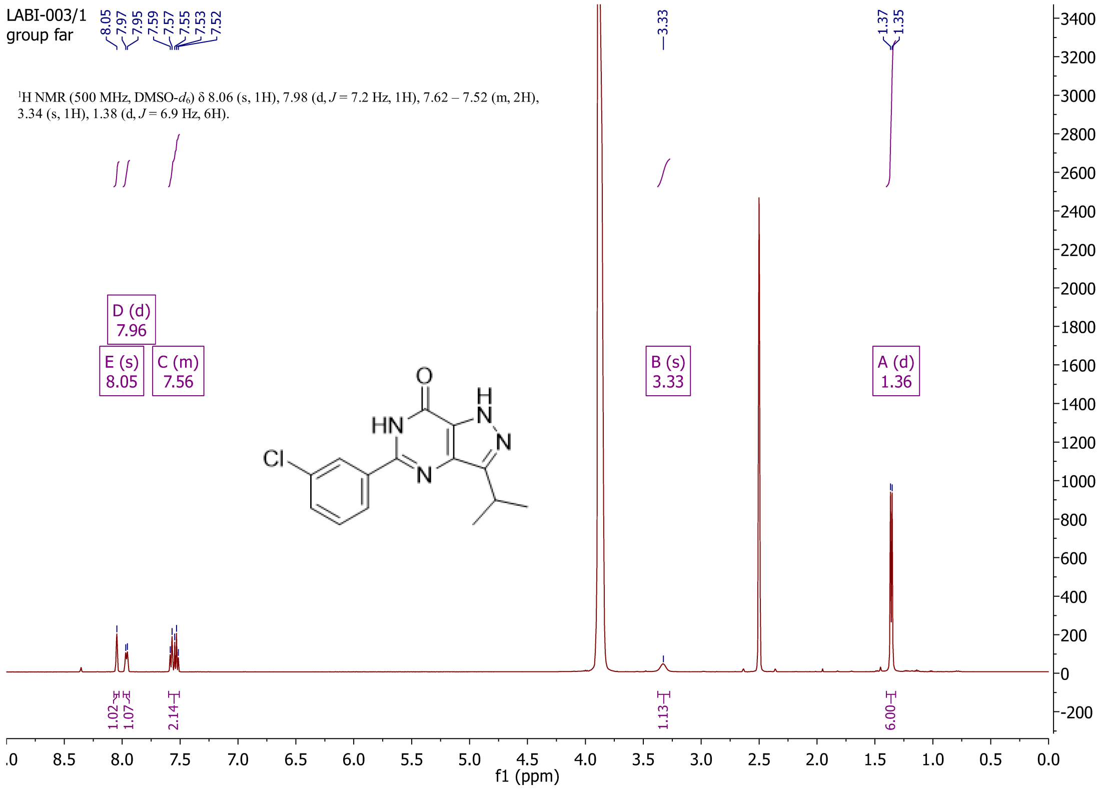

Figure S68. ¹H NMR spectrum of compound **24** (NPD-3591).

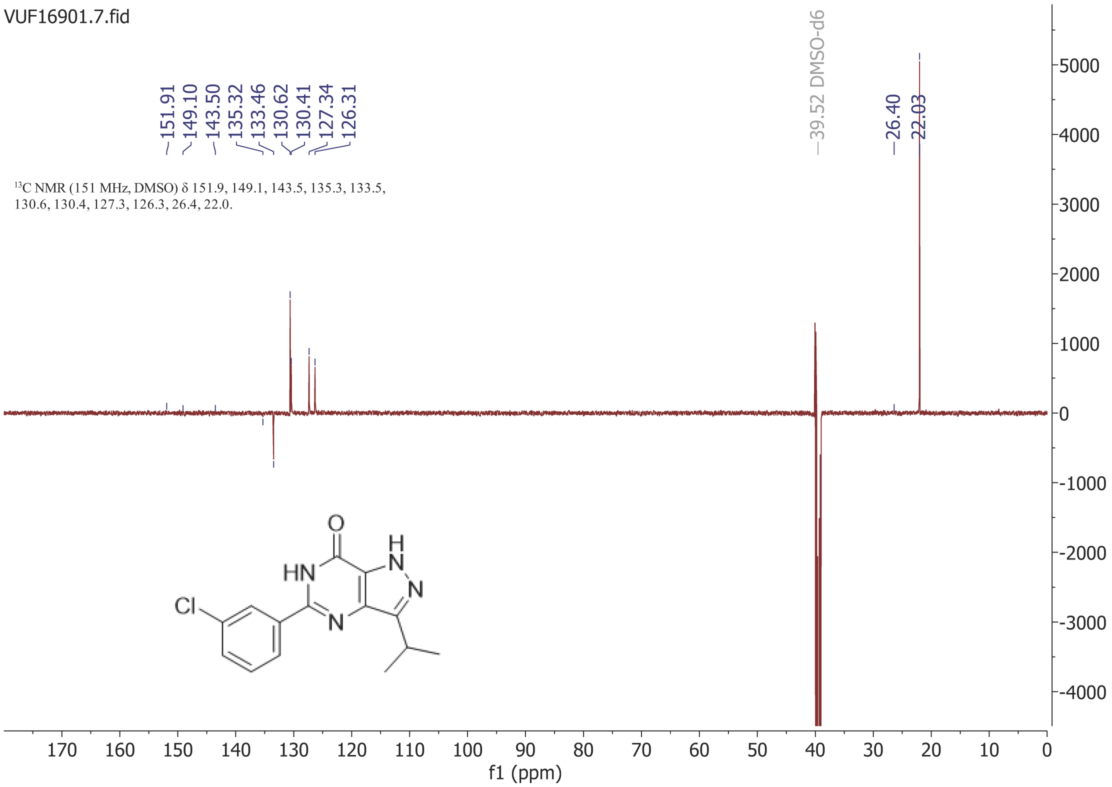

Figure S69. ¹³C NMR spectrum of compound **24** (NPD-3591).

Acquired by : Admin
Date Acquired : 25/4/2018 2:06:52 PM
Sample Name : YAZH01-206
Sample ID :
Tray# : 1
Vial# : 22
Injection Volume : 3
Data File : C:\LabSolutions\Data\2018\2018-wk17\YAZH01-206.lcd
Background File : blanco 25042018.lcd
Method File : Method SCAN ACID standard.lcm
Report Format : DefaultLCMS.lcr
Tuning File : C:\LabSolutions\Tuning\Tuning-ESI-pos-neg01072015a.lct
Processed by : Admin
Modified Date : 25/4/2018 3:20:15 PM

PDA Graph

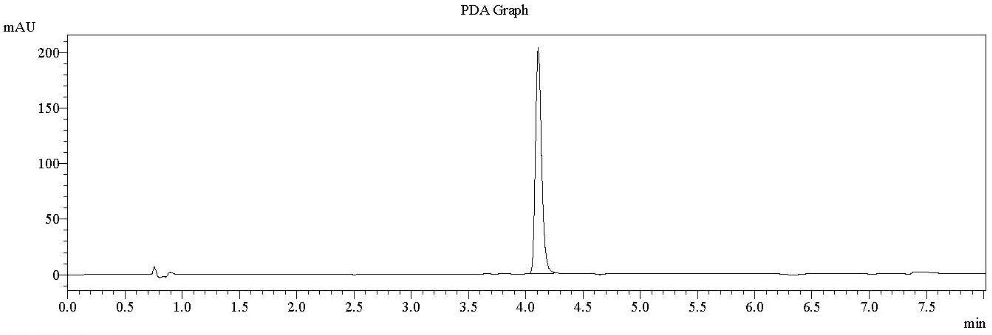

PDA Ch1 254nm 4nm

Peak#	Name	Ret. Time	Area	Area%
1		4.104	758929	100.000

MS Spectrum Graph

#:1 Ret.Time:Averaged 4.150-4.170(Scan#:416-418)
Mass Peaks:10 Base Peak:269.10(631781) Polarity:Pos Segment1 - Event1

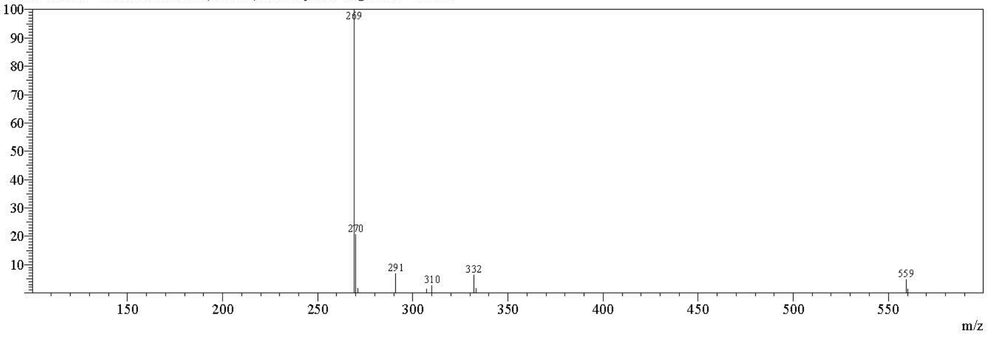

MS Spectrum Table

#:1 Ret.Time:
BG Mode:Calc 4.040<->4.370(405<->438)
Mass Peaks:10 Base Peak:269.10(631781) Polarity:Pos Segment1 - Event1

#	m/z	Abs.Inten.	Rel.Inten.	Charge	Polarity	Monoisotopic	#	m/z	Abs.Inten.	Rel.Inten.	Charge	Polarity	Monoisotopic
1	269.10	631781	100.00				6	310.10	16452	2.60			
2	270.00	129930	20.57				7	332.10	39970	6.33			
3	271.10	9945	1.57				8	333.10	9372	1.48			
4	291.05	43754	6.93				9	559.25	29401	4.65			
5	307.05	8423	1.33				10	560.20	8558	1.35			

Figure S70. LCMS spectrum of compound **25** (NPD-3382).

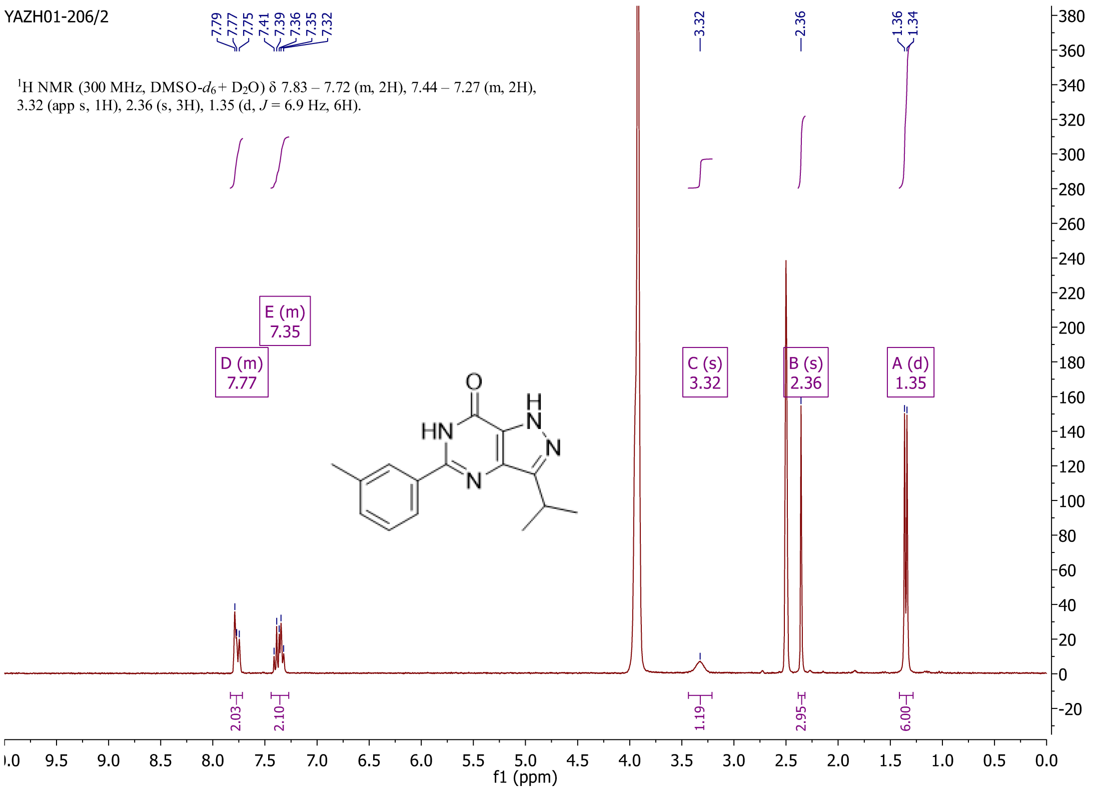

Figure S71. ^{1}H NMR spectrum of compound **25** (NPD-3382).

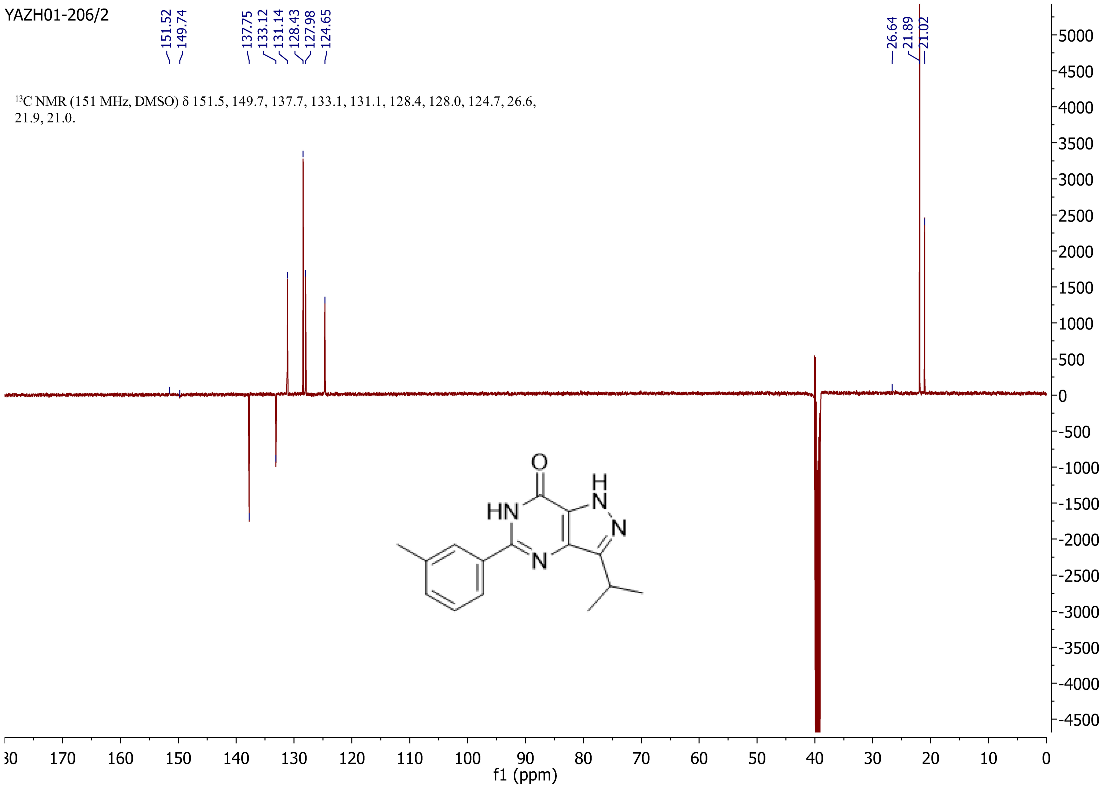

Figure S72. ^{13}C NMR spectrum of compound **25** (NPD-3382).

Acquired by	: Admin
Date Acquired	: 6/4/2018 11:20:23 AM
Sample Name	: YAZH01-197
Sample ID	:
Tray#	: 1
Vial#	: 4
Injection Volume	: 3
Data File	: C:\LabSolutions\Data\2018\2018-wk14\YAZH01-197.lcd
Background File	: blanco 06042018.lcd
Method File	: Method SCAN ACID standard.lcm
Report Format	: DefaultLCMS.lcr
Tuning File	: C:\LabSolutions\Tuning\Tuning-ESI-pos-neg01072015a.lct
Processed by	: Admin
Modified Date	: 6/4/2018 1:42:05 PM

PDA Graph

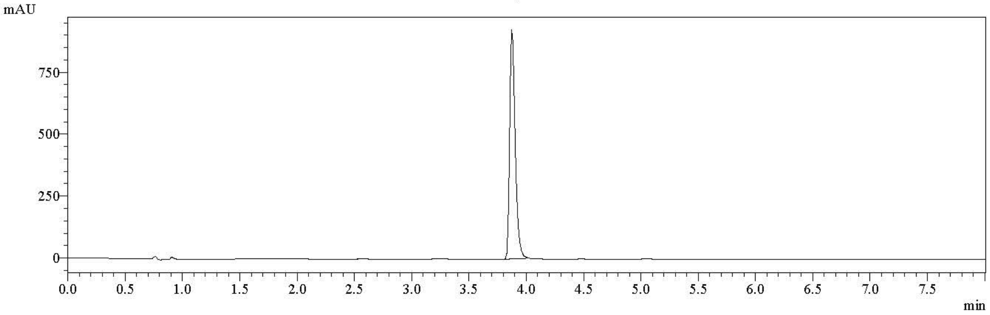

PDA Ch1 254nm 4nm

Peak#	Name	Ret. Time	Area	Area%
1		3.870	3116510	100.000

MS Spectrum Graph

#:1 Ret.Time:Averaged 3.920-3.940(Scan#:393-395)
Mass Peaks:11 Base Peak:285.05(1487272) Polarity:Pos Segment1 - Event1

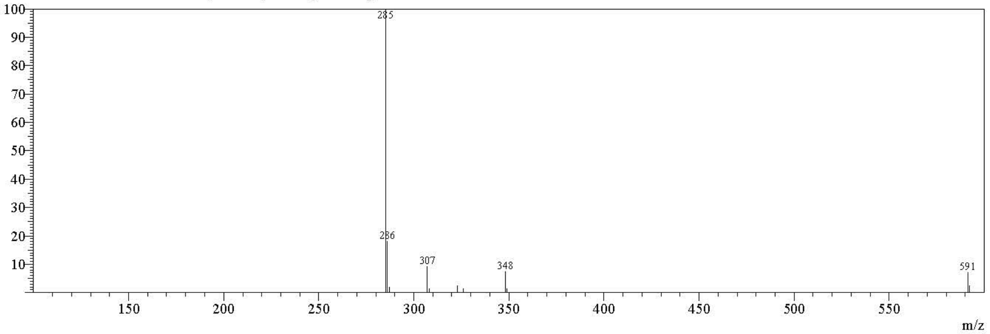

MS Spectrum Table

#:1 Ret.Time:
BG Mode:Calc 3.800<->4.210(381<->422)
Mass Peaks:11 Base Peak:285.05(1487272) Polarity:Pos Segment1 - Event1

#	m/z	Abs.Inten.	Rel.Inten.	Charge	Polarity	Monoisotopic
1	285.05	1487272	100.00			
2	286.10	270037	18.16			
3	287.10	27058	1.82			
4	307.10	136434	9.17			
5	308.05	21442	1.44			
6	323.00	35404	2.38			
7	326.10	21194	1.43			
8	348.10	108135	7.27			
9	349.10	20152	1.35			
10	591.25	106501	7.16			
11	592.30	36512	2.45			

Figure S73. LCMS spectrum of compound **26** (NPD-3375).

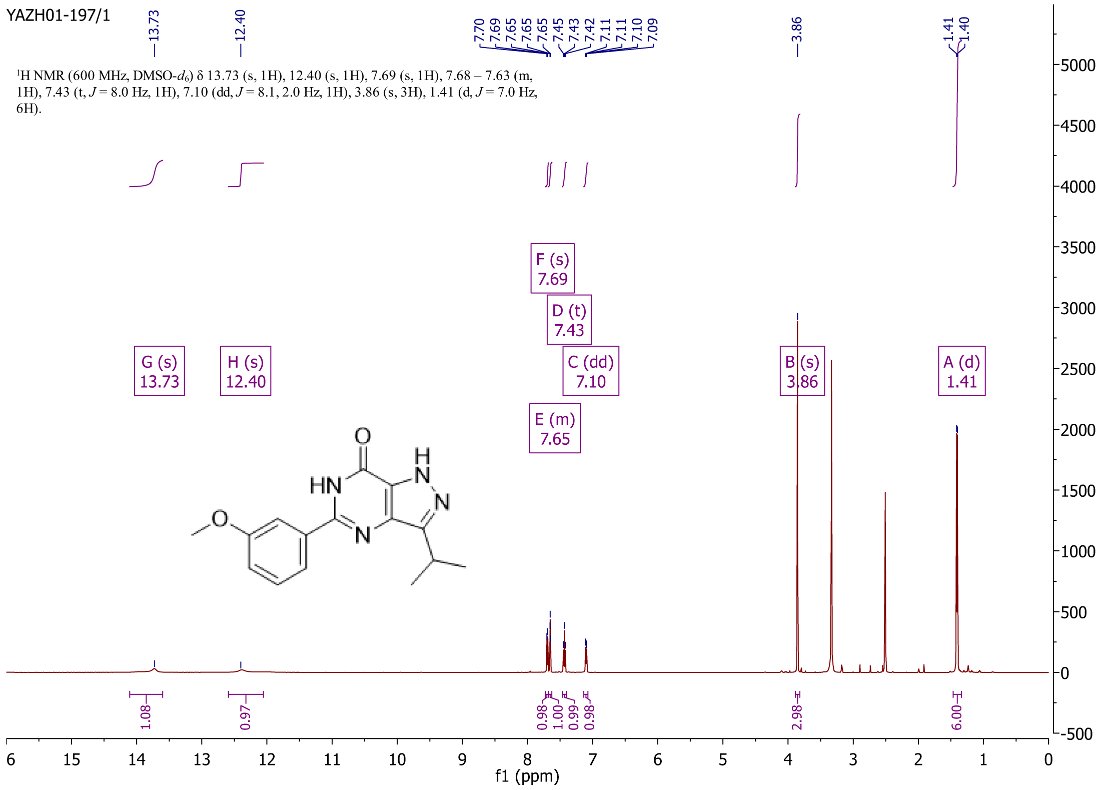

Figure S74. ^{1}H NMR spectrum of compound **26** (NPD-3375).

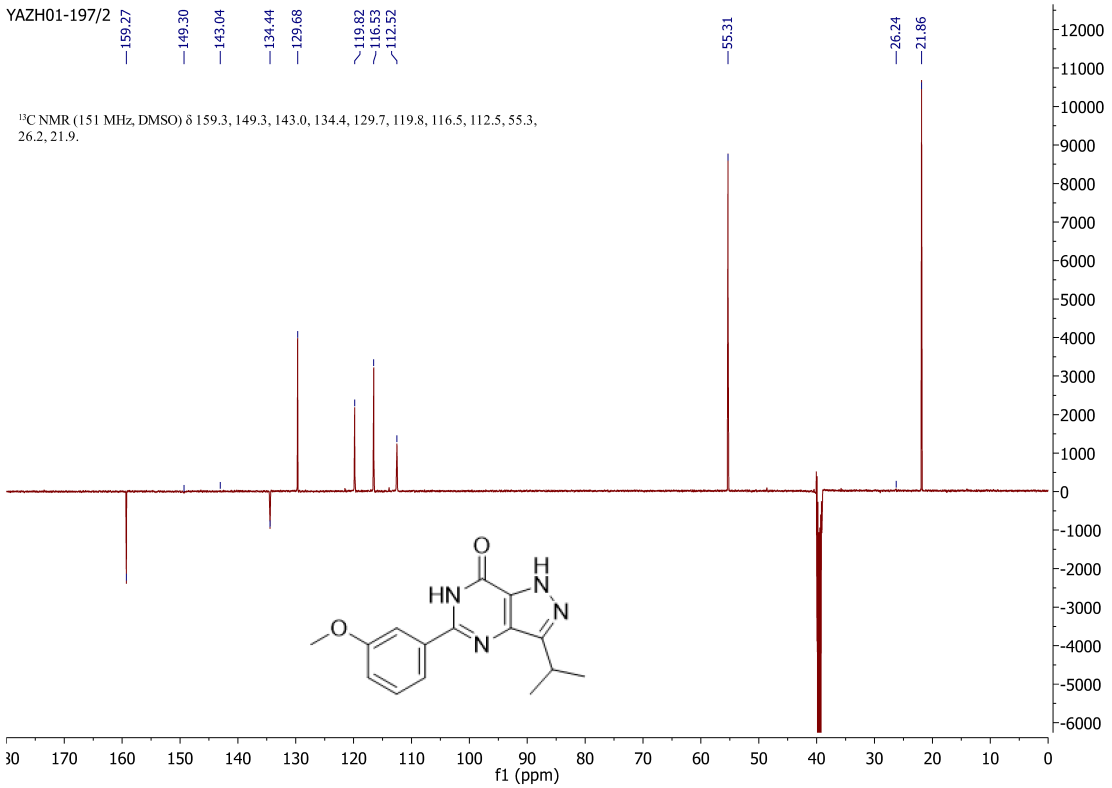

Figure S75. ^{13}C NMR spectrum of compound **26** (NPD-3375).

Acquired by	: Admin
Date Acquired	: 19/5/2016 5:09:21 PM
Sample Name	: YAZH-132
Sample ID	:
Tray#	: 1
Vial#	: 64
Injection Volume	: 1
Data File	: C:\LabSolutions\Data\2016 - wk20\YAZH-132.lcd
Background File	: blanco 19052016.lcd
Method File	: Method SCAN ACID standard.lcm
Report Format	: DefaultLCMS.lcr
Tuning File	: C:\LabSolutions\Tuning\Tuning-ESI-pos-neg01072015a.lct
Processed by	: Admin
Modified Date	: 7/6/2019 4:39:41 PM

PDA Graph

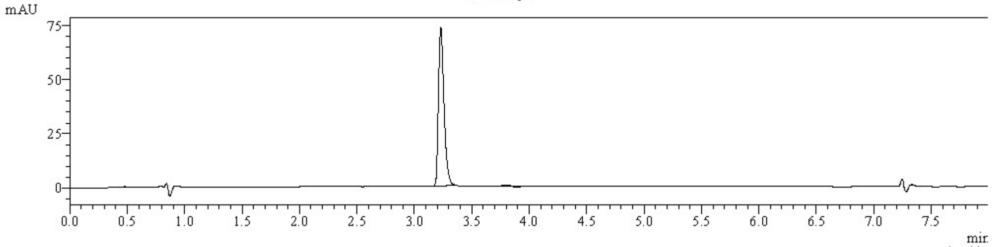

PeakTable

PDA Ch1 254nm 4nm

Peak#	Name	Ret. Time	Area	Area %
1		3.225	245962	99.351
2		3.795	1605	0.649

MS Chromatogram

YAZH-132 C:\LabSolutions\Data\2016\2016 - wk11-wk20\2016 - wk20\YAZH-132.lcd

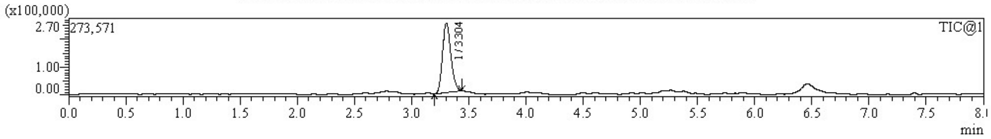

MS Spectrum Graph

#1 Ret.Time:Averaged 3.290-3.310(Scan#:330-332)
BG Mode:Calc 3.200<->3.440(321<->345)
Mass Peaks:10 Base Peak:271.05(170873) Polarity:Pos Segment1 - Event1

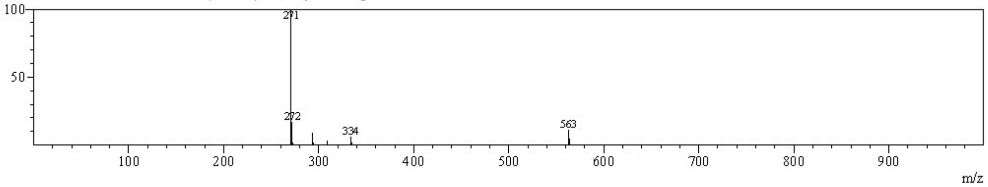

MS Spectrum Table

#:1 Ret.Time:
BG Mode:Calc 3.200<->3.440(321<->345)
Mass Peaks:10 Base Peak:271.05(170873) Polarity:Pos Segment1 - Event1

#	m/z	Abs.Inten.	Rel.Inten.	Charge	Polarity	Monoisotopic
1	271.05	170873	100.00			
2	272.00	28445	16.65			
3	273.10	2799	1.64			
4	293.05	15035	8.80			
5	294.10	3082	1.80			

#	m/z	Abs.Inten.	Rel.Inten.	Charge	Polarity	Monoisotopic
6	309.05	5301	3.10			
7	334.00	9979	5.84			
8	335.00	2379	1.39			
9	563.20	18408	10.77			
10	564.25	7953	4.65			

Figure S76. LCMS spectrum of compound **27** (NPD-2974).

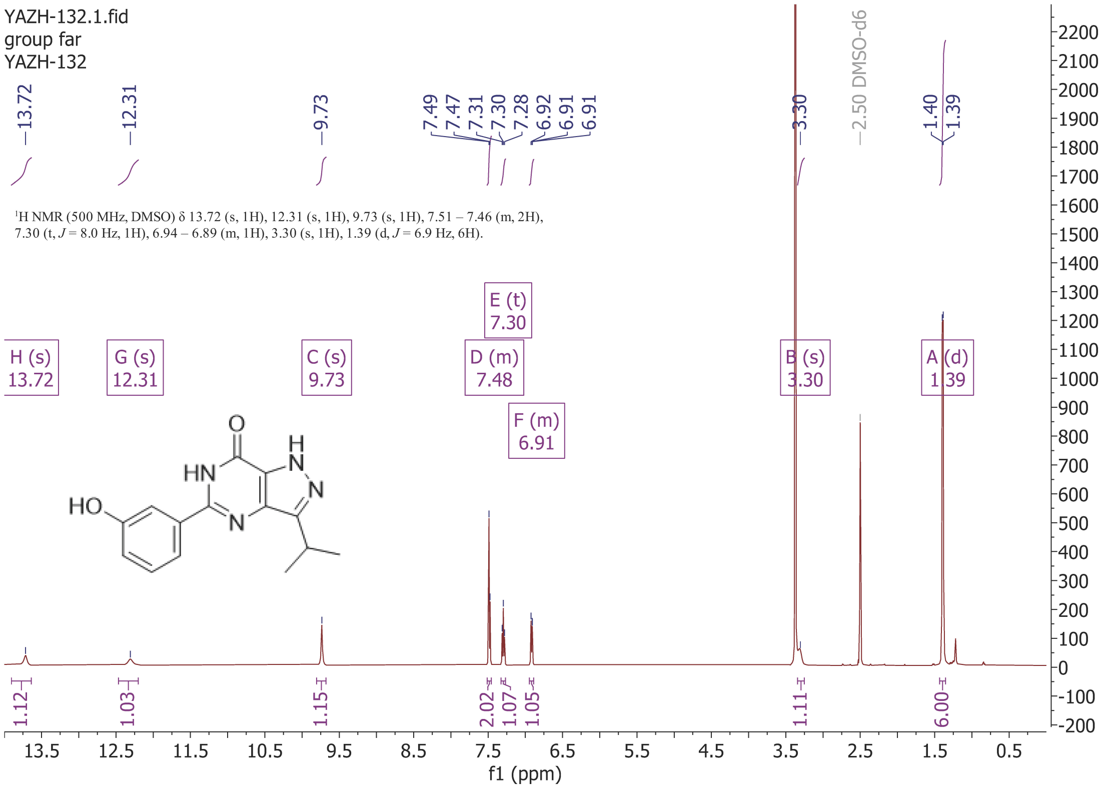

Figure S77. ^{1}H NMR spectrum of compound **27** (NPD-2974).

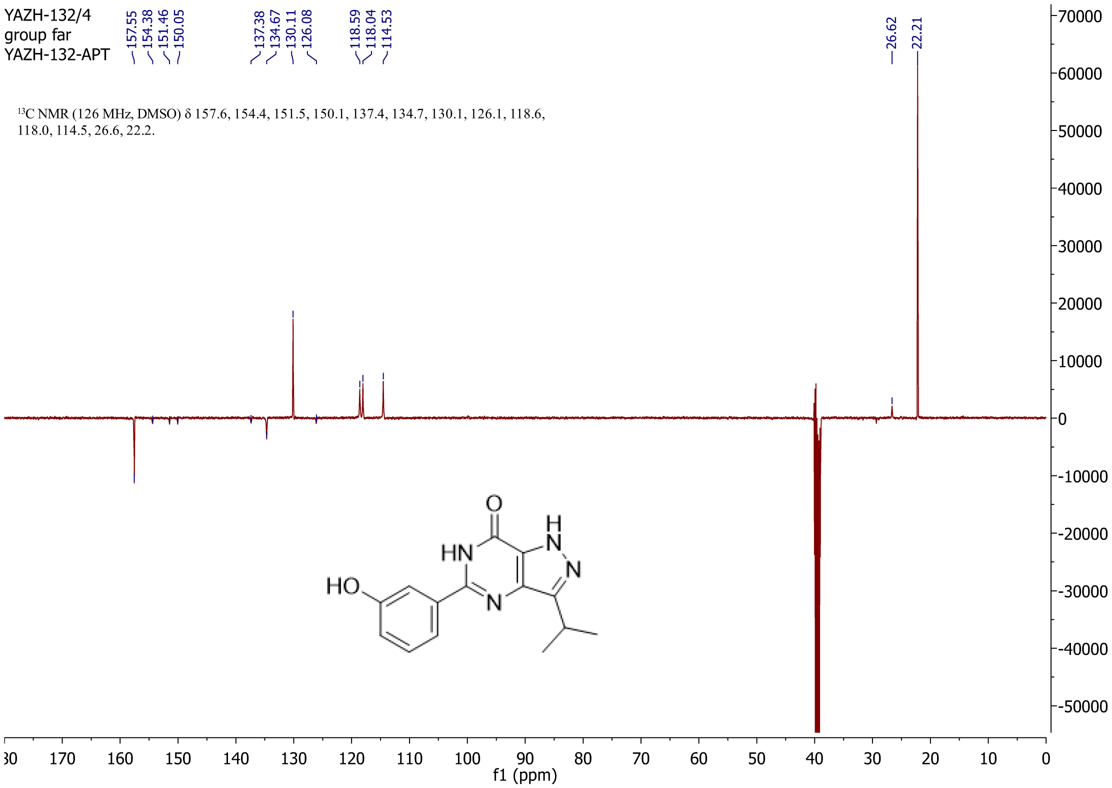

Figure S78. ^{13}C NMR spectrum of compound **27** (NPD-2974).

Acquired by : Admin
Date Acquired : 25/4/2018 6:05:16 PM
Sample Name : YAZH01-205
Sample ID :
Tray# : 1
Vial# : 4
Injection Volume : 3
Data File : C:\LabSolutions\Data\2018\2018-wk17\YAZH01-205.lcd
Background File : blanco 25042018.lcd
Method File : Method SCAN ACID standard.lcm
Report Format : DefaultLCMS.lcr
Tuning File : C:\LabSolutions\Tuning\Tuning-ESI-pos-neg01072015a.lct
Processed by : Admin
Modified Date : 26/4/2018 12:10:54 PM

PDA Graph

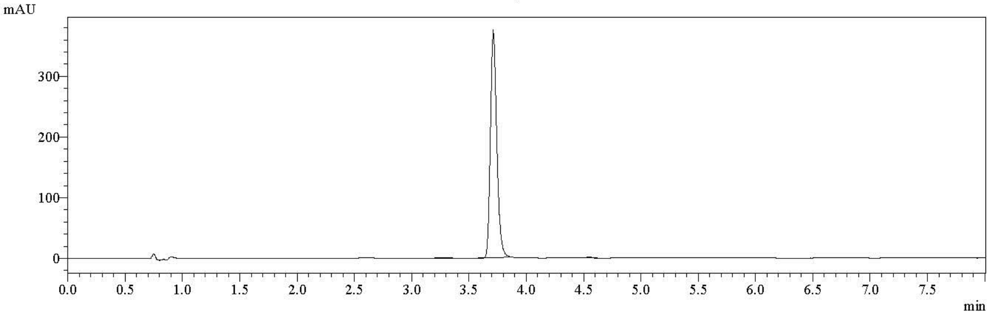

PDA Ch1 254nm 4nm

Peak#	Name	Ret. Time	Area	Area %
1		2.889	937	0.064
2		3.314	3541	0.242
3		3.707	1450372	99.147
4		4.339	1847	0.126
5		4.546	6154	0.421

MS Spectrum Graph

#:1 Ret.Time:Averaged 3.760-3.780(Scan#:377-379)
Mass Peaks:8 Base Peak:298.10(970419) Polarity:Pos Segment1 - Event1

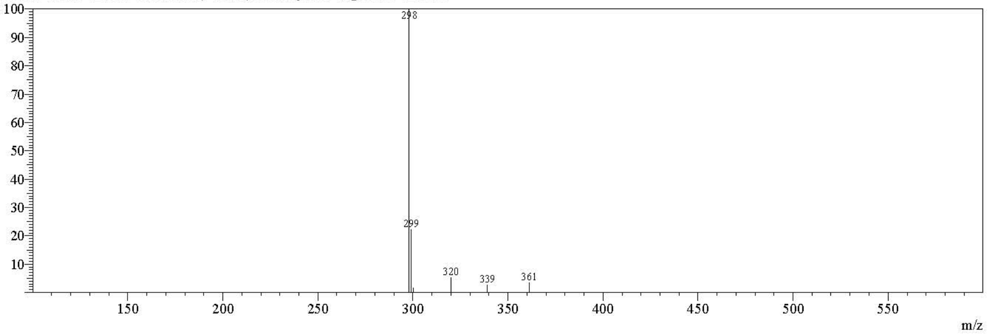

MS Spectrum Table

#:1 Ret.Time:
BG Mode:Calc 3.650<->3.990(366<->400)
Mass Peaks:8 Base Peak:298.10(970419) Polarity:Pos Segment1 - Event1

#	m/z	Abs.Inten.	Rel.Inten.	Charge	Polarity	Monoisotopic	#	m/z	Abs.Inten.	Rel.Inten.	Charge	Polarity	Monoisotopic
1	298.10	970419	100.00				5	339.15	24536	2.53			
2	299.10	215493	22.21				6	361.15	33629	3.47			
3	300.15	15720	1.62				7	617.35	36525	3.76			
4	320.05	49590	5.11				8	618.30	16194	1.67			

Figure S79. LCMS spectrum of compound **28** (NPD-3381).

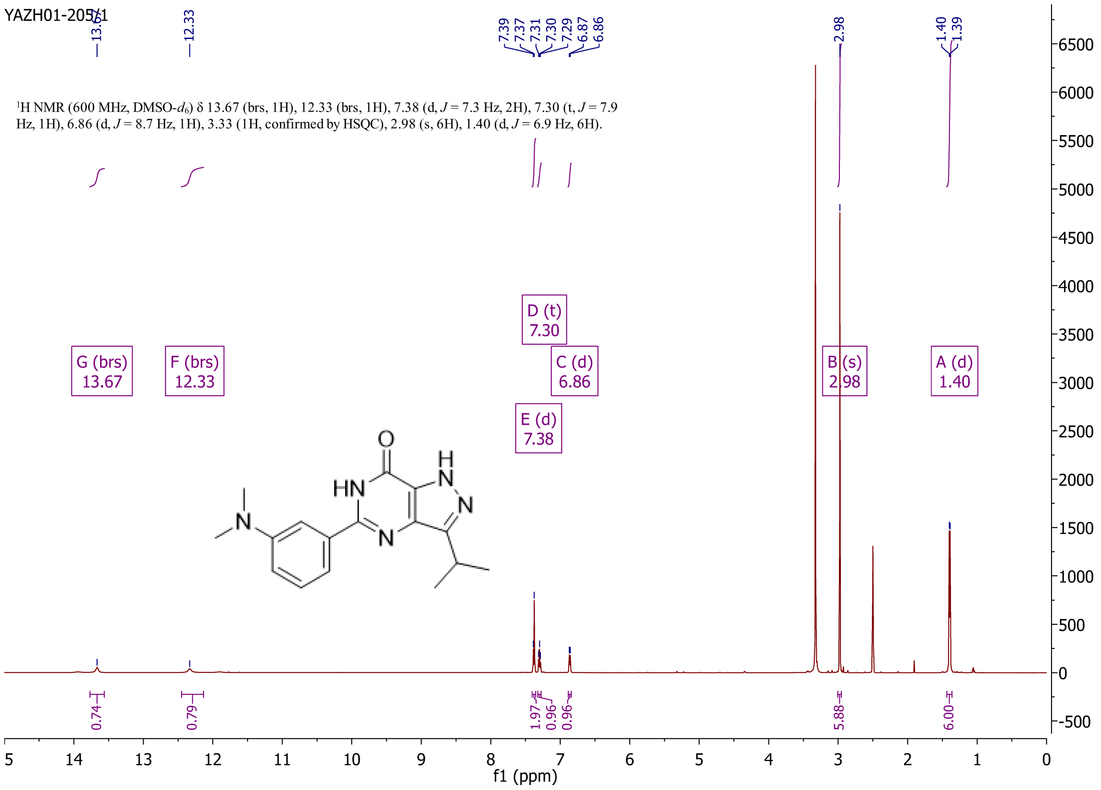

Figure S80. ^{1}H NMR spectrum of compound **28** (NPD-3381).

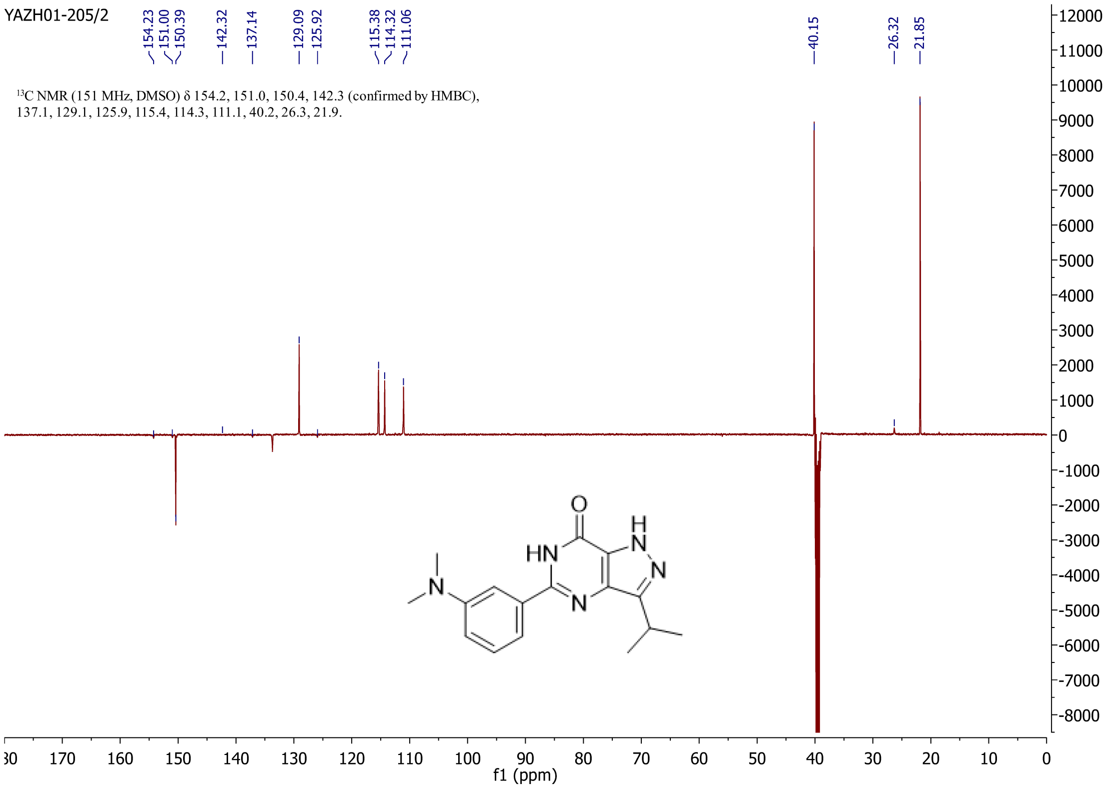

Figure S81. ^{13}C NMR spectrum of compound **28** (NPD-3381).

Acquired by : Admin
Date Acquired : 21/12/2018 10:46:27 AM
Sample Name : SHGI05_2
Sample ID :
Tray# : 1
Vial# : 4
Injection Volume : 3
Data File : C:\LabSolutions\Data\2018\2018-wk51\SHGI05_2.lcd
Background File : blanco 21122018.lcd
Method File : Method SCAN ACID standard.lcm
Report Format : DefaultLCMS.lcr
Tuning File : C:\LabSolutions\Tuning\Tuning-ESI-pos-neg01072015a.lct
Processed by : Admin
Modified Date : 18/6/2019 10:14:24 AM

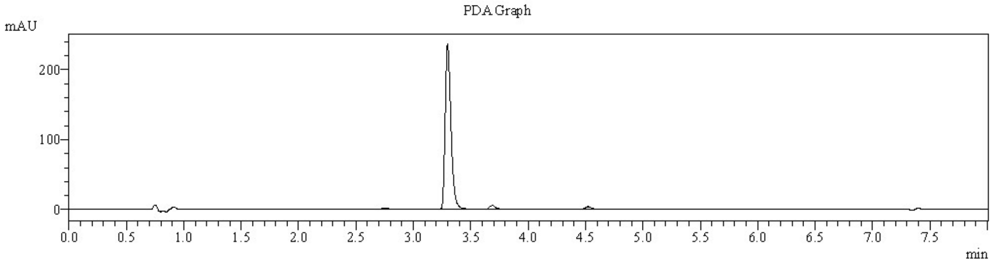

PeakTable

PDA Ch1 254nm 4nm

Peak#	Name	Ret. Time	Area	Area%
1		2.747	7230	0.852
2		3.294	809544	95.376
3		3.683	17912	2.110
4		4.516	14102	1.661

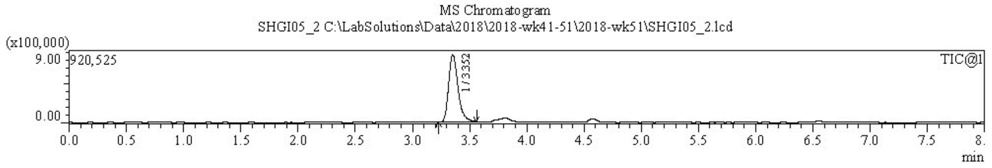

MS Spectrum Graph

#:1 Ret.Time:Averaged 3.340-3.360(Scan#:335-337)
BG Mode:Calc 3.230<->3.560(324<->357)
Mass Peaks:14 Base Peak:333.05(498382) Polarity:Pos Segment1 - Event1

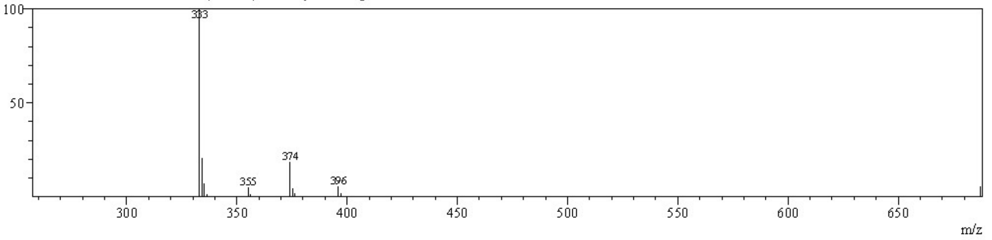

MS Spectrum Table

#:1 Ret.Time:
BG Mode:Calc 3.230<->3.560(324<->357)
Mass Peaks:14 Base Peak:333.05(498382) Polarity:Pos Segment1 - Event1

#	m/z	Abs.Inten.	Rel.Inten.	Charge	Polarity	Monoisotopic
1	257.20	9138	1.83			
2	333.05	498382	100.00			
3	334.05	101411	20.35			
4	335.05	33151	6.65			
5	336.20	5043	1.01			
6	355.10	22675	4.55			
7	356.10	6371	1.28			
8	374.10	91822	18.42			
9	375.10	20034	4.02			
10	376.05	8535	1.71			
11	396.05	26666	5.35			
12	397.10	7213	1.45			
13	687.20	26306	5.28			
14	688.30	9073	1.82			

Figure S82. LCMS spectrum of compound **29** (NPD-3598).

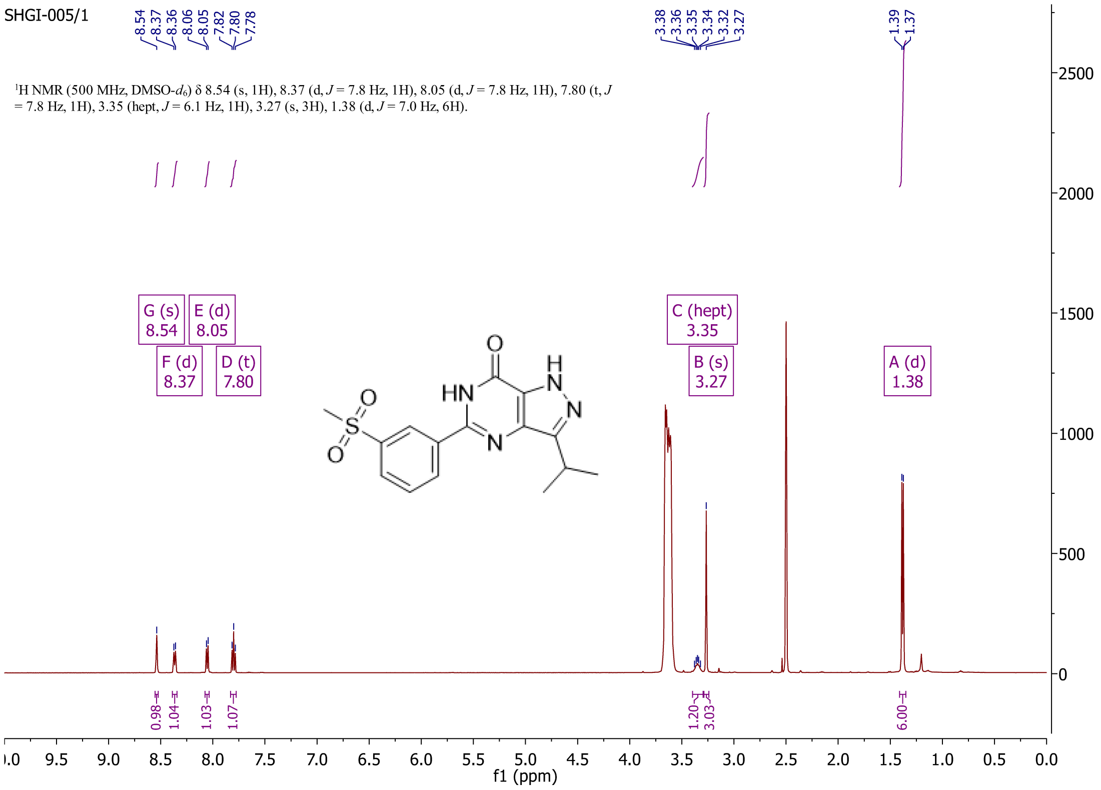

Figure S83. ^{1}H NMR spectrum of compound **29** (NPD-3598).

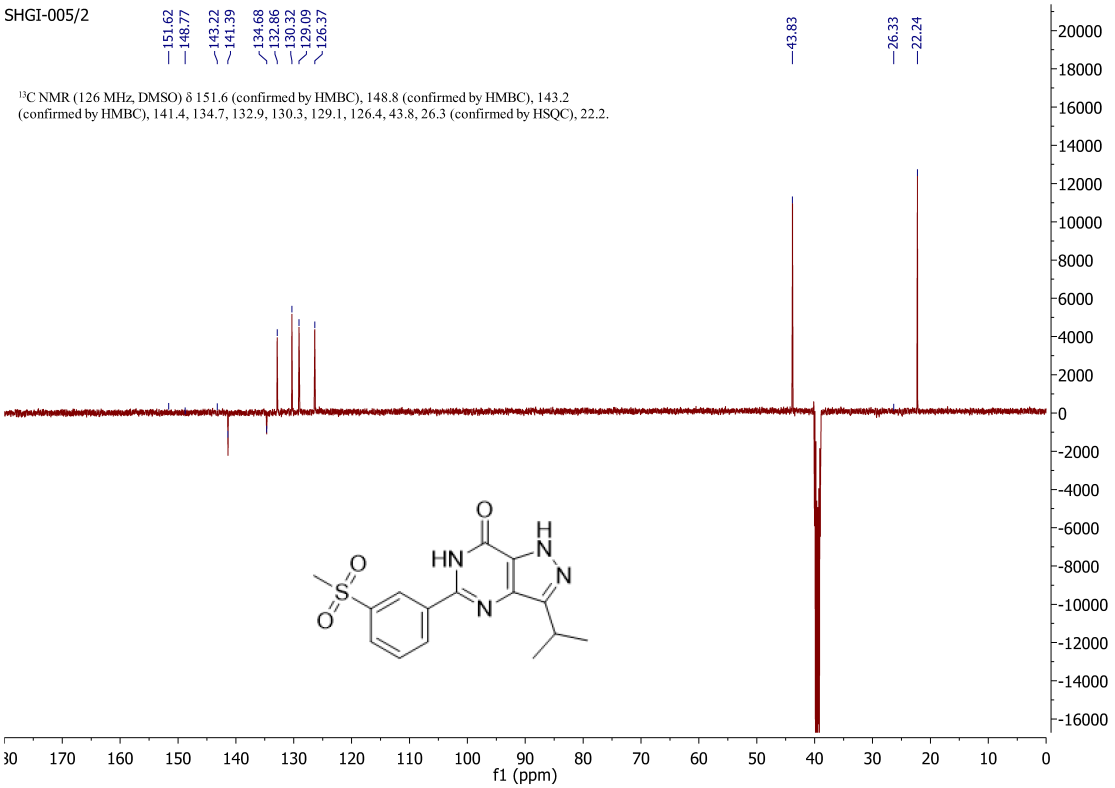

Figure S84. ^{13}C NMR spectrum of compound **29** (NPD-3598).

Acquired by	: Admin
Date Acquired	: 1/11/2017 10:01:55 AM
Sample Name	: YAZH01-165
Sample ID	:
Tray#	: 1
Vial#	: 1
Injection Volume	: 5
Data File	: C:\LabSolutions\Data\2017\2017-wk44\YAZH01-165.lcd
Background File	: blanco 01112017.lcd
Method File	: Method SCAN ACID standard.lcm
Report Format	: DefaultLCMS.lcr
Tuning File	: C:\LabSolutions\Tuning\Tuning-ESI-pos-neg01072015a.lct
Processed by	: Admin
Modified Date	: 1/11/2017 11:59:11 AM

Chromatogram
YAZH01-165 C:\LabSolutions\Data\2017\2017-wk44\YAZH01-165.lcd

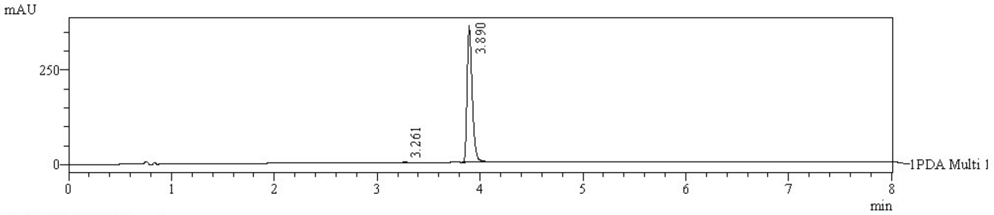

1 PDA Multi 1 / 254nm 4nm

PeakTable

PDA Ch1 254nm 4nm

Peak#	Name	Ret. Time	Area	Area %
1		3.261	4864	0.398
2		3.890	1217617	99.602

MS Chromatogram
YAZH01-165 C:\LabSolutions\Data\2017\2017-wk44\YAZH01-165.lcd

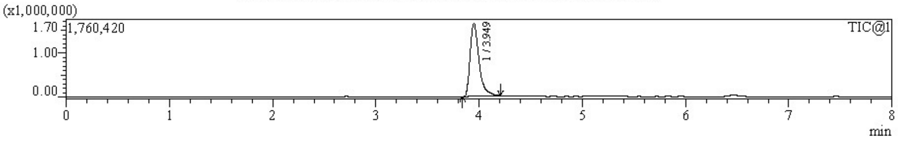

MS Spectrum Graph

#1 Ret.Time:Averaged 3.940-3.960(Scan#:395-397)
Mass Peaks:14 Base Peak:273.10(948365) Polarity:Pos Segment1 - Event1

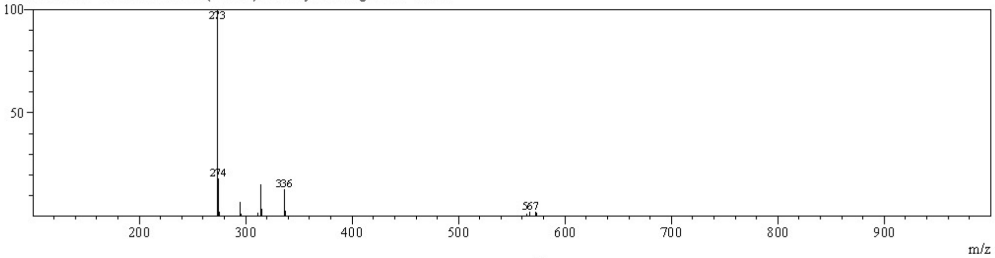

MS Spectrum Table

#:1 Ret.Time:
BG Mode:Calc 3.840<->4.210(385<->422)
Mass Peaks:14 Base Peak:273.10(948365) Polarity:Pos Segment1 - Event1

#	m/z	Abs.Inten.	Rel.Inten.	Charge	Polarity	Monoisotopic
1	273.10	948365	100.00			
2	274.10	171950	18.13			
3	275.10	19070	2.01			
4	295.05	61831	6.52			
5	296.05	10385	1.10			
6	311.00	12838	1.35			
7	314.10	144095	15.19			
8	315.10	29998	3.16			
9	336.10	121309	12.79			
10	337.15	24569	2.59			
11	564.45	10160	1.07			
12	567.25	19003	2.00			
13	572.25	18700	1.97			
14	572.90	15214	1.60			

Figure S85. LCMS spectrum of compound **30** (NPD-2975).

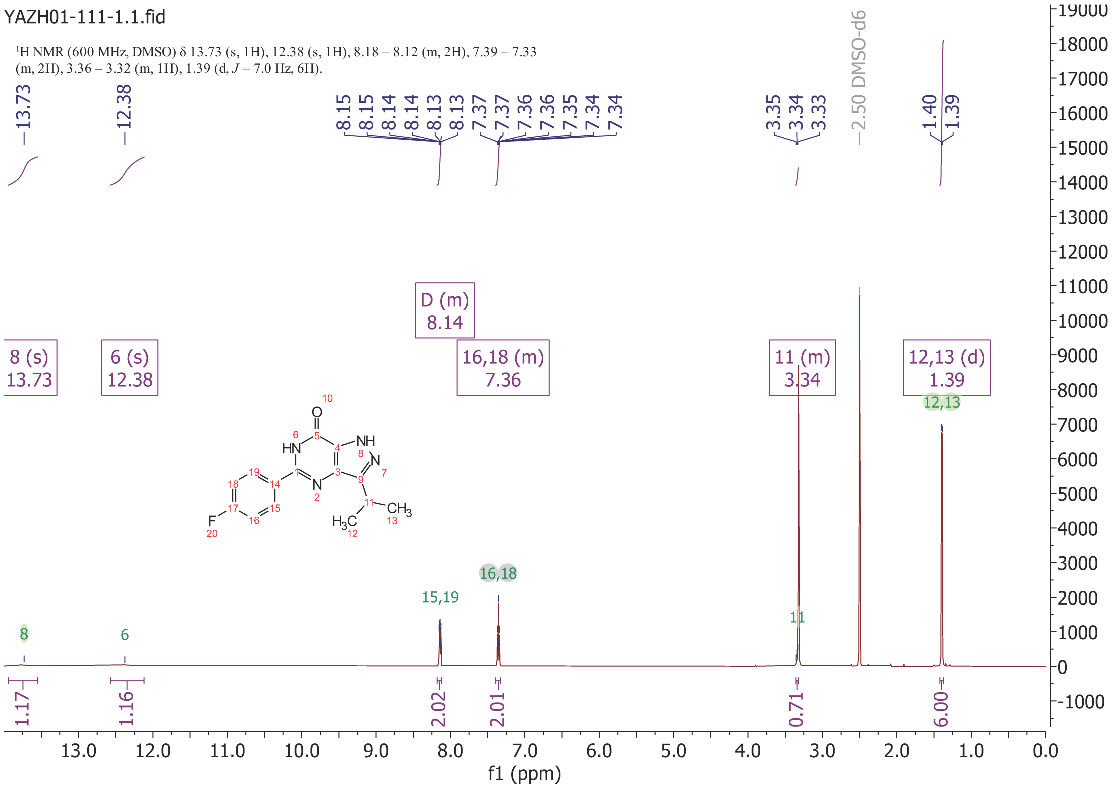

Figure S86. ¹H NMR spectrum of compound **30** (NPD-2975).

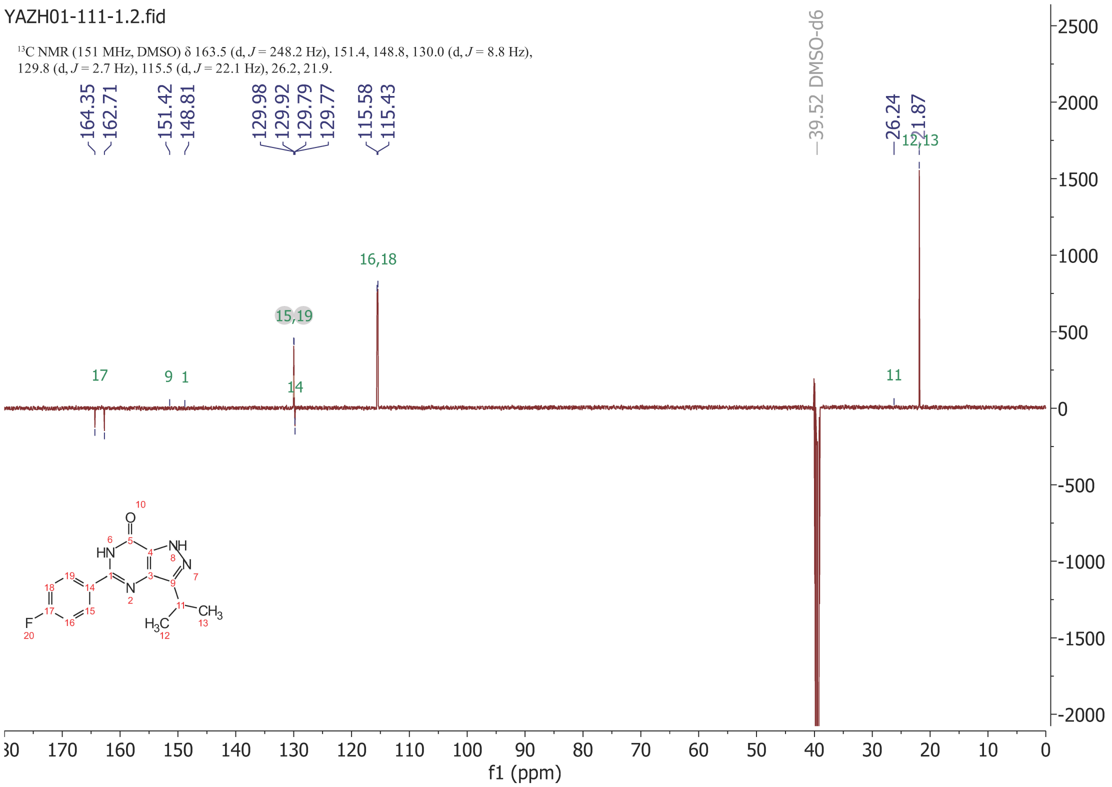

Figure S87. ¹³C NMR spectrum of compound **30** (NPD-2975).

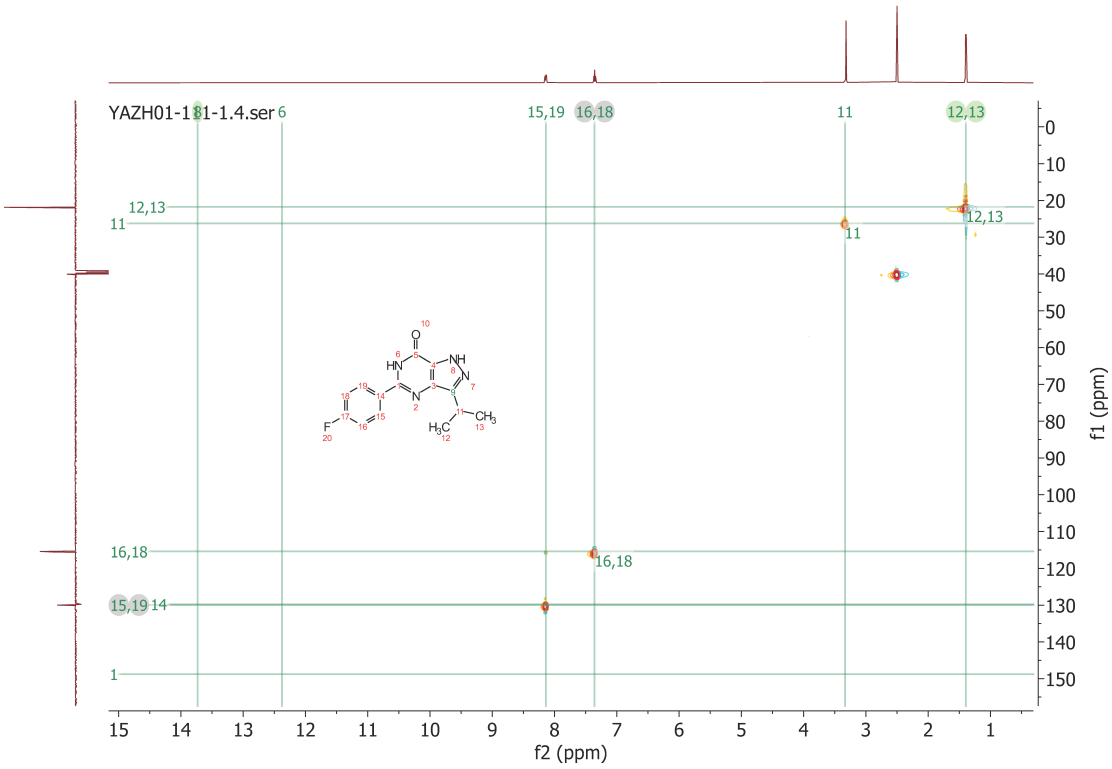

Figure S88. HSQC spectrum of compound **30** (NPD-2975).

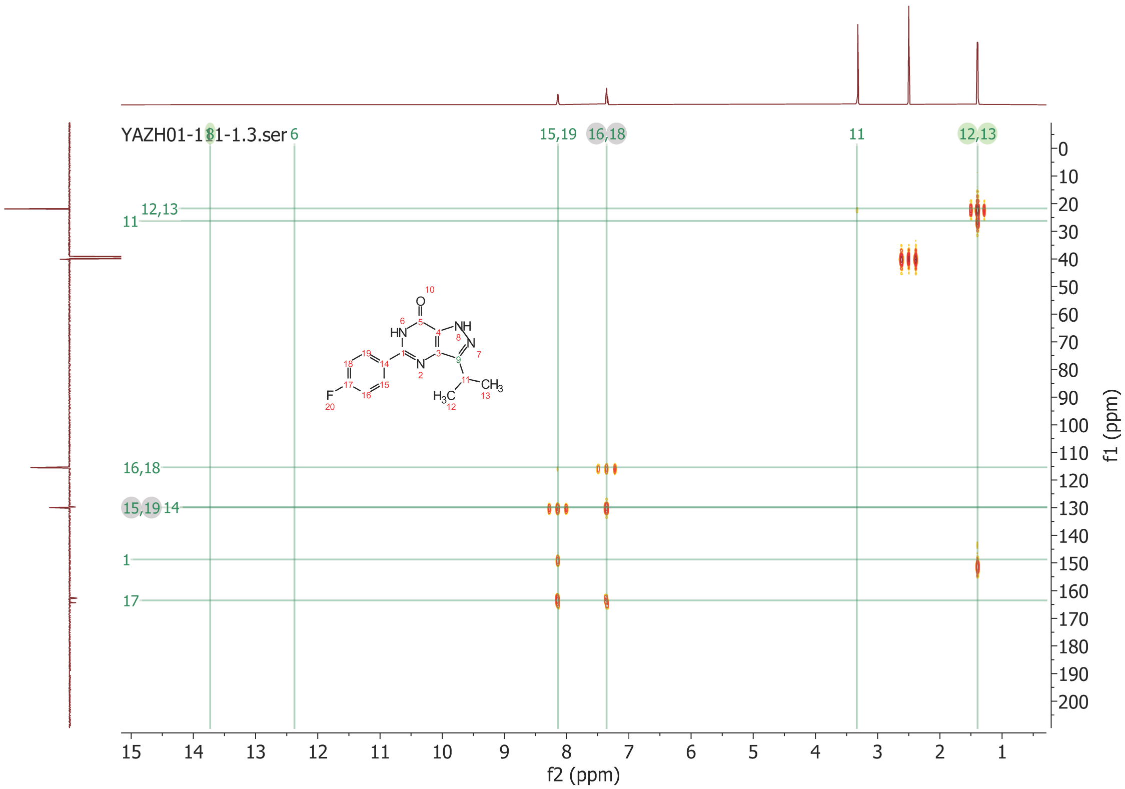

Figure S89. HMBC spectrum of compound **30** (NPD-2975).

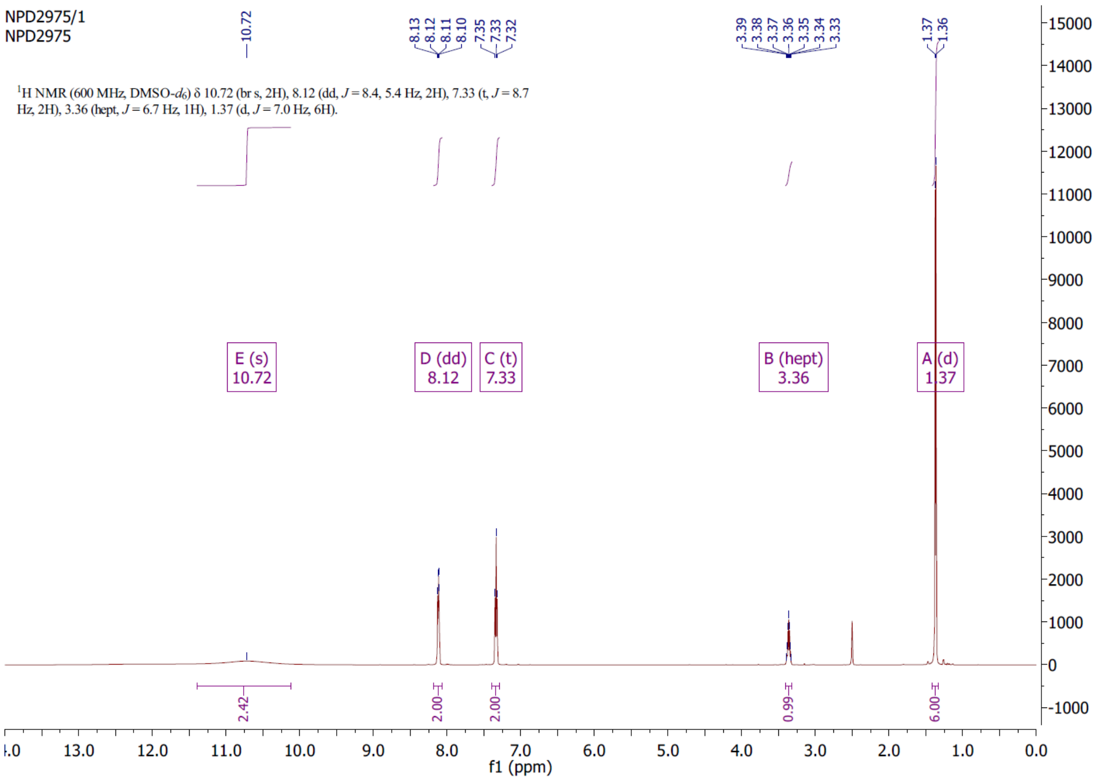

Figure S90. ^{1}H NMR spectrum of **30**·xHCl.

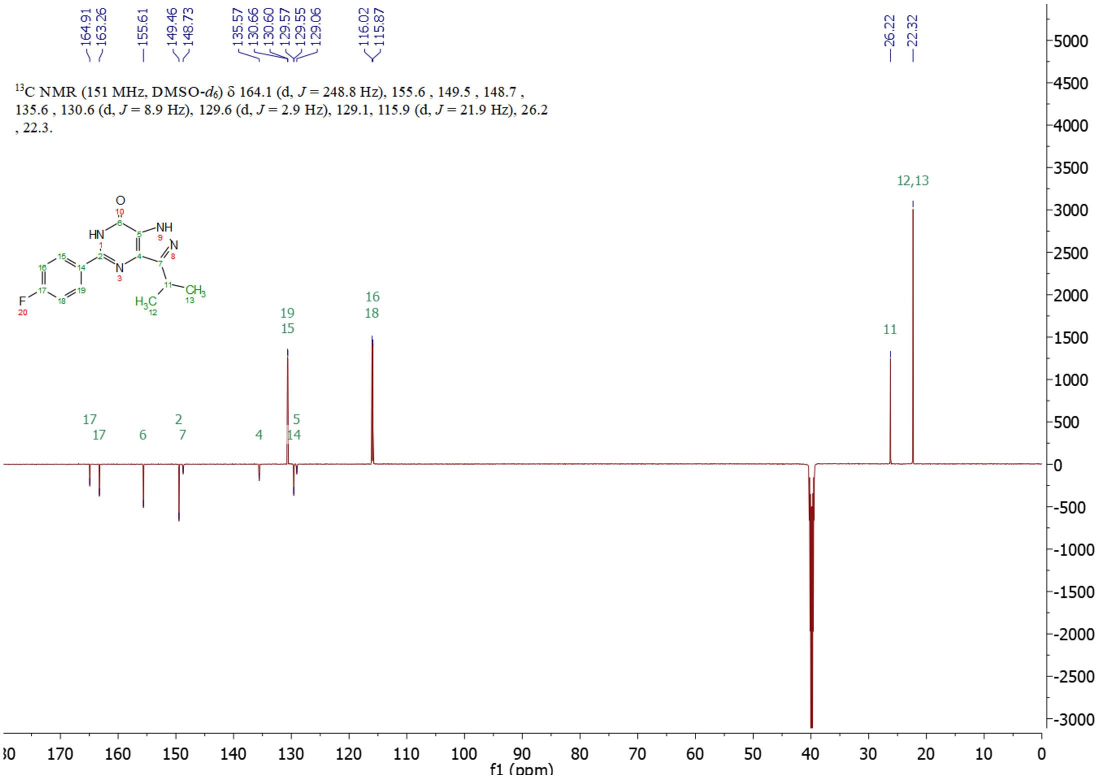

Figure S91. ^{13}C NMR spectrum of **30**·xHCl.

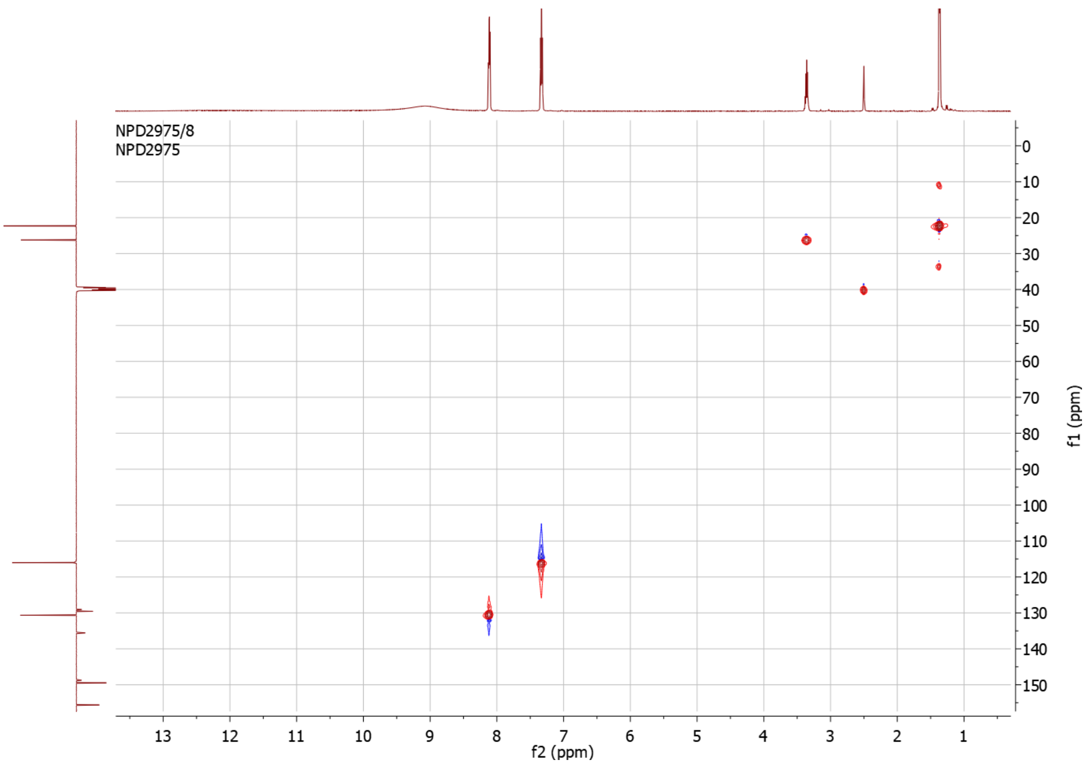

Figure S92. HSQC spectrum of **30**·xHCl.

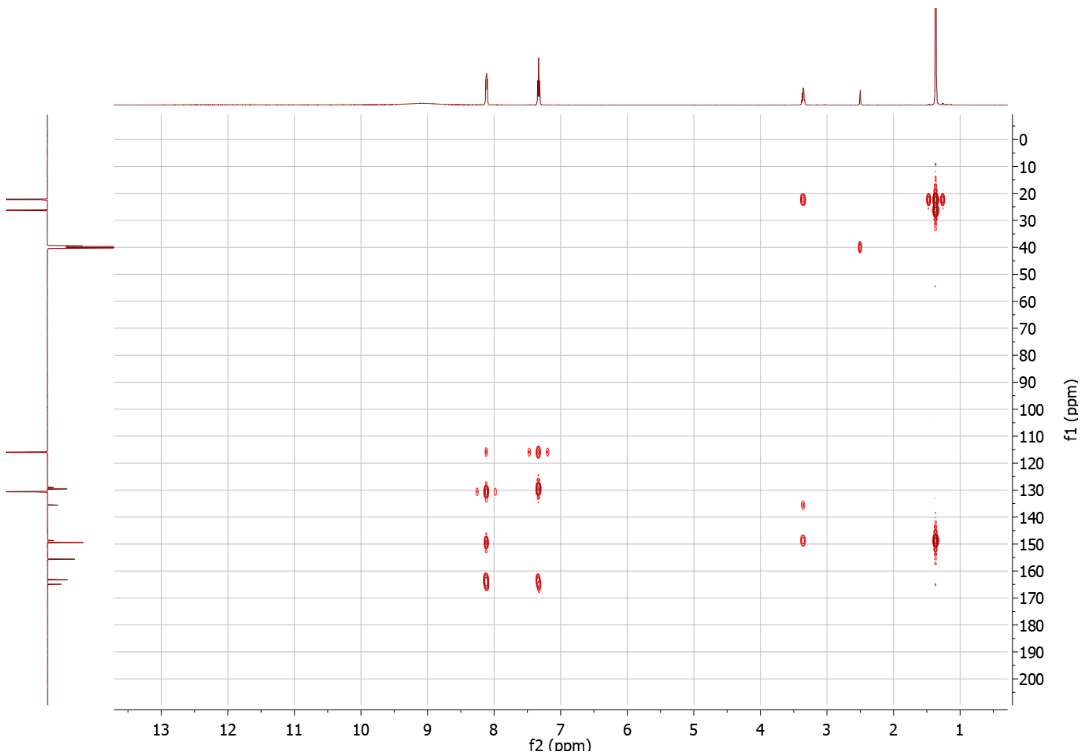

Figure S93. HMBC spectrum of **30**·xHCl.

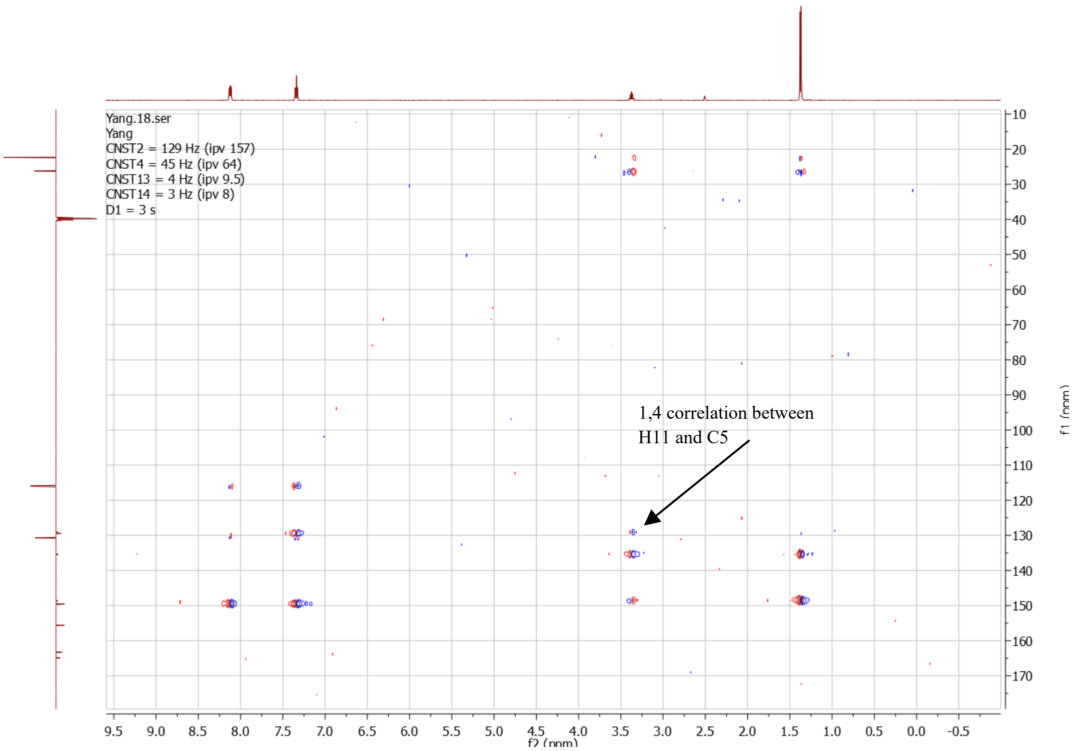

Figure S94. 1,n-ADEQUATE spectrum of **30**·xHCl.

Acquired by	: Admin
Date Acquired	: 3/7/2019 12:38:00 PM
Sample Name	: VUF16258
Sample ID	:
Tray#	: 1
Vial#	: 16
Injection Volume	: 3
Data File	: C:\LabSolutions\Data\2019\2019-wk27\VUF16258.lcd
Background File	: blanco 03072019.lcd
Method File	: Method SCAN ACID standard.lcm
Report Format	: DefaultLCMS.lcr
Tuning File	: C:\LabSolutions\Tuning\Tuning-ESI-pos-neg01072015a.lct
Processed by	: Admin
Modified Date	: 3/7/2019 1:00:26 PM

Chromatogram
VUF16258 C:\LabSolutions\Data\2019\2019-wk27\VUF16258.lcd

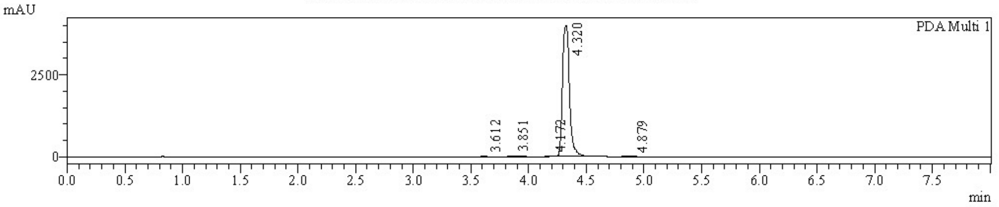

1 PDA Multi 1 / 254nm 4nm

PeakTabl

PDA Ch1 254nm 4nm

Peak#	Name	Ret. Time	Area	Area %
1		3.612	49161	0.290
2		3.851	136099	0.803
3		4.172	80388	0.474
4		4.320	16651820	98.220
5		4.879	36047	0.213

MS Chromatogram
VUF16258 C:\LabSolutions\Data\2019\2019-wk27\VUF16258.lcd

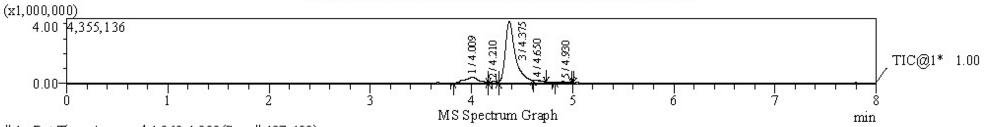

MS Spectrum Graph

#:1 Ret.Time:Averaged 4.360-4.380(Scan#:437-439)
Mass Peaks:20 Base Peak:289.05(1855458) Polarity:Pos Segment1 - Event1

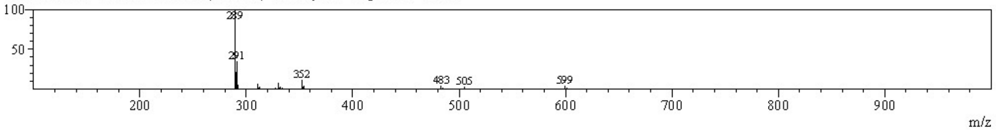

MS Spectrum Table

#:1 Ret.Time:
BG Mode:Calc 4.270<->5.010(428<->502)
Mass Peaks:20 Base Peak:289.05(1855458) Polarity:Pos Segment1 - Event1

#	m/z	Abs.Inten.	Rel.Inten.	Charge	Polarity	Monoisotopic	#	m/z	Abs.Inten.	Rel.Inten.	Charge	Polarity	Monoisotopic
1	289.05	1855458	100.00				11	332.05	49527	2.67			
2	290.00	382010	20.59				12	334.10	21179	1.14			
3	291.00	631041	34.01				13	352.00	198202	10.68			
4	292.00	97642	5.26				14	353.00	37343	2.01			
5	311.00	106208	5.72				15	354.05	64047	3.45			
6	312.05	20916	1.13				16	483.20	65350	3.52			
7	313.00	38894	2.10				17	485.15	23119	1.25			
8	327.00	24731	1.33				18	505.15	44990	2.42			
9	330.00	141151	7.61				19	599.15	61199	3.30			
10	331.00	29256	1.58				20	601.15	33455	1.80			

Figure S95. LCMS spectrum of compound **31** (NPD-3204).

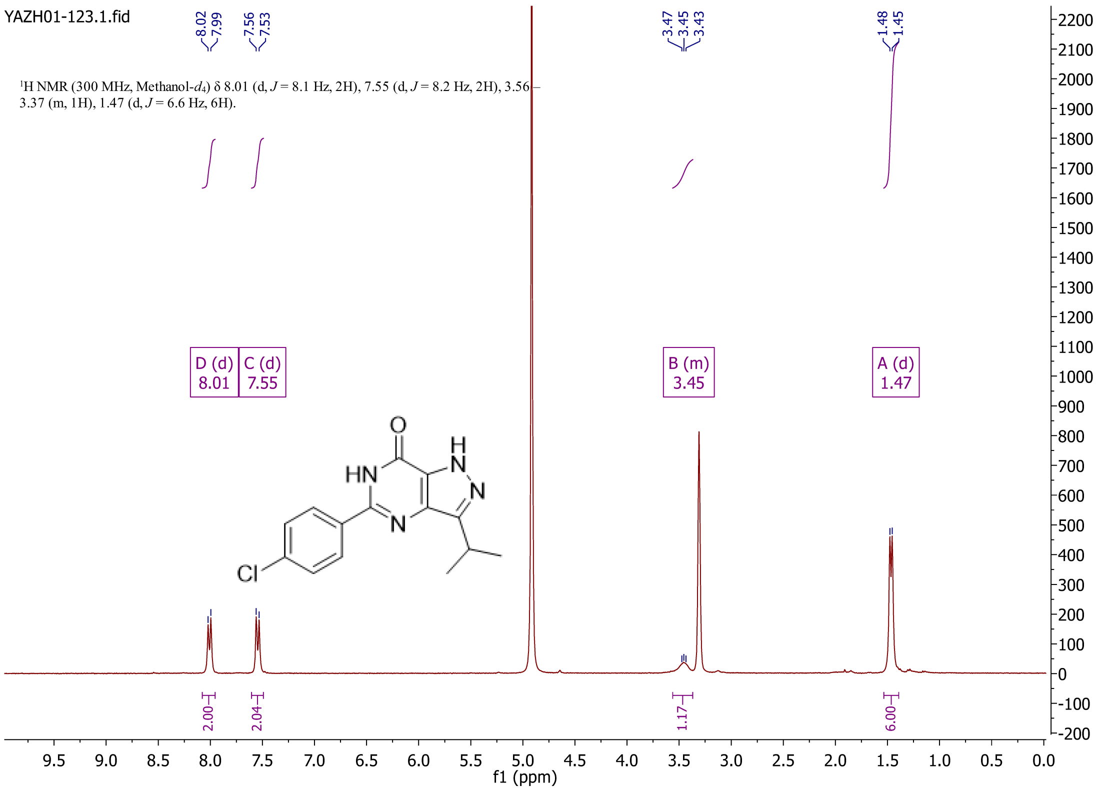

Figure S96. ^{1}H NMR spectrum of compound **31** (NPD-3204).

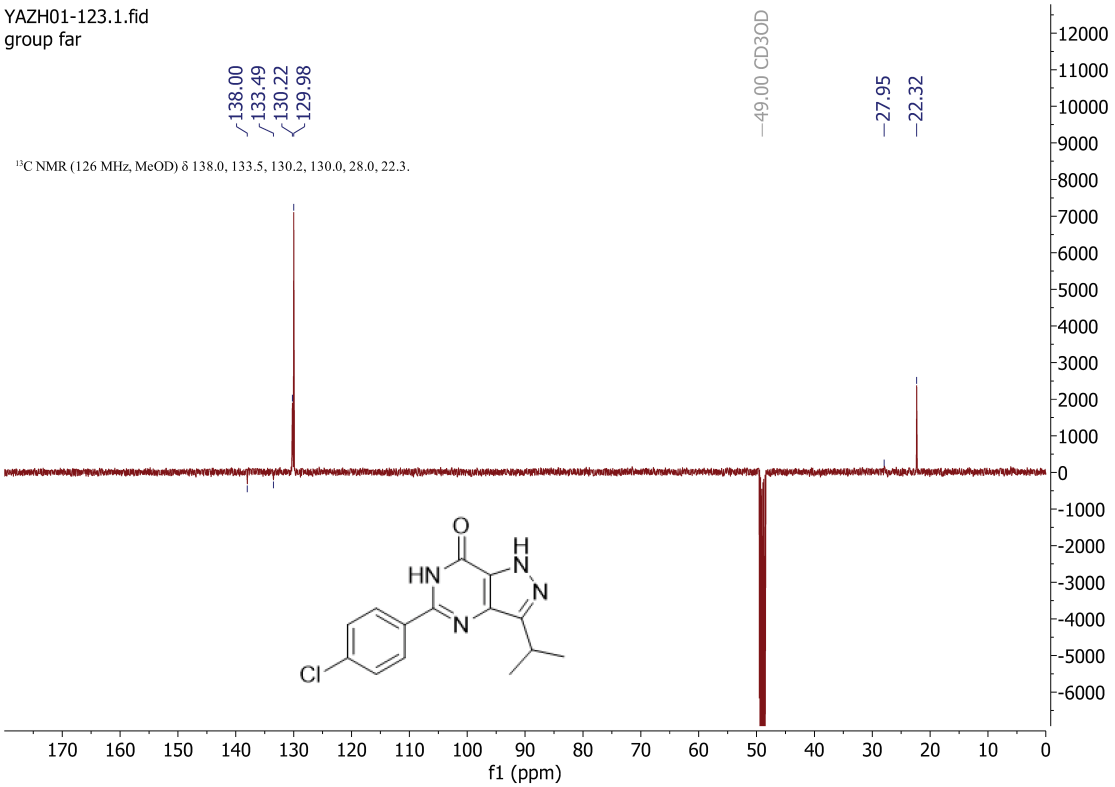

Figure S97. ^{13}C NMR spectrum of compound **31** (NPD-3204).

Acquired by	: Admin
Date Acquired	: 5/13/2016 6:38:45 PM
Sample Name	: YAZH-129
Sample ID	:
Tray#	: 1
Vial#	: 31
Injection Volume	: 1
Data File	: C:\LabSolutions\Data\2016 - wk19\YAZH-129.lcd
Background File	: blanco 13052016.lcd
Method File	: Method SCAN ACID standard.lcm
Report Format	: DefaultLCMS.lcr
Tuning File	: C:\LabSolutions\Tuning\Tuning-ESI-pos-neg01072015a.lct
Processed by	: Admin
Modified Date	: 5/17/2016 8:55:03 AM

PDA Graph

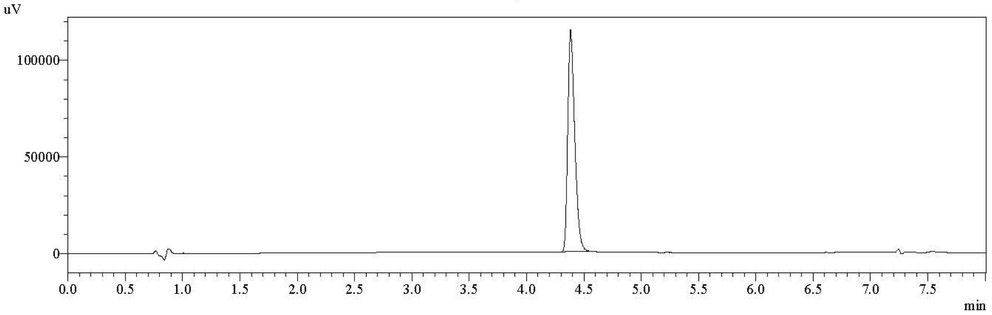

PDA Ch1 254nm 4nm

Peak#	Name	Ret. Time	Area	Area%
1		4.379	478820	100.000

MS Spectrum Graph

#:1 Ret.Time:Averaged 4.450-4.470(Scan#:446-448)
Mass Peaks:23 Base Peak:332.95(173681) Polarity:Pos Segment1 - Event1

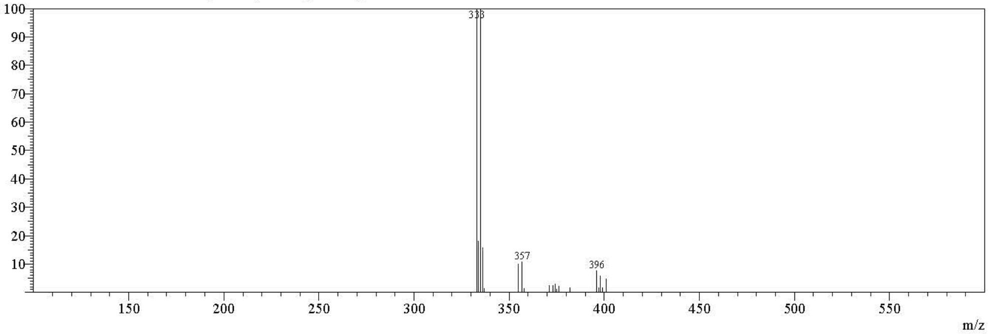

MS Spectrum Table

#:1 Ret.Time:
BG Mode:Calc 4.340<->4.680(435<->469)
Mass Peaks:23 Base Peak:332.95(173681) Polarity:Pos Segment1 - Event1

#	m/z	Abs.Inten.	Rel.Inten.	Charge	Polarity	Monoisotopic
1	332.95	173681	100.00			
2	333.95	31130	17.92			
3	334.95	173568	99.93			
4	335.95	27378	15.76			
5	336.95	2157	1.24			
6	354.90	17385	10.01			
7	356.85	18810	10.83			
8	357.80	2597	1.50			
9	371.00	4242	2.44			
10	372.90	4308	2.48			
11	374.00	5319	3.06			
12	374.95	1857	1.07			
13	375.95	3745	2.16			
14	381.95	2760	1.59			
15	396.00	13376	7.70			
16	397.10	3016	1.74			
17	398.00	10077	5.80			
18	399.00	2911	1.68			
19	401.10	8086	4.66			
20	688.55	3016	1.74			
21	689.25	2631	1.51			
22	689.65	2181	1.26			
23	691.25	2155	1.24			

Figure S98. LCMS spectrum of compound **32** (NPD-2971).

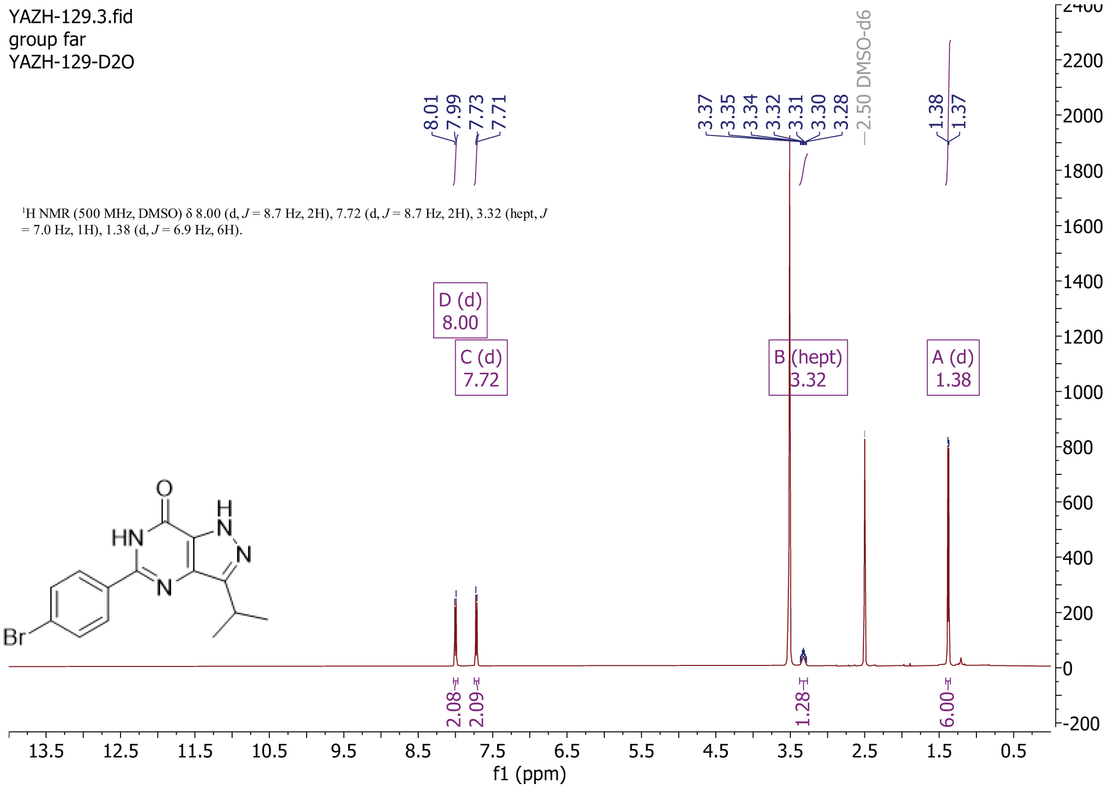

Figure S99. ^{1}H NMR spectrum of compound **32** (NPD-2971).

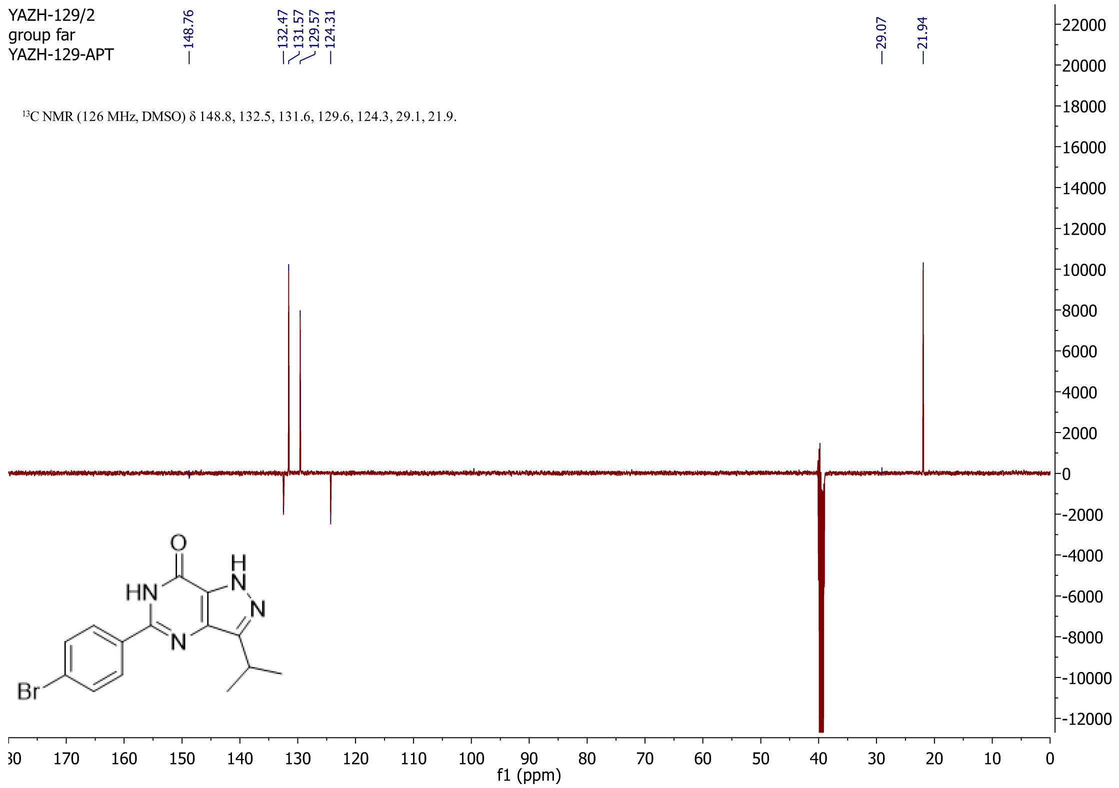

Figure S100. ^{13}C NMR spectrum of compound **32** (NPD-2971).

Acquired by	: Admin
Date Acquired	: 5/13/2016 6:47:19 PM
Sample Name	: YAZH-130
Sample ID	:
Tray#	: 1
Vial#	: 32
Injection Volume	: 1
Data File	: C:\LabSolutions\Data\2016 - wk19\YAZH-130.lcd
Background File	: blanco 13052016.lcd
Method File	: Method SCAN ACID standard.lcm
Report Format	: DefaultLCMS.lcr
Tuning File	: C:\LabSolutions\Tuning\Tuning-ESI-pos-neg01072015a.lct
Processed by	: Admin
Modified Date	: 5/17/2016 9:03:32 AM

PDA Graph

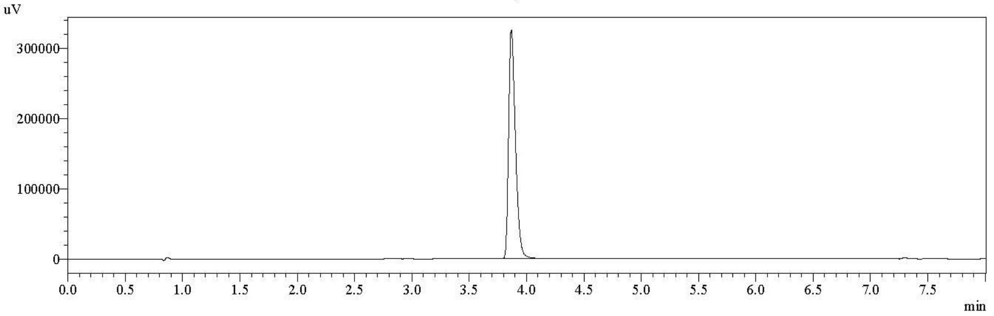

PDA Ch1 254nm 4nm

Peak#	Name	Ret. Time	Area	Area%
1		3.862	1345092	100.000

MS Spectrum Graph

#:1 Ret.Time:Averaged 3.930-3.950(Scan#:394-396)
Mass Peaks:8 Base Peak:285.05(784286) Polarity:Pos Segment1 - Event1

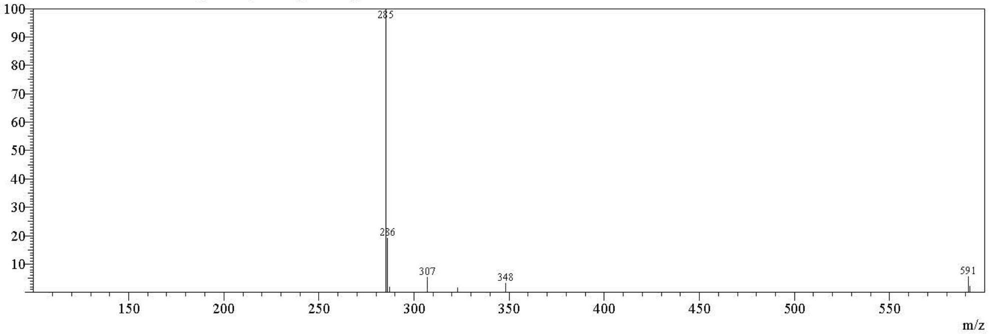

MS Spectrum Table

#:1 Ret.Time:
BG Mode:Calc 3.820<->4.170(383<->418)
Mass Peaks:8 Base Peak:285.05(784286) Polarity:Pos Segment1 - Event1

#	m/z	Abs.Inten.	Rel.Inten.	Charge	Polarity	Monoisotopic	#	m/z	Abs.Inten.	Rel.Inten.	Charge	Polarity	Monoisotopic
1	285.05	784286	100.00				5	323.05	13171	1.68			
2	286.05	149498	19.06				6	348.10	25832	3.29			
3	287.10	15769	2.01				7	591.25	43781	5.58			
4	306.95	41061	5.24				8	592.20	17656	2.25			

Figure S101. LCMS spectrum of compound **33** (NPD-2972).

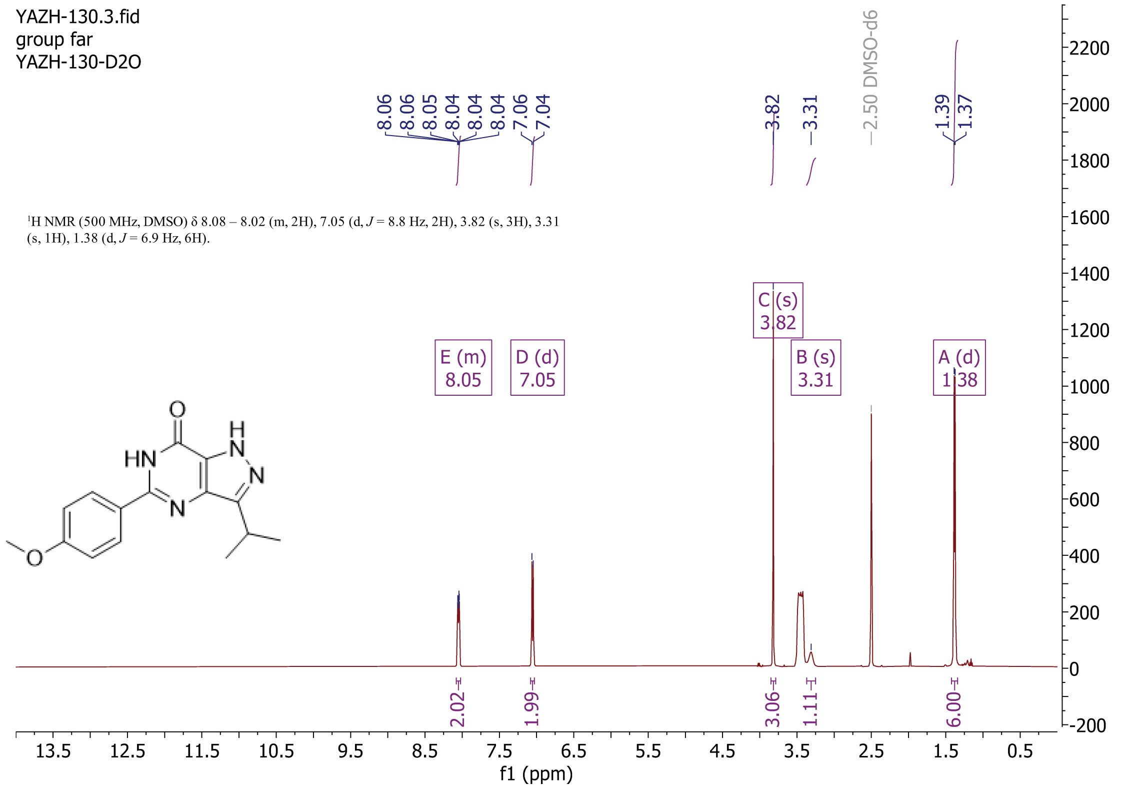

Figure S102. ^{1}H NMR spectrum of compound **33** (NPD-2972).

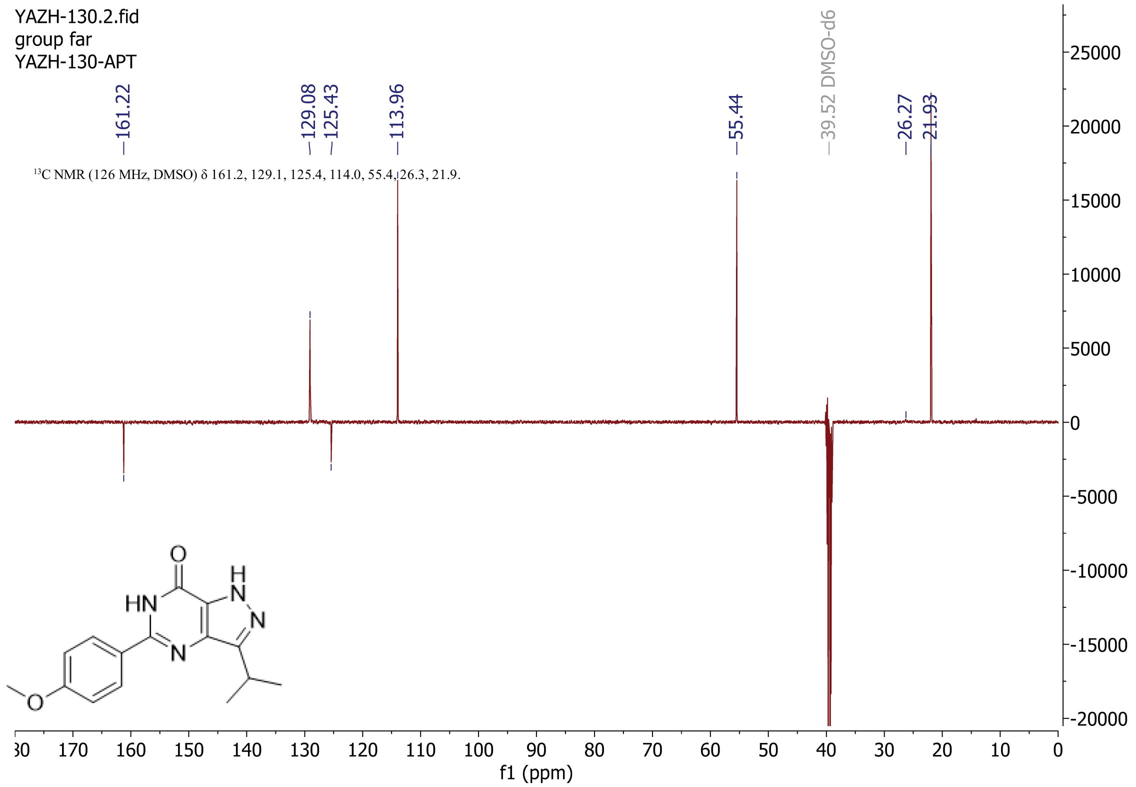

Figure S103. ^{13}C NMR spectrum of compound **33** (NPD-2972).

Acquired by	: Admin
Date Acquired	: 25/4/2018 1:58:14 PM
Sample Name	: YAZH01-201
Sample ID	:
Tray#	: 1
Vial#	: 21
Injection Volume	: 3
Data File	: C:\LabSolutions\Data\2018\2018-wk17\YAZH01-201.lcd
Background File	: blanco 25042018.lcd
Method File	: Method SCAN ACID standard.lcm
Report Format	: DefaultLCMS.lcr
Tuning File	: C:\LabSolutions\Tuning\Tuning-ESI-pos-neg01072015a.lct
Processed by	: Admin
Modified Date	: 25/4/2018 2:09:39 PM

Chromatogram
YAZH01-201 C:\LabSolutions\Data\2018\2018-wk17\YAZH01-201.lcd

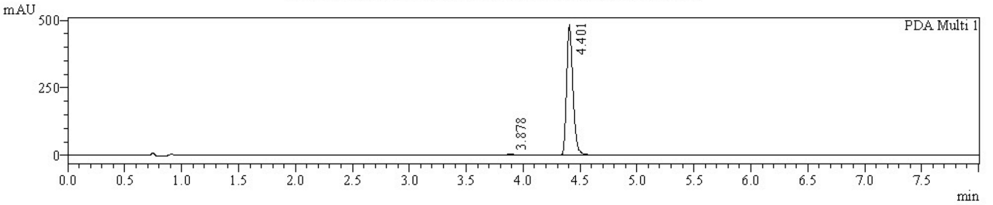

1 PDA Multi 1 / 254nm 4nm

PeakTabl

PDA Ch1 254nm 4nm

Peak#	Name	Ret. Time	Area	Area %
1		3.878	13465	0.726
2		4.401	1840199	99.274

MS Chromatogram
YAZH01-201 C:\LabSolutions\Data\2018\2018-wk17\YAZH01-201.lcd

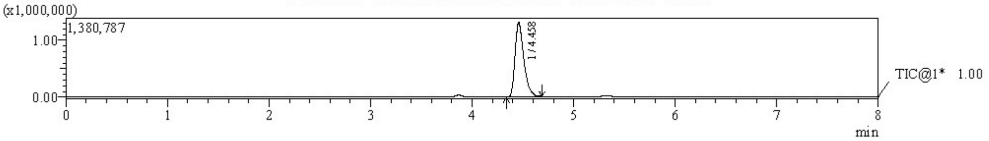

MS Spectrum Graph

#1 Ret.Time: Averaged 4.450-4.470(Scan#:446-448)
Mass Peaks:12 Base Peak:313.10(841573) Polarity:Pos Segment1 - Event1

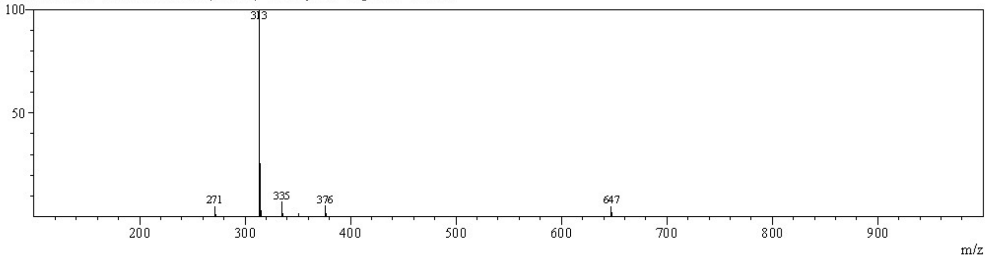

MS Spectrum Table

#:1 Ret.Time:
BG Mode:Calc 4.340<->4.690(435<->470)
Mass Peaks:12 Base Peak:313.10(841573) Polarity:Pos Segment1 - Event1

#	m/z	Abs.Inten.	Rel.Inten.	Charge	Polarity	Monoisotopic
1	271.05	40866	4.86			
2	272.05	8661	1.03			
3	313.10	841573	100.00			
4	314.10	213170	25.33			
5	315.10	22508	2.67			
6	335.10	58339	6.93			
7	336.10	10275	1.22			
8	351.10	11372	1.35			
9	376.10	42899	5.10			
10	377.15	12870	1.53			
11	647.25	40611	4.83			
12	648.35	13571	1.61			

Figure S104. LCMS spectrum of compound **34** (NPD-3377).

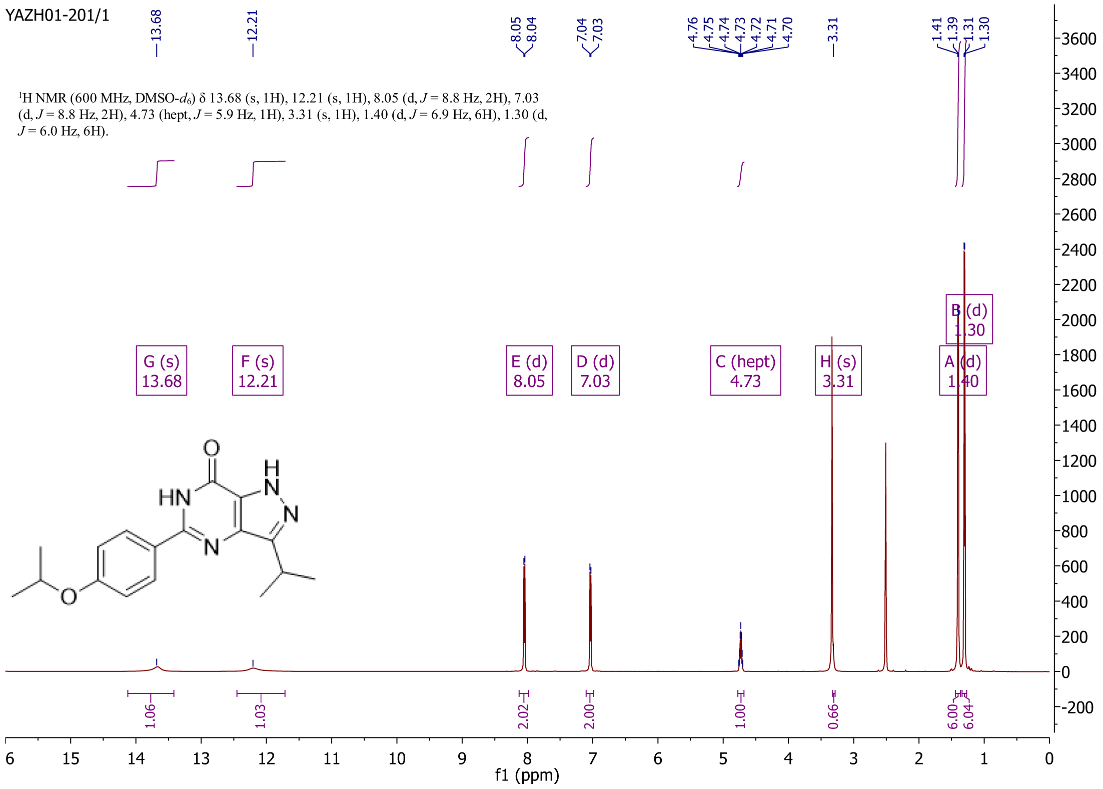

Figure S105. ¹H NMR spectrum of compound **34** (NPD-3377).

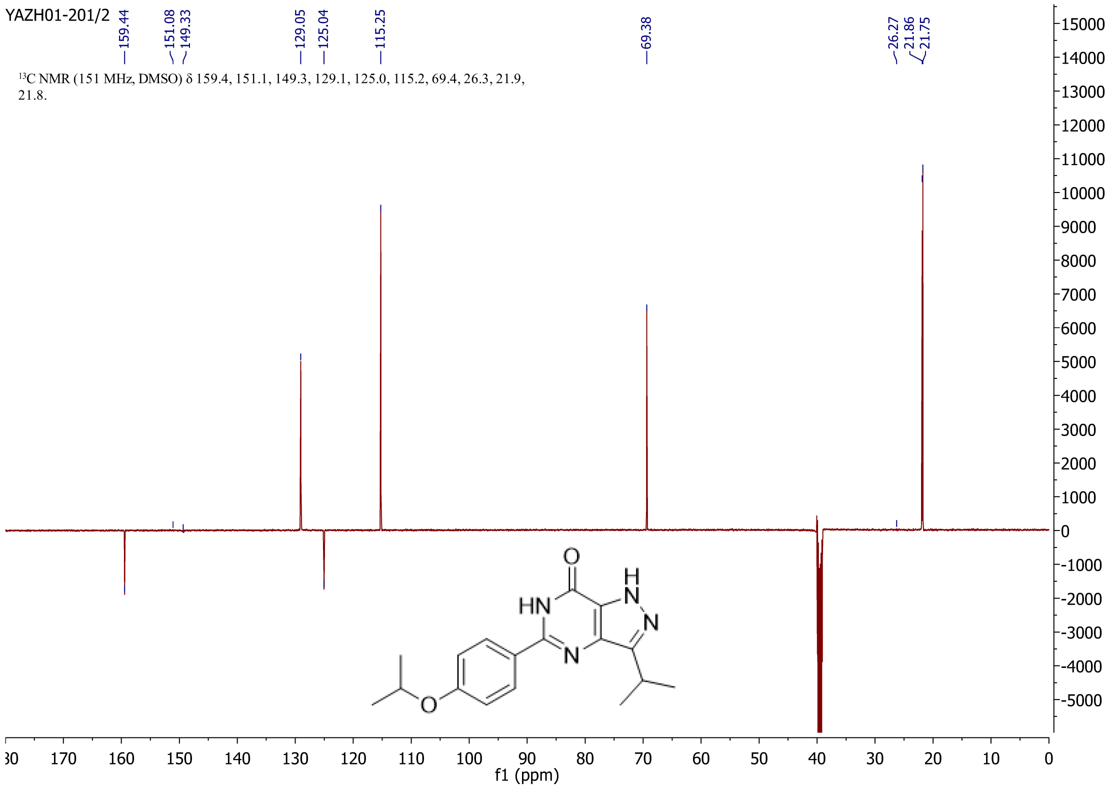

Figure S106. ¹³C NMR spectrum of compound **34** (NPD-3377).

Acquired by	: Admin
Date Acquired	: 7/27/2017 3:00:12 PM
Sample Name	: YAZH01-117
Sample ID	:
Tray#	: 1
Vial#	: 46
Injection Volume	: 10
Data File	: C:\LabSolutions\Data\2017\2017-wk30\YAZH01-117.lcd
Background File	: blanco 27072017.lcd
Method File	: Method SCAN ACID standard.lcm
Report Format	: DefaultLCMS.lcr
Tuning File	: C:\LabSolutions\Tuning\Tuning-ESI-pos-neg01072015a.lct
Processed by	: Admin
Modified Date	: 7/27/2017 3:39:01 PM

PDA Graph

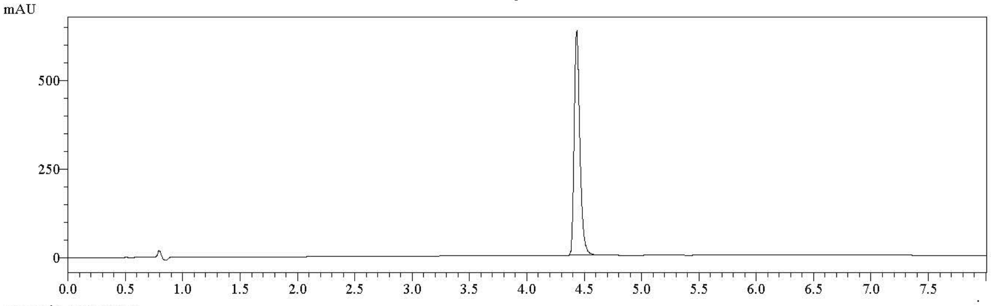

PDA Ch1 254nm 4nm

Peak#	Name	Ret. Time	Area	Area %
1		4.429	2182152	100.000

MS Spectrum Graph

#:1 Ret.Time:Averaged 4.480-4.500(Scan#:449-451)
Mass Peaks:9 Base Peak:323.10(1097653) Polarity:Pos Segment1 - Event1

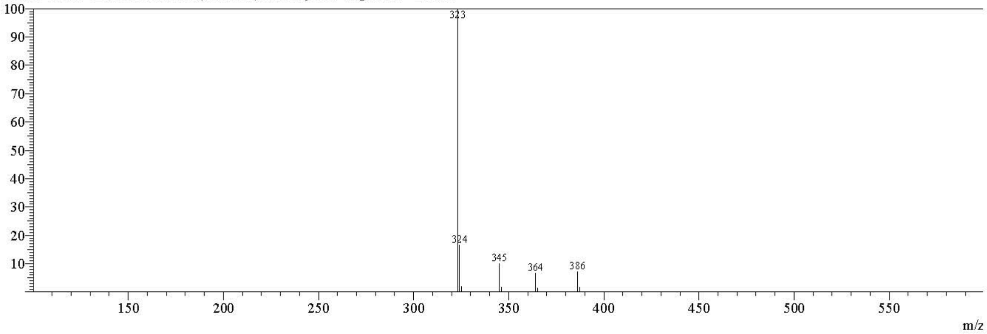

MS Spectrum Table

#:1 Ret.Time:
BG Mode:Calc 4.380<->4.740(439<->475)
Mass Peaks:9 Base Peak:323.10(1097653) Polarity:Pos Segment1 - Event1

#	m/z	Abs.Inten.	Rel.Inten.	Charge	Polarity	Monoisotopic
1	323.10	1097653	100.00			
2	324.15	181687	16.55			
3	325.05	20889	1.90			
4	345.05	108884	9.92			
5	346.15	18658	1.70			

#	m/z	Abs.Inten.	Rel.Inten.	Charge	Polarity	Monoisotopic
6	364.15	71298	6.50			
7	365.10	15570	1.42			
8	386.15	79321	7.23			
9	387.15	18237	1.66			

Figure S107. LCMS spectrum of compound **35** (NPD-3201).

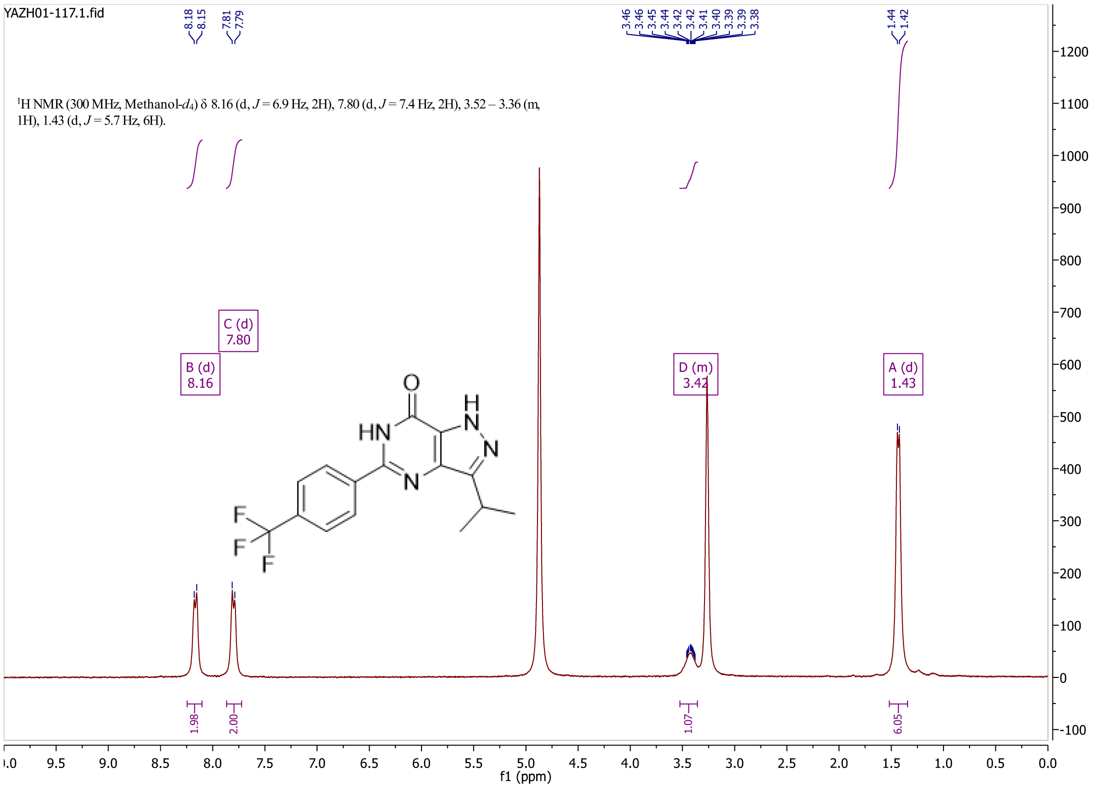

Figure S108. ^{1}H NMR spectrum of compound **35** (NPD-3201).

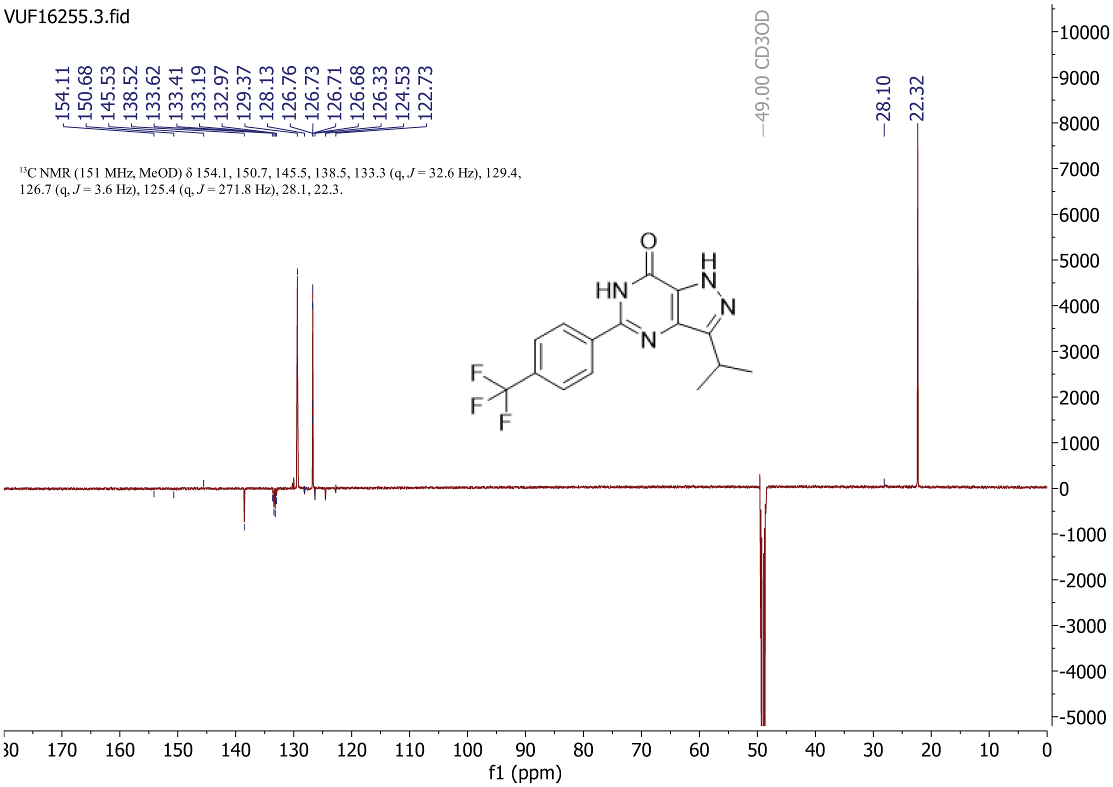

Figure S109. ^{13}C NMR spectrum of compound **35** (NPD-3201).

Acquired by	: Admin
Date Acquired	: 18/12/2018 4:08:05 PM
Sample Name	: SHGI02PROD
Sample ID	:
Tray#	: 1
Vial#	: 2
Injection Volume	: 3
Data File	: C:\LabSolutions\Data\2018\2018-wk51\SHGI02PROD.lcd
Background File	: blanco 18122018.lcd
Method File	: Method SCAN ACID standard.lcm
Report Format	: DefaultLCMS.lcr
Tuning File	: C:\LabSolutions\Tuning\Tuning-ESI-pos-neg01072015a.lct
Processed by	: Admin
Modified Date	: 18/12/2018 4:31:41 PM

Chromatogram
SHGI02PROD C:\LabSolutions\Data\2018\2018-wk51\SHGI02PROD.lcd

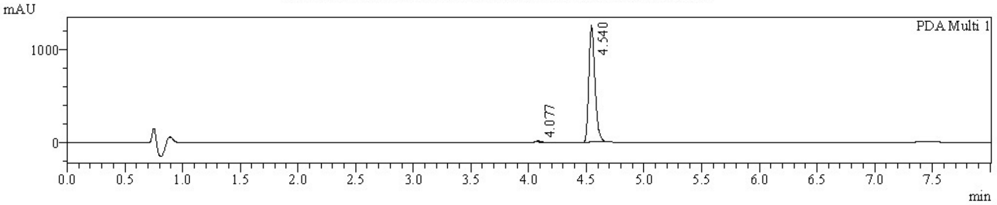

1 PDA Multi 1 / 230nm 4nm

PeakTabl

PDA Ch1 230nm 4nm

Peak#	Name	Ret. Time	Area	Area %
1		4.077	30020	0.658
2		4.540	4534064	99.342

MS Chromatogram
SHGI02PROD C:\LabSolutions\Data\2018\2018-wk51\SHGI02PROD.lcd

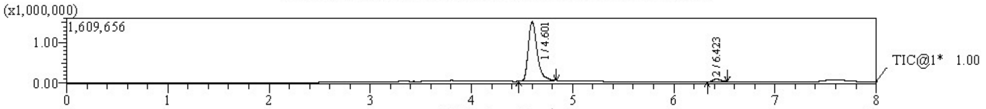

MS Spectrum Graph

#:1 Ret.Time:Averaged 4.590-4.610(Scan#:460-462)
Mass Peaks:9 Base Peak:339.05(1009719) Polarity:Pos Segment1 - Event1

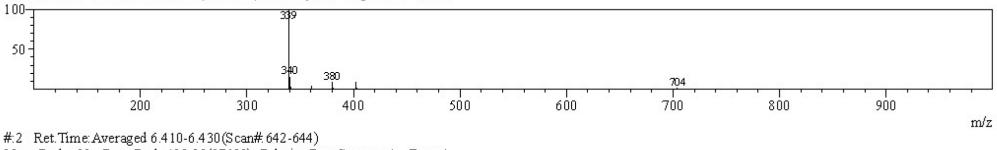

#:2 Ret.Time:Averaged 6.410-6.430(Scan#:642-644)
Mass Peaks:28 Base Peak:420.35(27693) Polarity:Pos Segment1 - Event1

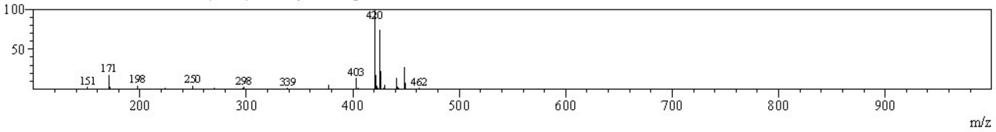

MS Spectrum Table

#:1 Ret.Time:
BG Mode:Calc 4.470<->4.840(448<->485)
Mass Peaks:9 Base Peak:339.05(1009719) Polarity:Pos Segment1 - Event1

#	m/z	Abs.Inten.	Rel.Inten.	Charge	Polarity	Monoisotopic	#	m/z	Abs.Inten.	Rel.Inten.	Charge	Polarity	Monoisotopic
1	339.05	1009719	100.00				4	361.05	44019	4.36			
2	340.05	154089	15.26				5	380.10	89234	8.84			
3	341.05	24179	2.39				6	381.10	17828	1.77			

Figure S110. LCMS spectrum of compound **36** (NPD-3597).

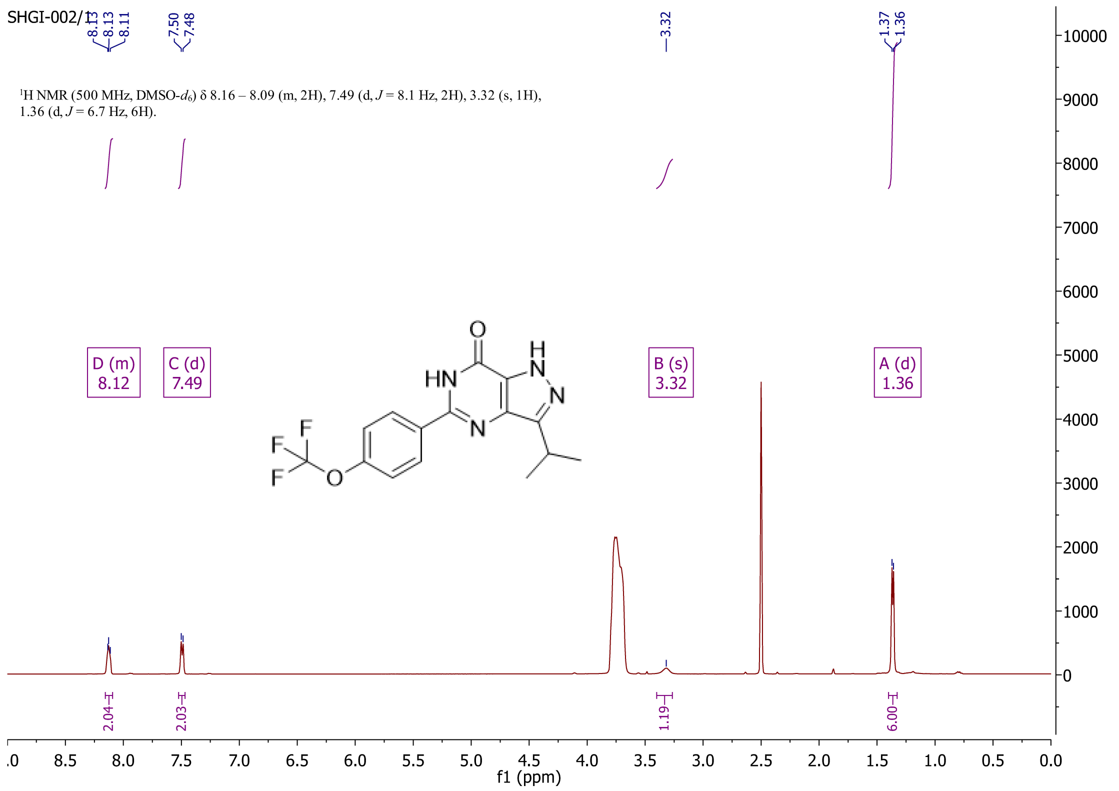

Figure S111. ^{1}H NMR spectrum of compound **36** (NPD-3597).

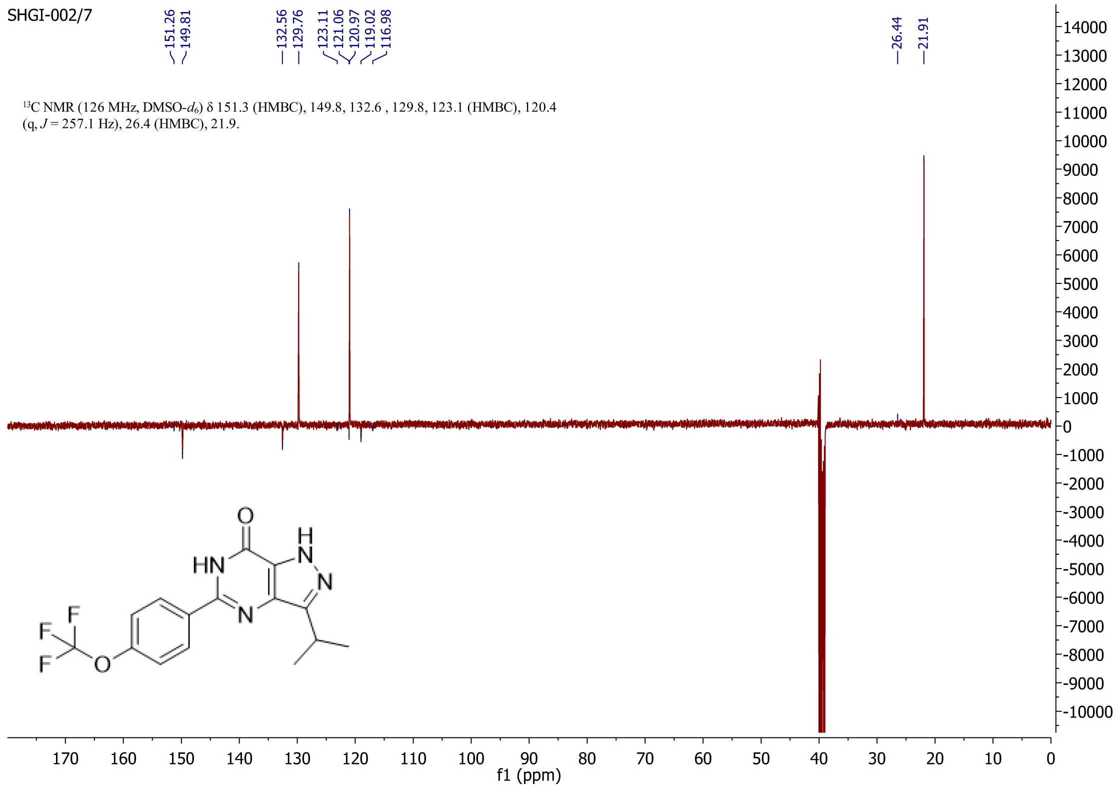

Figure S112. ^{13}C NMR spectrum of compound **36** (NPD-3597).

Acquired by	: Admin
Date Acquired	: 7/27/2017 3:08:48 PM
Sample Name	: YAZH01-119
Sample ID	:
Tray#	: 1
Vial#	: 47
Injection Volume	: 10
Data File	: C:\LabSolutions\Data\2017\2017-wk30\YAZH01-119.lcd
Background File	: blanco 27072017.lcd
Method File	: Method SCAN ACID standard.lcm
Report Format	: DefaultLCMS.lcr
Tuning File	: C:\LabSolutions\Tuning\Tuning-ESI-pos-neg01072015a.lct
Processed by	: Admin
Modified Date	: 7/27/2017 3:39:58 PM

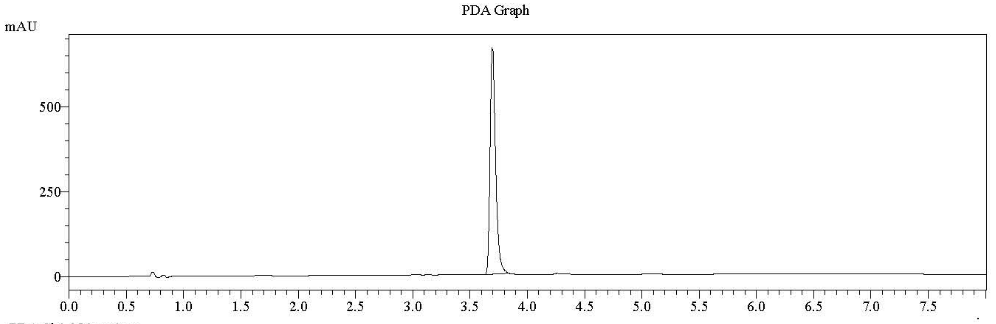

PDA Ch1 254nm 4nm

Peak#	Name	Ret. Time	Area	Area %
1		3.690	2247543	100.000

MS Spectrum Graph

#:1 Ret.Time:Averaged 3.740-3.760(Scan#:375-377)

Mass Peaks:11 Base Peak:280.10(244816) Polarity:Pos Segment1 - Event1

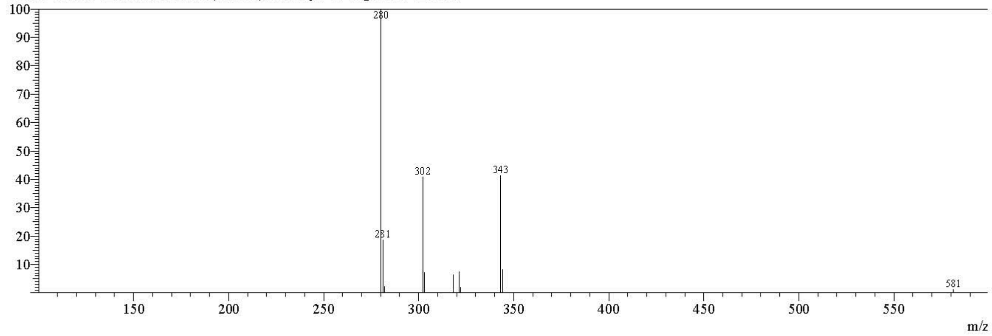

MS Spectrum Table

#:1 Ret.Time:

BG Mode:Calc 3.640<->3.950(365<->396)

Mass Peaks:11 Base Peak:280.10(244816) Polarity:Pos Segment1 - Event1

#	m/z	Abs.Inten.	Rel.Inten.	Charge	Polarity	Monoisotopic	#	m/z	Abs.Inten.	Rel.Inten.	Charge	Polarity	Monoisotopic
1	280.10	244816	100.00				7	321.15	17840	7.29			
2	281.10	45449	18.56				8	322.15	4426	1.81			
3	282.00	5095	2.08				9	343.15	100780	41.17			
4	302.10	99961	40.83				10	344.10	20274	8.28			
5	303.05	17719	7.24				11	581.25	3092	1.26			
6	318.10	15304	6.25										

Figure S113. LCMS spectrum of compound **37** (NPD-3203).

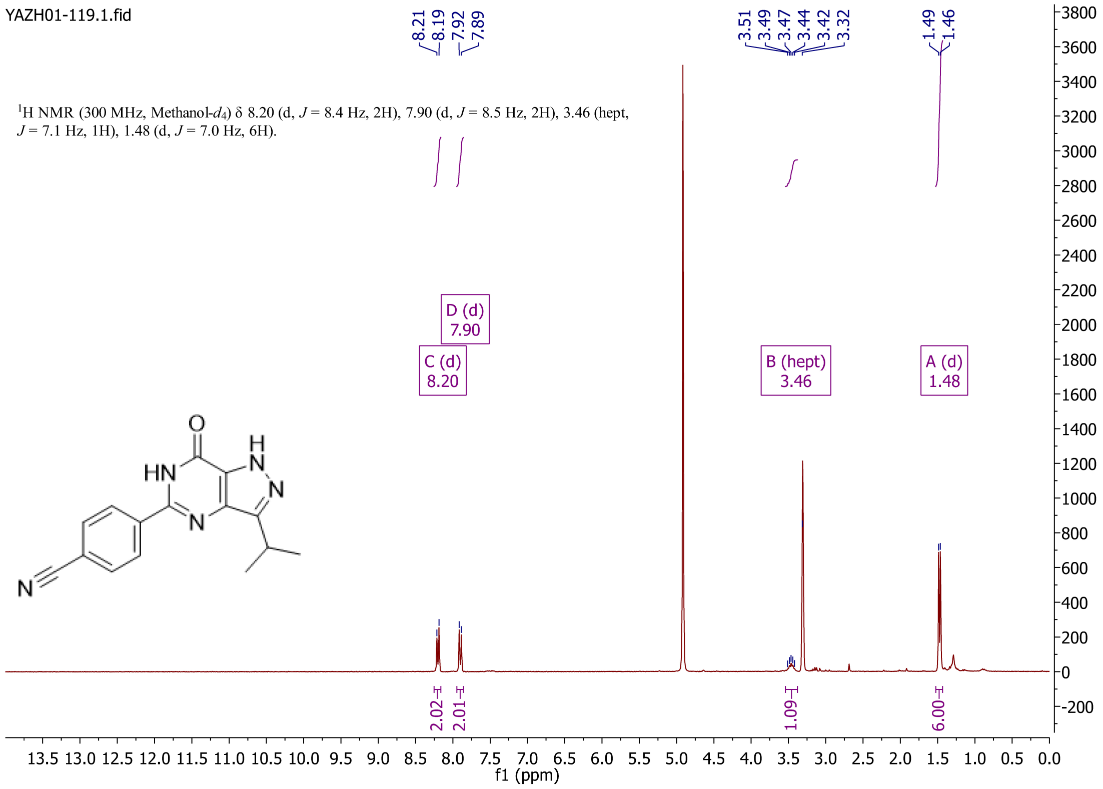

Figure S114. ^{1}H NMR spectrum of compound **37** (NPD-3203).

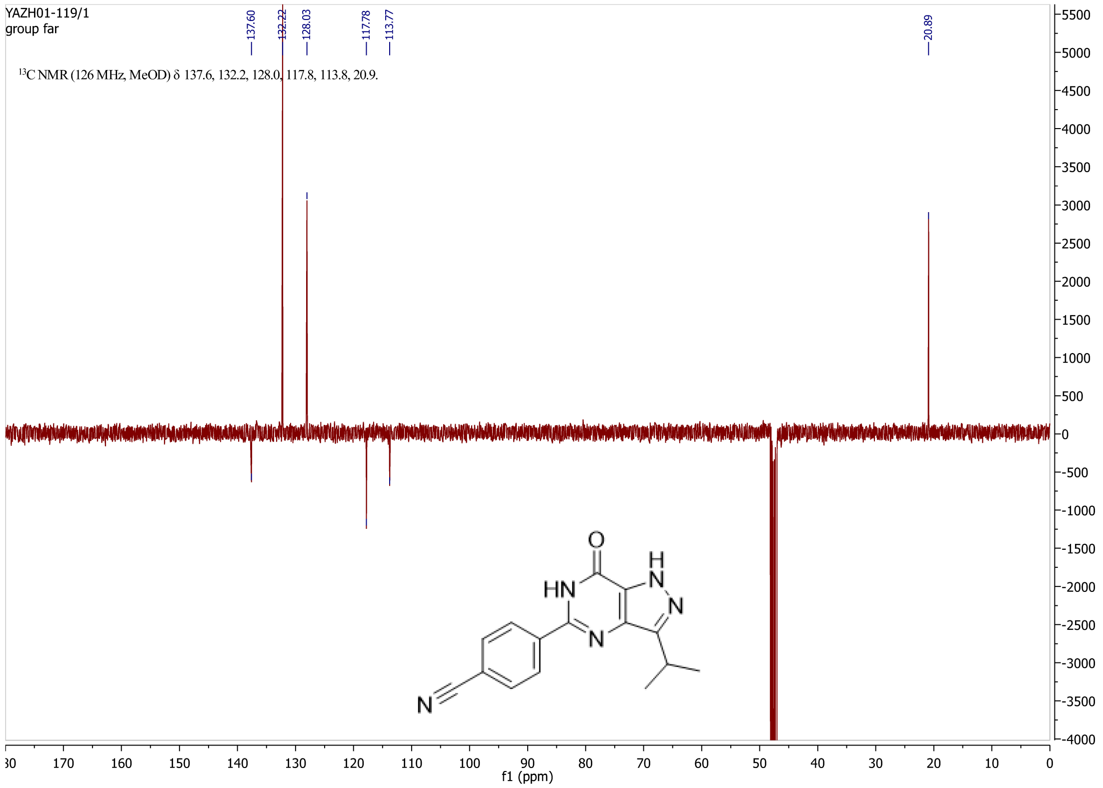

Figure S115. ^{13}C NMR spectrum of compound **37** (NPD-3203).

Acquired by : Admin
Date Acquired : 13/3/2018 3:55:15 PM
Sample Name : YAZH01-185
Sample ID :
Tray# : 1
Vial# : 17
Injection Volume : 3
Data File : C:\LabSolutions\Data\2018\2018-wk11\YAZH01-185.lcd
Background File : blanco_130318.lcd
Method File : Method SCAN ACID standard.lcm
Report Format : DefaultLCMS.lcr
Tuning File : C:\LabSolutions\Tuning\Tuning-ESI-pos-neg01072015a.lct
Processed by : Admin
Modified Date : 10/12/2020 3:34:19 PM

PDA Graph

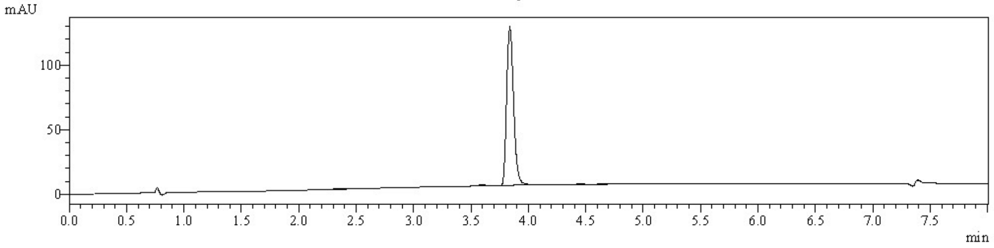

PeakTable

PDA Ch1 254nm 4nm

Peak#	Name	Ret. Time	Area	Area%
1		2.336	2622	0.496
2		2.611	990	0.187
3		3.189	795	0.151
4		3.590	3610	0.683
5		3.833	510440	96.587
6		4.450	3120	0.590
7		4.662	4106	0.777
8		4.923	1563	0.296
9		5.150	1231	0.233

MS Chromatogram
YAZH01-185 C:\LabSolutions\Data\2018\2018-wk11-20\2018-wk11\YAZH01-185.lcd

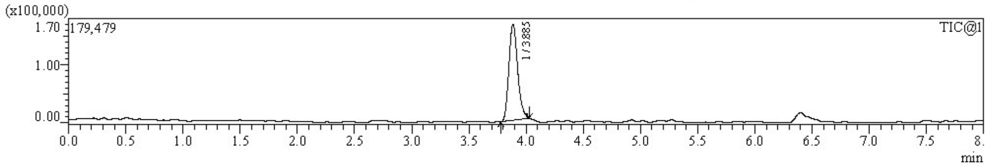

MS Spectrum Graph

#1 Ret.Time:Averaged 3.880-3.900(Scan#:389-391)
BG Mode:Calc 3.780<->4.030(379<->404)
Mass Peaks:12 Base Peak:313.10(118613) Polarity:Pos Segment1 - Event1

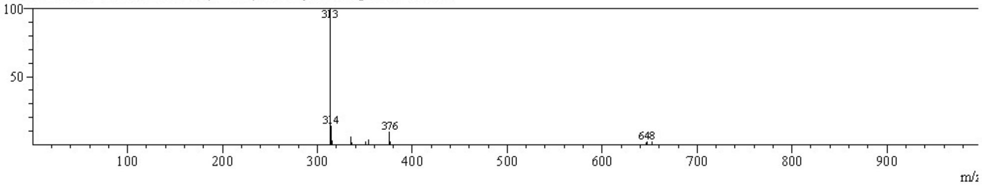

MS Spectrum Table

#:1 Ret.Time:
BG Mode:Calc 3.780<->4.030(379<->404)
Mass Peaks:12 Base Peak:313.10(118613) Polarity:Pos Segment1 - Event1

#	m/z	Abs.Inten.	Rel.Inten.	Charge	Polarity	Monoisotopic
1	313.10	118613	100.00			
2	314.10	16180	13.64			
3	315.20	3438	2.90			
4	335.10	6284	5.30			
5	336.15	1210	1.02			
6	351.05	2625	2.21			
7	354.10	3980	3.36			
8	376.05	11130	9.38			
9	377.15	2090	1.76			
10	646.65	1534	1.29			
11	647.65	2013	1.70			
12	652.65	2544	2.14			

Figure S116. LCMS spectrum of compound **38** (NPD-3305).

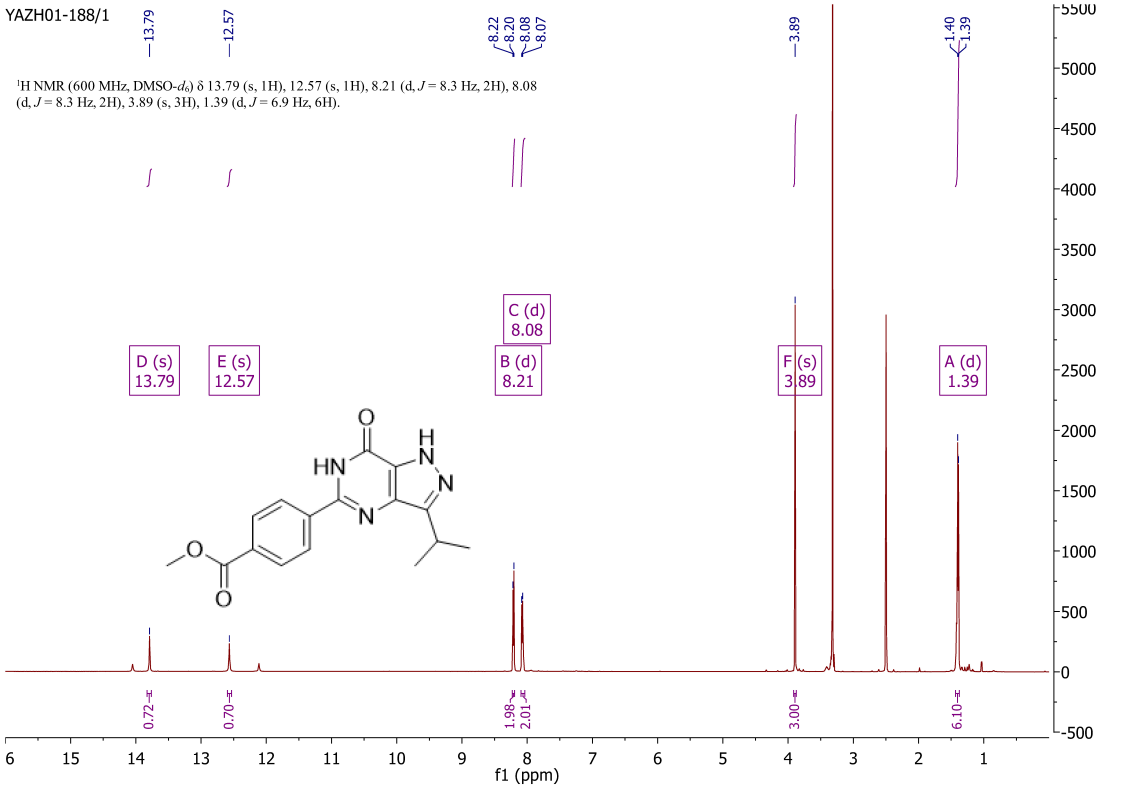

Figure S117. ^{1}H NMR spectrum of compound **38** (NPD-3305).

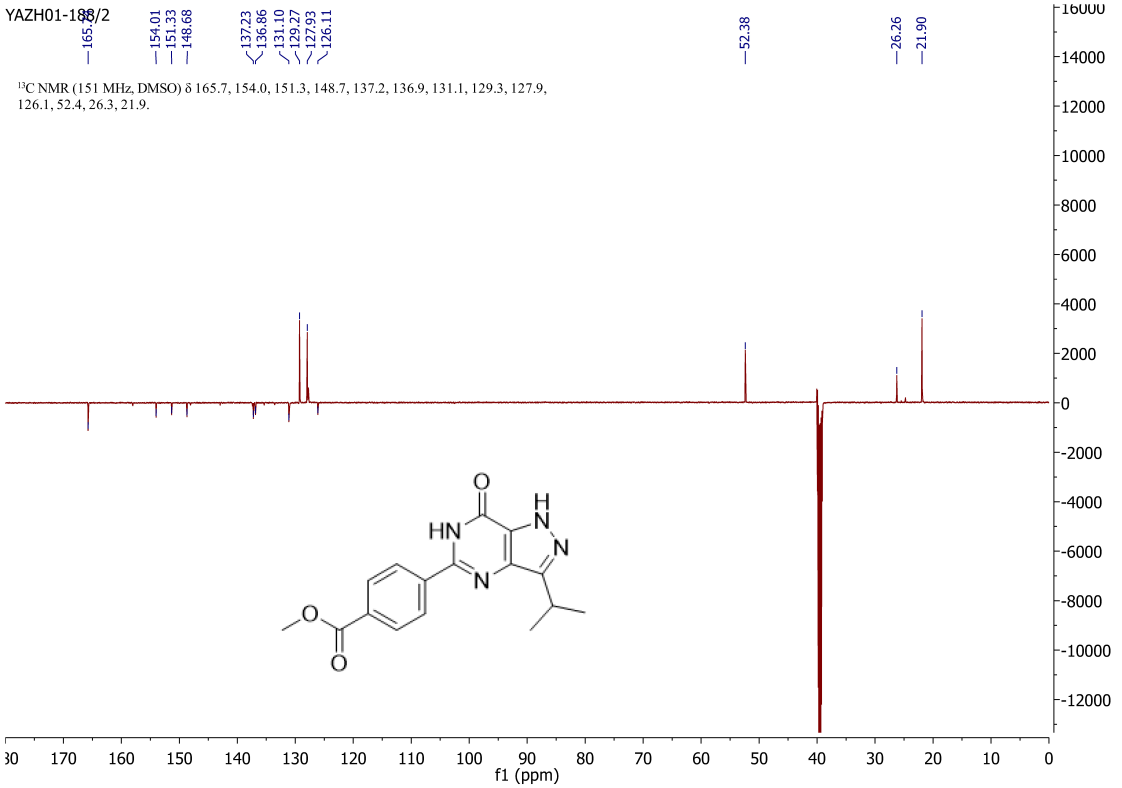

Figure S118. ^{13}C NMR spectrum of compound **38** (NPD-3305).

Acquired by	: Admin
Date Acquired	: 7/5/2018 1:38:34 PM
Sample Name	: YAZH01-211
Sample ID	:
Tray#	: 1
Vial#	: 26
Injection Volume	: 3
Data File	: C:\LabSolutions\Data\2018\2018-wk19\YAZH01-211.lcd
Background File	: blanco_070518.lcd
Method File	: Method SCAN ACID standard.lcm
Report Format	: DefaultLCMS.lcr
Tuning File	: C:\LabSolutions\Tuning\Tuning-ESI-pos-neg01072015a.lct
Processed by	: Admin
Modified Date	: 7/5/2018 1:56:05 PM

PDA Graph

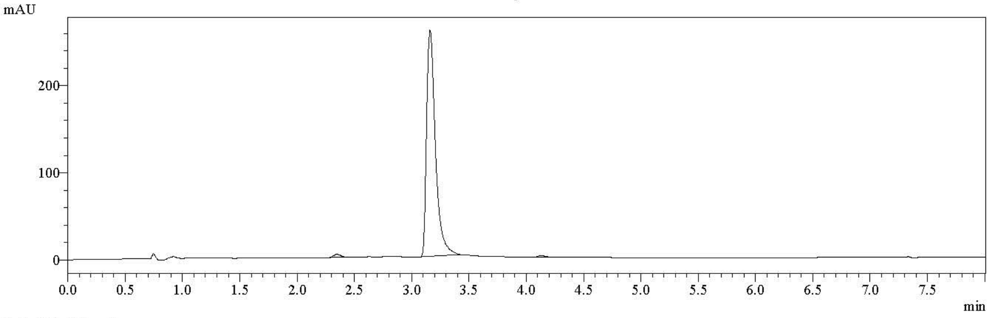

PDA Ch1 254nm 4nm

Peak#	Name	Ret. Time	Area	Area %
1		2.343	14270	1.014
2		3.156	1386801	98.514
3		4.125	6642	0.472

MS Spectrum Graph

#:1 Ret.Time:Averaged 3.220-3.240(Scan#:323-325)
Mass Peaks:16 Base Peak:299.10(86446) Polarity:Pos Segment1 - Event1

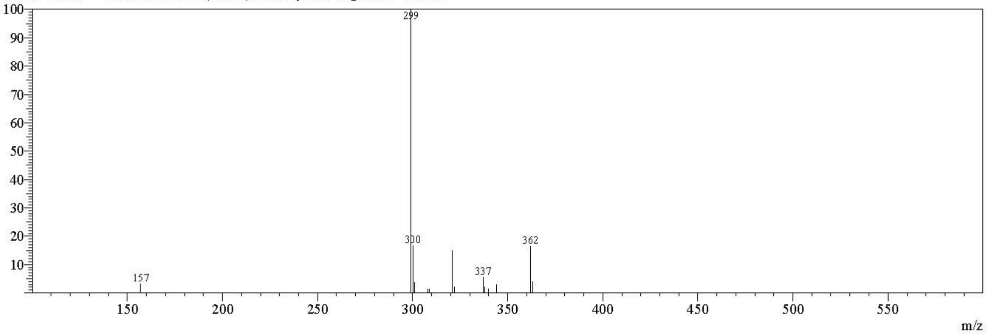

MS Spectrum Table

#:1 Ret.Time:
BG Mode:Calc 3.110<->3.470(312<->348)
Mass Peaks:16 Base Peak:299.10(86446) Polarity:Pos Segment1 - Event1

#	m/z	Abs.Inten.	Rel.Inten.	Charge	Polarity	Monoisotopic
1	157.00	2652	3.07			
2	299.10	86446	100.00			
3	300.05	14497	16.77			
4	300.95	3158	3.65			
5	307.95	1193	1.38			
6	308.95	1150	1.33			
7	321.00	12799	14.81			
8	321.95	1861	2.15			

#	m/z	Abs.Inten.	Rel.Inten.	Charge	Polarity	Monoisotopic
9	337.00	4685	5.42			
10	338.00	1800	2.08			
11	340.00	1203	1.39			
12	344.00	2475	2.86			
13	362.05	14214	16.44			
14	363.15	3410	3.94			
15	619.10	5256	6.08			
16	619.65	2539	2.94			

Figure S119. LCMS spectrum of compound **39** (NPD-3489).

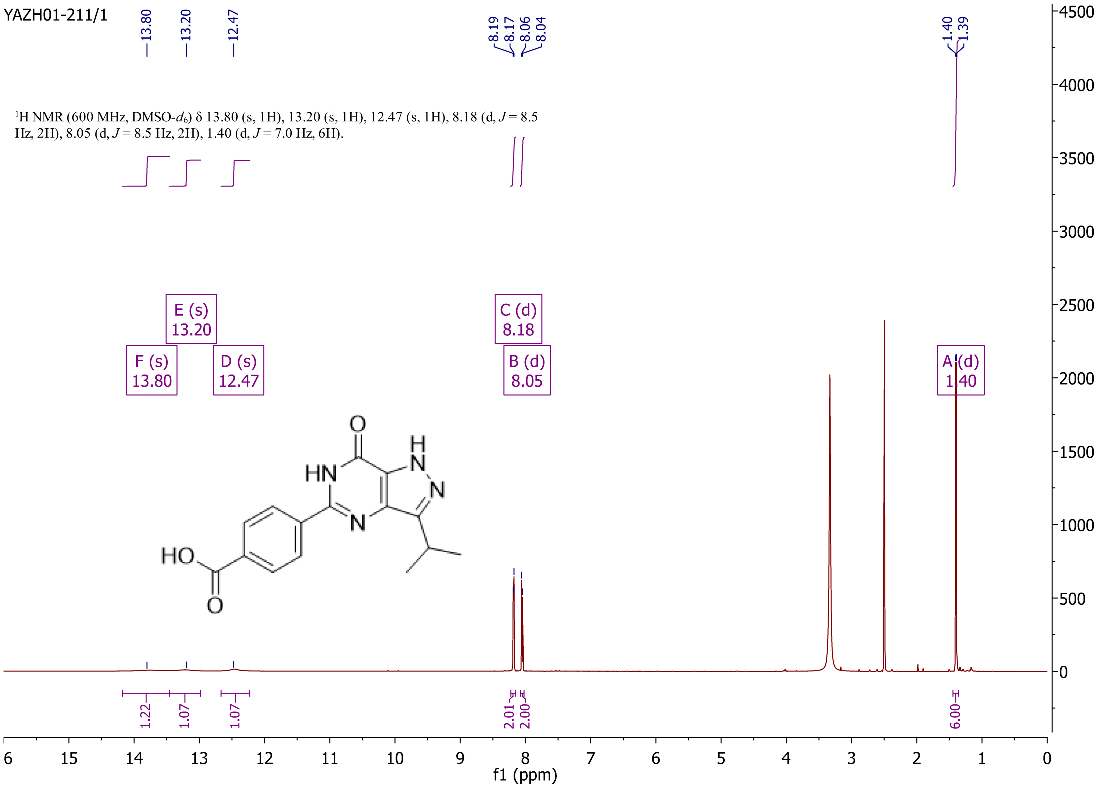

Figure S120. ^{1}H NMR spectrum of compound **39** (NPD-3489).

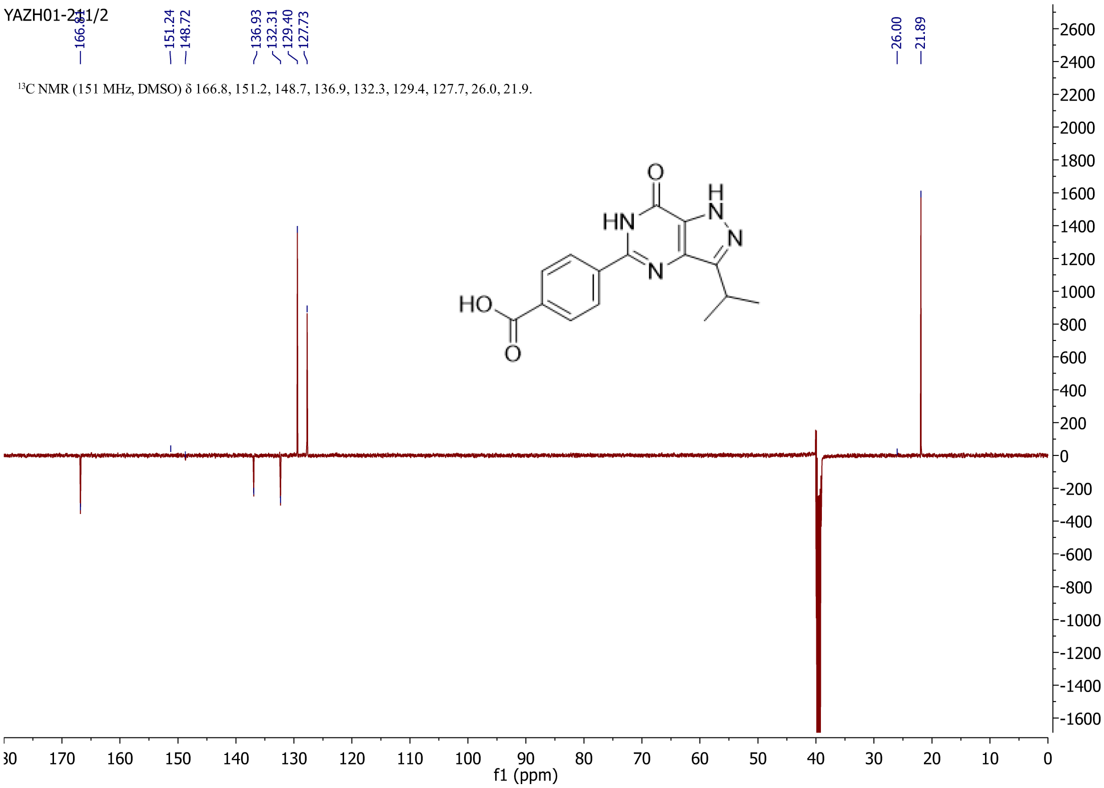

Figure S121. ^{13}C NMR spectrum of compound **39** (NPD-3489).

Acquired by	: Admin
Date Acquired	: 22/3/2018 3:18:17 PM
Sample Name	: YAZH01-190
Sample ID	:
Tray#	: 1
Vial#	: 17
Injection Volume	: 5
Data File	: C:\LabSolutions\Data\2018\2018-wk12\YAZH01-190.lcd
Background File	: blanco 22032018.lcd
Method File	: Method SCAN ACID standard.lcm
Report Format	: DefaultLCMS.lcr
Tuning File	: C:\LabSolutions\Tuning\Tuning-ESI-pos-neg01072015a.lct
Processed by	: Admin
Modified Date	: 22/3/2018 4:02:43 PM

Chromatogram
YAZH01-190 C:\LabSolutions\Data\2018\2018-wk12\YAZH01-190.lcd

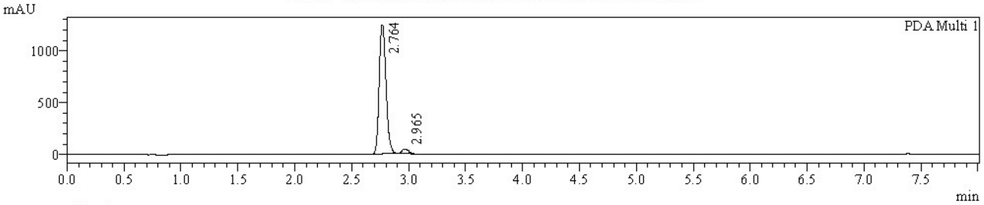

1 PDA Multi 1 / 254nm 4nm

PeakTable

PDA Ch1 254nm 4nm

Peak#	Name	Ret. Time	Area	Area %
1		2.764	5168702	96.904
2		2.965	165149	3.096

MS Chromatogram
YAZH01-190 C:\LabSolutions\Data\2018\2018-wk12\YAZH01-190.lcd

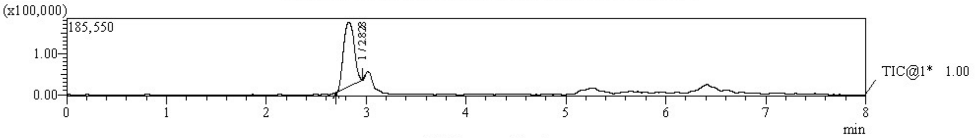

MS Spectrum Graph

#1 Ret.Time:Averaged 2.820-2.840(Scan#:283-285)
Mass Peaks:26 Base Peak:298.05(48235) Polarity:Pos Segment1 - Event1

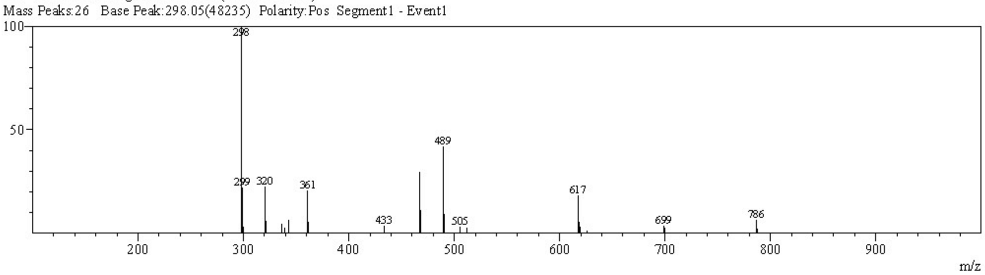

MS Spectrum Table

#:1 Ret.Time:
BG Mode:Calc 2.700<->2.960(271<->297)
Mass Peaks:26 Base Peak:298.05(48235) Polarity:Pos Segment1 - Event1

#	m/z	Abs.Inten.	Rel.Inten.	Charge	Polarity	Monoisotopic
1	298.05	48235	100.00			
2	299.15	10409	21.58			
3	300.00	1283	2.66			
4	320.05	10668	22.12			
5	321.10	2797	5.80			
6	336.10	1974	4.09			
7	339.05	1130	2.34			
8	343.05	3088	6.40			
9	361.10	9883	20.49			
10	362.05	2614	5.42			
11	433.20	1585	3.29			
12	467.20	14196	29.43			
13	468.10	5260	10.90			
14	489.25	20085	41.64			
15	490.10	4324	8.96			
16	505.25	1348	2.79			

Figure S122. LCMS spectrum of compound **40** (NPD-3371).

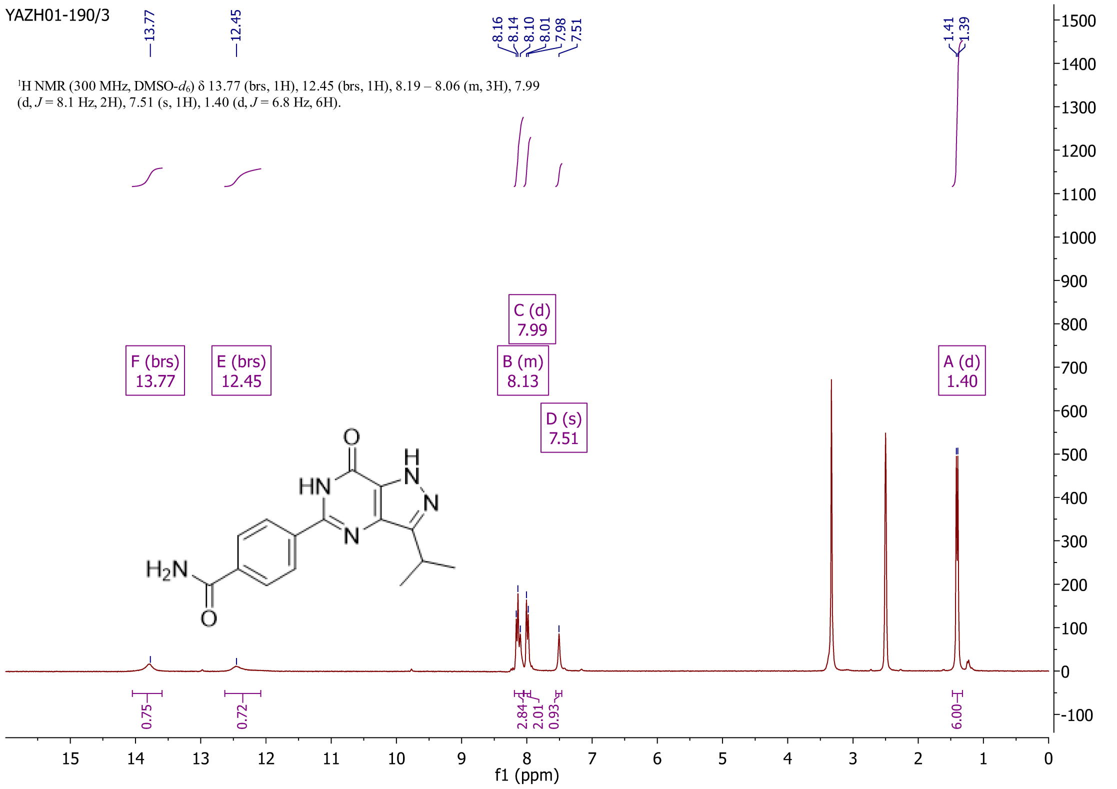

Figure S123. ^{1}H NMR spectrum of compound **40** (NPD-3371).

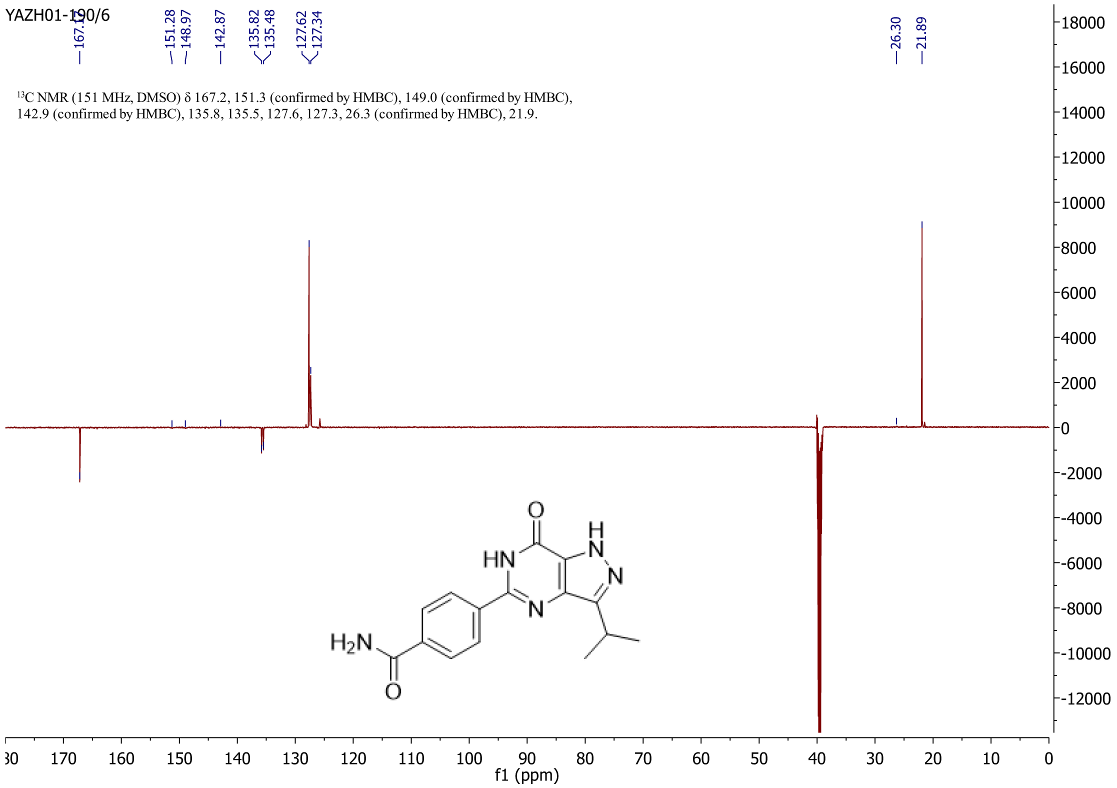

Figure S124. ^{13}C NMR spectrum of compound **40** (NPD-3371).

Acquired by	: Admin
Date Acquired	: 2/28/2023 5:02:29 PM
Sample Name	: VUF16558 YAZH01-199
Sample ID	:
Tray#	: 1
Vial#	: 21
Injection Volume	: 3
Data File	: C:\LabSolutions\Data\2023\2023-wk09\VUF16558 YAZH01-199.lcd
Background File	: blanco 28022023.lcd
Method File	: Method SCAN ACID standard.lcm
Report Format	: DefaultLCMS.lcr
Tuning File	: C:\LabSolutions\Tuning File\Tuning-ESI-pos-neg01072015.lct
Processed by	: Admin
Modified Date	: 2/28/2023 5:11:14 PM

Chromatogram
VUF16558 YAZH01-199 C:\LabSolutions\Data\2023\2023-wk09\VUF16558 YAZH01-199.lcd

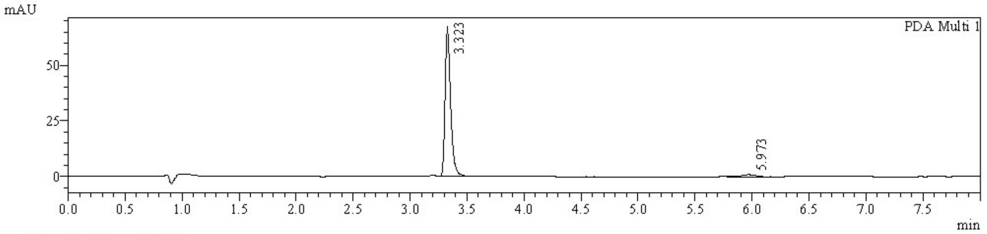

1 PDA Multi 1 / 254nm 4nm

PeakTable

PDA Ch1 254nm 4nm

Peak#	Ret. Time	Area	Height	Name	Area %
1	3.323	221821	67257		96.808
2	5.973	7314	917		3.192
Total		229135	68174		100.000

MS Chromatogram
VUF16558 YAZH01-199 C:\LabSolutions\Data\2023\2023-wk09\VUF16558 YAZH01-199.lcd

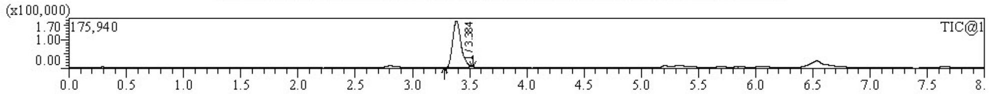

MS Spectrum Graph

#1 Ret.Time:Averaged 3.370-3.390(Scan#:338-340)
BG Mode:Calc 3.280<->3.530(329<->354)
Mass Peaks:12 Base Peak:333.00(115366) Polarity:Pos Segment1 - Event1

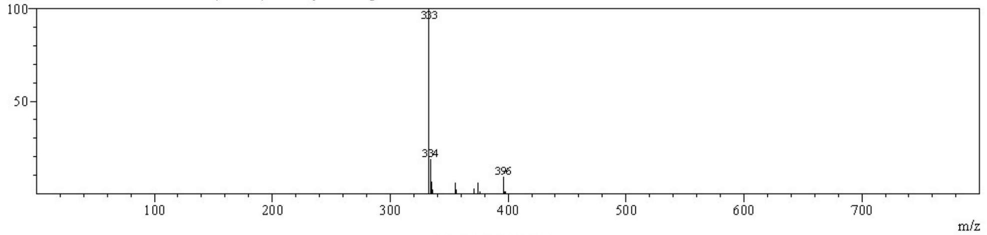

MS Spectrum Table

#1 Ret.Time:
BG Mode:Calc 3.280<->3.530(329<->354)
Mass Peaks:12 Base Peak:333.00(115366) Polarity:Pos Segment1 - Event

#	m/z	Abs.Inten.	Rel.Inten.	Charge	Polarity	Monoisotopic	#	m/z	Abs.Inten.	Rel.Inten.	Charge	Polarity	Monoisotopic
1	333.00	115366	100.00				7	371.10	2885	2.50			
2	334.05	21107	18.30				8	374.10	6548	5.68			
3	335.00	7305	6.33				9	376.15	1274	1.10			
4	336.00	2035	1.76				10	396.00	9968	8.64			
5	355.05	6657	5.77				11	397.05	1283	1.11			
6	356.20	2321	2.01				12	398.05	1174	1.02			

Figure S125. LCMS spectrum of compound **41** (NPD-3376).

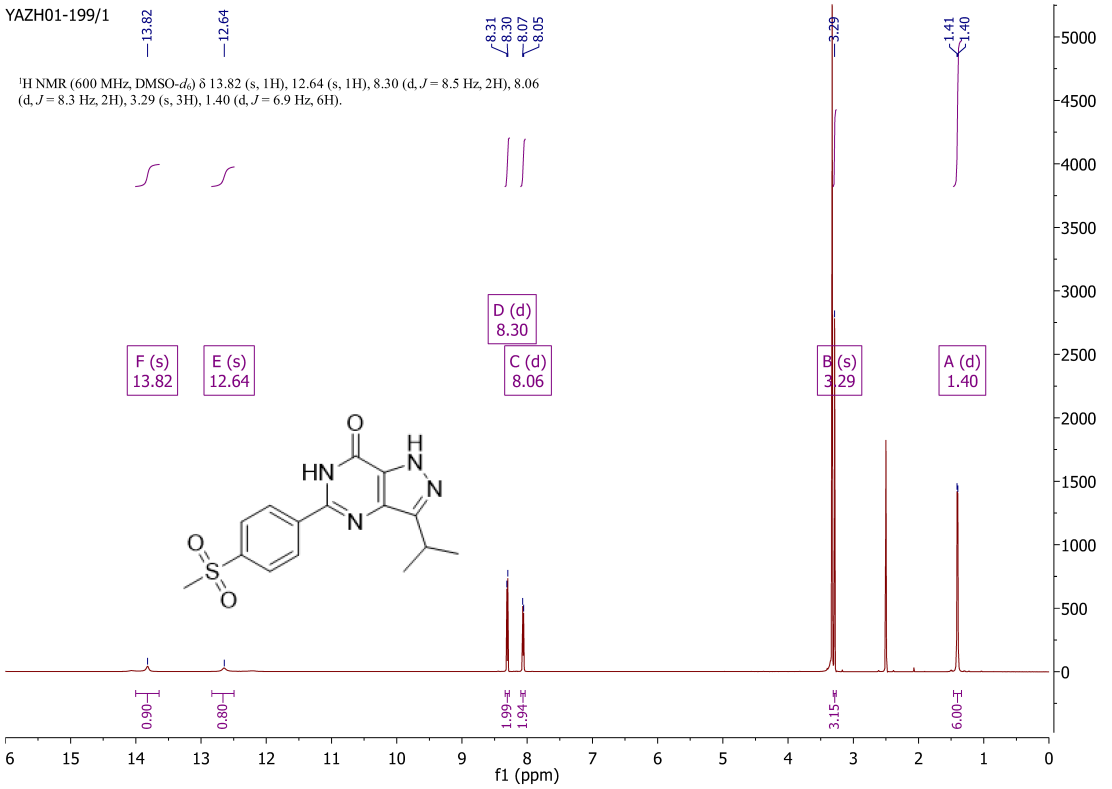

Figure S126. ^{1}H NMR spectrum of compound **41** (NPD-3376).

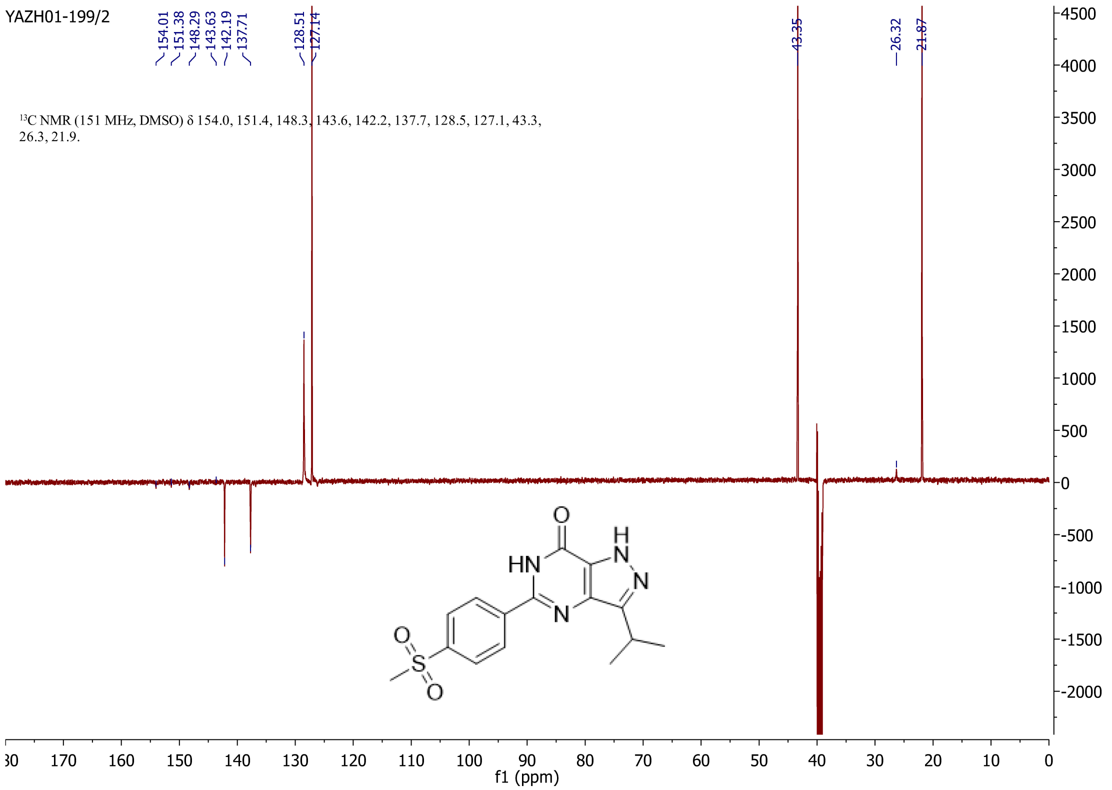

Figure S127. ^{13}C NMR spectrum of compound **41** (NPD-3376).

Acquired by	: Admin
Date Acquired	: 21/3/2018 5:07:49 PM
Sample Name	: YAZH01-191-4
Sample ID	:
Tray#	: 1
Vial#	: 20
Injection Volume	: 10
Data File	: C:\LabSolutions\Data\2018\2018-wk12\YAZH01-191-4.lcd
Background File	: blanco 21032018.lcd
Method File	: Method SCAN ACID standard.lcm
Report Format	: DefaultLCMS.lcr
Tuning File	: C:\LabSolutions\Tuning\Tuning-ESI-pos-neg01072015a.lct
Processed by	: Admin
Modified Date	: 22/3/2018 2:23:15 PM

PDA Graph

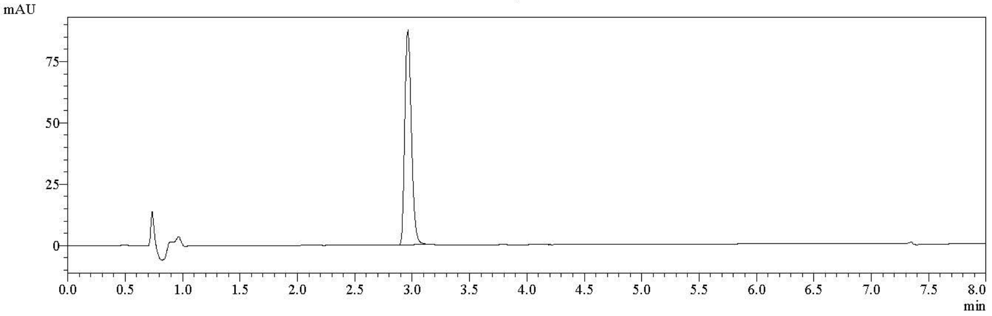

PDA Ch1 254nm 4nm

Peak#	Name	Ret. Time	Area	Area%
1		2.957	347658	100.000

MS Spectrum Graph

#:1 Ret.Time:Averaged 3.010-3.030(Scan#:302-304)
Mass Peaks:9 Base Peak:334.05(60255) Polarity:Pos Segment1 - Event1

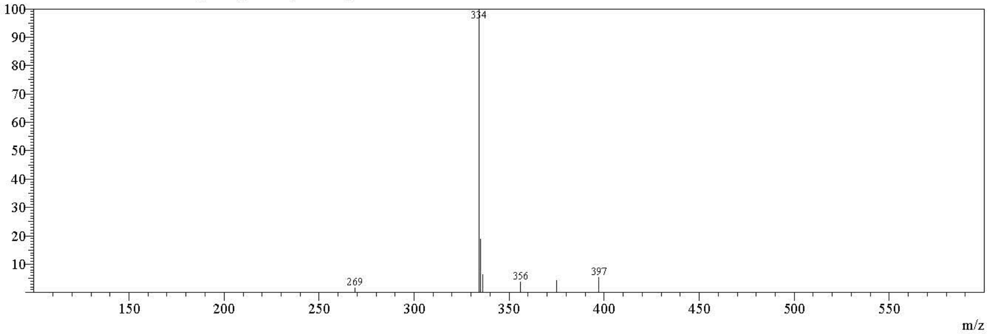

MS Spectrum Table

#:1 Ret.Time:
BG Mode:Calc 2.930<->3.140(294<->315)
Mass Peaks:9 Base Peak:334.05(60255) Polarity:Pos Segment1 - Event1

#	m/z	Abs.Inten.	Rel.Inten.	Charge	Polarity	Monoisotopic
1	268.85	1014	1.68			
2	334.05	60255	100.00			
3	335.00	11294	18.74			
4	336.00	3852	6.39			
5	356.00	2253	3.74			
6	375.00	2588	4.30			
7	397.15	3201	5.31			
8	686.70	734	1.22			
9	855.25	1087	1.80			

Figure S128. LCMS spectrum of compound **42** (NPD-3372).

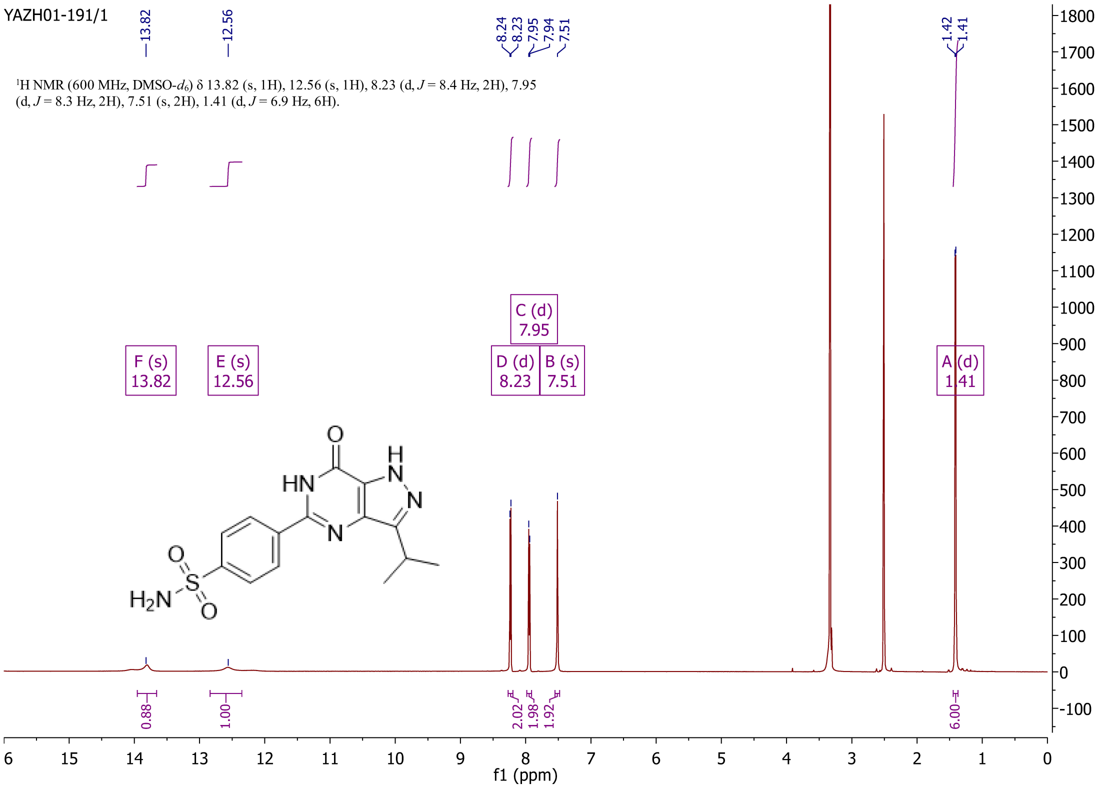

Figure S129. ^{1}H NMR spectrum of compound **42** (NPD-3372).

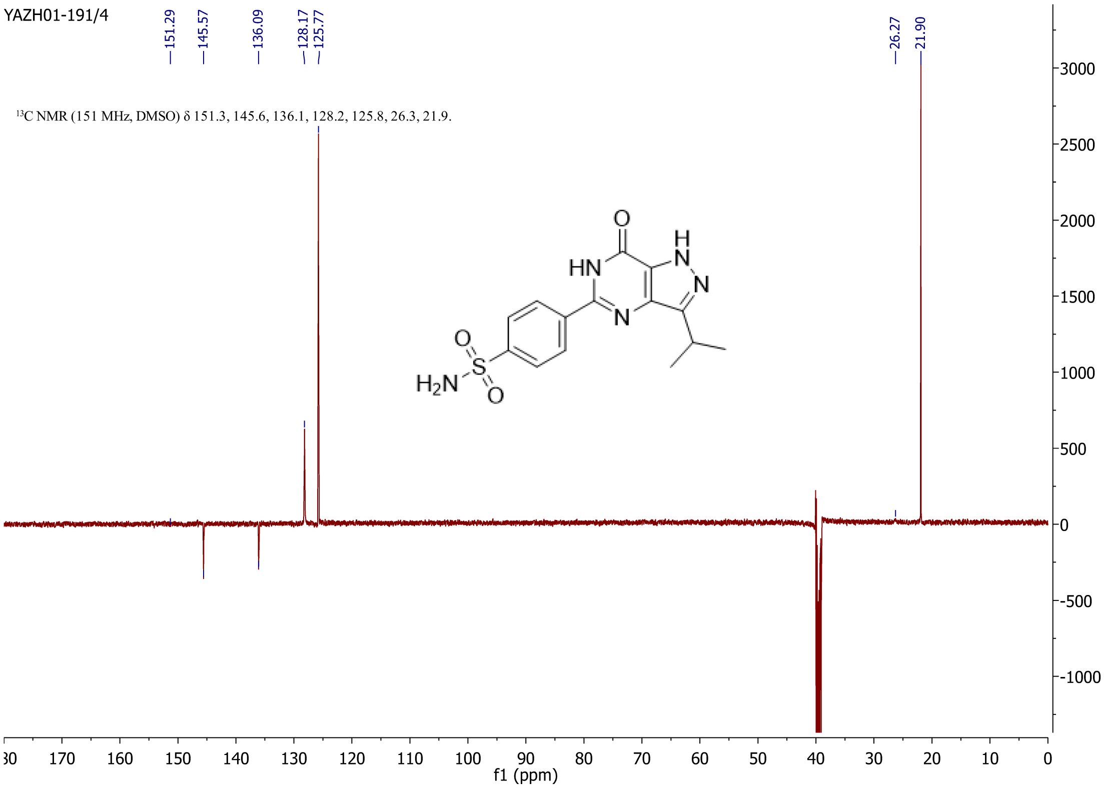

Figure S130. ^{13}C NMR spectrum of compound **42** (NPD-3372).

Acquired by	: Admin
Date Acquired	: 14/12/2017 10:02:22 AM
Sample Name	: YAZH01-176-2
Sample ID	:
Tray#	: 1
Vial#	: 9
Injection Volume	: 4
Data File	: C:\LabSolutions\Data\2017\2017-wk50\YAZH01-176-2.lcd
Background File	: blanco 14122017.lcd
Method File	: Method SCAN ACID standard.lcm
Report Format	: DefaultLCMS.lcr
Tuning File	: C:\LabSolutions\Tuning\Tuning-ESI-pos-neg01072015a.lct
Processed by	: Admin
Modified Date	: 14/12/2017 10:54:42 AM

Chromatogram
YAZH01-176-2 C:\LabSolutions\Data\2017\2017-wk50\YAZH01-176-2.lcd

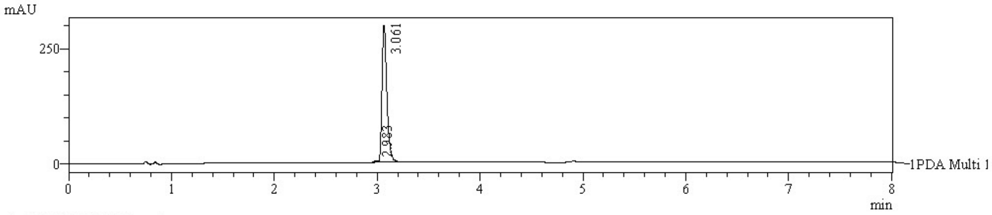

1 PDA Multi 1 / 254nm 4nm
PDA Ch1 254nm 4nm

Peak#	Name	Ret. Time	Area	Area%
1		2.983	6290	0.640
2		3.061	977114	99.360

MS Chromatogram
YAZH01-176-2 C:\LabSolutions\Data\2017\2017-wk50\YAZH01-176-2.lcd

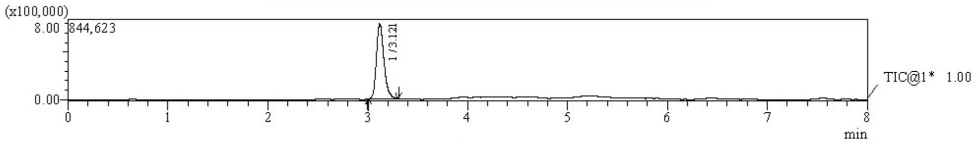

MS Spectrum Graph
#1 Ret.Time:Averaged 3.110-3.130(Scan#:312-314)
Mass Peaks:7 Base Peak:312.10(593218) Polarity:Pos Segment1 - Event1

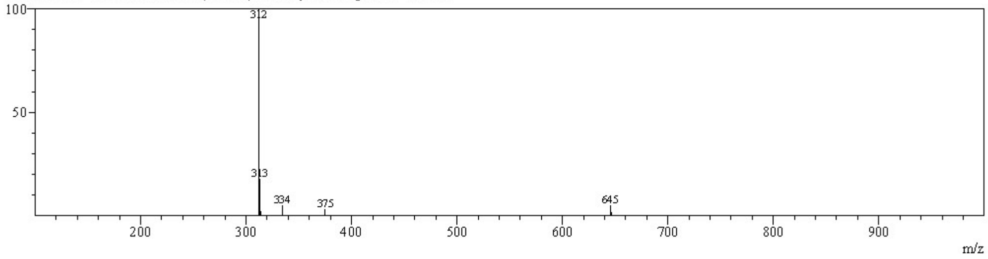

MS Spectrum Table

#:1 Ret.Time:
BG Mode:Calc 3.010<->3.310(302<->332)
Mass Peaks:7 Base Peak:312.10(593218) Polarity:Pos Segment1 - Event1

#	m/z	Abs.Inten.	Rel.Inten.	Charge	Polarity	Monoisotopic
1	312.10	593218	100.00			
2	313.10	104168	17.56			
3	314.05	12675	2.14			
4	334.05	27622	4.66			
5	375.10	18532	3.12			
6	645.25	28444	4.79			
7	646.25	10646	1.79			

Figure S131. LCMS spectrum of compound **43** (NPD-3280).

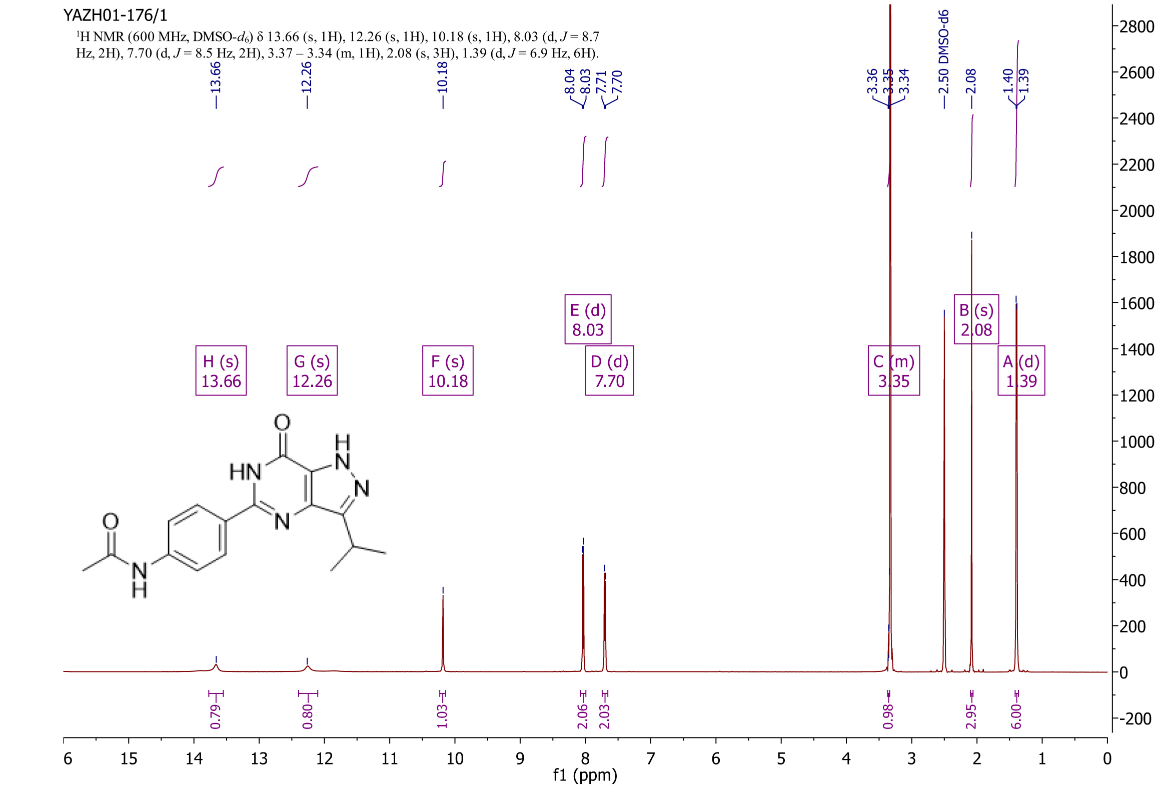

Figure S132. ^{1}H NMR spectrum of compound **43** (NPD-3280).

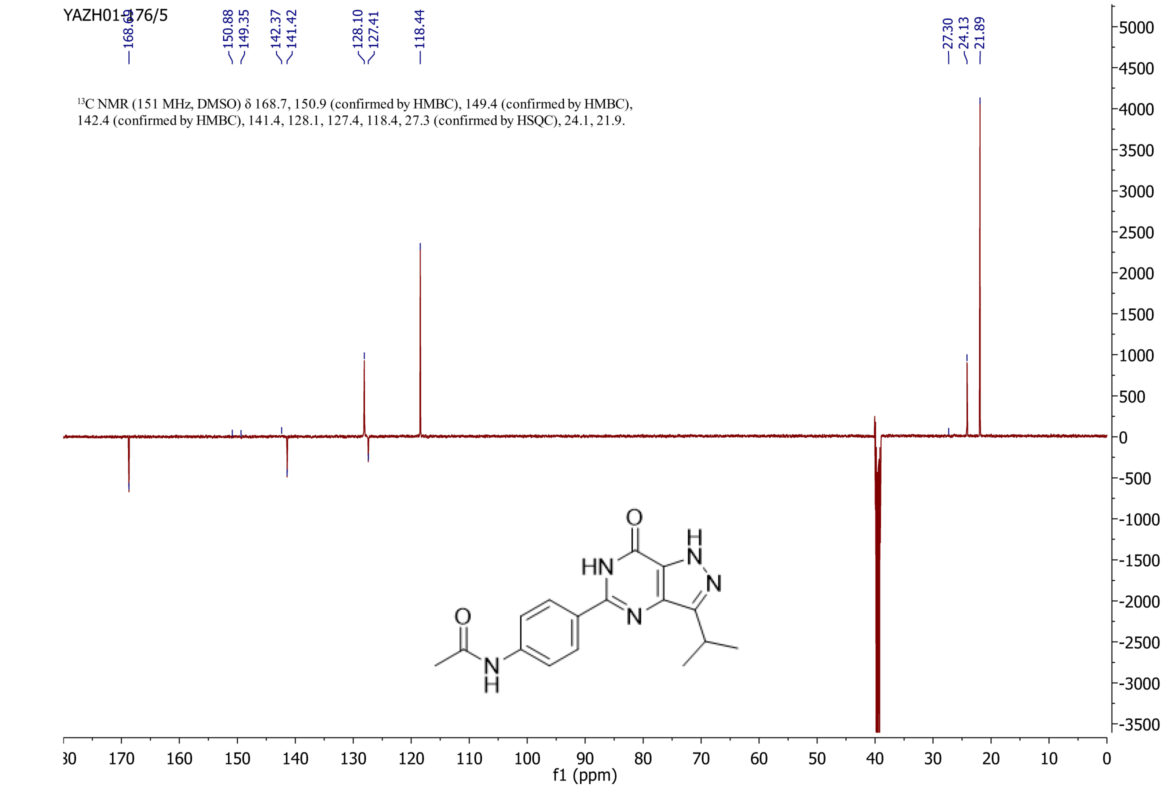

Figure S133. ^{13}C NMR spectrum of compound **43** (NPD-3280).

Acquired by	: Admin
Date Acquired	: 18/6/2019 3:48:26 PM
Sample Name	: VUF16503
Sample ID	:
Tray#	: 1
Vial#	: 36
Injection Volume	: 5
Data File	: C:\LabSolutions\Data\2019\2019-wk25\VUF16503.lcd
Background File	: blanco 18062019.lcd
Method File	: Method SCAN ACID standard.lcm
Report Format	: DefaultLCMS.lcr
Tuning File	: C:\LabSolutions\Tuning\Tuning-ESI-pos-neg01072015a.lct
Processed by	: Admin
Modified Date	: 18/6/2019 4:24:20 PM

PDA Graph

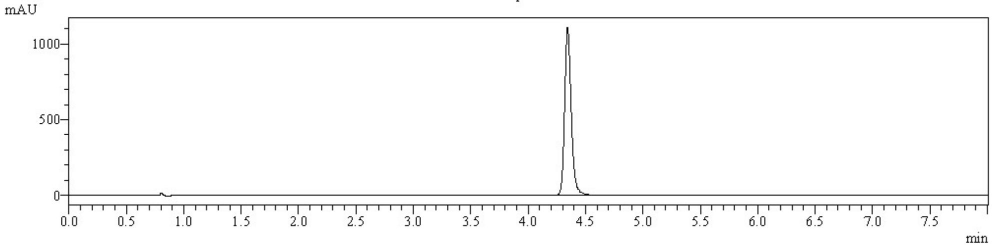

PeakTable

PDA Ch1 254nm 4nm

Peak#	Name	Ret. Time	Area	Area%
1		4.339	4313859	100.000

MS Chromatogram
VUF16503 C:\LabSolutions\Data\2019\2019-wk25\VUF16503.lcd

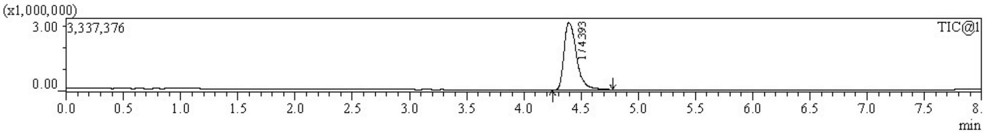

MS Spectrum Graph

#:1 Ret.Time:Averaged 4.380-4.400(Scan#:439-441)
BG Mode:Calc 4.250<->4.780(426<->479)
Mass Peaks:8 Base Peak:338.10(2315029) Polarity:Pos Segment1 - Event1

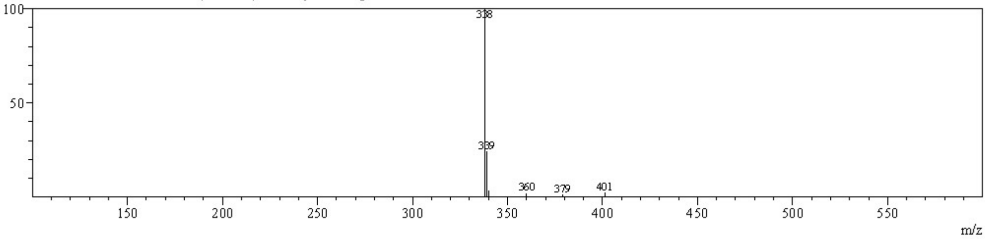

MS Spectrum Table

#:1 Ret.Time:
BG Mode:Calc 4.250<->4.780(426<->479)
Mass Peaks:8 Base Peak:338.10(2315029) Polarity:Pos Segment1 - Event1

#	m/z	Abs.Inten.	Rel.Inten.	Charge	Polarity	Monoisotopic
1	338.10	2315029	100.00			
2	339.15	558399	24.12			
3	340.15	69830	3.02			
4	360.05	44771	1.93			
5	379.15	26705	1.15			
6	401.15	51029	2.20			
7	697.40	39606	1.71			
8	702.95	24657	1.07			

Figure S134. LCMS spectrum of compound **44** (NPD-3283).

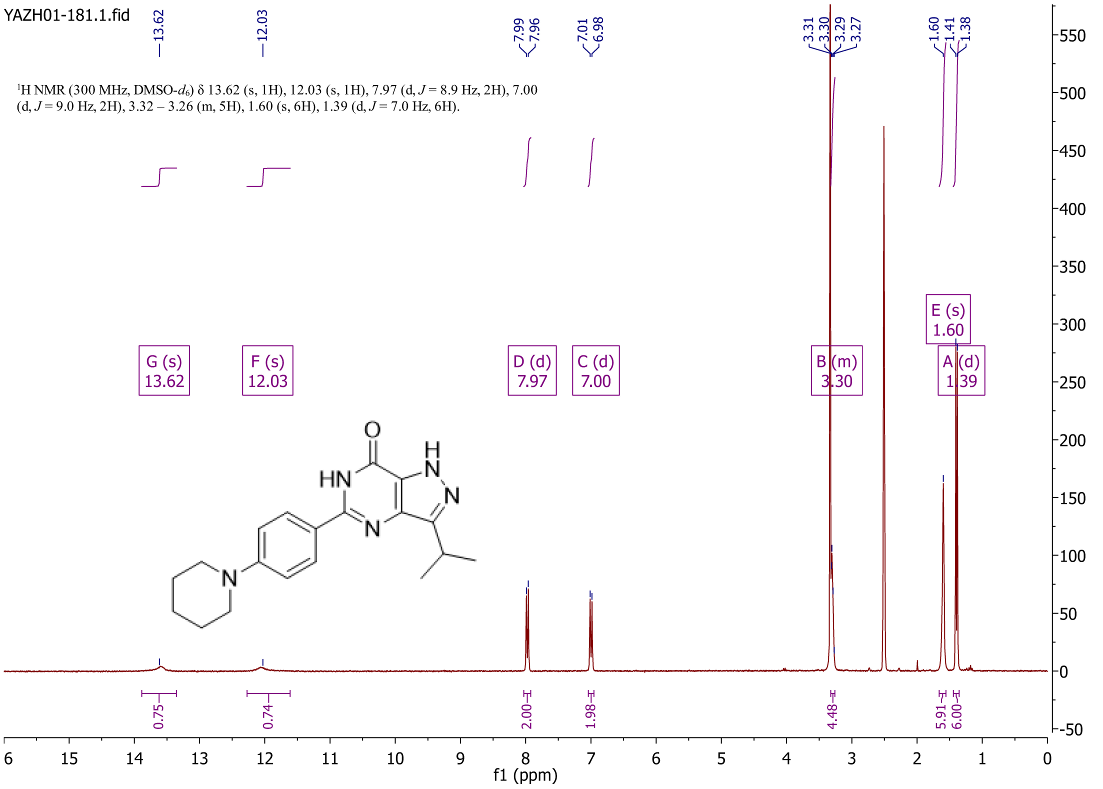

Figure S135. ¹H NMR spectrum of compound **44** (NPD-3283).

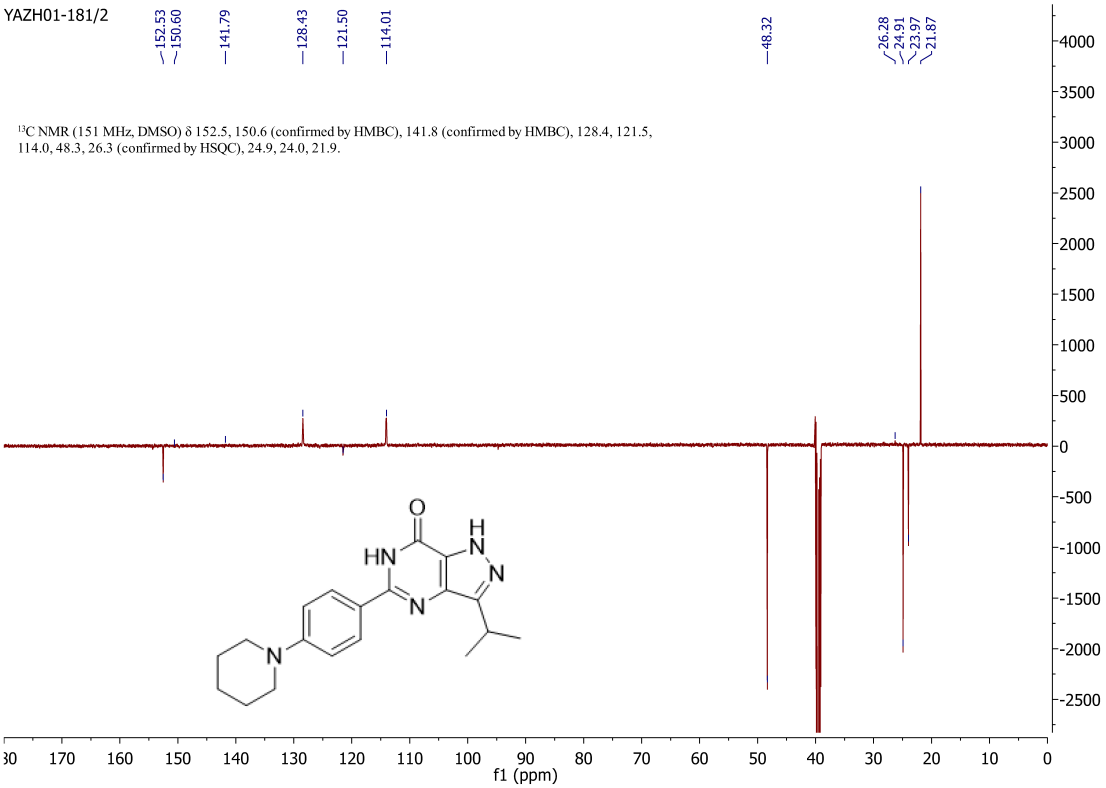

Figure S136. ¹³C NMR spectrum of compound **44** (NPD-3283).

Acquired by : Admin
Date Acquired : 12/3/2018 11:54:17 AM
Sample Name : YAZH01-179
Sample ID :
Tray# : 1
Vial# : 8
Injection Volume : 5
Data File : C:\LabSolutions\Data\2018\2018-wk11\YAZH01-179.lcd
Background File : blanco_120318.lcd
Method File : Method SCAN ACID standard.lcm
Report Format : DefaultLCMS.lcr
Tuning File : C:\LabSolutions\Tuning\Tuning-ESI-pos-neg01072015a.lct
Processed by : Admin
Modified Date : 7/6/2019 5:56:39 PM

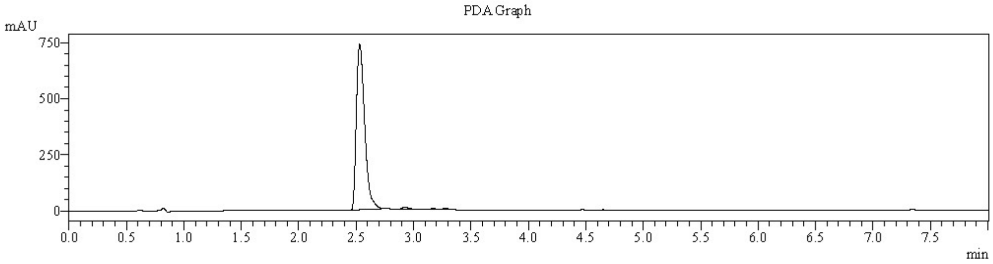

PeakTable

PDA Ch1 254nm 4nm

Peak#	Name	Ret. Time	Area	Area %
1		2.526	3685980	98.260
2		2.922	37216	0.992
3		3.163	13353	0.356
4		3.272	14686	0.392

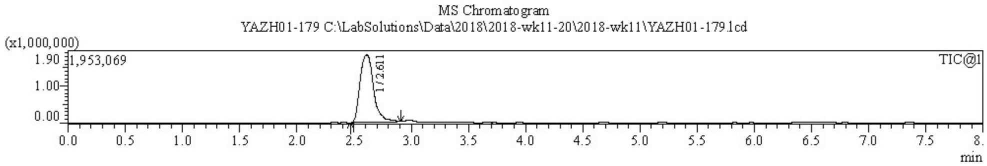

MS Spectrum Graph

#1 Ret.Time:Averaged 2.600-2.620(Scan#:261-263)
BG Mode:Calc 2.470<->2.910(248<->292)
Mass Peaks:8 Base Peak:353.15(1236663) Polarity:Pos Segment1 - Event1

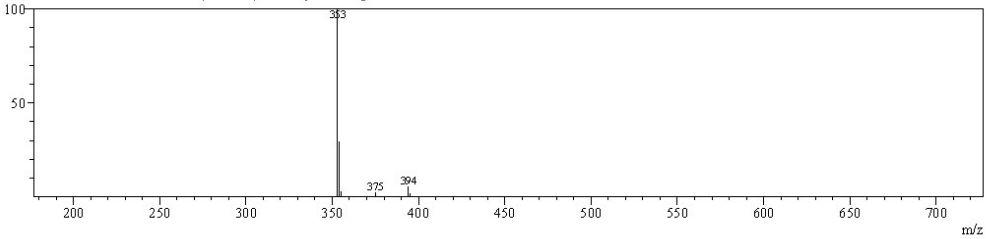

MS Spectrum Table

#:1 Ret.Time:
BG Mode:Calc 2.470<->2.910(248<->292)
Mass Peaks:8 Base Peak:353.15(1236663) Polarity:Pos Segment1 - Event1

#	m/z	Abs.Inten.	Rel.Inten.	Charge	Polarity	Monoisotopic
1	176.95	20460	1.65			
2	353.15	1236663	100.00			
3	354.15	365052	29.52			
4	355.15	31125	2.52			
5	375.20	26493	2.14			
6	394.20	66244	5.36			
7	395.20	19763	1.60			
8	727.35	20062	1.62			

Figure S137. LCMS spectrum of compound **45** (NPD-3282).

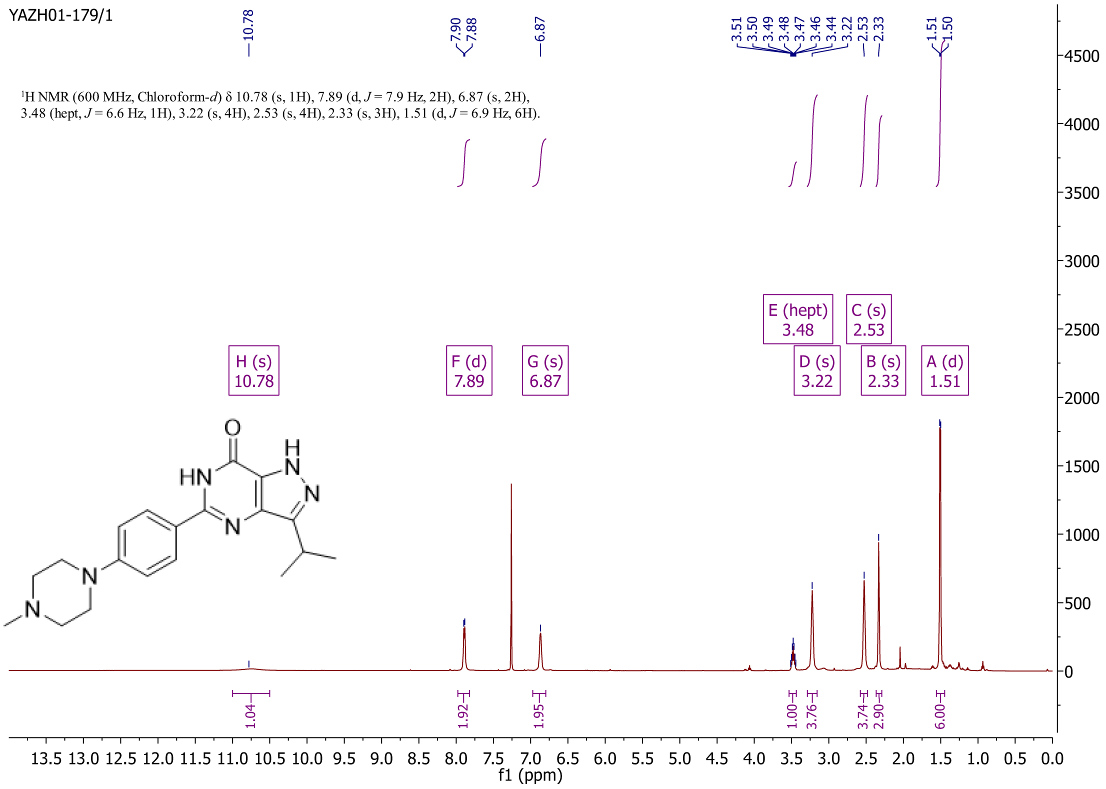

Figure S138. ¹H NMR spectrum of compound **45** (NPD-3282).

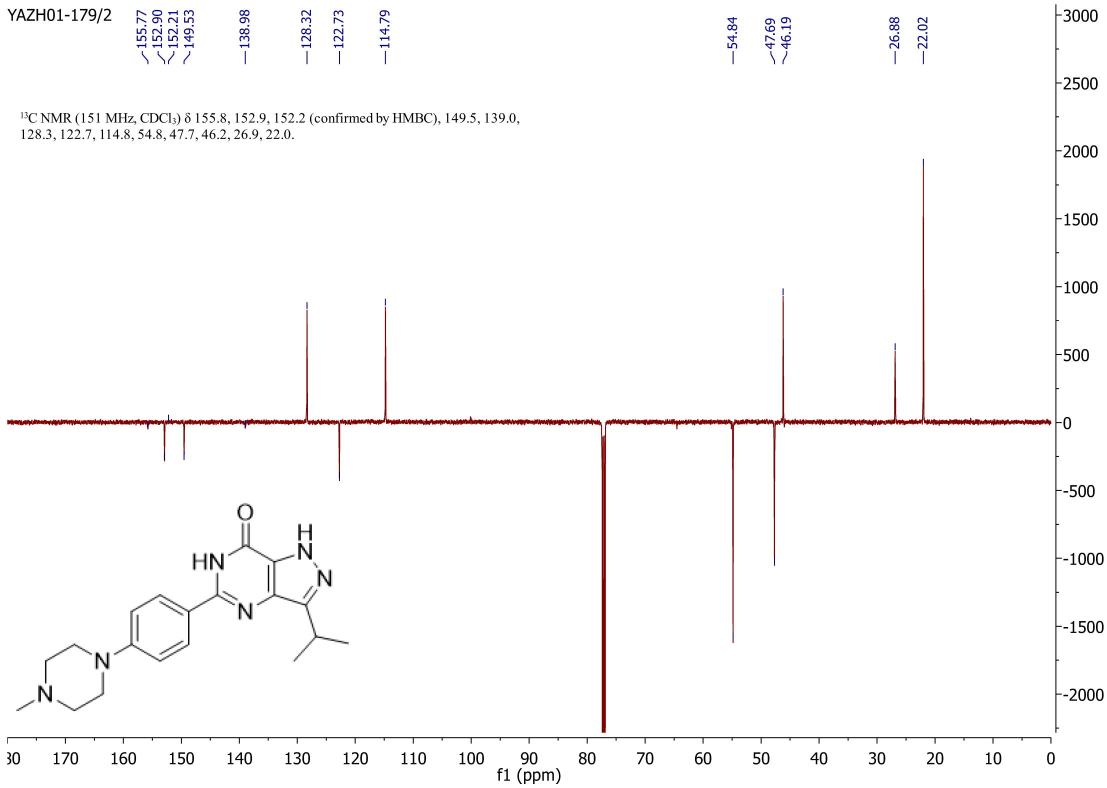

Figure S139. ¹³C NMR spectrum of compound **45** (NPD-3282).

Acquired by : Admin
Date Acquired : 17/5/2018 4:25:17 PM
Sample Name : YAZH01-212_LCMS
Sample ID :
Tray# : 1
Vial# : 11
Injection Volume : 6
Data File : C:\LabSolutions\Data\2018\2018-wk20\YAZH01-212_LCMS-1.lcd
Background File : blanco 170518.lcd
Method File : Method SCAN ACID standard.lcm
Report Format : DefaultLCMS.lcr
Tuning File : C:\LabSolutions\Tuning\Tuning-ESI-pos-neg01072015a.lct
Processed by : Admin
Modified Date : 17/5/2018 6:30:24 PM

PDA Graph

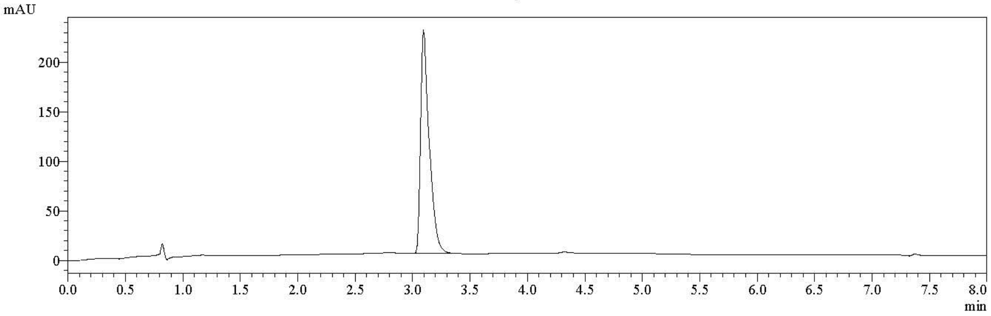

PDA Ch1 254nm 4nm

Peak#	Name	Ret. Time	Area	Area%
1		3.091	1176731	100.000

MS Spectrum Graph

#:1 Ret.Time:Single 3.180(Scan#:319)
Mass Peaks:10 Base Peak:323.15(35241) Polarity:Pos Segment1 - Event1

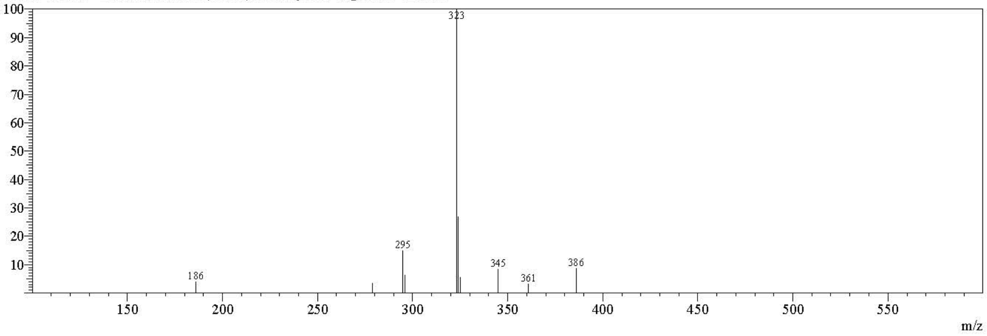

MS Spectrum Table

#:1 Ret.Time:
BG Mode:None
Mass Peaks:10 Base Peak:323.15(35241) Polarity:Pos Segment1 - Event1

#	m/z	Abs.Inten.	Rel.Inten.	Charge	Polarity	Monoisotopic	#	m/z	Abs.Inten.	Rel.Inten.	Charge	Polarity	Monoisotopic
1	185.95	1430	4.06				6	324.05	9489	26.93			
2	278.85	1179	3.35				7	325.05	1972	5.60			
3	294.80	5287	15.00				8	345.00	2941	8.35			
4	295.80	2225	6.31				9	360.95	1155	3.28			
5	323.15	35241	100.00				10	385.95	3045	8.64			

Figure S140. LCMS spectrum of compound **46** (NPD-3490).

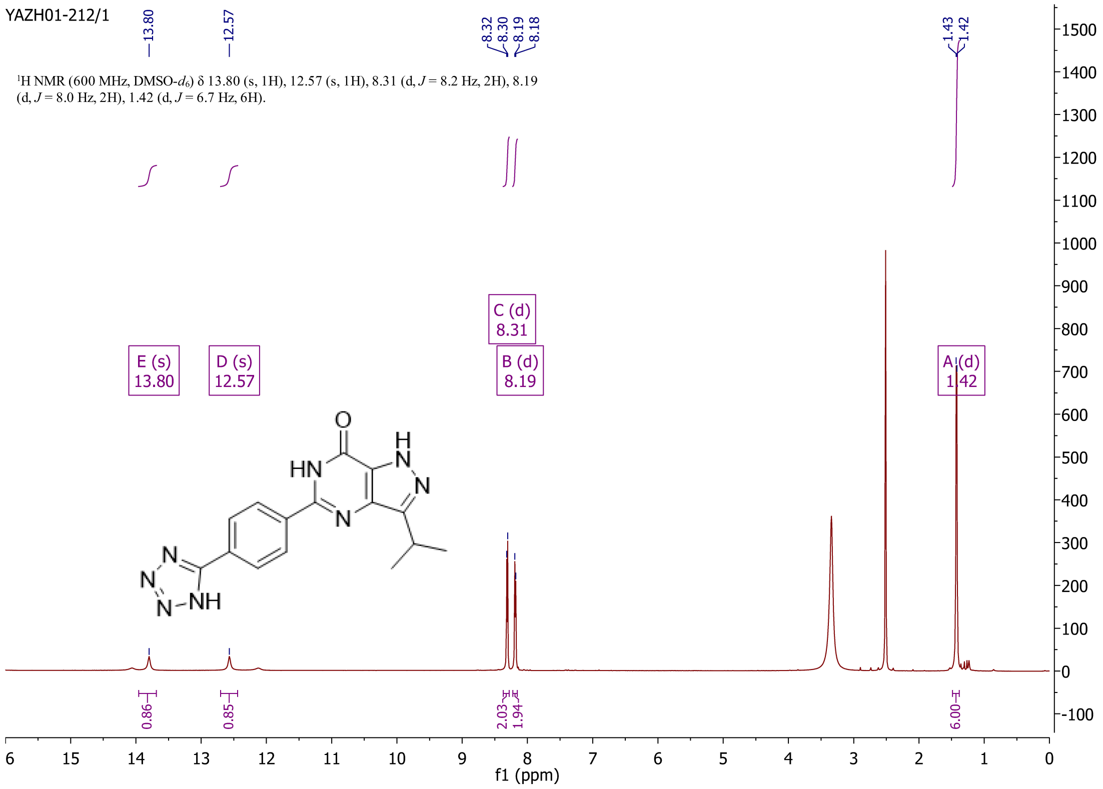

Figure S141. ^{1}H NMR spectrum of compound **46** (NPD-3490).

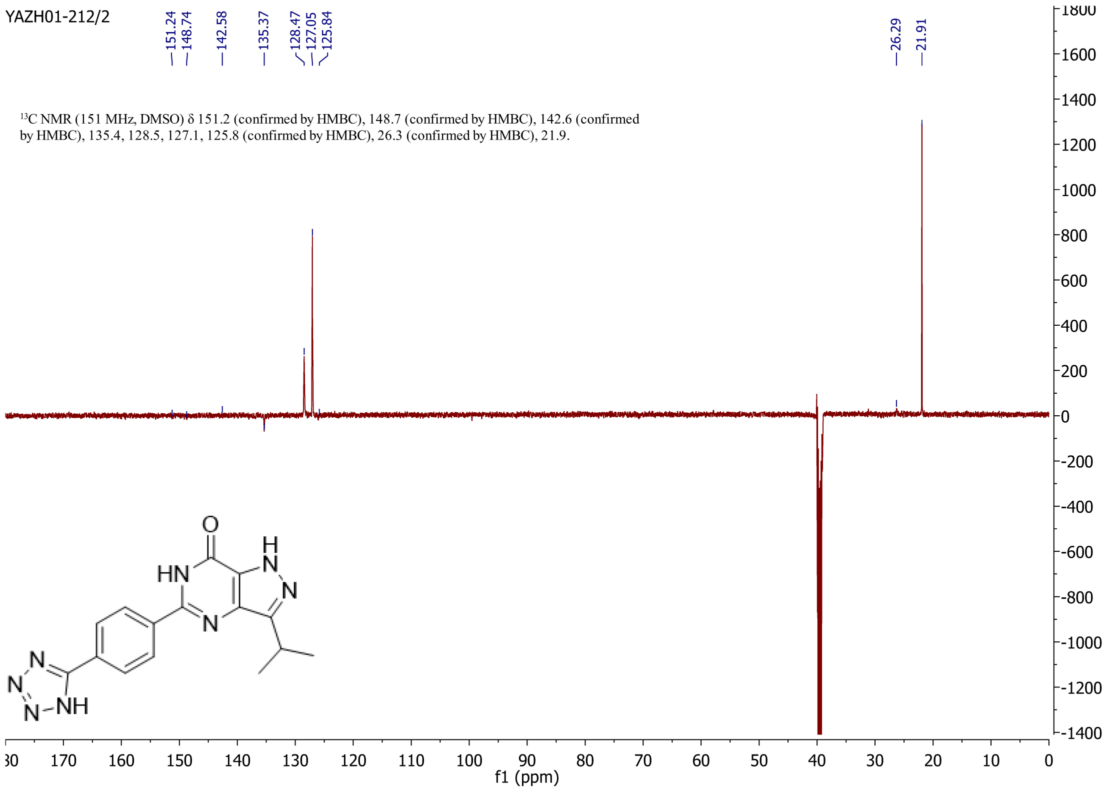

Figure S142. ^{13}C NMR spectrum of compound **46** (NPD-3490).
